# Effects of Lateral and Terminal Chains of X-Shaped Bolapolyphiles with Oligo(phenylene ethynylene) Cores on Self-Assembly Behaviour. Part 1: Transition between Amphiphilic and Polyphilic Self-Assembly in the Bulk

**DOI:** 10.3390/polym9100471

**Published:** 2017-09-26

**Authors:** Silvio Poppe, Marco Poppe, Helgard Ebert, Marko Prehm, Changlong Chen, Feng Liu, Stefan Werner, Kirsten Bacia, Carsten Tschierske

**Affiliations:** 1 Department of Chemistry, Martin Luther University Halle-Wittenberg, Kurt-Mothes Str. 2, 06120 Halle, Germany; silviopoppe@googlemail.com (S.P.); marco.poppe@googlemail.com (M.P.); Helgard.Ebert@gmx.de (H.E.); marko.prehm@chemie.uni-halle.de (M.P.); 2 State Key Laboratory for Mechanical Behavior of Materials, Xi’an Jiaotong University, Xi’an 710049, China; Chenchanglong@stu.xjtu.edu.cn (C.C.); feng.liu@xjtu.edu.cn (F.L.); 3 Department of Chemistry, Martin Luther University Halle-Wittenberg, Kurt-Mothes Str. 3, 06120 Halle, Germany; stefan.werner@chemie.uni-halle.de (S.W.); kirsten.bacia@chemie.uni-halle.de (K.B.)

**Keywords:** self-assembly, amphiphiles, polyphiles, bolaamphiphiles, liquid crystals, honeycombs, fluorophobic effect, block molecules, oligo(phenylene ethynylene), oligo(ethylene oxide)

## Abstract

Polyphilic self-assembly leads to compartmentalization of space and development of complex structures in soft matter on different length scales, reaching from the morphologies of block copolymers to the liquid crystalline (LC) phases of small molecules. Whereas block copolymers are known to form membranes and interact with phospholipid bilayers, liquid crystals have been less investigated in this respect. Here, series of bolapolyphilic X-shaped molecules were synthesized and investigated with respect to the effect of molecular structural parameters on the formation of LC phases (part 1), and on domain formation in phospholipid bilayer membranes (part 2). The investigated bolapolyphiles are based on a rod-like π-conjugated oligo(phenylene ethynylene) (OPE) core with two glycerol groups being either directly attached or separated by additional ethylene oxide (EO) units to both ends. The X-shape is provided by two lateral alkyl chains attached at opposite sides of the OPE core, being either linear, branched, or semiperfluorinated. In this report, the focus is on the transition from polyphilic (triphilic or tetraphilic) to binary amphiphilic self-assembly. Polyphilic self-assembly, i.e., segregation of all three or four incorporated units into separate nano-compartments, leads to the formation of hexagonal columnar LC phases, representing triangular honeycombs. A continuous transition from the well-defined triangular honeycomb structures to simple hexagonal columnar phases, dominated by the arrangement of polar columns on a hexagonal lattice in a mixed continuum formed by the lipophilic chains and the OPE rods, i.e., to amphiphilic self-assembly, was observed by reducing the length and volume of the lateral alkyl chains. A similar transition was found upon increasing the length of the EO units involved in the polar groups. If the lateral alkyl chains are enlarged or replaced by semiperfluorinated chains, then the segregation of lateral chains and rod-like cores is retained, even for enlarged polar groups, i.e., the transition from polyphilic to amphiphilic self-assembly is suppressed.

## 1. Introduction

**Scheme 1:**
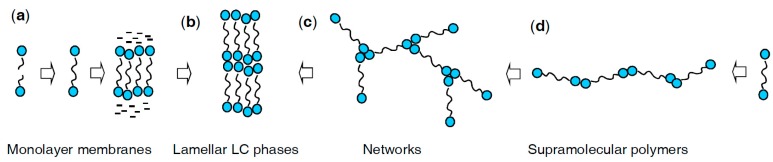
Modes of self-assembly of bolaamphiphiles.

Bolaamphiphiles are compounds composed of a lipophilic segment terminated at both ends by identical or different polar and hydrophilic (anionic, cationic, zwitterionic, nonionic) groups [1]. These compounds represent an important class of membrane-forming materials, emerging at the early stages of development of the biosphere, and still forming the cell membranes of archaea bacteria [2]. The high stability of the membranes formed by bolaamphiphiles is the result of the cooperativity of self-assembly due to the covalent end-to-end connection of pairs of lipids, thus forming monolayer membranes by lateral (side-by-side) aggregation in aqueous systems (Scheme 1a).

Bolaamphiphiles are also capable of forming supramolecular polymers and networks by terminal (end-to-end) aggregation (Scheme 1c,d) [3,4,5,6]. In the bulk, the combination of terminal and lateral aggregation leads to lamellar liquid crystalline (LC) phases with exceptional stability (Scheme 1b) [7,8]. In these monolayer lamellar phases, the central lipophilic units and the polar end groups are segregated into distinct layers, thus behaving as binary amphiphiles. The formation of either linear polymers or infinite layers mainly depends on the type of the polar groups and the directionality of their interactions. Directed hydrogen bonding, as for example found for linear carboxylic acid/pyridine complexes and other typical supramolecular hydrogen bonded recognition sites, favour polymer formation [5,9]. Reducing the strict directionality of the attractive intermolecular interactions by using conformationally flexible glycerol, carbohydrate, and oligo(ethylene oxide) (EO) units or ionic groups, allows simultaneous interactions between the end-groups into different directions, thus leading to branching with formation of nets (Scheme 1c), and finally, to the nano-segregation of polar and non-polar units with formation of lamellar LC phases (Scheme 1b) [10]. Stiffening of the lipophilic units, as for example by introduction of π-conjugated rods, favours the lamellar organizations [10], whereas additional lateral groups distort this mode of self-assembly, leading to networks and honeycombs.

**Figure 1:**
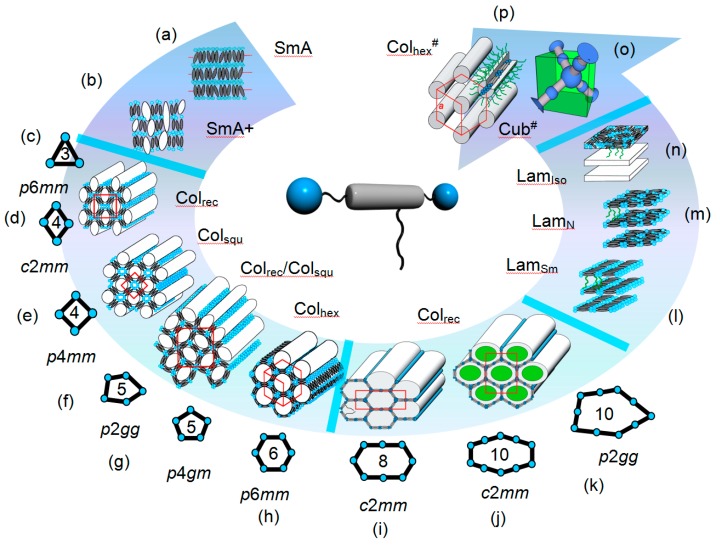
Liquid crystalline self-assembly of T-shaped bolapolyphiles. Besides the smectic phases (**a**,**b**) and the small polygonal honeycombs with triangular and quadrangular shape (**c**–**e**), considered here, also larger pentagonal and hexagonal honeycombs (**f**–**h**), giant honeycombs formed by end-to-end connected pairs of molecules (**i**–**k**), special types of lamellar phases with coplanar rod alignment (**l**–**n**), as well as cubic network phases (**o**) and columnar phases involving coaxial rod-bundles (**p**) are known [15,17].

**Scheme 2:**
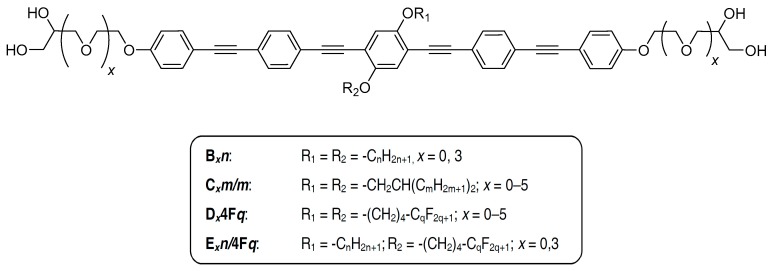
Structures of the compounds under investigation and their notation; the subscript *x* is only used if *x* ≠ 0; *n*, *m* indicate the length of alkyl chains, whereas *q* is the number of fluorinated carbons; the glycerol groups are racemic, i.e., all compounds represent racemic mixtures of diastereomers.

Prominent examples of these laterally substituted compounds are T-shaped and X-shaped bolapolyphiles composed of a rod-like π-conjugated core, terminally functionalized by hydrogen bonding glycerol groups at each end, and carrying one (T-shaped) [11,12] or two lipophilic lateral chains at opposite sides of the rod-like unit (X-shaped) [13]. These flexible lateral chains cause a frustration of the layer-like organization for steric reasons (Figure 1a,b). Moreover, the flexible chains are more or less incompatible with the rigid rod-like cores, thus tending to organize in distinct compartments, and therefore, these molecules are considered as bolapolyphiles [14,15,16,17]. The bulk self-assembly of these compounds is complex, and a huge variety of distinct LC phases can be observed, as shown in Figure 1. The typical feature of their self-assembly is the formation of polygonal honeycombs where the polar end groups form columns which are interconnected by the rod-like cores, thus forming a network with 2D periodicity in the *a-b* plane and being expanded into the third dimension *c* (Figure 1c–k) [14,15,16,17]. Depending on the ratio between chain volume and space available in the prismatic cells (mainly determined by the length of the rod-like units), the cross-sectional shape of the prismatic cells changes. As observed experimentally [10,11,12,13,14,15,16,17] and supported by simulations [18,19,20,21], there is a transition from rhombic or triangular via square, pentagonal to hexagonal cells with growing lateral chain volume or with decreasing rigid core length. Occasionally, complex tiling patterns composed of cells with different shapes were found [22], especially if different lateral chains (e.g., fluorinated and non-fluorinated) were combined [23,24,25]. For lateral groups with further increased volume, giant honeycombs (Figure 1i–k) [25,26,27] and lamellar phases with coplanar organization of rods (Lam) (Figure 1l–n) were observed [28,29,30,31]. For compounds with branched lateral chains, coaxial rod-bundle phases with cubic network structure (Figure 1o) [32,33] or forming columns on a hexagonal lattice were reported (Figure 1p) [34,35]. Some of these structures can be considered as soft self-assembled supramolecular analogues of the porous solid-state materials based on covalent organic frameworks (COFs) [36,37,38]. The observed tiling patterns are also similar to self-assembled 2D-nets formed on and stabilized by solid surfaces [39,40]. In the LC phases discussed here, these nets are expanded in the third dimension, with formation of honeycombs. The transition from 2D to 3D connectivity provides cooperativity [41,42], and therefore, surface stabilization is not required for these LC phases to be thermodynamically stable. Herein we focus on X-shaped bolapolyphiles involving a relatively long oligo(phenylene ethynylene) (OPE) core (Scheme 2) [23,43,44,45,46,47]. For these compounds with long rigid cores, the prismatic cell size of the honeycombs is limited to triangular [48,49] and square shapes [50], even if very long and bulky lateral chains are used. These large molecules with molecular weights between 1 and 2 kg·mol^−1^ could be considered as triblock rod-coil oligomers with strictly uniform size distribution [16,51].

Formation of complex morphologies is also known for linear and star-shaped multiblock copolymers, being flexible high molecular mass analogs of the block molecules mentioned above [52,53,54]. Triangular honeycomb morphologies have been observed for flexible star-shaped triblock copolymers [55], as well as for other types of low molecular weight rod–coil polyphiles, namely the facial amphiphiles [56,57]. Highly flexible bolaamphiphilic triblock copolymers (ABA triblocks), such as pluronics, i.e., FEO-PPO-PEO (poly(ethylene oxide)-poly(propylene oxide)-poly(ethylene oxide)) are known to interact with lipid membranes by polar interactions of the EOs with the polar head groups, and insertion of the PPO blocks into the inner lipophilic part of the bilayers, provided they are in the liquid crystalline state. This often leads to non-specific membrane compartmentalization [58]. Similar effects were observed for other types of block copolymers [59]. Replacing the flexible polymers and oligomers by amphiphiles or polyphiles involving rigid rod-like units could possibly provide more specific effects on the membranes, if the lipophilic part fits with the membrane dimensions [60,61,62,63,64,65,66,67,68]. Therefore, the length of the OPE core (*L*_core_, ~3.1 nm) involved in the considered X-shaped bolapolyphiles was chosen to correspond approximately to the diameter of the lipophilic layer of phospholipid biomembranes (~3.0 nm). The laterally attached alkyl chains (linear or branched) were expected to improve the compatibility of the rods with the alkyl chains of the phospholipids, whereas replacing the alkyl chains by semiperfluorinated chains is assumed to modify the interaction with the phospholipids [69,70]. If incorporated into lipid membranes, these X-shaped bolapolyphiles could provide membrane stabilization or destabilization (channel formation) [71], domain formation [72,73,74,75] and membrane compartmentalization [76] and might contribute to the knowledge about the effects of rod-like molecules (e.g., cholesterol) [75] or superstructures (α-helices of integral proteins) [77,78] on the membrane properties. Furthermore, ordered organizations of π-conjugated systems in lipid membranes are of potential interest for artificial light harvesting systems [79] and transmembrane electronic conduction [80]. In previous work it was shown that the OPE based X-shaped bolapolyphile **B12**, having two linear dodecyl chains, can form unique hexagonal star shaped domains in the phospholipid model membranes (DPPC, DOPC) of giant unilamellar vesicles (GUVs), obviously stabilizing these vesicles by avoiding faceting at the L_α_-L_β_′ transition [43,44,45].

The purpose of the work presented here is twofold, and therefore, it is split into two parts, presented in two back-to-back papers. The first part focuses on a deeper understanding of the effects of molecular structure, namely side chain architecture (chain length, chain branching, fluorination) and terminal polar group structure (number of EO units) on the self-assembly of X-shaped bolapolyphiles in LC bulk systems. The main focus here is on the investigation of the transition between amphiphilic and polyphilic modes of self-assembly. In the second part [81], selected examples of these compounds were investigated with respect to their domain formation in the lipid double layers of GUVs by means of laser confocal fluorescence microscopy. The focus of that part is on the investigation of the effects of the molecular structure on domain formation.

The distinct groups of X-shaped bolapolyphiles **B**–**E** under investigation are shown in Scheme 2. These include the previously reported X-shaped compounds, **B*n***, having two glycerol end groups, and two identical linear lateral alkyl chains, with chain length *n* = 4–12 and 18, at opposite sides of the OPE core [43,44,45,46], and the related compound, **B_3_12**, with three additional EO units between the rod-like core and the glycerol units [43]. The new series of compounds, **C**, is characterized by having two branched lateral alkyl chains at opposite sides of the OPE core [47]. For compounds **C*_x_m/m***, the length of all four branches (*m*) is identical (*m* = 2–18), and the total number of carbons *n* in each lateral group, including the –OCH_2_– linking group and the ternary CH group at the branching point, is *n* = 2*m* + 2. For all compounds, absence of subscript *x* indicates compounds with simple glycerol groups, and the subscript *x* is used for compounds involving additional EO units (*x* = 1–5). In the series **D** and **E**, both chains or only one *n*-alkyl chain of the X-shaped compounds **B*_x_n*** were partly fluorinated, respectively. In the detailed designation of compounds **D*_x_*4F*q*** and **E*_x_n*/4F*q***, the number **4** indicates the length of the aliphatic spacer unit between core and perfluorinated segment (–(CH_2_)_4_)–), and ***n*** is the length of the alkyl chain in compounds **E*_x_n*/4F*q***; **F*q*** indicates the number of carbons in the fluorinated segment of the semiperfluorinated chain(s). Most of these fluorinated compounds are derived from **B*_x_*12**, in which one or both dodecyl (–C_12_H_25_) chains is replaced by –(CH_2_)_4_C_6_F_13_ chains (compounds **E_3_12/4F9**, **D4F9** and **D_1_4F9**–**D_5_4F9**). The –(CH_2_)_4_C_6_F_13_ chain, involving only 10 carbons in total, is used because it has approximately the same volume as the aliphatic dodecyl chain.

## 2. Materials and Methods

### 2.1. Synthesis

**Scheme 3:**
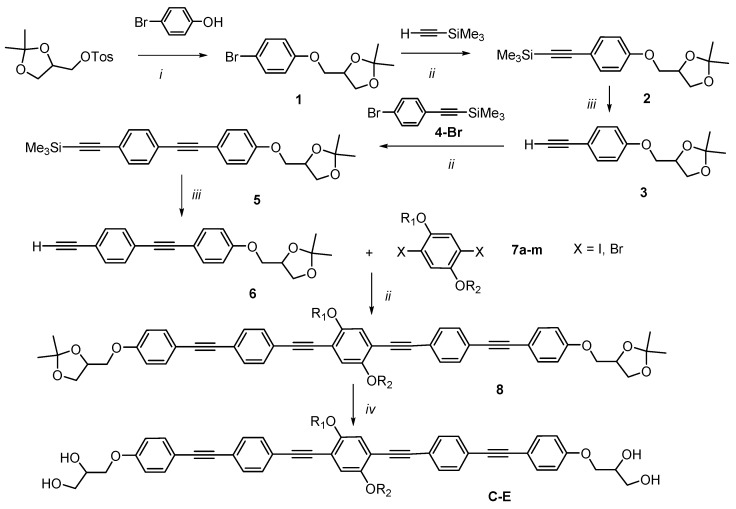
Synthesis of compounds **C**–**E** with simple 1-glycerol end groups; for details of the structures of R_1_ and R_2_, see Scheme 2. Reagents and conditions: (i) K_2_CO_3_, DMF, 120 °C, 6 h, 93%; (ii) [Pd(PPh_3_)_4_], CuI, NEt_3_, reflux, 6 h, 80–90%; (iii) K_2_CO_3_, CH_2_Cl_2_/MeOH (2:1), 20 °C, 2 h, 60–65%; (iv) PPTS, MeOH/THF (1:1), 50 °C, 12 h, 40–60%.

**Scheme 4:**
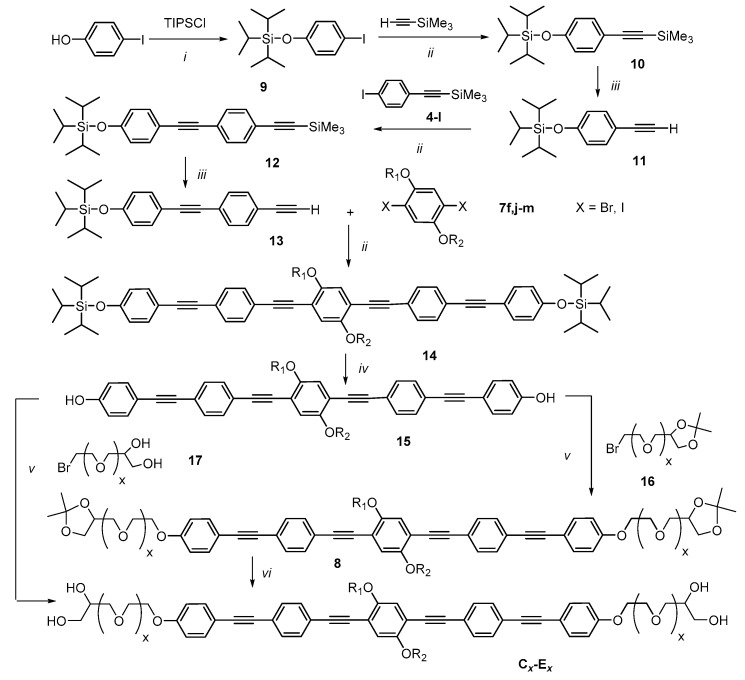
Synthesis of the bolapolyphiles **C*_x_***–**E*_x_*** involving EO units in the polar groups; for details of the structures of R_1_ and R_2_, see Scheme 2. Reagents and conditions: (i) Imidazole, CH_2_Cl_2_, 20 °C, 12 h, 56%; (ii) [Pd(PPh_3_)_4_], CuI, NEt_3_, reflux, 6 h, 72%; (iii) K_2_CO_3_, CH_2_Cl_2_/MeOH (2:1), 20 °C, 2 h, 95%; (iv) Bu_4_NF, THF, 20 °C, 2 h , 40–50%; (v) K_2_CO_3_, DMF, 120 °C, 6 h, 80–90%; (vi) PPTS, MeOH/THF (1:1), 50 °C, 12 h, 60–80% (for structures of **7f, j**–**m**, see Supporting Information).

The synthesis of compounds **B_x_*n*** has been reported previously [43,44,45,46]. The synthesis of all other bolapolyphiles **C**–**E** was conducted via series of repetitive Sonogashira cross-coupling reactions [82] and required protection/deprotection procedures, as shown in Scheme 3 and Scheme 4.

The synthesis of the glycerol terminated compounds **C**–**E** (*x* = 0, Scheme 3) starts with 4-bromo(2,3-*O*-isopropylidene-2,3-dihydroxypropyl-1-oxy)benzene (**1**) [83], which is coupled with trimethylsilylacetylene to yield compound **2** [84]. After K_2_CO_3_ catalyzed desilylation [85], the obtained 4-ethynyl(2,3-*O*-isopropylidene-2,3-dihydroxypropyl-1-oxy)benzene (**3**) [43] was coupled with 4-bromo(trimetylsilylethynyl)benzene (**4-Br**) [86] to yield the 4′-(2,3-*O*-isopropylidene-2,3-dihydroxypropyl-1-oxy) substituted 4-etynyltolane **6** after desilylation [43]. Two equivalents of **6** were coupled with the appropriately functionalized 2,5-dibromo- or 2,5-diiodohydroquinone ethers **7** [87,88], giving the bolapolyphiles **C**–**E** after deprotection of the 1,2-diol groups with pyridinium *p*-toluenesulfonate (PPTS) in methanol and THF [47,89]. For the synthesis of the diiodohydroquinone ethers **7a**–**i** with two identical branched chains (X = I, R_1_ = R_2_ = –CH_2_CH(C*_m_*H_2*m*+1_)_2_), the branched alkyl bromides were obtained from diethyl malonate by a sequence of alkylation [90], dealkoxycarbonylation [91], reduction [92], and bromination [93], were etherified with 2,5-diiodohydroquinone (Scheme S1) [94]. The diiodohydoquinone ether **7k**, with two semiperfluorinated chains (X = I, R_1_ = R_2_ = –(CH_2_)_4_C_6_F_13_), was prepared in an analogous way using tridecafluorodecyl bromide (Schemes S2 and S3) [92,93,95]. For the synthesis of the non-symmetric dibromohydroquinone ethers **7l, m** (X = Br, R_1_ = –C*_n_*H_2*n*+1_, R_2_ = –(CH_2_)_4_C_6_F_13_) 4-benzyloxy-2,5-dibromophenol was alkylated with the appropriate alkyl bromide first, the benzyloxy group was cleaved by catalytic hydrogenation and then the semiperfluorinated chain was attached by alkylation of the phenolic OH group with tridecafluorodecyl bromide (Scheme S4).

For the synthesis of the EO containing compounds **C*_x_***–**E*_x_***, the OPE core is built up first starting from T*i*PS protected 4-bromophenol (**9**) [96], followed by subsequent Sonogashira coupling with trimetylsilylacetylene and 4-iodo(trimethylsilylethynyl)benzene (**4-I**) [86] and protodesilylations. The thus obtained 4′-triisopropylsilyloxysubstituted 4-ethynyltolane **13** [43] was then coupled with the appropriate 2,5-dihalohydroquinonediether **7** [87], and desilylated using fluoride catalysis [97] to yield the OPE derived biphenol **15**. Compound **15** was then used for Williamson etherification with the bromides **16** having an isopropylidene protected 1,2-diol group [98,99,100]. Deprotection of the bisacetonides **8** [101] yields the desired compounds **C*_x_m/m*** (Scheme 3 and Scheme 4). For the synthesis of the compounds **D*_x_*4F*q*** and **E*_x_n*/4F*q*** with *x* ≥ 1 and having fluorinated lateral chains, the bromides **17** with a non-protected 1,2-diol group (for the synthesis of **16** and **17**, see Scheme S5) were used for the final alkylation step. Procedures and the analytical data of the newly synthesized X-shaped bolapolyphiles are collated in the Supporting Information together with representative NMR spectra (Figures S9–S30).

### 2.2. Methods

**Optical Polarizing Microscopy (POM)**. Transition temperatures were measured using a Mettler FP-82 HT hot stage and control unit in conjunction with a Leica DMRXP polarizing microscope (Leica Microsystems GmbH, Wetzlar, Germany). Textures of the liquid crystalline mesophases were recorded with a Leica MC120 HD camera (Leica Microsystems GmbH, Wetzlar, Germany).

**DSC measurements**. DSC-thermograms were recorded on a DSC-7 (Perkin-Elmer GmbH & Co. KG, Überlingen, Germany) in 30 μL Al-pans with heating and cooling rates of 10 K·min^−1^, see Figures S1 and S2.

**X-ray scattering on powder-like and aligned samples**. X-ray investigations were carried out at Cu K_α_ line (λ = 1.54 Å) using a standard Coolidge tube source with a Ni-filter. The diffraction patterns were recorded with a 2D detector (Vantec 500, Bruker, Billerica, MA, USA), and the exposure time was 15 min for WAXS and 30 min for SAXS. Powder-like samples were prepared in 1 mm capillaries, the samples were held in a temperature-controlled oven, and the distance between the sample and the detector was 9.5 cm (WAXS) or 27.4 cm (SAXS). For aligned samples, a droplet of the sample on a glass substrate placed on a temperature-controlled heating stage was investigated. The droplets were prepared in the isotropic state and then cooled (rate: 1 K·min^−1^) to the measuring temperature. The distance between the sample and the detector was 9.0 cm (WAXS) or 26.8 cm (SAXS), and the beam was parallel to the substrate.

**Synchrotron X-ray diffraction**. High-resolution small-angle powder diffraction experiments were recorded on Beamline BL16B1 at Shanghai Synchrotron Radiation Facility, SSRF, Shanghai, China. Samples were held in evacuated 1 mm capillaries. A Linkam hot stage with a thermal stability within 0.2 °C was used, with a hole for the capillary drilled through the silver heating block and mica windows attached to it on each side. A MarCCD 165 detector was used. *q* calibration and linearization were verified using several orders of layer reflections from silver behenate and a series of *n*-alkanes. The measurement of the positions and intensities of the diffraction peaks were carried out using program, where experimental diffractograms were fitted using Gaussian shaped peaks. The diffraction peaks were indexed on the basis of their peak positions, and the lattice parameters and the plane/space groups were subsequently determined.

Numerical XRD data are collated in Tables S1–S3, wide angle scatterings are shown in Figures S6 and S7.

**Electron density reconstruction.** Once the diffraction intensities are measured and the corresponding plane group determined, 2D electron density maps can be reconstructed, on the basis of the general formula

*E*(*xy*) = Σ*_hkl_ F*(*hk*) × exp[i2π(*hx* + *ky*)]. (1)

Here, *F*(*hk*) is the structure factor of a diffraction peak with index (*hk*). It is normally a complex number and the experimentally observed diffraction intensity

*I*(*hk*) = K × *F*(*hk*) × F*(*hk*) = K × |*F*(*hk*)|^2^. (2)

Here, K is a constant related to the sample volume, incident beam intensity etc. In this paper, we are only interested in the relative electron densities, hence this constant is simply taken to be 1. Thus, the electron density

*E*(*xy*) = Σ*_hkl_* sqrt[*I*(*hk*)] × exp[i2π(*hx* + *ky*) + *φ_hk_*]. (3)

As the observed diffraction intensity *I*(*hk*) is only related to the amplitude of the structure factor |*F*(*hk*)|, the information about the phase of *F*(*hk*), *φ_hk_*, cannot be determined directly from experiment. However, the problem is much simplified when the structure of the ordered phase is centrosymmetric, and hence, the structure factor *F*(*hk*) is always real, and *φ_hk_* is either 0 or *π.*

This makes it possible for a trial-and-error approach, where candidate electron density maps are reconstructed for all possible phase combinations, and the “correct” phase combination is then selected on the merit of the maps, helped by prior physical and chemical knowledge of the system. This is especially useful for the study of nanostructures, where normally only a limited number of diffraction peaks are observed.

## 3. Results and Discussion

### 3.1. Effects of Variation of the Lipophilic Lateral Chains

**Figure 2:**
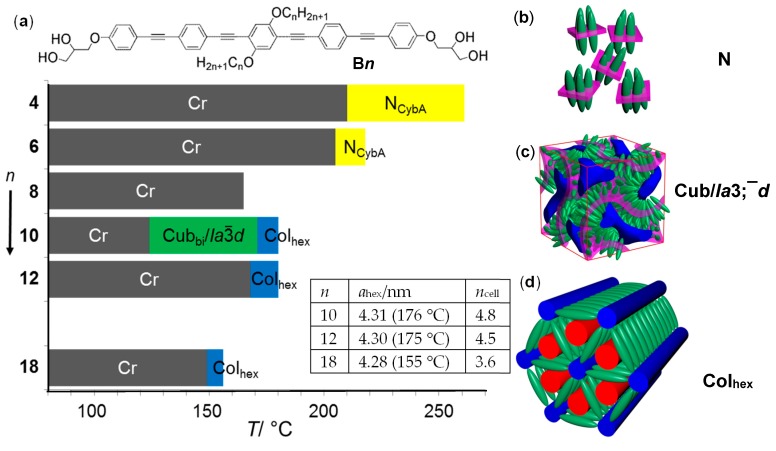
(**a**) Bar diagram showing the phase sequences of compounds **B*n***, depending on the alkyl chain length *n*, as recorded on cooling with a rate of 10 K·min^−1^; the hexagonal lattice parameter *a*_hex_, and the number of molecules in a unit cell (*n*_cell_) with a height *h* = 0.45 nm, corresponding to the maximum of the diffuse wide angle scattering, are shown for compounds **B10**, **B12**, and **B18** [46]; abbreviations: N = nematic LC phase; Col_hex_ = hexagonal columnar phase with *p*6*mm* plane group, representing a triangular LC honeycomb; Cub/*Ia3;¯d* = bicontinuous cubic phase with *Ia3;¯d* space group (gyroid); Cr = solid crystalline state; the isotropic liquid state (Iso) is at the right side of the bars; (**b**–**d**) shows models of the organization of compounds, **B*n***, in the distinct LC phases; the OPE rods are shown in all cases in green, whereas networks/columns of the glycerol groups (blue) and the lateral lipophilic chains (red) are only shown if they form long range ordered domains in (**c**,**d**); magenta indicates the minimal surface in (**c**) and the local smectic clusters in the (cybotactic) N phase in (**b**); (**d**) shows a structure with *n*_cell_ = 3, corresponding to *n*_wall_ = *n*_cell_/3 = 1 [46].

**Linear alkyl chains (B/*n*).** The effect of the length of linear alkyl chains on the LC self-assembly of X-shaped bolapolyphiles has been studied previously [43,44,45,46], and is summarized in Figure 2, for the purpose of comparison with the series of new compounds **C**–**E**. The general feature in this series is that elongation of the alkyl chains leads to a transition from a nematic phase (**B4**, **B6**) to a hexagonal columnar phase (**B10**–**B18**), with a gyroid type bicontinuous cubic phase (*Ia3;¯d* space group) as intermediate structure for **B10** [46].

In the nematic phase, there is only orientational order of the OPE cores, i.e., all three molecular building blocks are mixed, and no positional long-range order can develop (Figure 2b). Upon transition to the cubic phase (Figure 2c), the polar groups are separated into two interwoven networks (blue), and the space between them is filled by a continuum involving the mixed aromatic cores (green) and lateral alkyl chains (not shown). In this phase, the molecules behave like binary (bola)amphiphiles, forming only two distinct sets of compartments. All compounds with *n* = 12 to 18, and also compound **B10** at higher temperature, form hexagonal columnar phases with *a*_hex_ ~ 4.3 nm, in line with the formation of triangular honeycombs in all cases. This indicates that all three incompatible segments, the polar groups, the rod-like OPE cores and the lateral alkyl chains, segregate into their own domains (Figure 2d). Overall, lateral chain elongation first leads to emergence of binary polar–apolar amphiphilicity (N → Cub), which then turns to polyphilicity (triphilicity) as rigid–flexible amphiphilicity emerges in the Col_hex_ phase.

**Branched lateral chains (C*m*/*m*)**. Replacing linear by branched alkyl chains allows easy access to increased chain volume at restricted lateral extension of the chains if compared with related linear alkyl chains. The transition temperatures of the series **C*m/m***, with two identical branched lateral chains at opposite sides of the OPE core, are collated in Table 1.

**Figure 3:**
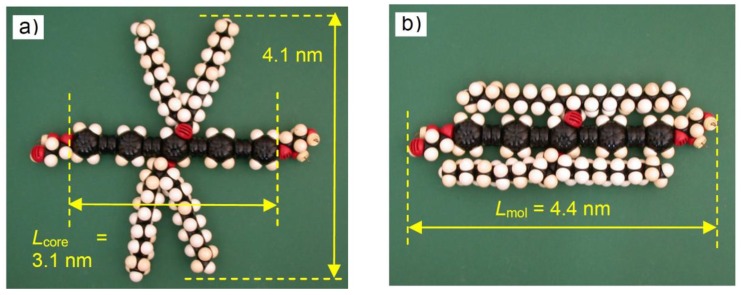
Molecular models showing (**a**) a star-shaped and (**b**) twinned H conformation of **C12/12** with molecular dimensions.

**Figure 4:**
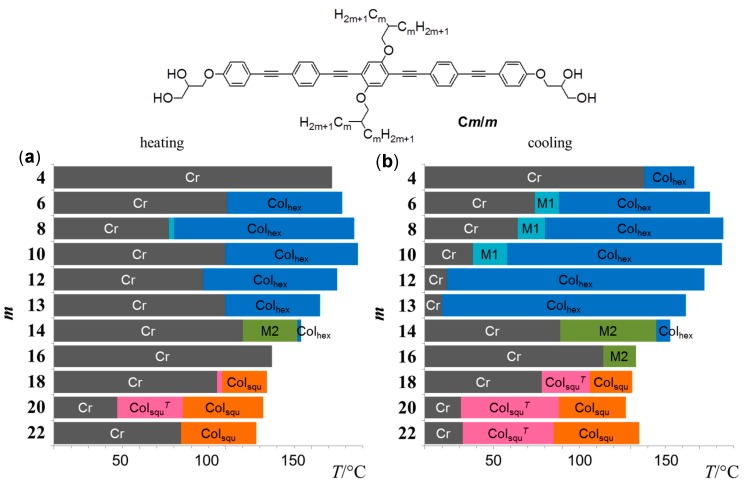
Bar diagrams showing the phase sequences and transition temperatures of compounds **C*m/m*** depending on the length *m* (**a**) as observed on heating and (**b**) on cooling with 10 K·min^−1^; for abbreviations, see Figure 2 and Table 1.

In the series **C*m/m***, the branching is close to the core unit, and the two branches are identical. This has a significant effect on chain conformation and chain alignment. Besides the star-shaped conformation (Figure 3a), an organization of the alkyl chains almost parallel to the aromatic OPE cores (twinned H-shape, Figure 3b) is easily possible. The chain length of the branches was changed from *m* = 2 to *m* = 22, corresponding to an increase of the total number of aliphatic carbons (both side chains together) from 12 to 92. The phase sequence depending on *m* is shown graphically in Figure 4. Depending on the chain length *m*, columnar LC phases, either with hexagonal (*m* = 4–14) or square symmetry (*m* = 18–22), are dominating. The square phases have previously been analyzed in detail [47], therefore, the focus is here on the Col_hex_ phases.

**Figure 5:**
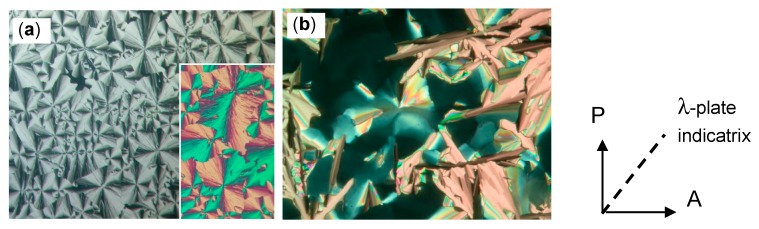
Representative textures of the Col_hex_ phases of compounds **C*m/m***, as observed between crossed polarizers between glass plates with approximately the same distance of 15 μm in (**a**,**b**); (**a**) **C4/4** at *T* = 165 °C, and (**b**) **C12/12** at *T* = 170 °C. The inset in (**a**) shows the texture with additional λ-retarder plate, the blue-shifted fans (green color) are parallel to the main indicatrix direction and thus define the orientation of the high-index axis as radial rather than tangential within the spherulitic domains. Because in these spherulites the columns are known to be tangential (forming cycles around the central defect), and the high-index axis parallel to the OPE long axis, it follows that the OPEs are perpendicular to the columns; this indicates negative birefringence. At the right, the orientation of polarizer (P) and analyzer (A) and the slow axis orientation of the λ-plate are shown.

Representative textures of the Col_hex_ phases, as observed by microscopic investigation between crossed polarizers, are shown in Figure 5. The spherulitic textures indicate columnar LC phases, and the dark areas indicate regions with an alignment of the columns perpendicular to the substrate surfaces. That these regions are optically isotropic confirms uniaxiality of these columnar phases, i.e., the lattice can have square or hexagonal symmetry. Investigations with a λ-retarder plate indicate that the intramolecular π-conjugation pathway is perpendicular to the column long axis (northeast and southwest areas of the spherulites, being parallel to the indicatrix slow axis, are blue-shifted; see inset in Figure 5a), being in line with a honeycomb structure of these columnar phases.

**Figure 6:**
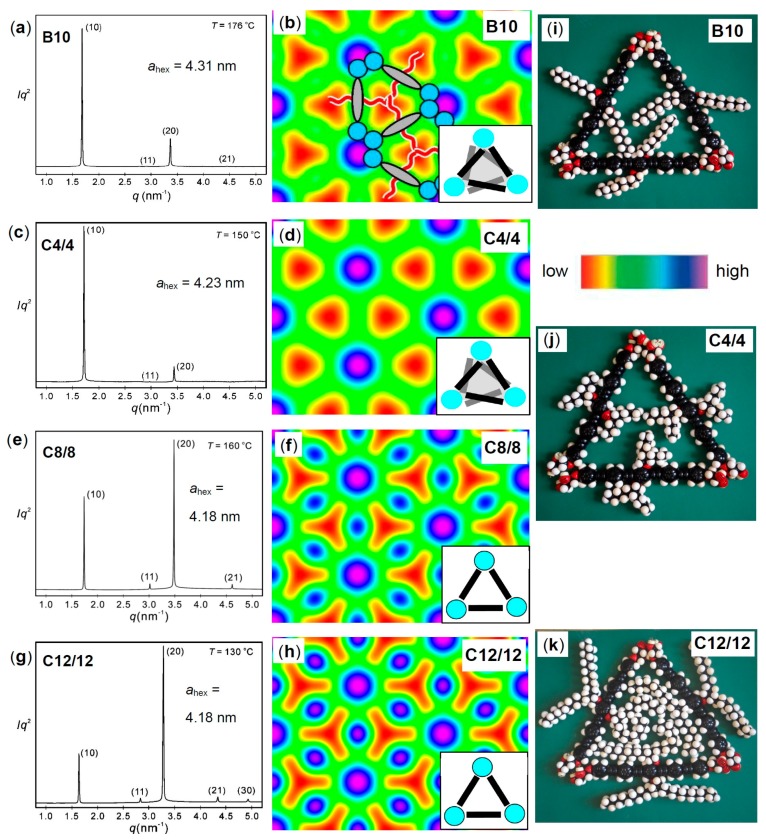
(**a**,**c**,**e**,**g**) SAXS patterns and (**b**,**d**,**f**,**h**) the corresponding reconstructed ED maps of the hexagonal columnar phases (used phase combination is 0π0π) of the noted compounds at the given temperatures; (**i**,**j**,**k**) show the organization of three molecules **B10**, **C4/4**, and **C12/12**, respectively, in the cross-section of a triangular cell with only one molecule in the lateral cross-section of the honeycomb walls (*n*_wall_ = 1.0); in (**k**), the space in the triangular cells is completely filled, whereas there is significant excess free space in the cells in (**i**,**j**). The insets show the models of the phase structures (see also Figure 7); for numerical XRD data, see Table S1, for representative WAXS patterns, see Figure S6a–c.

The SAXS patterns, showing at least three sharp scatterings corresponding to a ratio of the *d* values of 1:1/3^1/2^:1/2 confirm the hexagonal lattice (*p*6*mm*, see Figure 6c,e,g and Table S1). In the wide-angle region, there is only diffuse scattering, with a maximum at *d* = 0.45–0.46 nm corresponding to the lateral mean distance between the molecules (Figure S6a–c). This confirms that the individual molecules do not have fixed positions, i.e., that the compounds are in the LC state. The lattice parameter of the Col_hex_ phases is almost constant from *m* = 4 to *m* = 14 (*a*_hex_ = 4.1–4.4 nm), and thus, being in the range of the length of the rod-like part of the molecules, including the glycerol groups (*L*_mol_ = 4.4 nm, see Figure 3b), as typical for triangular honeycomb LCs. The triangular honeycomb structure of all Col_hex_ phases is further confirmed by the reconstructed electron density (ED) maps shown in Figure 6d,f,h for compounds **C4/4**, **C8/8**, and **C12/12** as examples. The large high ED dots on the hexagonal lattice (blue/purple) indicate the columns of the glycerols, the alkyl chains have lowest ED (red) and fill the triangular cells, which are separated by the honeycomb walls with medium ED (green), where the OPE cores are located. In some ED maps, the central tetrasubstituted benzenes show up as additional high ED dots (blue /purple) in the middle of the walls.

**Figure 7:**
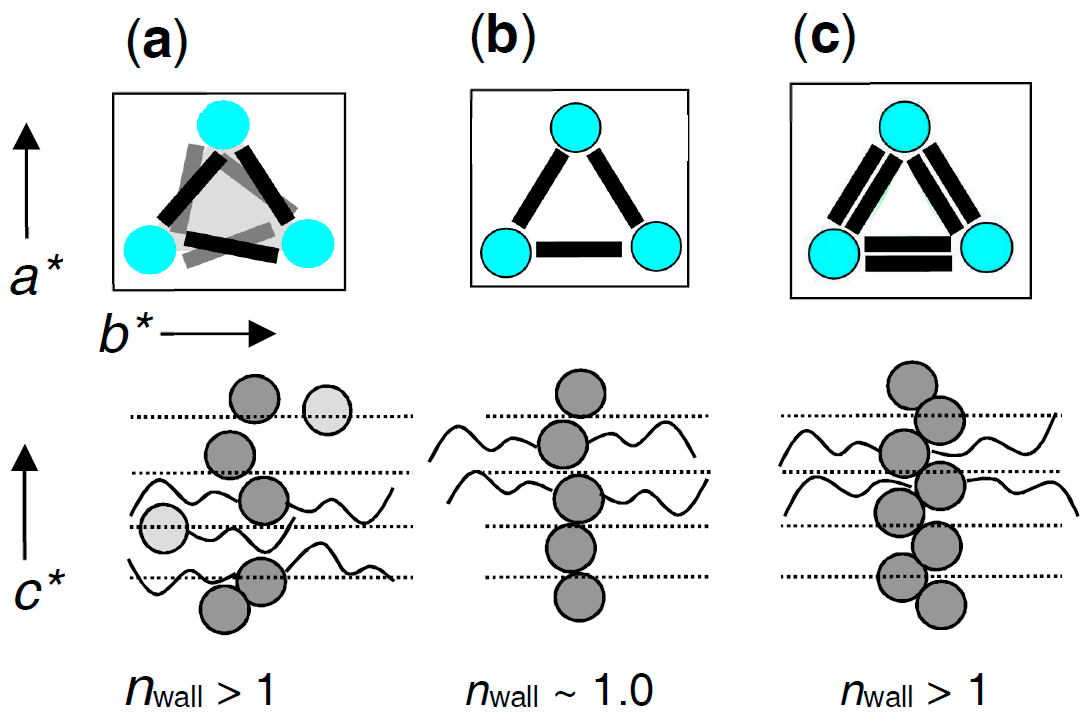
Cross-sections through one prismatic cell with view along direction c* (upper row) and through a single honeycomb wall perpendicular to c* (lower row, the gray cycles represent cuts through the OPE cores perpendicular to their long axes; the horizontal dotted lines indicate the height of each segment with *h* = 0.45 nm; (**a**) weakly segregated structure with additional disordered molecules filling the space in the triangular cells (light gray); (**b**) well-segregated structure with a single molecule in the lateral cross-section of the walls; and (**c**) laterally staggered organization, allowing dense packing of the aromatics and retaining the capability of all lateral chains to escape into the prismatic cells.

It is interesting that even the compound with only *m* = 4 can form a (monotropic) hexagonal columnar LC phase, though these chains cannot fill the triangular cells, and can even barely reach their centers (see Figure 6j). This means that the free space in the prismatic cells with triangular cross-section has to be filled by additional molecules, and therefore, more than just three molecules are involved in each unit cell (see Table 1, for calculations, see Table S4). For **C4/4**, *n*_cell_ amounts to 4.7 molecules. In principle, the additional molecules (compared with the theoretical number of *n*_cell_ = 3) can either be incorporated by lateral staggering of the OPE cores (Figure 7c), or the excess molecules fill the free space in the triangular cells more or less randomly (Figure 7a). In contrast, as shown in Figure 6k, complete space filling is achieved for **C12/12**. For this compound, *n*_cell_ is close to the theoretical value of three molecules (see Table 1), meaning that the walls are formed by a non-staggered organization with just a single molecule in the cross-section (*n*_wall_ ~ 1; Figure 7b). Remarkably, also, the SAXS patterns depend on the size of the lateral chains, being most evident from the different intensities of the (20) reflection with respect to the (10) reflection (Figure 6c,e,g), indicating differences in ED modulation. In the reconstructed ED maps, the distinct high ED dots (blue/purple) in the middle of the triangular walls separating the low ED triangles (red) in the ED map of **C12/12**, are missing in the ED map of **C4/4**, indicating some mixing of the high ED rod-like cores with the low ED alkyl chains. This means that for the short chain compounds, there is an increased deviation from precisely parallel arrangement of the OPE cores in the honeycomb walls, which leads a reduced sharpness of the interfaces, as shown in Figure 7a. The reduced order of the OPE cores is also evident from the comparison of the textures in Figure 5. The smaller birefringence in the Col_hex_ phase of **C4/4** (Figure 5a, gray spherulites) compared to the longer homologue **C12/12** (Figure 5b, red spherulites) is indicative for the reduced order parameter of the OPE rods in the honeycombs of **C4/4**. The same arguments can also be applied to the series of compounds **B*n*** with linear chains (see Figure 2a and Figure 6a,b,i). Overall, with decreasing lateral chain volume, a transition from a well-developed polyphilic (triphilic) self-assembly towards (binary) amphiphilic self-assembly takes place. This means that despite that the triangular honeycomb structure is fundamentally retained, significant local disorder evolves.

As mentioned above, complete space filling is achieved for **C12/12** and **C13/13**, and further chain elongation to **C14/14** leads to triangular honeycombs involving slightly less than exactly three molecules per unit cell (*n*_cell_ = 2.7–2.8; see Table 1). This leads to triangular honeycombs with reduced stability (the Col_hex_-Iso transition temperature goes down, see Figure 4) and an alternative, not yet solved LC phase (M2) replaces the triangular honeycomb at lower temperature. For compound **C16/16**, the mesophase stability is the lowest, and the Col_hex_ phase is completely replaced by the monotropic M2 phase. For even longer homologues with *m* = 18–22, square columnar phases with *p*4*mm* lattice (Col_squ_, see Table 1 and Figure 4), representing square honeycombs, were found [47]. A special feature of these square phases is a temperature dependent transition from a simple square phase having the molecules organized, on average, perpendicular to the prismatic cells long axes (Col_squ_), to a square phase with smaller lattice parameter *a*_squ_ on cooling, having a tilted organization of the OPE cores in the honeycomb walls (Col_squ_*^T^*), as described previously [47]. It appears that for the square honeycombs, the tilting of the OPE cores in the walls represents an alternative way to adjust the prismatic cell volume to the actually available lateral chain volume. In this case, segregation of OPE cores and lateral chain is fully retained, meaning that only for compounds with short chains, space adjustment can be achieved by incorporation of additional molecules, being associated with reduced segregation (Figure 7a). In contrast, for longer chains with enhanced incompatibility, the mixing of the chains with the OPE cores becomes unfavourable, and in this case, tilting allows the adjustment of cell volume and lateral chain volume with full retention of polyphilicity.

**Figure 8:**
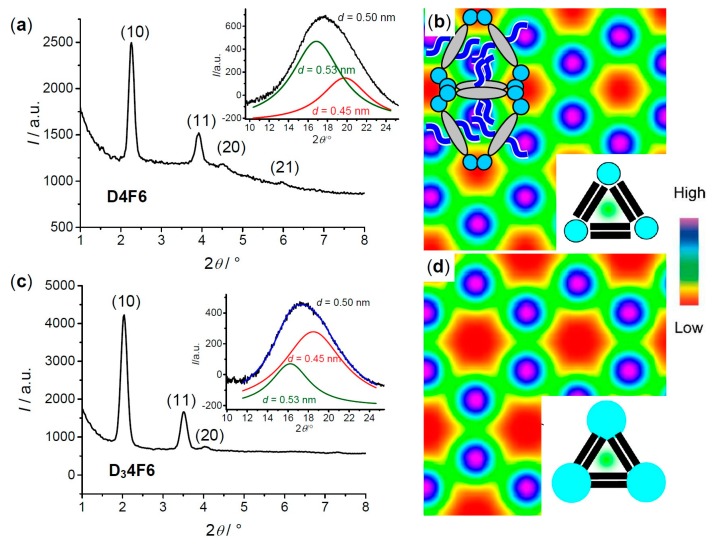
XRD patterns and reconstructed ED maps of the chain fluorinated compounds **D*_x_*4F6**. (**a**,**c**) SAXS patterns (the insets show the WAXS with fitting to two maxima, green and red lines, respectively) and (**b**,**d**) reconstructed ED maps (chosen phase combination is π0π) of compounds (**a**,**b**) **D4F6** at *T* = 160 °C and (**c**,**d**) **D_3_4F6** at *T* = 80 °C, with schematic models of molecular organization shown in the insets of (**b**,**d**); double molecules shown for the walls of the triangles are actually the projected view along the columns axes, and indicate an increased average number of molecules in the diameter, corresponding to *n*_wall_ = 1.6, due to staggering of the aromatics, as shown in Figure 7c; for further details and XRD patterns of the other compounds **D*_n_*4F6**, see Figures S5 and S7, with numerical XRD data collated in Table S3.

**Replacing linear aliphatic by semiperfluorinated lateral chains (D4F6 and E12/4F*q*)**. Compound **D4F6** can be considered as the fluorinated analogue of **B12** [43,44,45,46], having almost the same chain volume (–C_12_H_25_: c.v. = 0.329 nm^3^; –(CH_2_)_4_C_6_F_13_: c.v. = 0.332 nm^3^ ; c.v. = crystal volume as calculated with the increments given in [103]). As shown in Table 2, chain fluorination obviously retains the triangular honeycomb, and leads to a substantial increase of the stability of this honeycomb by 26 K, and expansion of the stable (enantiotropic) phase range from 46 to 71 K. The lattice parameter (*a*_hex_ = 4.51 nm) is only slightly larger than found for the related hydrocarbon **B12** (*a*_hex_ = 4.30 nm), in line with a triangular honeycomb structure, which is for **D4F6**, confirmed by the reconstructed ED map (see Figure 8b). Due to the fluorinated chains, the highest ED (blue/purple) is now found in the prismatic cells, and the ED of the columns involving the glycerol groups is the lowest (red); the OPE cores have medium ED (green).

Enhanced mesophase stability is attributed to the segregation of the fluorinated segments from the non-fluorinated parts (R_F_), known to stabilize LC self-assembly [104,105,106]. This stronger segregation of R_F_ compared to R_H_ inhibits the mixing between lateral chains and the OPE rods, thus leaving only the possibility of lateral staggering of the OPE cores in the cylinder walls for reduction of the space in the triangular honeycombs (Figure 7c). With growing effective wall diameter also, the number of glycerol units organized in the diameter of the polar columns grows, and the increased polar column diameter expands the hexagonal lattice. The observed expansion excludes the possibility of tilting (*longitudinal* staggering) of the OPE cores in the honeycomb walls, which would lead to a reduction of the lattice parameter. The lateral staggering of the aromatics (see Figure 7c) gives rise to a value of about 1.6 molecules arranged on average in the lateral cross-section of each 0.45 nm high segment of the honeycomb walls (*n*_wall_ = 1.6) in the Col_hex_ phase of **D4F6**. Obviously, for the compounds with fluorinated lateral chains, the lateral staggering, leading to an increase of *n*_wall_, is preferred over the longitudinal staggering, leading to tilt. This might be due to the easier accommodation of the linear chains between the staggered cores (see Figure 7c), compared to the more bulky branched chains, which would provide a stronger distortion of the core packing. In addition, the preorganization of the branched chains is parallel to the prismatic cell long axes if their length exceeds the OPE core length (2*m* > 24, see Figure 3b); this supports the tilt by adjusting the OPE alignment to the chain alignment [47]. In contrast, in compounds **D** and **E** the non-branched linear chains cannot exceed the OPE length and these chains are fixed almost perpendicular to the rod-like cores; this removes this driving force of tilt stabilization. Hence, lateral staggering and tilting represent two alternative ways to adjust the cell volume to the lateral chain volume while retaining a polyphilic mode of self-assembly.

**Figure 9:**
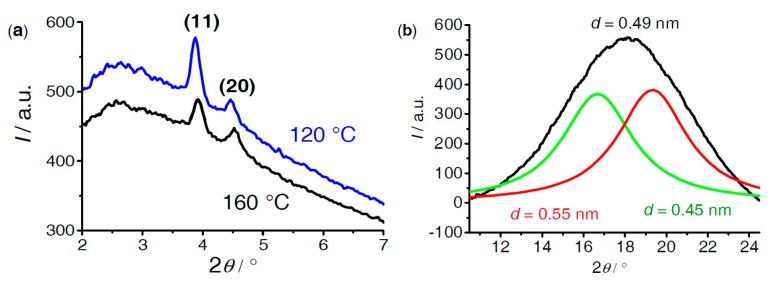
XRD patterns of compound **E12/4F6**; (**a**) SAXS patterns recorded at 120 and 160 °C, respectively; and (**b**) WAXS pattern at 120 °C, with fittings to two maxima representing the mean distances between the R_F_ chains (0.55 nm) and the aliphatic molecular segments (0.45 nm).

Compound **E12/4F6** (Table 2), involving only one fluorinated lateral chain, also forms a Col_hex_ phase, having a phase stability being intermediate between the fluorinated (**D4F6**) and the related non-fluorinated compounds (**B12**). The determined lattice parameter of *a*_hex_ = 4.50 nm is almost identical with the value of compound **D4F6** with two fluorinated chains, and can be explained in the same way. The slight reduction of the hexagonal lattice parameter to *a*_hex_ = 4.45 nm, by alkyl chain elongation from 12 to 18 (compound **E18/4F6**), is in line with the suggested model. Accordingly, the improved space filling by the longer -C_18_H_37_ chains reduces the number of molecules required in the lateral cross-section of the honeycomb walls. Therefore, *n*_wall_ goes down to 1.4, and the smaller diameter of the polar columns gives rise to a reduced *a*_hex_. The SAXS pattern of **E12/4F6**, combining fluorinated and non-fluorinated lateral chains, shown in Figure 9a, is quite different from the pattern of the Col_hex_ phase of **D4F6**, having exclusively fluorinated chains (Figure 8a). It is dominated by a diffuse small angle scattering, a sharp (10) reflection is missing, and the (11) and (20) reflections represent the only observable sharp scatterings. This kind of pattern is indicative for a segregation of fluorinated and non-fluorinated lateral chains into different prismatic cells. However, there is no long-range correlation between the different cells. This averages the local *p*3*m*1 lattice of the segregated structure with alternating cells containing either the fluorinated or non-fluorinated chains into a long range averaged *p*6*mm* lattice [23].

### 3.2. Variation of the Terminal Polar Groups

**Bolapolyphiles with two branched alkyl chains and EO groups (C*_x_m*/*m*)**. Previous work on bolapolyphiles was mainly focused on compounds with simple glycerol groups; here, we report a systematic study of the effect of enlarging the polar groups. First, the focus is on the series with *m* = 12 (Table 3).

**Figure 10:**
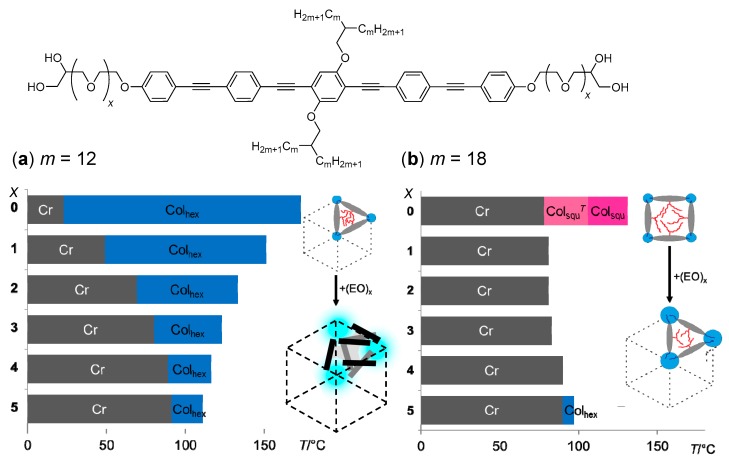
Bar diagrams illustrating the dependence of the LC phases and their transition temperatures (cooling with 10 K·min^−1^) on the number of EO units (*x*) and models of the molecular organization; (**a**) for **C*_x_*12/12**; and (**b**) for **C*_x_*18/18**.

**Figure 11:**
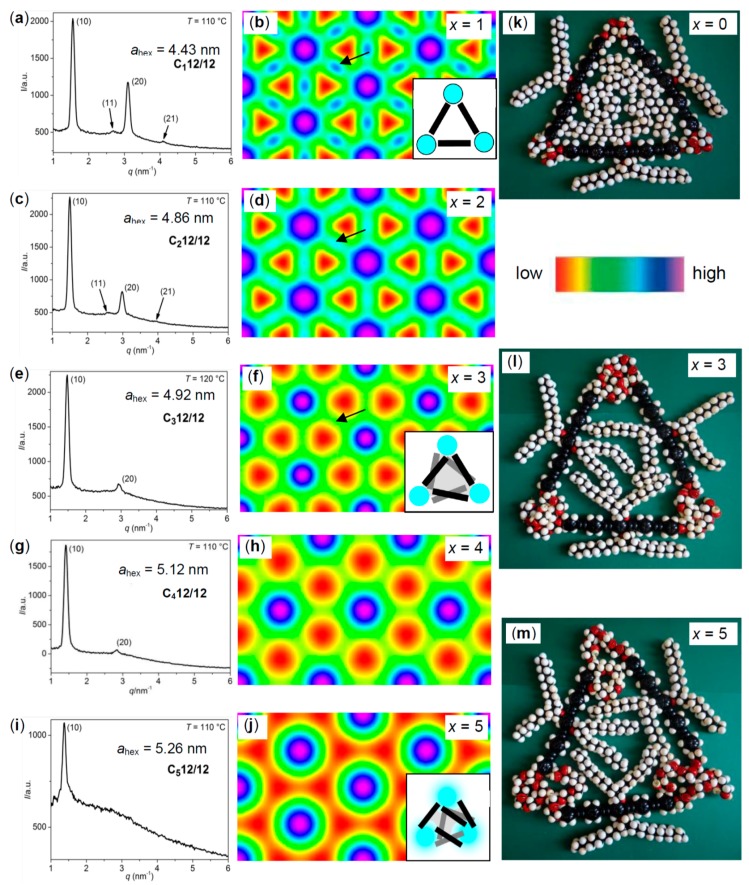
(**a**,**c**,**e**,**g**,**i**) SAXS patterns and (**b**,**d**,**f**,**h**,**j**) the derived ED maps of the Col_hex_ phases (for used phase combinations, see Table S2) of compounds **C*_x_*12/12** depending on *x*; (**k**–**m**) show models of three molecules **C12/12**, **C_3_12/12**, and **C_5_12/12**, respectively, forming a triangular cell with the correct dimensions; for (**k**) and especially (**l**), reduced order of the OPE cores and partial mixing of EO and alkyl chains leads to improved space filling. The insets show the models of the phase structures; for numerical XRD data, see Table S2, the WAXS patterns are displayed in Figure S6.

All compounds **C*_x_*12/12** form hexagonal columnar phases, with the Col_hex_-Iso transition temperatures (*T*_hex-iso_), as well as the associated transition enthalpies, strongly decreasing with growing *x* (*T*_hex-iso_ decreases from 175 to 126 °C, and the corresponding Δ*H* from 12 to 2 kJ·mol^−1^); because the melting temperatures increase, the Col_hex_ temperature range narrows considerably with growing *x* (Figure 10a). The hexagonal lattice parameter grows with increasing *x*, from *a*_hex_ = 4.43 nm for *x* = 0 to 5.26 nm for *x* = 5 (Table 3). This is in line with the significantly increasing molecular length achieved by introduction of the EO units at both ends. Assuming a fully stretched *all-trans* conformation of the EO units would lead to an increase of *L*_mol_ = 4.4 nm for *x* = 0 to *L*_mol_ = 7.7 nm for *x* = 5, as determined with CPK models (see Table 3). However, as the oligo(ethylene oxides) are known to prefer non-linear folded or helical conformations [107], the actual effect of the molecular length on the lattice parameter is smaller and determined by the growing diameter of the polar columns (see Figure 11l,m).

**Figure 12:**
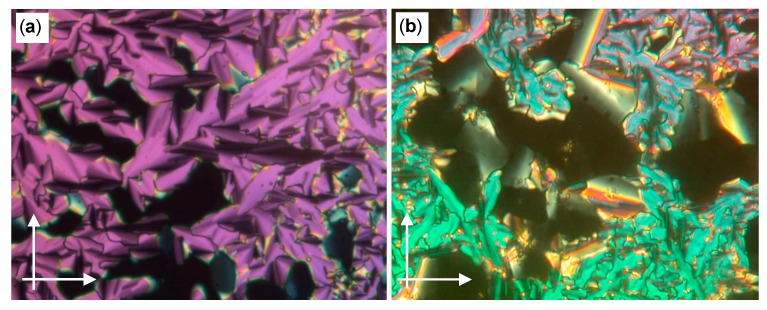
Representative textures of the Col_hex_ phases of compounds with extended polar groups; (**a**) compound **C_5_12/12** at *T* = 111 °C (the texture of **C_4_12/12** is shown in Figure S3), and (**b**) **D_3_4F6** at *T* = 120 °C; the dark areas in both textures represent homeotropic areas where the columns are aligned perpendicular to the glass substrates, indicating optical uniaxiality of these columnar phases; the orientation of the polarizers is indicated with arrows.

The development of the SAXS pattern with growing *x* (see Figure 11a,c,e,g,i) shows that the intensity of the (11), (20), and (21) reflections decrease with respect to the intensity of the (10) reflection. For **C_3_12/12**, only the (20) reflection could be observed besides the layer reflection, and for **C_5_12/12** also, the (20) reflection has obviously disappeared. Though this diffraction pattern with only one reflection could alternatively be interpreted as resulting from a lamellar structure, based on the optical textures (see Figure 12a), a lamellar phase can be excluded. For all compounds of the series **C_x_12/12**, the same kind of spherulitic texture with homeotropic areas is observed under the polarizing microscope, which is typical for the optical uniaxial Col_hex_ phases (see also Figure S3). In addition, for **C_5_12/12** (Figure 11i), the small angle scattering becomes a bit diffuse, suggesting that also, the coherence length of the periodicity of the hexagonal 2D lattice is reduced a bit, in line with increasing disorder. The development of the SAXS pattern leads to the conclusion that with growing EO content, the triangular honeycomb structure becomes more and more distorted, leading to diffuse interfaces between the nano-segregated lateral alkyl chains and the OPE cores. The number of molecules in a hexagonal unit cell with an assumed height of *h* = 0.45 nm is *n*_cell_ = 3.2–3.5, and does not change with EO chain elongation. This means that the expansion of *a*_hex_ should be mainly caused by the growing diameter of the polar columns. A partial mixing of the EOs with the lateral alkyl chains can, in this case, fill the excess space in the expanding honeycombs (see Figure 11k–m). This allows the OPE cores to become less ordered (see insets in Figure 11b,f,j), as evidenced by the ED maps reconstructed from the diffraction patterns (Figure 11b,d,f,h,j). With growing *x*, the high ED dots in the middle of the honeycomb walls disappear (arrows in Figure 11b,d,f) meaning that the position of the OPEs becomes less well defined. For *x* = 5, only the hexagonal lattice of the polar columns is retained, and OPE cores and alkyl chains appear to be almost completely mixed (Figure 11j). It appears that this series of compounds provides an example for the transition from a well-defined triply segregated triangular honeycomb structure to a simpler hexagonal columnar phase, composed of polar columns in a lipophilic matrix formed by mixed OPE cores and alkyl chains. Thus, the behaviour changes from the typical self-assembly of (bola)polyphiles, and becomes more similar to the self-assembly of binary (bola)amphiphiles (see insets in Figure 11b,f,j).

For the series **C*_x_*18/18** with significantly larger lateral chains, the situation is different (Table 3 and Figure 10b). Compound **C18/18** forms a square honeycomb, and polar group expansion completely removes any LC phase. Crystalline materials were obtained for all compounds with *x* = 1–4; only for **C_5_18/18**, a small range of a metastable (monotropic) hexagonal columnar phase was observed (see SAXS pattern in Figure S4b and texture in Figure S3b). This is in line with the expansion of the core unit by elongation of the EO units, which provides more space inside the prismatic cells. The alkyl chains cannot fill these expanded square cells, whereas the triangular cells are still too small to accommodate these chains. As a consequence, formation of a honeycomb becomes disfavoured for *x* = 1–4. Only for sufficiently long terminal groups (*x* = 5) can the triangular cells accommodate the chains, and a hexagonal columnar phase with triangular honeycomb structure is formed. That the hexagonal lattice parameter of **C_5_18/18** (*a*_hex_ = 5.60 nm) is a bit larger than that measured for **C_5_12/12** (*a*_hex_ = 5.26 nm) is in line with the flexibility of the large polar groups, allowing an easier accommodation of the longer alkyl chains by expansion of the triangular cells. In contrast to **C_5_12/12**, in the SAXS pattern of the Col_hex_ phase of **C_5_18/18**, the (11), (20), and (21) reflections can be observed besides the (10) reflection (Figure S5), and the ED map (see Figure S8) is similar to **C_2_12/12** (Figure 11d), showing well segregated areas for rods and alkyl chains. This means that the larger alkyl chains, providing an improved space filling of the prismatic cells and an increased incompatibility with the OPE cores, stabilize a triangular honeycomb with relatively sharp interfaces. In this case, the polyphilic mode of self-assembly is retained for compounds with very long EO chains.

**Bolapolyphiles combining semiperfluorinated lateral chains and EO groups (D*_x_*4F6)**. The effect of head group expansion on the self-assembly of the X-shaped bolapolyphiles with two semiperfluorinated lateral chains was studied for the series **D*_x_*4F6**. All compounds **D*_x_*4F6** form enantiotropic Col_hex_ phases in broad temperature ranges (Table 4), as confirmed by XRD and PM (see Table S3 and Figure 12b). Similar to the series **C*_x_*12/12** (Table 3), the LC phase stability decreases with elongation of the EO chains, and the transition enthalpies decrease, starting with *x* = 2 (*T*_hex-iso_ from 207 to 128 °C, and Δ*H* of this transition decreases from 5 to 6 to only 1.2 kJ·mol^−1^). The lattice parameters in the Col_hex_ phases of all compounds **D*_x_*4F6** are ~0.1 nm larger than for compounds **C*_x_*12/12** (compare Table 3 and Table 4), though, the chain volume of compounds **D*_x_*4F6** (*V*_chain_ = 0.33 nm^3^) is significantly smaller compared to the related compounds **C*_x_*12/12** (*V*_chain_ = 1.30 nm^3^). This is in line with the unusually large number of molecules per unit cell (*n*_cell_ = 4.7–4.9) of all fluorinated compounds, leading to values of *n*_wall_ ~ 1.6 in all cases. It should be noted that despite the larger intermolecular distances provided by the R_F_ segments, the same height of *h* = 0.45 nm is used for all calculations, thus excluding that the calculated *n*_cell_ values could be due to an enlarged unit cell size. As already found for the series **C*_x_*12/12**, there is practically no effect of the number of EO units (*x*) on *n*_cell_, and the lattice parameter *a*_hex_ grows from 4.51 nm for *x* = 0 to 5.35 nm for *x* = 5 (Table 4). In the series **D*_x_*4F6**, there is almost no change of the intensity distribution of the scatterings in the SAXS patterns depending on *x* (see Figure 8a,c and Figure S5), if measured at the same reduced temperature (distance to the *T*_hex-iso_ transition). Therefore, the ED maps of all compounds **D*_x_*4F6** are very similar; only the area of the low ED spots (red) on the hexagonal lattice, corresponding to the positions of the polar groups, grows with increasing number of EO units, as expected and shown in Figure 8b,d for *x* = 0 and *x* = 3. This also confirms that the chosen phase combination (π0π) used for ED reconstruction should be correct. In the ED maps of all compounds **D*_x_*4F6**, there is a clear segregation of the R_F_ segments into relatively small distinct high ED domains in the centers of the triangular cells (see Figure 8b,d). This means that segregation of the R_F_ segments from the other molecular parts is retained for all homologues, independent of the number of EO units. Accordingly, a well-defined triangular honeycomb structure with complete segregation of R_F_-segments into separate columns and a laterally staggered organization of the aromatics in the walls (*n*_wall_ ~ 1.6, see Figure 7c) are, in this case, maintained, independent of the size of the polar end groups. The mismatch between the space available in the triangular prismatic cells, and the constant volume provided by the lateral chains, grows with increasing *x.* It is compensated by penetration of some of the EO chains into the space available in the shells of the aliphatic spacers (R_H_) surrounding the R_F_ cores in the triangular prismatic cells. In this way, the low ED areas of the polar groups are enlarged (Figure 8b,d), R_H_/EO segregation as well as chain/OPE segregation are retained, and any disorder of the OPE rods is avoided, i.e., polyphilicity is retained.

The effect of increasing chain fluorination on the LC self-assembly of the glycerol substituted compounds **B12**, **E12/4F6**, and **D4F6**, with distinct ratios of –C_12_H_25_ and –(CH_2_)_4_C_6_F_13_ chains, is shown in Table 2. In Table 5, the related compounds **B_3_12**, **E_3_12/4F6**, and **D_3_4F6**, with additional EO_3_ units between rod and glycerol groups, are compared. In contrast to **B12**, which forms a triangular honeycomb up to 175 °C, compound **B_3_12** has a lamellar phase with much lower LC phase stability.

**Figure 13:**
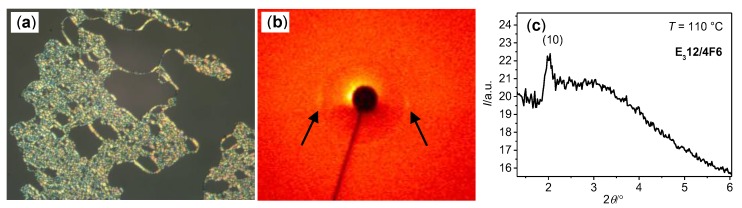
Investigation of the SmA+ phase of compound **E_3_12/4F6**. (**a**) Oily streaks texture with dark homeotropic areas as observed between crossed polarizers at *T* = 130 °C; (**b**) SAXS pattern of an aligned sample at *T* = 110 °C (arrows indicate the weak layer reflections); and (**c**) 2*θ* scan over this pattern.

Compound **E_3_12/4F6**, with one semiperfluorinated chain, forms exclusively a lamellar phase, too. The lamellar phase of **B_3_12** is unstable and immediately crystallizes, whereas that of **E_3_12/4F6** is enantiotropic in the temperature range between 99 and 124 °C, mainly due to the lower melting point. The lamellar organization is indicated by the typical oily streaks texture with large optical uniaxial homeotropic areas (Figure 13a). The optical uniaxiality of the smectic phase (Figure 13a) confirms the absence of a uniform tilt (SmA phase). In this lamellar phase, the layer distance determined by XRD (*d* = 4.38 nm) is quite a bit shorter than the molecular length with the most stretched conformation of the EO chains (*L*_mol_ = 6.5 nm). This is in line with a smectic phase with monolayer structure built up by alternating non-polar layers involving the aromatic cores, the alkyl chains, and the R_F_ segments and polar layers involving the disordered EO chains and the glycerols. In the SAXS pattern, there is an additional diffuse scattering around 2*θ* ~ 2–3° (see Figure 13c), corresponding to a mean distance of ~4.4 nm, which could indicate a partial separation of the R_F_ and R_H_ chains from the rod-like OPE cores into distinct domains with short range correlation. This kind of SmA+ phase (see Figure 1b) with diffuse small angle scatterings and very weak layer reflection has previously been observed for lamellar phases of polyphiles close to the crossover to the organization in honeycombs [11,12].

Only the compound with two fluorinated lateral chains, **D_3_4F6**, shows a broad region of a Col_hex_ phase with triangular honeycomb structure, as indicated by the typical texture (Figure 12b) and SAXS pattern (Figure S5c). It appears that the two fluorinated chains provide a slightly better space filling (0.329 nm^3^ for –C_12_H_25_ and 0.332 nm^3^ for –(CH_2_)_4_C_6_F_13_ [103]), thus leading to the transition from SmA+ to Col_hex_. In addition, the enhanced degree of fluorination can favour the segregation of the lateral chains into larger domains, capable of adopting long range order, thus contributing to the SmA+ to Col_hex_ transition by destabilizing the lamellar and stabilizing the honeycomb organization. This shows that for the OPE derived bolapolyphiles, the triangular honeycomb is the first polygonal honeycomb occurring besides the lamellar organization. Depending on the molecular structure, this transition can take place via a bicontinuous cubic phase (Figure 2) [46] or via a distorted SmA+ phase, with local segregation of rods and lateral chains [11].

## 4. Conclusions

Series of new bolapolyphilic X-shaped molecules with lateral alkyl chains being either linear, branched or semiperfluorinated, and with glycerol groups, being either directly attached or separated by 1–5 additional ethylene oxide (EO) units at both ends of a rod-like π-conjugated oligo(phenylene ethynylene) (OPE) core have been synthesized and investigated (Scheme 2).

**Figure 14:**
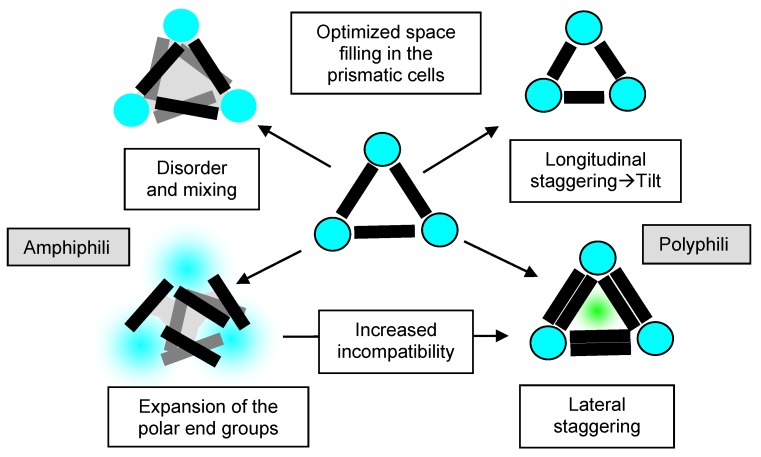
Transitions between polyphilic (triphilic) and amphiphilic self-assembly in the polygonal honeycomb LC phases of X-shaped bolapolyphiles, shown for the example of triangular honeycombs (see also Figure 7).

The self-assembly in the bulk state is dominated by the formation of hexagonal columnar LC phases, representing triangular honeycombs, where the polar glycerol groups (together with the EO units) form columns on a hexagonal lattice, which are interconnected by ribbons of parallel organized OPE rods forming the walls of the honeycombs. The resulting prismatic cells, with triangular cross-section, are filled by the lipophilic lateral chains. A continuous transition from a well-defined triangular honeycomb structure to a hexagonal columnar phase, with almost fully mixed alkyl chains and aromatics (see Figure 7a), and thus, dominated by the arrangement of polar columns on a hexagonal lattice, was observed by reducing the length and volume of the lateral alkyl chains (Figure 14). In this case, partial mixing of aromatic cores and lateral chains is required to fill the space available in the triangular prismatic cells. A similar transition was observed upon increasing the length of the EO units involved in the polar groups. In this case, the swelling of the polar columns increases the space available in the prismatic cells, thus requiring the mixing of rod and chains to efficiently fill this space. If the lateral alkyl chains are elongated or replaced by semiperfluorinated chains, then the segregation of lateral chains and rod-like cores is retained to a much larger extend. In this case, space filling of the prismatic cells is mainly achieved by formation of thicker honeycomb walls, i.e., lateral staggering of the aromatic cores leads to a lateral shift in the organization of the rod-like cores, which in this way, reduces the space available in the prismatic cells. This means that in this case, segregation is retained. Segregation is also retained by longitudinal staggering, giving rise to a tilt of the rod-like cores in the honeycomb walls, which is an alternative way to reduce the space available in the prismatic cells, as observed for the square honeycombs on cooling [47].

Combining fluorinated and non-fluorinated chains at opposite sides of the OPE core generates tetraphiles, where adjacent prismatic cells of the self-assembled honeycombs can be filled with different chains. However, in the case reported here, the R_F_ chains are too short to enable a long range periodic lattice for the “two-color” superlattice with reduced symmetry [23].

Overall, the competition between the ordering force of nano-segregation on the one side, and the steric and geometric frustration, as well as entropic disordering forces on the other side, leads to transitions between polyphilic and simple amphiphilic self-assembly for the polyphiles reported herein [17], representing low molecular mass high-*χ*–low-*N*-analogues of the related block copolymers [108,109].

## Figures and Tables

**Table 1:**
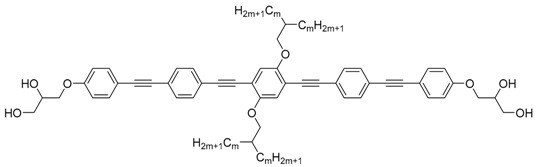
Mesophase, phase transition temperatures (*T*/°C) as observed on heating (top lines) and cooling (bottom lines), the corresponding enthalpy values (Δ*H*/(kJ·mol^−1^), in square brackets), and other data of compounds **C*m/m***, depending on the length of the branches *m* ^a^.

C*m/m*	Phase transitions	*a* (*T*/°C)	*n*_cell_	*n*_wall_
**C2/2**	Cr 215 [57.2] Iso	-	-	-
	Iso 205 [36.2] Cr			
**C4/4**	Cr 172 [62.6] Iso	4.23 (150 °C)	4.68	1.56
	Iso 167 [3.1] Col_hex_ [18.8] 138 Cr			
**C6/6**	Cr 111 [27.0] Col_hex_ 178 [4.5] Iso	4.08 (150 °C)	3.79	1.26
	Iso 176 [4.6] Col_hex_ 88 [0.7] M1 74 [5.4] Cr			
**C8/8**	Cr 77 [21.0] M1 80 [0.7] Col_hex_ 185 [7.7] Iso	4.16 (160 °C)	3.51	1.17
	Iso 184 [7.8] Col_hex_ 80 [0.7] M1 64 [16.3] Iso			
**C10/10**	Cr 110 [19.6] Col_hex_ 187 [11.0] Iso	4.35 (130 °C)	3.46	1.15
	Iso 183 [11.6] Col_hex_ 58 [0.8] M1 38 [12.6] Cr			
**C12/12**	Cr 97 [70.1] Col_hex_ 175 [11.9] Iso	4.43 (130 °C)	3.24	1.08
	Iso 173 [12.4] Col_hex_ 23 [18.9] Cr			
**C13/13**	Cr 110 [68.1] Col_hex_ 165 [9.3] Iso	4.38 (130 °C)	3.03	1.01
	Iso 162 [9.2] Col_hex_ 20 [24.8] Cr			
**C14/14**	Cr 120 [80.0] M2 152 [2.1] Col_hex_ 154 [7.5] Iso	4.24 (154 °C)	2.73	0.91
	Iso 153 [7.6] Col_hex_ 145 [1.6] M2 89 [103.6] Cr			
**C16/16**	Cr 137 [103.1] Iso	-	-	-
	Iso 133 [8.7] M2 114 [92.7] Cr			
**C18/18**	Cr 105 [76.4] Col_squ_^T^ 108 [2.7] Col_squ_ 134 [10.5] Iso	4.05 (120 °C)	2.5	1.23
	Iso 131 [10.4] Col_squ_ 106 [1.9] Col_squ_^T^ 78 [68.6] Cr	3.83 (100 °C)		
**C20/20**	Cr 47 [21.7] Col_squ_^T^ 85 [1.1] Col_squ_ 132 [9.3] Iso	4.09 (100 °C)	2.3	1.17
	Iso 127 [10.5] Col_squ_ 88 [1.0] Col_squ_^T^ 31 [30.0] Cr	3.90 (80 °C)		
**C22/22**	Cr 84 [33.8] Col_squ_ 128 [12.7] Iso	4.12 (110 °C)	2.2	1.11
	Iso 125 [13.1] Col_squ_ 75 [1.2] Col_squ_^T^ 32 [49.4] Cr	4.00 (70 °C)		

^a^ Peak temperatures as determined by DSC, with rate 10 K·min^−1^, representative DSCs are shown in Figure S1a–c; for abbreviations, see Figure 2 and Figure 4. *n*_cell_ = 0.893 *V*_cell_/*V*_mol_, where *V*_mol_ was calculated with crystal volume increments [102], *V*_cell_ = *a*_squ_^2^ × *h* for the Col_squ_ phases and *V*_cell_ = 3^1/2^/2 × *a*_squ_^2^ × *h* (*h* = 0.45 nm) for the Col_hex_ phases, the factor 0.893 is the correction for the LC state, which is assumed to have an intermediate packing density between the liquid (0.55) and the crystalline state (0.7) [103]; *n*_wall_ = number of molecules arranged side-by-side in the honeycomb walls (*n*_cell_/3 for Col_hex_ and *n*_cell_/2 for Col_squ_); for details, see Table S4; for compounds **C18**–**C22** see [47]; abbreviations: Col_squ_ = square columnar phase with *p*4*mm* plane group (square LC honeycomb); Col_squ_*^T^* = Col_squ_ phase with tilted organization of the molecules in the honeycomb walls; M1, M2 = mesophases with unsolved structure; for other abbreviations, see Figure 2.

**Table 2:**
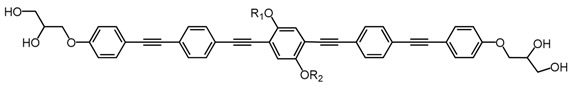
Comparison of the mesophases, phase transition temperatures (*T*/°C) as observed on heating (top lines) and cooling (bottom lines), the corresponding enthalpy values (Δ*H*/(kJ·mol^−1^), in square brackets), and other data of compounds **D** and **E**, with fluorinated lateral chains ^a^.

Comp.	R_1_	R_2_	Phase transitions	*a*_hex_ (*T*/°C)	*n*_cell_	*n*_wall_
**C12** [43,44,45]	–C_12_H_25_	–C_12_H_25_	Cr 178 [65.1] Col_hex_ 181 [3.9] Iso	4.30 (175)	4.5	1.5
			Iso 180 [4.0] Col_hex_ 168 [63.6] Cr			
**D4F6**	–(CH_2_)_4_C_6_F_13_	–(CH_2_)_4_C_6_F_13_	Cr 143 [26.0] Col_hex_ 222 [7.2] Iso	4.51 (160)	4.7	1.6
			Iso 220 [7.0] Col_hex_ 97 [16.4] Cr			
**E12/4F6**	–(CH_2_)_4_C_6_F_13_	–C_12_H_25_	Cr 112 [*22.9*] Col_hex_ 197 [*5.9*] Iso	4.50 (160)	4.7	1.6
			Iso 195 [5.9] Col_hex_ 92 [3.2] Cr			
**E18/4F6**	–(CH_2_)_4_C_6_F_13_	–C_18_H_37_	Cr 140 [51.8] Col_hex_ 185 [5.1] Iso	4.42 (150)	4.1	1.4
			Iso 182 [4.9] Col_hex_ 81 [19.0] Cr			

^a^ Peak temperatures as determined by DSC with rate 10 K·min^−1^ from the second DSC heating scans; for DSCs, see Figure S2; for abbreviations, see Figure 2; for calculation of *n*_cell_ and *n*_wall_, see Table S6.

**Table 3:**
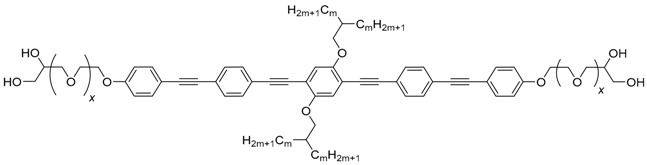
Comparison of the mesophases, and phase transition temperatures (*T*/°C), as observed on heating (top lines) and cooling (bottom lines), the corresponding enthalpy values (Δ*H*/(kJ·mol^−1^), in square brackets), and other data, of compounds **C_x_12/12** and **C_x_18/18**, depending on the number of EO units *x* ^a^.

C*m/m*	*x*	Phase transitions	*a*_hex_ (*T*/°C)	*n*_cell_	*n*_wall_	*L*_mol_ (nm)
**C12/12** [43,44,45]	0	Cr 97 [70.1] Col_hex_ 175 [11.9] Iso	4.43 (130)	3.23	1.08	4.4
		Iso 173 [12.4] Col_hex_ 23 [18.9] Cr				
**C_1_12/12**	1	Cr 92 [63.1] Col_hex_ 152 [5.6] Iso	4.67 (110)	3.42	1.14	5.2
		Iso 150 [5.4] Col_hex_ 44 [47.6] Cr				
**C_2_12/12**	2	Cr 89 [56.3] Col_hex_ 135 [3.2] Iso	4.86 (110)	3.44	1.15	5.9
		Iso 133 [3.4] Col_hex_ 69 [57.6] Cr				
**C_3_12/12**	3	Cr 96 [67.5] Col_hex_ 125 [3.5] Iso	4.92 (120)	3.37	1.12	6.6
		Iso 123 [3.3] Col_hex_ 80 [66.7] Cr				
**C_4_12/12**	4	Cr 99 [69.9] Col_hex_ 118 [2.5] Iso	5.12 (110)	3.56	1.19	7.3
		Iso 116 [2.6] Col_hex_ 89 [70.7] Cr				
**C_5_12/12**	5	Cr 100 [72.1] Col_hex_ 113 [2.2] Iso	5.26 (110)	3.59	1.19	8.0
		Iso 111 [2.4] Col_hex_ 91 [72.9] Cr				
**C18/18** [47]	0	Cr 105 [76.4] Col_squ_^T^ 108 [2.7] Col_squ_ 134 [10.5] Iso	4.05 (120)	-	-	4.4
		Iso 131 [10.4] Col_squ_ 106 [1.9] Col_squ_^T^ 78 [68.6] Cr	3.83 (100)			
**C_1_18/18**	1	Cr 108 [80.0] Iso	-	-	-	5.2
		Iso 81 [99.3] Cr				
**C_2_18/18**	2	Cr 97 [88.3] Iso	-	-	-	5.9
		Iso 81 [100.1] Cr				
**C_3_18/18**	3	Cr 97 [95.1] Iso	-	-	-	6.6
		Iso 83 [102.7] Cr				
**C_4_18/18**	4	Cr 100 [79.9] Cr	-	-	-	7.3
		Iso 90 [93.3] Cr				
**C_5_18/18**	5	Cr 98 [92.3] Iso	5.60 (95)	3.27	1.09	8.0
		Iso 97 [-] Col_hex_ 90 [96.6] Cr				

^a^ Peak temperatures as determined by DSC with rate 10 K·min^−1^ from the second DSC heating and cooling scans (see Figure S1c–f); for abbreviations, see Figure 2 and Table 1; for numerical XRD data, see Table S2, and for calculation of *n*_cell_ and *n*_wall_, see Table S5; the molecular lengths, *L*_mol_, have been measured with fully stretched EO chains in the most extended conformation between the ends of the primary OH groups at both ends.

**Table 4:**
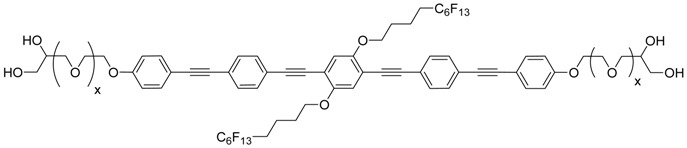
Mesophases and phase transition temperatures (*T*/°C), as observed on heating (top lines) and cooling (bottom lines), the corresponding enthalpy values (Δ*H*/(kJ·mol^−1^), in square brackets), and other parameters of compounds **D*_x_*4F6**, depending on the number of EO units *x* ^a^.

Comp.	*x*	Phase transitions	*a*_hex_/nm (*T*/°C)	*n*_cell_	*n*_wall_
**D4F6**	0	Cr 143 [26.0] Col_hex_ 222 [7.2] Iso	4.51(160)	4.65	1.55
		Iso 220 [7.0] Col_hex_ 97 [16.4] Cr			
**D_1_4F6**	1	Cr 138 [40.5] Col_hex_ 203 [6.2] Iso	4.78 (160)	4.84	1.61
		Iso 200 [6.1] Col_hex_			
**D_2_4F6**	2	Cr 103 [23.0 Colhex 180 [4.3] Iso	4.97 (140)	4.89	1.63
		Iso 178 [4.6] Col_hex_ 80 [18.2] Cr			
**D_3_4F6**	3	Cr 107 [23.9] Col_hex_ 156 [2.3] Iso	5.05 (140)	4.73	1.58
		Iso 153 [2.5] Col_hex_ 88 [20.8] Cr	5.00 (80)		
**D_5_4F6**	5	Cr 99 [24.0] Col_hex_ 128 [1.2] Iso	5.35 (110)	4.72	1.57
		Iso 125 [1.4] Col_hex_ 86 [23.2] Cr			

^a^ Peak temperatures as determined by DSC with rate 10 K·min^−1^, from the second DSC heating and cooling scans (Figure S2); for abbreviations, see Figure 2; numerical XRD data are collated in Table S3; for calculation of *n*_cell_ and *n*_wall_, see Table S6.

**Table 5:**
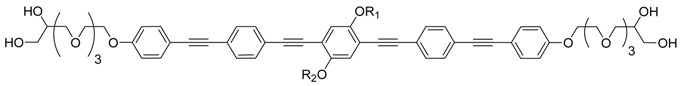
Comparison of the mesophases, phase transition temperatures (*T*/°C), corresponding enthalpy values (Δ*H*/(kJ·mol^−1^), in square brackets), and other parameters, depending on the degree of lateral chain fluorination at almost identical side chain volume ^a^.

Comp.	R_1_	R_2_	Phase transitions	*d*, *a*_hex_/nm (*T*/°C)	*n*_wall_
**B_3_12**	–C_12_H_25_	–C_12_H_25_	Cr 131 [*57.9*] (SmA+ 131) ^b^ Iso	*-*	*-*
**E_3_12/4F6**	–C_12_H_25_	(CH_2_)_4_C_6_F_13_	Cr 99 [*39.7*] SmA+ 124 [*1.0*] Iso	4.38 (110)	*-*
**D_3_4F6**	(CH_2_)_4_C_6_F_13_	(CH_2_)_4_C_6_F_13_	Cr 107 [20.8] Col_hex_ 156 [2.5] Iso	5.05 (140)	1.58

^a^ Peak temperatures as determined by DSC with rate 10 K·min^−1^; (Figure S2); abbreviations: SmA+ lamellar phase with short range correlation of the nano-segregated domains of the lateral chains (see Figure 1b) for other abbreviations, see Figure 2; numerical XRD data are collated in Table S3, for calculation of *n*_wall_, see Table S6; ^b^ Enthalpy involves both transitions, and the sample immediately crystallizes during formation of the SmA+ phase.

## Supplementary Materials

The following are available online at www.mdpi.com/2073-4360/9/10/471/s1. Additional data: Figures S1 and S2: DSC traces; Figure S3: Textures; Figures S4 and S5: SAXS patterns; Figure S6 and S7: WAXS patterns; Figure S8: Reconstructed ED maps; Tables S1–S3 numerical XRD data; Tables S4–S6: Structural data. Synthesis and analytical data of all new compounds with Schemes S1–S5 showing details of the synthesis and Figures S9–S30 showing representative NMR spectra of selected compounds.
