# Supplementary material for: Effects of Lateral and Terminal Chains of X-Shaped Bolapolyphiles with Oligo(phenylene ethynylene) Cores on Self-Assembly Behaviour. Part 1: Transition between Amphiphilic and Polyphilic Self-Assembly in the Bulk
Source: Polymers (Basel). 2017 Sep 26;9(10):471. doi: 10.3390/polym9100471 (PMC6418615; doi:10.3390/polym9100471)
Supplement: Supplementary file 1 [file polymers-09-00471-s001.pdf]

Supporting Information

Effects of lateral and terminal chains of X-shaped bolapolyphiles with oligo(phenylene enthynylene) cores on self-assembly behaviour

Part 1: Transition between amphiphilic and polyphilic self-assembly in the bulk

Silvio Poppe,[1,#] Marco Poppe,[1,#] Helgard Ebert,[1] Marko Prehm,[1] Changlong Chen,[2] F Liu,[2] Sentefan Werner,[3] Kirsten Bacia,[3] Carsten Tschierske[1,*]

[1] Department of Chemistry, Martin Luther University Halle-Wittenberg, Kurt-Mothes-Str. 2, 06120 Halle, Germany
[2]State Key Laboratory for Mechanical Behavior of Materials, Xi'an Jiaotong University, Xi'an 710049, P. R. China
Department of Chemistry, Martin Luther University Halle-Wittenberg, Kurt-Mothes Str. 3, 06120 Halle, Germany

# these authors contributed equally

Content

1. Additional Data…………………………………………………………….. S2
1.1 DSCs…………………………………………………………………………. S2
1.2 Textures……………………………………………………………………… S3
1.3 SAXS………………………………………………………………………… S5
1.4 WAXS………………………………………………………………………… S9
1.5 ED maps……………………………………………………………………… S10
1.6 Structural parameters……………………………………………………….. S11
2. Synthesis and Analytical Data……………………………………………… S12
2.1 General remarks and procedures…………………………………………… S12
2.2 Synthesis of the 1,4-dialkoxy-2,5-diiodobenzenes with branched alkyl chains (**5**)… S15
2.3 Synthesis of the 1,4-disubstituted-2,5-dihalobenzenes with semiperfluorinated alkyl chains (**6**)……………………………………………………………..... S21
2.4 Preparation of the 1,2-*O*-isopropylidene protected hydrophilic units……………… S24
2.5 Synthesis of compounds **C*m/m***……………………………………………… S28
2.6 Synthesis of compounds **C*ₓm/m***…………………………………………….. S34
2.7 Synthesis of the compounds with semiperfluorinated chains (**D_x4F6, E_x12/4F6, E18/4F6**)………………………………………………………………………. S40
2.8 Synthesis of compounds **$E_3$12**……………………………………………... S45
3. Representative NMR Spectra………………………………………………… S46
4. References……………………………………………………………………. S58

1. Additional Data

1.1 DSCs

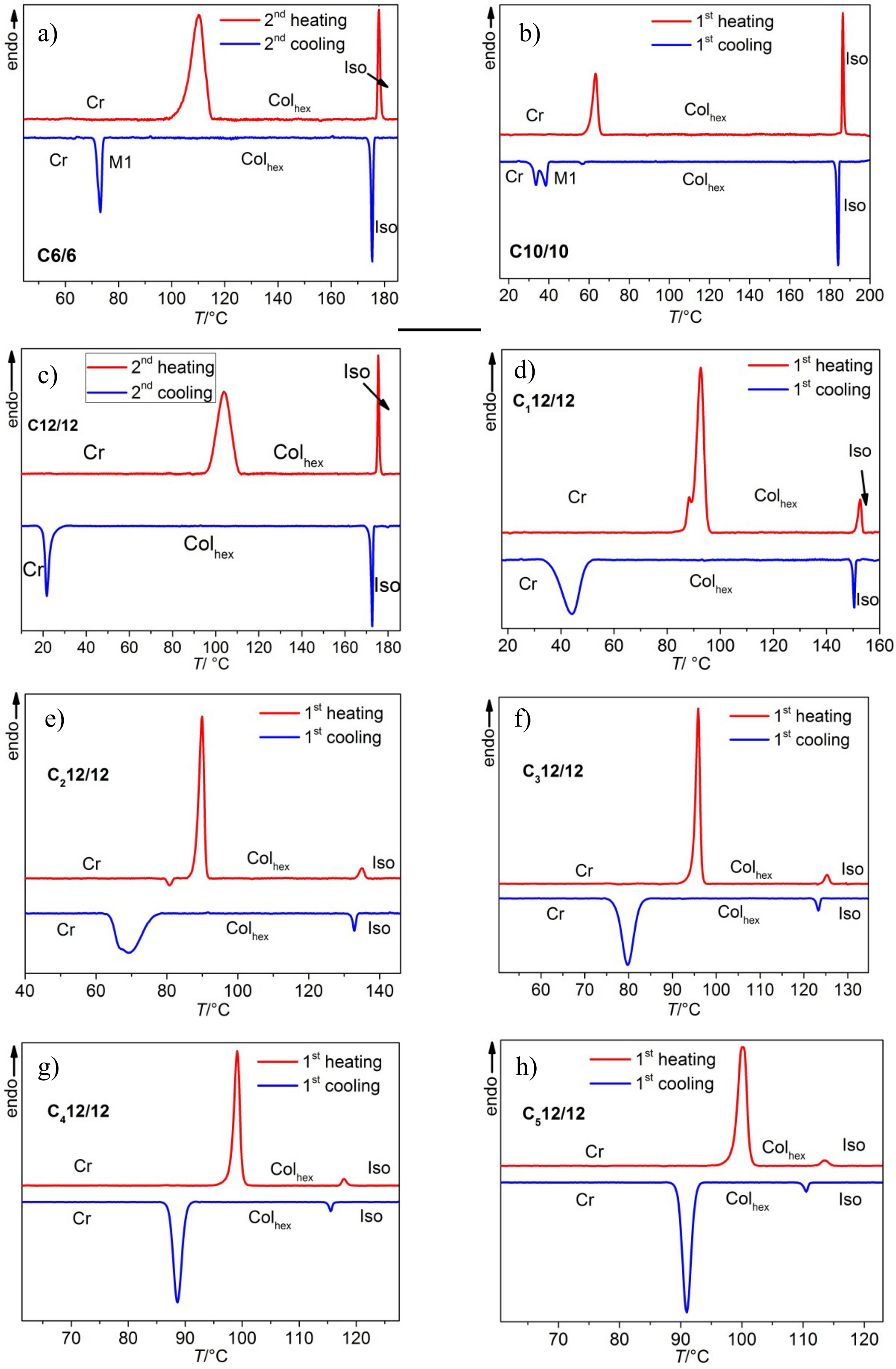

Figure S1. DSC heating and cooling traces of compounds a) **C6/6**, b) **C10/10** and c-h) **C*x*12/12** recorded at 10 K/min.

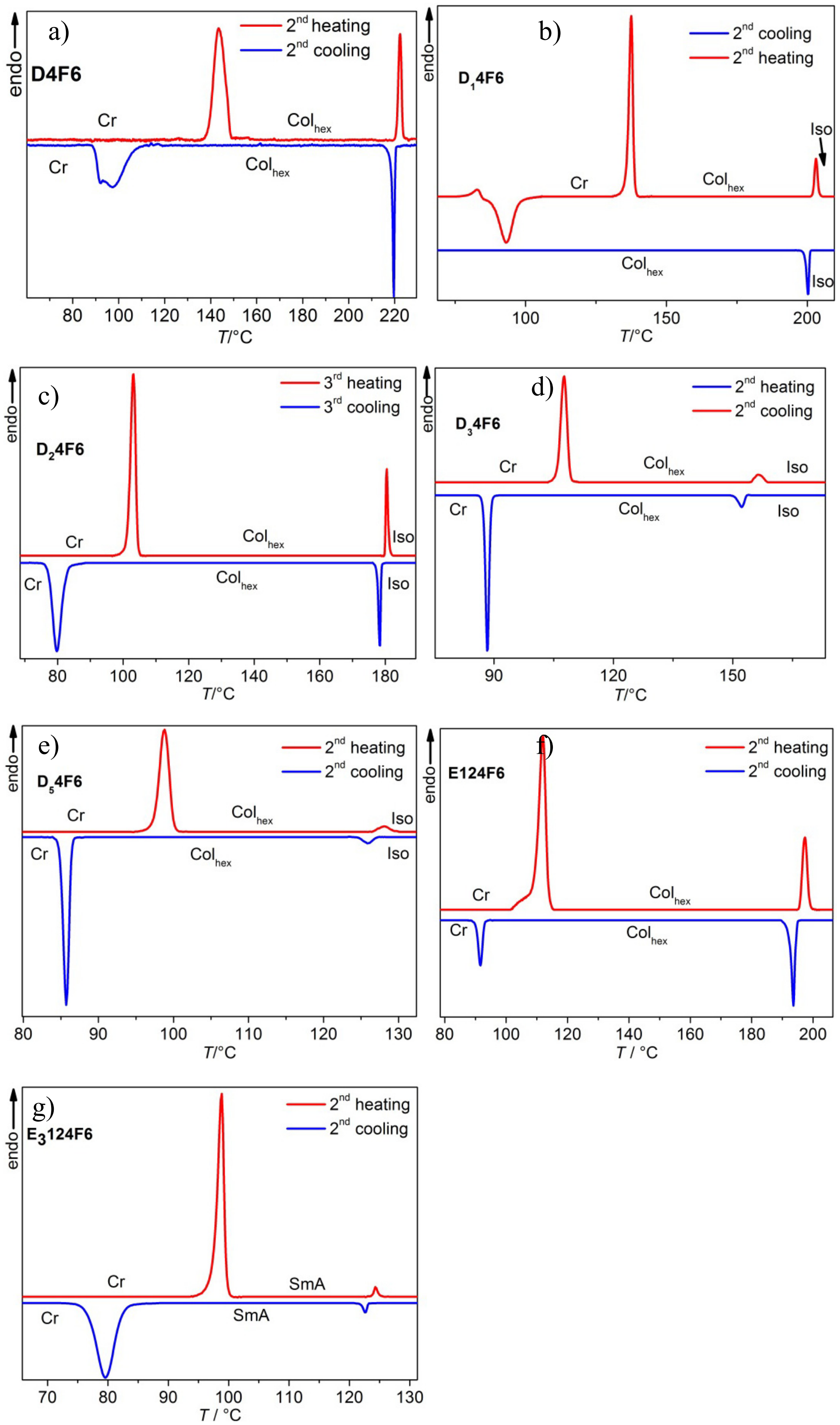

Figure S2. DSC heating and cooling traces of compounds **D$_x$4F6** and **E$_x$12/4F6** recorded at 10 K//min.

1.2 Textures

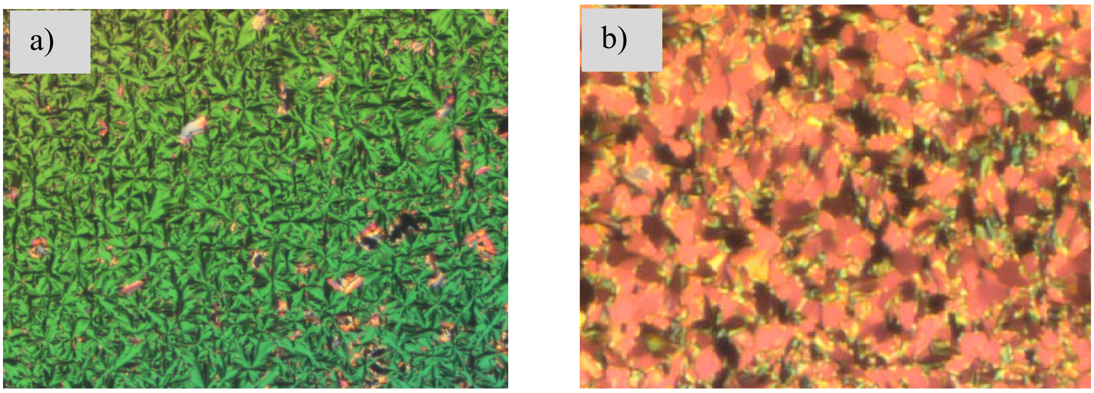

Figure S3. Texture of the Col$_{hex}$ phase of a) **C$_4$12/12** at 110 °C and b) **C$_5$18/18** at 95 °C between crossed polarizers.

1.3 SAXS data

Table S1. Experimental and calculated *d*-spacings of the observed SAXS reflections of the Col_{hex} phases of compounds **C*m/m***.

**Comp.**	(*hk*)	2θ (°)	d_{obs} (nm)	d_{cal} (nm)	Δ	intensity	phase	a_{hex} (T/°C)
**C4/4**	(10)	2.41	3.66	3.66	0.00	100.0	0	4.23 (150 °C)
	(11)	4.18	2.11	2.12	0.01	0.3	π	
	(20)	4.82	1.83	1.83	0.00	10.0	0	
**C6/6**	(10)	2.51	3.53	3.53	0.00	100		4.08 (150 °C)
	(20)	5.01	1.76	1.76	0.00	8.5		
**C8/8**	(10)	2.45	3.61	3.60	0.01	65.3	0	4.16 (160 °C)
	(11)	4.25	2.08	2.08	0.00	3.8	π	
	(20)	4.91	1.80	1.80	0.00	100.0	0	
	(21)	6.48	1.36	1.36	0.00	1.7	π	
**C10/10**	(10)	2.34	3.77	3.77	0.00	27.6		4.35 (130 °C)
	(11)	4.05	2.18	2.18	0.00	2.7		
	(20)	4.69	1.88	1.88	0.00	100.0		
	(21)	6.21	1.42	1.42	0.00	2.1		
	(30)	7.08	1.25	1.26	0.01	1.2		
**C12/12**	(10)	2.30	3.84	3.84	0.00	26.9	0	4.43 (130 °C)
	(11)	2.20	2.21	2.22	0.01	2.6	π	
	(20)	4.60	1.92	1.92	0.00	100.0	0	
	(21)	6.10	1.45	1.45	0.00	1.8	π	
	(30)	6.90	1.28	1.28	0.00	2.0	0	
**C13/13**	(10)	2.32	3.80	3.79	0.01	34.5		4.28 (140 °C)
	(11)	4.04	2.19	2.19	0.00	2.2		
	(20)	4.65	1.90	1.90	0.00	100.0		
	(21)	6.19	1.43	1.43	0.00	1.5		
	(30)	7.00	1.26	1.26	0.00	2.0		
**C14/14**	(10)	2.41	3.67	3.67	0.00	66.5		4.24 (130 °C)
	(11)	4.16	2.12	2.12	0.00	1.4		
	(20)	4.18	1.84	1.84	0.00	100.0		
	(21)	6.35	1.39	1.39	0.00	0.8		

Table S2. Experimental and calculated *d*-spacings of the observed SAXS reflections of the Col_{hex} phases of compounds $\mathbf{C}_x\boldsymbol{m/m}$.

**Comp.**	(*hk*)	*2θ* (°)	d_{obs} (nm)	d_{cal} (nm)	Δ	intensity	phase	a_{hex} (*T*/°C)
$\mathbf{C_112/12}$	(10)	2.183	4.047	4.047	0.00	100	0	4.67 (110 °C)
	(11)	3.759	2.350	2.337	0.01	2.6	π	
	(20)	4.360	2.027	2.024	0.00	49.7	0	
	(21)	5.750	1.537	1.530	0.01	0.8	π	
$\mathbf{C_212/12}$	(10)	2.099	4.209	4.209	0.00	100	0	4.86 (110 °C)
	(11)	3.594	2.458	2.430	0.03	2.3	π	
	(20)	4.195	2.106	2.105	0.00	20.5	0	
	(21)	5.566	1.588	1.590	0.00	0.5	π	
$\mathbf{C_312/12}$	(10)	2.074	4.260	4.260	0.00	100	0	4.92 (120 °C)
	(20)	4.131	2.139	2.130	0.01	7.2	0	
$\mathbf{C_412/12}$	(10)	1.991	4.437	4.437	0.00	100	0	5.12 (110 °C)
	(20)	3.973	2.224	2.218	0.01	4.08	0	
$\mathbf{C_512/12}$	(10)	1.938	4.558	4.558	0.00	100	0	5.26 (110 °C)
$\mathbf{C_518/18}$	(10)	1.82	4.85	4.85	0.00	100.0	0	5.60 (95 °C)
	(11)	3.10	2.80	2.80	0.00	2.7	π	
	(20)	3.64	2.43	2.43	0.00	28.8	0	
	(21)	4.83	1.83	1.83	0.00	1.4	π	

Table S3. Experimental and calculated *d*-spacings of the observed SAXS reflections of the mesophases of compounds **D$_x$4F6** and **E$_x$12/4F6** with fluorinated chains

Comp.	*(hk)*	2θ (°)	d_{obs} (nm)	d_{cal} (nm)	Δ	intensity	phase	a_{hex} (*T*/°C)
**D4F6**	(10)	2.262	3.906	3.906	0.00	1.00	π	4.51 (160 °C
	(11)	3.915	2.257	2.255	0.00	0.28	0	
	(20)	4.522	1.953	1.953	0.00	0.06	π	
**D$_1$4F6**	(10)	2.133	4.142	4.140	0.00	1.00		4.78 (160 °C)
	(11)	3.688	2.396	2.390	0.01	0.15		
	(20)	4.265	2.071	2.070	0.00	0.01		
**D$_2$4F6**	(10)	2.052	4.305	4.304	0.00	1.00		4.97 (140 °C)
	(11)	3.550	2.489	2.485	0.00	0.13		
**D$_3$4F6**	(10)	2.020	4.373	4.373	0.00	1.00		5.05 (140 °C)
	(11)	3.490	2.532	2.525	0.01	0.12		
**D$_3$4F6**	(10)	2.040	4.331	4.330	0.00	1.00	π	5.00 (80 °C)
	(11)	3.510	2.517	2.500	0.02	0.29	0	
	(20)	4.040	2.187	2.165	0.01	0.02	π	
**D$_5$4F6**	(10)	1.906	4.635	4.633	0.00	1.00		5.35 (110 °C)
	(11)	3.307	2.672	2.675	0.00	0.08		
**E12/4F6**	(10)	2.558	3.454	3.895	0.40	0.49		4.50 (160 °C)
	(11)	3.929	2.249	2.249	0.00	1.00		
	(20)	4.505	1.961	1.948	0.01	0.71		
**E$_3$12/4F6**	(10)	2.021	4.372	4.372	0.00	1.00		4.37 (110 °C)
**E18/4F6**	(10)	2.321	3.830	3.830	0.00	0.19		4.42 (150 °C)
	(11)	3,998	2.210	2.210	0.00	0.40		
	(20)	4.642	1.910	1.910	0.00	1.00		

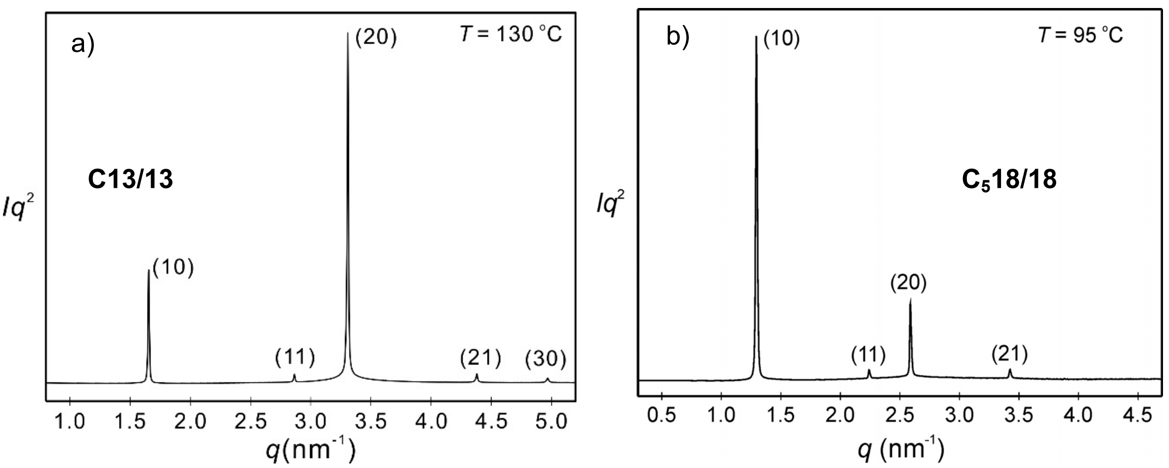

Figure S4. SAXS patterns of compounds a) **C13/13** at 130 °C and b) **$C_5$18/18** at 95 °C.

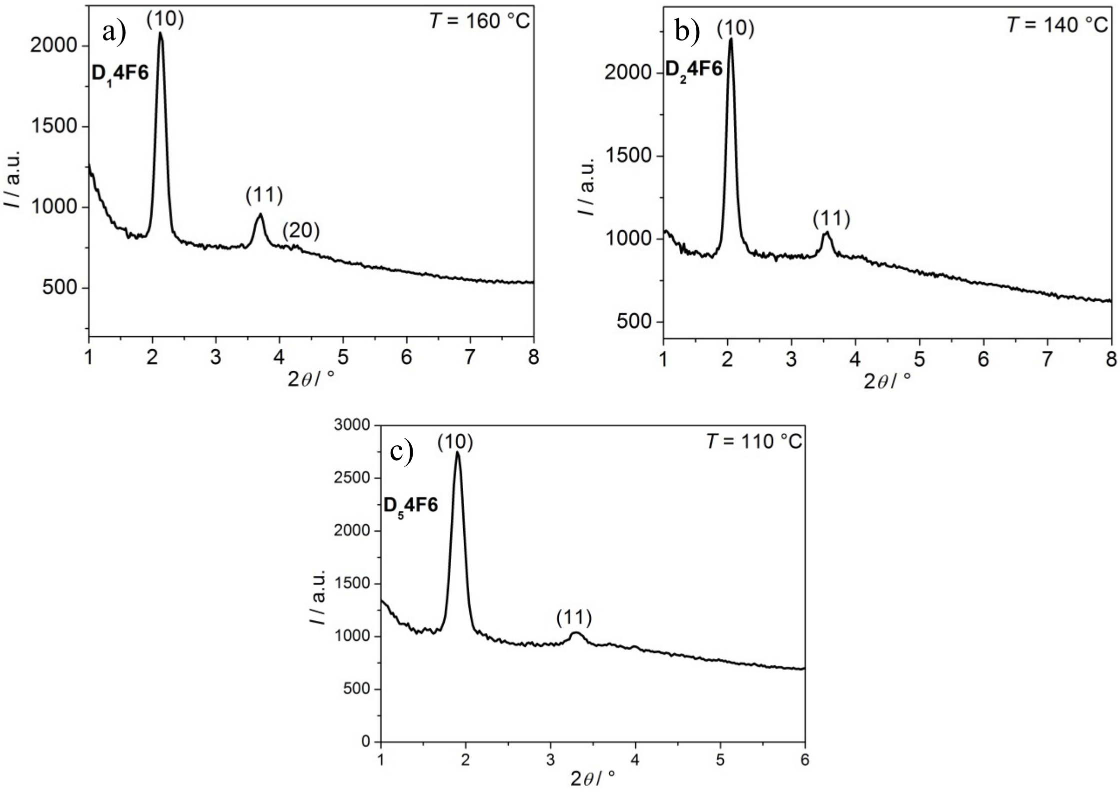

Figure S5. SAXS patterns of the LC phases of compounds a) **$D_1$4F6**, b) **$D_2$4F6,** and c) **$D_5$4F6** at the given temperatures.

1.4 WAXS data

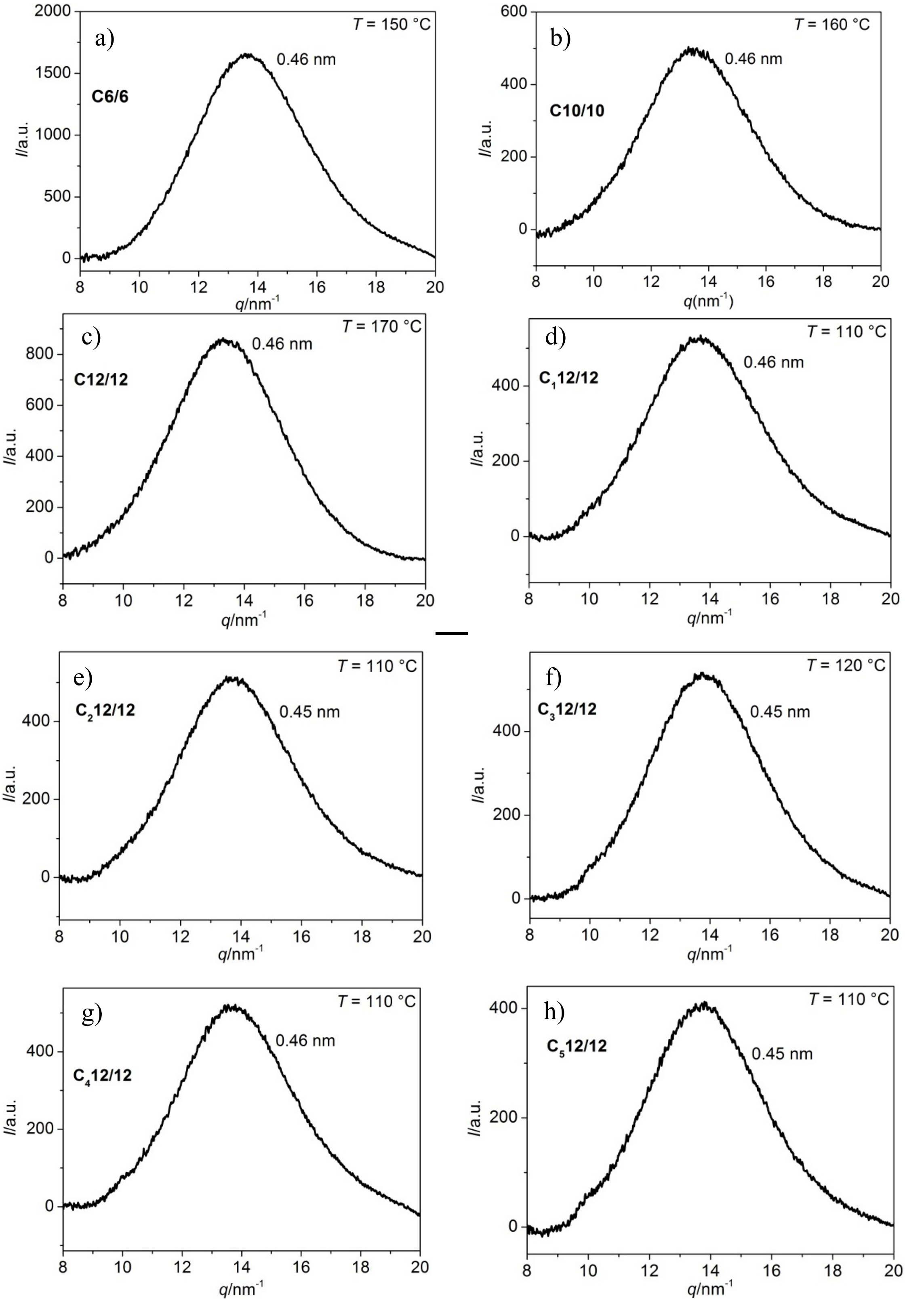

Figure S6. Wide angle scatterings of compounds a) **C6/6**, b) **C10/10** and c-h) **C_x12/12** at given temperatures.

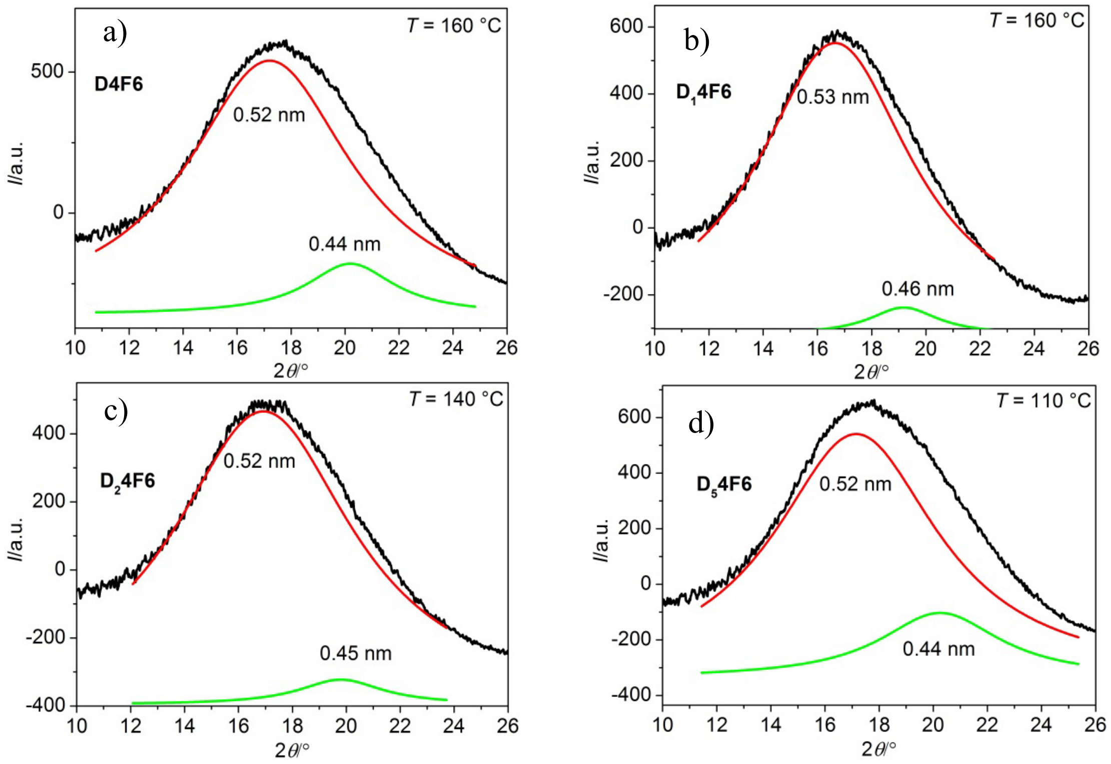

Figure S7. Wide angle scatterings of compound **D_x4F6** with fittings to two maxima.

1.5 ED map

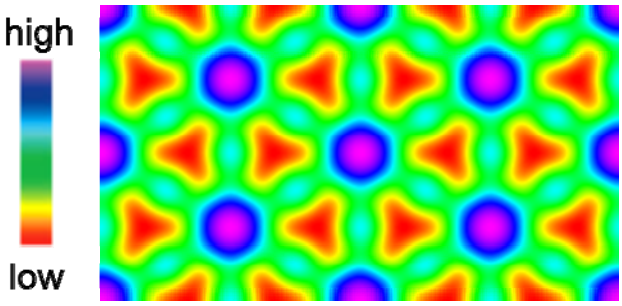

Figure S8. Reconstructed ED map of the Col_{hex} phase of **$C_5$18/18**.

1.6 Structural parameters

Table S4. Structural data of the Col_{hex} phases of compounds **C*m/m*.** [a]

Comp.	T (°C)	a_{hex} (nm)	h (nm)	V_{cell} (nm^3)	V_{mol} (nm^3)	$n_{cell,cr}$	$n_{cell,lc}$	$n_{wall,cr}$	$n_{wall,lc}$
**C4/4***	150	4.23	0.46	7.13	1.36	5.24	4.68	1.75	1.56
**C6/6**	150	4.08	0.46	6.63	1.56	4.25	3.79	1.42	1.26
**C8/8***	160	4.16	0.46	6.89	1.76	3.93	3.51	1.31	1.17
**C10/10***	130	4.35	0.46	7.54	1.95	3.87	3.46	1.29	1.15
**C12/12***	130	4.43	0.46	7.82	2.15	3.63	3.24	1.21	1.08
**C13/13***	130	4.38	0.46	7.64	2.25	3.40	2.89	1.13	1.01
**C14/14***	154	4.24	0.46	7.16	2.35	3.05	2.73	1.02	0.91

[a] h = maximum of the diffuse wide angle scattering, corresponding ti the height of the unit cell; V_{cell} = volume of the unit cell calculated according to $a_{hex}^2/2 \times 3^{1/2} \times h$; V_{mol} = volume for a single molecule as calculated using crystal volume increments; [S1] $n_{cell,cr}$ = number of molecules in the unit cell, calculated according to $n_{cell,cr} = V_{cell}/V_{mol}$ (average packing coefficient in the crystal is $k = 0.7$);[S2] $n_{cell,lc}$ = number of molecules in the unit cell in the LC state as estimated from the average of $n_{cell,cr}$ and $n_{cell,liq}$ ($n_{cell,liq}$ = number of molecules in the unit cell of an isotropic liquid with an average packing coefficient $k = 0.55$, calculated according to $n_{cell,liq} = 0.55/0.7 \times n_{cell,cr}$); $n_{wall,cr}$, $n_{wall,lc}$ = average number of molecules in the lateral cross section of the honeycomb walls calculated according to $n_{cell}/3$. * measured with Synchrotron XRD.

Table S5. Structural data of the Col_{hex} phases of compounds **C$_x$*m/m*.** [a]

Comp.	T (°C)	a_{hex} (nm)	h (nm)	V_{cell} (nm^3)	V_{mol} (nm^3)	$n_{cell,cr}$	$n_{cell,lc}$	$n_{wall,cr}$	$n_{wall,lc}$
**$C_1$12/12**	110	4.67	0.46	8.69	2.27	3.83	3.42	1.28	1.14
**$C_2$12/12**	110	4.86	0.45	9.20	2.39	3.85	3.44	1.28	1.15
**$C_3$12/12**	120	4.92	0.45	9.43	2.50	3.77	3.37	1.26	1.12
**$C_4$12/12**	110	5.12	0.46	10.44	2.62	3.98	3.13	1.56	1.18
**$C_5$12/12**	110	5.26	0.46	11.02	2.74	4.02	3.59	1.34	1.19
**$C_5$18/18***	95	5.60	0.45	12.22	3.34	3.66	3.27	1.22	1.09

[a]. for explanations, see Table S4; * measured with Synchrotron XRD.

Table S6. Structural data of the Col$_{hex}$ phases of compounds **D$_x$4F6** and **E*n*/4F6** with fluorinated chains.[a]

Comp.	T (°C)	a_{hex} (nm)	h (nm)	V_{cell} (nm^3)	V_{mol} (nm^3)	$n_{cell,cr}$	$n_{cell,lc}$	$n_{wall,cr}$	$n_{wall,lc}$
**D4F6**	160	4.51	0.44	7.93	1.52	5.21	4.65	1.74	1.55
**D$_1$4F6**	160	4.78	0.46	8.90	1.64	5.43	4.84	1.81	1.61
**D$_2$4F6**	140	4.97	0.45	9.62	1.76	5.47	4.89	1.82	1.63
**D$_3$4F6**	140	5.05	0.45	9.93	1.87	5.31	4.73	1.77	1.58
**D$_5$4F6**	110	5.35	0.45	11.15	2.11	5.28	4.72	1.76	1.57
**E12/4F6**	160	4.50	0.45	7.89	1.50	5.25	4.69	1.75	1.56
**E18/4F6**	150	4.42	0.45	7.61	1.65	4.61	4.12	1.54	1.37

[a]. for explanations, see Table S4;

2. Synthesis and Analytical Data

2.1 General remarks and procedures

Analysis

The purity was checked by thin-layer chromatography (TLC, silica gel 60 F254, Merck). Column chromatography was performed with silica gel 60 (0.063-0.2, Merck), flash-chromatography with silica gel 60 (0.040-0.063, Merck). Triethylamine was distilled from CaH_2 and stored over molecular sieve. DMF was stored over molecular sieve.
^{1}H-, ^{13}C-NMR spectra (Varian Unity 500 and Varian Unity 400 spectrometers) were recorded in $CDCl_3$ or pyridine-d$_5$ solutions, with tetramethylsilane as internal standard). Compounds **B-F** can only be dissolved in pyridine-d$_5$; all measurements were operated at 27 °C.
Elementary analysis were performed using a Leco CHNS-932 elemental analyzer.
Mass spectra were recorded with a Bruker HR-ESI-TOF. The measurements were performed in THF (1mg/mL) with 0.1 mg/mL LiCl.

Starting materials

1-Bromo-2-ethylbutane **(21a)**, *n*-bromodecane, *n*-bromododecane, *n*-bromooctane, *n*-bromotetradecane, *n*-bromoeicosane were used as obtained from *Sigma-Aldrich*. *n*-Bromohexadecane, *n*-bromooctadecane and *n*-bromodocosane were used as obtained from *abcr*. Diethyl malonate and *n*-bromobutane were used as obtained from *VEB Laborchemie Apolda*.
3-[4-(4-Ethynylphenylethynyl)phenyl]-1,2-*O*-isopropylidenepropane-1,2-diol (**6**)[S3], 1,4-dihydroxy-2,5-diiodobenzene[S4], 1,4-bis(2-octadecyleicosyl-1-oxy)-2,5-diiodobenzene (**7j**)[S5], [4-(4-ethynylphenylethynyl)phenoxy]triisopropylsilane (**13**)[S3], **C18/18**, **C20/20**, **C22/22**[S5] and 1,2-*O*-isopropylidene-1,2-dihydroxypropane-3-toluene-4-sulfonate **(30a)**[S3] were synthesized according to the procedures given in the literature.

All chiral compounds (glycerol derivatives) were used as racemic mixtures and therefore compounds **B-E** represent mixtures of 4 diasteroemers.

General procedures

P1: Dialkylation of diethyl malonate[S6]: The reaction was carried out under an argon atmosphere. Sodium hydride (2.6 equ, 60% in mineral oil) was slowly suspended in dry DMF (100 mL/~30-50 mmol malonate) and the mixture was cooled to 0 °C. Diethyl malonate (1 equ.) and the appropriate *n*-bromoalkane (3 equ) in DMF (50 mL) was added one after another and the mixture was stirred at room temperature for 3 h. After reaction water (250 mL) was added and the mixture was extracted with diethyl ether (3x 100 mL). The combined organic layers were washed with sat. aqu. LiCl, water and brine. After drying over anhydrous Na_2SO_4 the solvent was removed under reduced pressure. The residue was purified by column chromatography.

P2: Dealkoxycarbonylation[S7]: A mixture of the diethyl 2,2'-dialkylmalonate **18** (1 equ), LiCl (1.3 equ), and water (1.3 equ) in DMSO (100 mL/~50 mmol malonate) was stirred at reflux for 24 h. After cooling to room temperature water (150 mL) was added. The mixture was extracted with diethyl ether (3 x 50 mL) and the combined organic layers washed with water (3 x 50 mL). After drying over anhydrous Na_2SO_4 the solvent was removed under reduced pressure. The residue was purified by column chromatography.

P3: Reduction with $LiAlH_4$[S8]: The reaction was carried out under an argon atmosphere. $LiAlH_4$ (2.3 equ) was slowly suspended in dry diethyl ether (100 mL/~20-50 mmol carboxylate). The ethyl carboxylate **19** (1 equ) was dissolved in dry diethyl ether (100 mL) and added dropwise to the suspension. The mixture was heated to reflux for 6 h. After completion of the reaction water was added dropwise with stirring until the excess of $LiAlH_4$ was destroyed. The precipitate was dissolved by adding H_2SO_4 (10%, 50 mL) dropwise. The mixture was extracted with diethyl ether (3 x 50 mL) and the combined organic layers were washed with sat. aqu. $Na_2S_2O_3$, water and brine. After drying over anhydrous Na_2SO_4 the solvent was removed under reduced pressure and the residue was purified by column chromatography.

P4: Bromination of alkanols[S9]: The branched alkanol **20** (1 equ), Bu_4NHSO_4 (tip of a spatula) and conc. H_2SO_4 (1 mL) was suspended in HBr (48%, 30 mL) and heated to reflux for 24 h. After cooling to room temperature the mixture was extracted with diethyl ether (3 x 50 mL). The combined organic layers were washed with water and brine and dried over anhydrous Na_2SO_4. After removal of the solvent the residue was purified by column chromatography.

P5: Etherification[S 10]: A mixture of 1,4-dihydroxy-2,5-dihalobenzene (1 equ.), the bromoalkane (**21** or **23**) (2.5 equ.), K_2CO_3 (5 equ.) and Bu_4NI (tip of a spatula) in anhydrous DMF (100 mL/~5 mmol hydroquinone) was stirred at 120 °C for 12 h. After cooling to room temperature, the reaction was poured into water (50 mL) and the aqueous layer was extracted with Et_2O (3x50 mL). The combined organic layers were washed with saturated aqu. LiCl, water and brine. After drying over anhydrous Na_2SO_4, filtration and evaporation of the solvent, the crude product was purified by column chromatography.

P6: Sonogashira cross coupling reaction[S11]: A mixture of 1,4-dialkoxy-2,5-dihalobenzene **7** (1 equ.) and the appropriate acetylene **6** or **13** (2.1 equ.) was dissolved in purified Et_3N (50 mL/~5 mmol dihalobenzene). After degassing with argon for 30 min $[Pd(PPh_3)_4]$ (3 mol%)

and CuI (2 mol%) were added and the mixture was refluxed for 6 h. After removing the solvent the obtained residue was purified by column chromatography.

P7 Etherification of the oligoethyleneglycols[S 12]: 1,2-*O*-isopropylidene-3-*p*-toluenesulfonyl-*rac*-glycerol (1 equ) was dissolved in dry DMF (50-100 mL/~30 mmol) and cooled at 5 °C. After stepwise adding of NaH (3 equ) the corresponding oligoethyleneglycol (20 equ) was also added stepwise to the solution. The reaction was heated to 140 °C for 8 hours. The reaction was quenched with water and DCM was added. After the phase separation the aqueous layer was extracted with DCM. The combined organic layers were washed with water and brine and dried over Na_2SO_4. After evaporation of the solvent the obtained residue was purified by column chromatography.

P8 Tosylation[S13]: The ethyleneglycol monoether **29** (1 equ) was dissolved in dry pyridine (50 mL/~10 mmol) und cooled at -5 °C. After stepwise adding of tosyl chloride (1.1 equ), temperature should not be above 0 °C. After stirring for additional 2 hours at -5 °C the formed precipitate was filtered off. The pyridine was evaporated and the residue was dissolved in DCM. The organic layer was washed with sat. $NaHCO_3$, water and brine. After drying over $NaSO_4$ the solvent was evaporated and the obtained residue was purified by column chromatography.

P9 Bromination by exchange reaction with LiBr[S14]: The appropriate tosylate **30** (1 equ) and LiBr (3 equ) were dissolved in acetone (50 mL/~10 mmol) und refluxed for 6 hours. After the reaction was finished the solvent was evaporated and the residue was purified by column chromatography.

P10 Deprotection of T*i*PS protected phenols with Bu_4NF[S 15]: The appropriate T*i*PS protected phenol **14** (1 equ) was dissolved in THF (30 mL/~5 mmol). Afterwards $Bu_4NF \cdot 3H_2O$ (1.3 equ) was added and the mixture was stirred for 60 min at room temperature. The reaction was quenched with water and DCM was added. After phase separation the aqueous layer was extracted with DCM. The combined organic layers were washed with water and brine. After drying over $NaSO_4$ the solvent was evaporated and the residue was purified by column chromatography.

P11: Deprotection of the isopropylidene group with PPTS[S 16]**:** A mixture of the appropriate isopropylidene acetal **7** (1 equ.) and PPTS (tip of a spatula) was dissolved in THF/MeOH (1:1, 40 mL/~5 mmol) and stirred at 50 °C for 12 h. After finishing the reaction the solvent was removed and the residue solved in DCM. The organic layer was washed with $NaHCO_3$ solution (3 x 50 mL), water and brine. After drying over Na_2SO_4 the solvent was removed and the residue purified with column chromatography.

P12 Deprotection of the isopropyliden group with diluted acid[S8]: A mixture of appropriate isopropylidene acetal **16** (1 equ) and diluted hydrochloric acid (10%, 5 mL) was dissolved in MeOH (50 mL/~1-5 mmol) and stirred at reflux for 6 h. The mixture was extracted with EtOAc and the organic phase was washed with saturated $NaHCO_3$-solution, water and brine. After drying over Na_2SO_4 the solvent was removed and the residue was used without further purification.

P13 Hydrogenolysis of the benzyl protection group[S9]: The protected phenol **26** (1 equ) is dissolved in THF (100 mL/~2-3 mmol) and degassed. Pd/C (10 wt % Pd, 0.3 g) is added and the mixture is stirred under hydrogen atmosphere (30 psi) overnight at 40 °C. After this the

mixture is filtere through celite and the solvent is removed under reduced pressure. The resiude is purified by column chromatography.

2.2 Synthesis of the 1,4-dialkoxy-2,5-diiodobenzenes with branched alkyl chains (5)

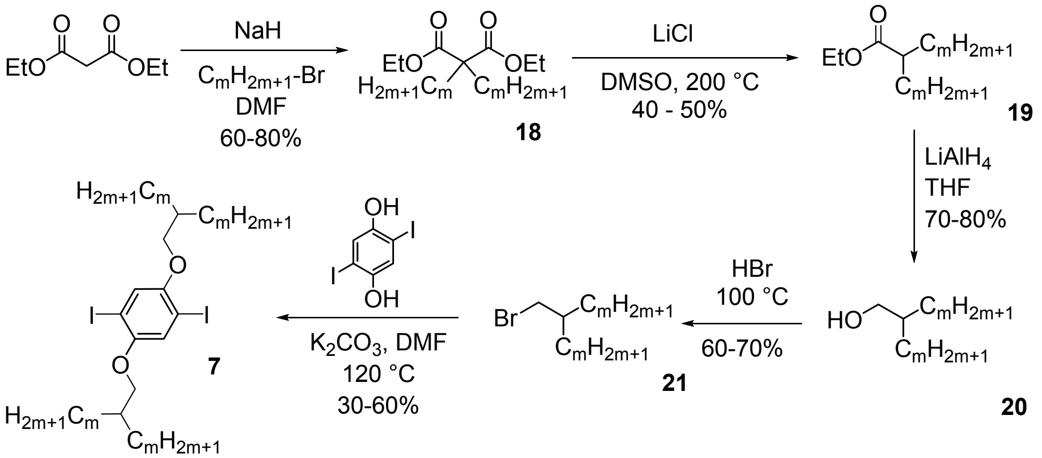

Scheme S1. Synthesis of the branched alkyl bromides and the symmetric the 1,4-dialkoxy-2,5-diiodobenzenes (**7**).

2.2.1 1-Bromo-2-butylhexane (21b)

Diethyl 2,2-dibutylmalonate (18b): Synthesized according to P1 from diethyl malonate (10.00 g, 62.5 mmol), *n*-bromobutane (25.70 g, 0.19 mol) and NaH (7.50 g, 0.19 mol, 60% in mineral oil) in DMF (200 mL). Purification by column chromatography (eluent: $CHCl_3$/*n*-hexane: 1:1). Colourless liquid, $C_{15}H_{28}O_4$, M = 272.20 g/mol, yield: 16.48 g (97%), **^{1}H-NMR** (400 MHz, $CDCl_3$) δ 4.17 (q, ^3J(H,H) = 7.1 Hz, 4H, –O–CH_2–CH_3), 1.93 – 1.80 (m, 4H, –CH_2–), 1.44 – 1.03 (m, 14H, –O–CH_2–CH_3), 0.89 (t, ^3J(H,H) = 7.3 Hz, 6H, –CH_3) ppm.

Ethyl 2-butylhexaonate (19b): Synthesized according to P2 from **18b** (16.48 g, 60.3 mmol), LiCl (3.30 g, 78.4 mmol) and H_2O (1.40 g, 78.4 mmol, 60% in mineral oil) in DMSO (100 mL). Purification by column chromatography (eluent: $CHCl_3$/*n*-hexane: 1:1). Colourless liquid, $C_{12}H_{24}O_2$, M = 200.18 g/mol, yield: 12.05 g (98%), **^{1}H-NMR** (500 MHz, $CDCl_3$) δ 4.13 (q, ^3J(H,H) = 7.1 Hz, 2H, –OCH_2–), 2.35 – 2.25 (m, 1H, –CH–), 1.68 – 1.52 (m, 2H, –CH_2–), 1.49 – 1.15 (m, 11H, –CH_2–, –CH_3), 0.88 (t, ^3J(H,H) = 7.1 Hz, 6H, , –CH_3) ppm.

2-Butylhexane-1-ol (20b): Synthesized according to P3 from **19b** (12.05 g, 60.3 mmol) and $LiAlH_4$ (3.40 g, 90.0 mmol) in Et_2O (100 mL). Purification by column chromatography (eluent: $CHCl_3$). Colourless liquid, $C_{10}H_{22}O$, M = 158.17 g/mol, yield: 6.90 g (73%), **^{1}H-NMR** (400 MHz, $CDCl_3$) δ 3.54 (t, ^3J(H,H) = 5.4 Hz, 2H, –CH_2–OH), 1.52 – 1.13 (m, 14H, –CH_2–, –CH–, –OH), 0.90 (t, ^3J(H,H) = 6.6 Hz, 6H, –CH_3) ppm.

1-Bromo-2-butylhexane (21b): Synthesized according to P4 from **20b** (16.48 g, 60.3 mmol), Bu_4NHSO_4 (tip of spatula), HBr (40 mL, 48%) and H_2SO_4 (2 mL). Purification by column chromatography (eluent: *n*-hexane). Colourless liquid, $C_{10}H_{21}Br$, M = 164.02 g/mol, yield: 6.28 g (65%), **^{1}H-NMR** (400 MHz, $CDCl_3$) δ 3.45 (d, ^3J(H,H) = 4.8 Hz, 2H, –CH_2–Br), 1.58 (m, 1H, –CH–), 1.47 – 1.15 (m, 12H, –CH_2–), 0.91 (t, ^3J(H,H) = 6.9 Hz, 6H, –CH_3) ppm.

2.2.2 1-Bromo-2-hexyloctane (21c)

Diethyl 2,2-dioctylmalonate (18c): Synthesized according to P1 from diethyl malonate (5.00 g, 31.3 mmol), *n*-bromohexane (11.90 g, 71.9 mmol) and NaH (2.90 g, 71.9 mmol, 60% in mineral oil) in DMF (100 mL). Purification by column chromatography (eluent: $CHCl_3$/*n*-hexane: 1:1). Colourless oil, $C_{19}H_{36}O_4$, M = 328.26 g/mol, yield: 10.15 g (97%), **^{1}H-NMR** (500 MHz, $CDCl_3$) δ 4.18 (q, ^3J(H,H) = 7.1 Hz, 4H, –OCH_2–), 1.92 – 1.83 (m, 4H, –CH_2–), 1.40 – 1.09 (m, 22H, –CH_2–, –CH_3), 0.88 (t, ^3J(H,H) = 6.9 Hz, 6H, –CH_3) ppm.

Ethyl 2-octyldecanoate (19c): Synthesized according to P2 from **18c** (11.15 g, 31.0 mmol), LiCl (1.70 g, 40.0 mmol) and H_2O (0.72 g, 40.0 mmol) in DMSO (100 mL). Purification by column chromatography (eluent: $CHCl_3$/*n*-hexane: 1:1). Colourless oil, $C_{16}H_{32}O_2$, M = 256.24 g/mol, yield: 4.69 g (59%), **^{1}H-NMR** (400 MHz, $CDCl_3$) δ 4.13 (q, ^3J(H,H) = 7.1 Hz, 2H, –OCH_2–), 2.36 – 2.25 (m, 1H, –CH–), 1.67 – 1.10 (m, 23H, –CH_2–, –CH_3), 0.87 (t, ^3J(H,H) = 6.9 Hz, 6H, –CH_3) ppm.

2-Hexyloctane-1-ol (20c): Synthesized according to P3 from **19c** (4.69 g, 28.3 mmol) and $LiAlH_4$ (2.10 g, 54.9 mmol) in Et_2O (100 mL). Purification by column chromatography (eluent: $CHCl_3$). Colourless oil, $C_{14}H_{30}O$, M = 214.23 g/mol, yield: 2.40 g (62%), **^{1}H-NMR** (400 MHz, $CDCl_3$) δ 3.59 – 3.49 (m, 2H, –CH_2–OH), 1.51 – 1.40 (m, 1H, –CH–), 1.37 – 1.20 (m, 20H, –CH_2–), 1.16 – 1.10 (m, 1H, –OH), 0.94 – 0.83 (m, 6H, –CH_3) ppm.

1-Bromo-2-hexyloctane (21c): Synthesized according to P4 from **20c** (2.40 g, 11.3 mmol), Bu_4NHSO_4 (tip of spatula), HBr (30 mL, 48%) and H_2SO_4 (1 mL). Purification by column chromatography (eluent: *n*-hexane). Colourless oil, $C_{14}H_{29}Br$, M = 276.15 g/mol, yield: 1.95 g (80%), **^{1}H-NMR** (400 MHz, $CDCl_3$) δ 3.45 (d, ^3J(H,H) = 4.8 Hz, 2H, –CH_2–Br), 1.68 – 1.58 (m, 1H, –CH–), 1.45 – 1.20 (m, 20H, –CH_2–), 0.89 (t, ^3J(H,H) = 6.8 Hz, 6H, –CH_3) ppm.

2.2.3 1-Bromo-2-octyldecane (21d)

Diethyl 2,2-dioctylmalonate (18d): Synthesized according to P1 from diethyl malonate (5.00 g, 31.3 mmol), *n*-bromooctane (18.10 g, 93.8 mmol) and NaH (2.90 g, 71.9 mmol, 60% in mineral oil) in DMF (100 mL). Purification by column chromatography (eluent: $CHCl_3$/*n*-hexane: 1:1). Colourless liquid, $C_{23}H_{44}O_4$, M = 384.32 g/mol, yield: 11.32 g (94%), **^{1}H-NMR** (500 MHz, $CDCl_3$) δ 4.18 (q, ^3J(H,H) = 7.1 Hz, 4H, –OCH_2–), 1.91 – 1.82 (m, 4H, –CH_2–), 1.39 – 1.07 (m, 30H, –CH_2–, –CH_3), 0.88 (t, ^3J(H,H) = 7.0 Hz, 6H, –CH_3) ppm.

Ethyl 2-octyldecanoate (19d): Synthesized according to P2 from **18d** (11.32 g, 29.5 mmol), LiCl (1.62 g, 38.3 mmol) and H_2O (0.68 g, 38.3 mmol) in DMSO (100 mL). Purification by column chromatography (eluent: $CHCl_3$/*n*-hexane: 1:1). Colourless liquid, $C_{20}H_{40}O_2$, M = 312.30 g/mol, yield: 7.29 g (79%), **^{1}H-NMR** (400 MHz, $CDCl_3$) δ 4.13 (q, ^3J(H,H) = 7.2 Hz, 2H, –OCH_2–), 2.36 – 2.25 (m, 1H, –CH–), 1.67 – 1.03 (m, 31H, –CH_2–, –CH_3), 0.87 (t, ^3J(H,H) = 6.9 Hz, 6H, –CH_3) ppm.

2-Octyldecane-1-ol (20d): Synthesized according to P3 from **19d** (7.29 g, 23.4 mmol) and $LiAlH_4$ (2.00 g, 53.2 mmol) in Et_2O (100 mL). Purification by column chromatography (eluent: $CHCl_3$). Colourless liquid, $C_{18}H_{38}O$, M = 270.29 g/mol, yield: 5.08 g (81%), **^{1}H-NMR** (400 MHz, $CDCl_3$) δ 3.54 (d, ^3J(H,H) = 5.5 Hz, 2H, –CH_2–OH), 1.50 – 1.39 (m, 1H, –CH–), 1.46 – 1.20 (m, 28H, –CH_2–), 1.17 – 1.08 (m, 1H, –OH), 0.88 (t, ^3J(H,H) = 6.9 Hz, 6H, –CH_3) ppm.

1-Bromo-2-octyldecane (21d): Synthesized according to P4 from **20d** (5.08 g, 18.9 mmol), Bu_4NHSO_4 (tip of spatula), HBr (30 mL, 48%) and H_2SO_4 (1 mL). Purification by column chromatography (eluent: *n*-hexane). Colourless liquid, $C_{18}H_{37}Br$, M = 332.21 g/mol, yield: 4.42 g (71%), **^{1}H-NMR** (400 MHz, $CDCl_3$) δ 3.44 (d, ^3J(H,H) = 4.8 Hz, 2H, –CH_2–Br), 1.67 – 1.58 (m, 1H, –C*H*–), 1.42 – 1.20 (m, 28H, –CH_2–), 0.88 (t, ^3J(H,H) = 6.9 Hz, 6H, –CH_3) ppm.

2.2.4 1-Bromo-2-decyldodecane (21e)

Diethyl 2,2-didecylmalonate (18e): Synthesized according to P1 from diethyl malonate (5.00 g, 31.3 mmol), *n*-bromodecane (17.30 g, 78.1 mmol) and NaH (3.10 g, 78.1 mmol, 60% in mineral oil) in DMF (100 mL). Purification by column chromatography (eluent: $CHCl_3$/*n*-hexane: 1:1). Colourless liquid, $C_{27}H_{52}O_4$, M = 440.39 g/mol, yield: 12.80 g (93%), **^{1}H-NMR** (400 MHz, $CDCl_3$) δ 4.17 (q, ^3J(H,H) = 7.1 Hz, 4H, –OCH_2–), 1.91 – 1.80 (m, 4H, –CH_2–), 1.39 – 1.04 (m, 38H, –CH_2–, –CH_3), 0.88 (t, ^3J(H,H) = 6.8 Hz, 6H, –CH_3) ppm.

Ethyl 2-decyldodecanoate (19e): Synthesized according to P2 from **18e** (12.80 g, 29.1 mmol), LiCl (1.60 g, 37.8 mmol) and H_2O (0.68 g, 37.8 mmol) in DMSO (50 mL). Purification by column chromatography (eluent: $CHCl_3$/*n*-hexane: 1:1). Colourless liquid, $C_{24}H_{48}O_2$, M = 368.37 g/mol, yield: 7.74 g (72%), **^{1}H-NMR** (400 MHz, $CDCl_3$) δ 4.13 (q, ^3J(H,H) = 7.1 Hz, 2H, –OCH_2–), 2.36 – 2.25 (m, 1H, –C*H*–), 1.69 – 1.10 (m, 39H, –CH_2–, –CH_3), 0.88 (t, ^3J(H,H) = 6.9 Hz, 6H, –CH_2–) ppm.

2-Decyldodecane-1-ol (20e): Synthesized according to P3 from **19e** (7.74 g, 21.0 mmol) and $LiAlH_4$ (1.24 g, 33.6 mmol) in Et_2O (100 mL). Purification by column chromatography (eluent: $CHCl_3$). Colourless liquid, $C_{22}H_{46}O$, M = 326.35 g/mol, yield: 6.33 g (92%), **^{1}H-NMR** (400 MHz, $CDCl_3$) δ 3.54 (d, ^3J(H,H) = 5.5 Hz, 2H, –CH_2–OH), 1.60 – 1.50 (m, 1H, –C*H*–), 1.45 – 1.20 (m, 37H, –CH_2–, –O*H*), 0.88 (t, ^3J(H,H) = 6.8 Hz, 6H, –CH_3) ppm.

1-Bromo-2-decyldodecane (21e): Synthesized according to P4 from **20e** (6.33 g, 19.4 mmol), Bu_4NHSO_4 (tip of spatula), HBr (30 mL, 48%) and H_2SO_4 (1 mL). Purification by column chromatography (eluent: *n*-hexane). Colourless liquid, $C_{22}H_{45}Br$, M = 388.27 g/mol, yield: 4.05 g (54%), **^{1}H-NMR** (400 MHz, $CDCl_3$) δ 3.44 (d, ^3J(H,H) = 4.8 Hz, 2H, –CH_2–Br), 1.67 – 1.54 (m, 1H, –C*H*–), 1.45 – 1.17 (m, 36H, –CH_2–), 0.88 (t, ^3J(H,H) = 6.8 Hz, 6H, –CH_3) ppm.

2.2.5 1-Bromo-2-dodecyltetradecane (21f)

Diethyl 2,2-didodecylmalonate (18f): Synthesized according to P1 from diethyl malonate (5.00 g, 31.3 mmol), *n*-bromododecane (23.3 g, 93.8 mmol) and NaH (2.25 g, 93.8 mmol, 60% in mineral oil) in DMF (100 mL). Purification by column chromatography (eluent: $CHCl_3$/*n*-hexane: 1:1). Colourless liquid, $C_{31}H_{60}O_4$, M = 496.45 g/mol, yield: 13.09 g (84%), **^{1}H-NMR** (400 MHz, $CDCl_3$) δ 4.17 (q, ^3J(H,H) = 7.1 Hz, 4H, –OCH_2–), 1.92 – 1.78 (m, 4H, –CH_2–), 1.39 – 1.04 (m, 46H, –CH_2–, –CH_3), 0.88 (t, ^3J(H,H) = 6.8 Hz, 6H, –CH_3) ppm.

Ethyl 2-dodecyltetradecanoate (19f): Synthesized according to P2 from **18f** (13.09 g, 26.2 mmol), LiCl (2.30 g, 52.6 mmol) and H_2O (0.47 g, 26.3 mmol) in DMSO (100 mL). Purification by column chromatography (eluent: $CHCl_3$/*n*-hexane: 1:1). Colourless liquid, $C_{28}H_{56}O_2$, M = 424.43 g/mol, yield: 8.40 g (76%), **^{1}H-NMR** (400 MHz, $CDCl_3$) δ 4.16 – 4.06 (m, 2H, –OCH_2–), 2.37 – 2.22 (m, 1H, –C*H*–), 1.59 – 1.24 (m, 47H, –CH_2–, –CH_3), 0.88 (t, ^3J(H,H) = 6.8 Hz, 6H, –CH_3) ppm.

2-Dodecyltetradecane-1-ol (20f): Synthesized according to P3 from **19f** (8.40 g, 19.8 mmol) and $LiAlH_4$ (1.00 g, 25.8 mmol) in Et_2O (150 mL). Purification by column chromatography (eluent: $CHCl_3$). White solid, $C_{26}H_{54}O$, M = 382.42 g/mol, yield: 5.90 g (78%), **^{1}H-NMR** (400 MHz, $CDCl_3$) δ 3.54 (t, ^3J(H,H) = 5.6 Hz, 2H, –CH_2–OH), 1.45 – 1.40 (m, 1H, –C*H*–), 1.40 – 1.20 (m, 44 H, –CH_2–), 1.14 (t, ^3J(H,H) = 5.7 Hz, 1H, –O*H*), 0.88 (t, ^3J(H,H) = 6.8 Hz, 6H, –CH_3) ppm.

1-Bromo-2-dodecyltetradecane (21f): Synthesized according to P4 from **20f** (2.00 g, 5.2 mmol), Bu_4NHSO_4 (tip of spatula), HBr (50 mL, 48%) and H_2SO_4 (1 mL). Purification by column chromatography (eluent: *n*-hexane). Colourless liquid, $C_{26}H_{53}Br$, M = 444.33 g/mol, yield: 1.50 g (65%), **^{1}H-NMR** (400 MHz, $CDCl_3$) δ 3.44 (d, ^3J(H,H) = 4.8 Hz, 2H, –CH_2–Br), 1.64 – 1.55 (m, 1H, –C*H*–), 1.55 – 1.07 (m, 44H, –CH_2–), 0.88 (t, ^3J(H,H) = 6.8 Hz, 6H, –CH_3) ppm.

2.2.6 1-Bromo-2-tridecylpentadecane (21g)

Diethyl 2,2-ditridecylmalonate (18g): Synthesized according to P1 from diethyl malonate (8.00 g, 50.5 mmol), *n*-bromotridecane (25.00 g, 0.11 mol) and NaH (6.00 g, 0.15 mol, 60% in mineral oil) in DMF (100 mL). Purification by column chromatography (eluent: $CHCl_3$/*n*-hexane: 1:1). Colourless liquid, $C_{33}H_{64}O_4$, M = 524.48 g/mol, yield: 21.90 g (92%), **^{1}H-NMR** (400 MHz, $CDCl_3$) δ 4.17 (q, ^3J(H,H) = 7.1 Hz, 4H, –OCH_2–), 1.92 – 1.79 (m, 4H, –CH_2–), 1.57 – 1.06 (m, 50H, –CH_2–, –CH_3), 0.88 (t, ^3J(H,H) = 6.8 Hz, 6H, –CH_3) ppm.

Ethyl 2-tridecylpentadecanoate (19g): Synthesized according to P2 from **18g** (21.90 g, 46.6 mmol), LiCl (2.60 g, 60.6 mmol) and H_2O (1.10 g, 60.6 mmol) in DMSO (100 mL). Purification by column chromatography (eluent: $CHCl_3$/*n*-hexane: 1:1). Colourless liquid, $C_{30}H_{60}O_2$, M = 452.46 g/mol, yield: 9.70g (52%), **^{1}H-NMR** (400 MHz, $CDCl_3$) δ 4.13 (q, ^3J(H,H) = 7.1 Hz, 2H, –OCH_2–), 2.36 – 2.25 (m, 1H, –C*H*–), 1.68 – 1.06 (m, 51H, –CH_2–, –CH_3), 0.88 (t, ^3J(H,H) = 6.8 Hz, 6H, –CH_3) ppm.

2-Tridecylpentadecane-1-ol (20g): Synthesized according to P3 from **19g** (9.70 g, 24.3 mmol) and $LiAlH_4$ (1.00 g, 25.5 mmol) in Et_2O (150 mL). Purification by column chromatography (eluent: $CHCl_3$). White solid, $C_{28}H_{58}O$, M = 410.45 g/mol, mp. 48 °C, yield: 7.35 g (85%), **^{1}H-NMR** (400 MHz, $CDCl_3$) δ 3.54 (t, ^3J(H,H) = 5.6 Hz, 2H, –CH_2–OH), 1.60 – 1.19 (m, 49H, –C*H*–, –CH_2–), 1.14 (t, ^3J(H,H) = 5.7 Hz, 1H, –O*H*), 0.88 (t, ^3J(H,H) = 6.8 Hz, 6H, –CH_3) ppm.

1-Bromo-2-tridecylpentadecane (21g): Synthesized according to P4 from **20g** (7.35 g, 20.6 mmol), Bu_4NHSO_4 (tip of spatula), HBr (50 mL, 48%) and H_2SO_4 (1 mL). Purification by column chromatography (eluent: *n*-hexane). White solid, $C_{28}H_{57}Br$, M = 472.36 g/mol, mp. 48 °C, yield: 4.60 g (53%), **^{1}H-NMR** (400 MHz, $CDCl_3$) δ 3.44 (d, ^3J(H,H) = 4.7 Hz, 2H, –CH_2–Br), 1.67 – 1.58 (m, 1H, –C*H*–), 1.42 – 1.12 (m, 48H, –CH_2–), 0.88 (t, ^3J(H,H) = 6.7 Hz, 6H, –CH_3) ppm.

2.2.7 1-Bromo-2-tetradecylhexadecane (21h)

Diethyl 2,2-ditetradecylmalonate (18h): Synthesized according to P1 from diethyl malonate (5.00 g, 31.3 mmol), *n*-bromotetradecane (19.90 g, 71.9 mmol) and NaH (3.75 g, 93.8 mmol, 60% in mineral oil) in DMF (100 mL). Purification by column chromatography (eluent: $CHCl_3$/*n*-hexane: 1:1). White solid, $C_{35}H_{68}O_4$, M = 552.51 g/mol, mp. 39 °C, yield: 13.60 g

(79%), ^{1}**H-NMR** (400 MHz, $CDCl_3$) δ 4.17 (q, ^3J(H,H) = 7.1 Hz, 4H, –OCH_2–), 1.92 – 1.80 (m, 4H, –CH_2–), 1.57 – 1.07 (m, 54H, –CH_2–, –CH_3), 0.88 (t, ^3J(H,H) = 6.8 Hz, 6H, –CH_3) ppm.

Ethyl 2-tetradecylhexadecanoate (19h): Synthesized according to P2 from **18h** (13.60 g, 24.6 mmol), LiCl (1.04 g, 24.6 mmol) and H_2O (0.44 g, 24.6 mmol) in DMSO (50 mL). Purification by column chromatography (eluent: $CHCl_3$/*n*-hexane: 1:1). White solid, $C_{32}H_{64}O_2$, *M* = 480.49 g/mol, mp. 37 °C, yield: 11.05 g (93%), ^{1}**H-NMR** (400 MHz, $CDCl_3$) δ 4.13 (q, ^3J(H,H) = 7.1 Hz, 2H, –OCH_2–), 2.36 – 2.24 (m, 1H, –C*H*–), 1.68 – 1.06 (m, 55H, –CH_2–, –CH_3), 0.88 (t, ^3J(H,H) = 6.8 Hz, 6H, –CH_3) ppm.

2-Tetradecylhexadecane-1-ol (20h): Synthesized according to P3 from **19h** (11.05 g, 23.0 mmol) and $LiAlH_4$ (1.13 g, 29.9 mmol) in Et_2O (200 mL). Purification by column chromatography (eluent: $CHCl_3$). White solid, $C_{30}H_{62}O$, *M* = 438.48 g/mol, mp. 51 °C, yield: 8.03 g (80%), ^{1}**H-NMR** (400 MHz, $CDCl_3$) δ 3.53 (t, ^3J(H,H) = 5.5 Hz, 2H, –CH_2–OH), 1.59 – 1.20 (m, 53H, –C*H*–, –CH_2–), 1.15 (t, ^3J(H,H) = 5.6 Hz, 1H, –O*H*), 0.88 (t, ^3J(H,H) = 6.7 Hz, 6H, –CH_3) ppm.

1-Bromo-2-tetradecylhexadecane (21h): Synthesized according to P4 from **20h** (8.03 g, 18.04 mmol), Bu_4NHSO_4 (tip of spatula), HBr (50 mL, 48%) and H_2SO_4 (1 mL). Purification by column chromatography (eluent: *n*-hexane). White solid, $C_{30}H_{61}Br$, *M* = 500.40 g/mol, mp. 42 °C, yield: 5.99 g (65%), ^{1}**H-NMR** (400 MHz, $CDCl_3$) δ 3.44 (d, ^3J(H,H) = 4.8 Hz, 2H, –CH_2–Br), 1.65 – 1.58 (m, 1H, –C*H*–), 1.49 – 1.18 (m, 52H, –CH_2–), 0.88 (t, ^3J(H,H) = 6.8 Hz, 6H, –CH_3) ppm.

2.2.8 1-Bromo-2-hexadecyloctadecane (21i)

Diethyl 2,2-dihexadecylmalonate (18i): Synthesized according to P1 from diethyl malonate (5.00 g, 31.3 mmol), *n*-bromohexadecane (28.60 g, 93.8 mmol) and NaH (3.80 g, 93.8 mmol, 60% in mineral oil) in DMF (100 mL). Purification by column chromatography (eluent: $CHCl_3$/*n*-hexane: 1:1). White solid, $C_{39}H_{76}O_4$, *M* = 608.57 g/mol, mp = 38 °C, yield: 13.04 g (67%), ^{1}**H-NMR** (400 MHz, $CDCl_3$) δ 4.17 (q, ^3J(H,H) = 7.1 Hz, 4H, –OCH_2–), 1.91 – 1.79 (m, 4H, –CH_2–), 1.41 – 1.05 (m, 62H, –CH_2–, –CH_3), 0.88 (t, ^3J(H,H) = 6.7 Hz, 6H, –CH_3) ppm.

Ethyl 2-hexadecyloctadecanoate (19i): Synthesized according to P2 from **18i** (13.04 g, 21.4 mmol), LiCl (1.18 g, 27.9 mmol) and H_2O (0.50 g, 27.9 mmol) in DMSO (50 mL). Purification by column chromatography (eluent: $CHCl_3$/*n*-hexane: 1:1). White solid, $C_{36}H_{72}O_2$, *M* = 536.55 g/mol, mp. 41 °C, yield: 9.05 g (79%), ^{1}**H-NMR** (400 MHz, $CDCl_3$) δ 4.13 (q, ^3J(H,H) = 7.1 Hz, 2H, –OCH_2–), 2.37 – 2.22 (m, 1H, –C*H*–), 1.67 – 1.06 (m, 57H, –CH_2–, –CH_3), 0.88 (t, ^3J(H,H) = 6.8 Hz, 6H, –CH_3) ppm.

2-Hexadecyloctadecane-1-ol (20i): Synthesized according to P3 from **19i** (9.05 g, 16.9 mmol) and $LiAlH_4$ (1.00 g, 25.3 mmol) in Et_2O (100 mL). Purification by column chromatography (eluent: $CHCl_3$). White solid, $C_{34}H_{70}O$, *M* = 438.48 g/mol, mp. 60 °C, yield: 4.11 g (50%), ^{1}**H-NMR** (400 MHz, $CDCl_3$) δ 3.54 (t, ^3J(H,H) = 5.6 Hz, 2H, –CH_2–OH), 1.51 – 1.06 (m, 62H, –C*H*–, –CH_2–, –O*H*), 0.88 (t, ^3J(H,H) = 6.8 Hz, 6H, –CH_3) ppm.

1-Bromo-2-hexadecyloctadecane (21i): Synthesized according to P4 from **20i** (4.11 g, 8.4 mmol), Bu_4NHSO_4 (tip of spatula), HBr (40 mL, 48%) and H_2SO_4 (2 mL). Purification by column chromatography (eluent: *n*-hexane). White solid, $C_{34}H_{69}Br$, *M* = 556.46 g/mol, mp. 56

°C, yield: 2.87 g (62%), ^{1}**H-NMR** (500 MHz, $CDCl_3$) δ 3.44 (d, ^{3}J(H,H) = 4.8 Hz, 2H, –CH_2–Br), 1.62 – 1.57 (m, 1H, –C*H*–), 1.65 – 1.10 (m, 60H, –CH_2–), 0.88 (t, ^{3}J(H,H) = 7.0 Hz, 6H, –CH_3) ppm.

2.2.9 1,4-Dialkoxy-2,5-diiodobenzenes (7)

1,4-Bis(2-ethylbutyl-1-oxy)-2,5-diiodobenzene (7a): Synthesized according to P5 from **21a** (0.52 g, 3.2 mmol), 1,4-dihydroxy-2,5-diiodobenzene (0.50 g, 1.4 mmol), K_2CO_3 (0.95 g, 6.9 mmol) and Bu_4NI (tip of spatula) in DMF (50 mL). Purification by column chromatography (eluent: *n*-hexane). Colourless liquid, $C_{18}H_{28}I_2O_2$, *M* = 530.02 g/mol, yield: 0.24 g (33%), ^{1}**H-NMR** (500 MHz, $CDCl_3$) δ 7.17 (s, 2H, Ar–*H*), 3.82 (d, ^{3}J(H,H) = 5.4 Hz, 4H, –OCH_2–), 1.74 – 1.61 (m, 2H, –C*H*–), 1.62 – 1.38 (m, 8H, –CH_2–), 0.94 (t, ^{3}J(H,H) = 7.5 Hz, 12H, –CH_3) ppm.

1,4-Bis(2-butylhexyl-1-oxy)-2,5-diiodobenzene (7b): Synthesized according to P5 from **21b** (1.04 g, 4.7 mmol), 1,4-dihydroxy-2,5-diiodobenzene (0.75 g, 2.1 mmol), K_2CO_3 (1.45 g, 10.5 mmol) and Bu_4NI (tip of spatula) in DMF (50 mL). Purification by column chromatography (eluent: *n*-hexane). Colourless liquid, $C_{26}H_{44}I_2O_2$, *M* = 642.14 g/mol, yield: 0.61 g (45%), ^{1}**H-NMR** (500 MHz, $CDCl_3$) δ 7.15 (s, 2H, Ar–*H*), 3.80 (d, ^{3}J(H,H) = 5.5 Hz, 4H, –OCH_2–), 1.84 – 1.73 (m, 2H, –C*H*–), 1.56 – 1.22 (m, 24H, –CH_2–), 0.91 (t, ^{3}J(H,H) = 7.0 Hz, 12H, –CH_3) ppm.

1,4-Bis(2-hexyloctyl-1-oxy)-2,5-diiodobenzene (7c): Synthesized according to P5 from **21c** (1.52 g, 5.5 mmol), 1,4-dihydroxy-2,5-diiodobenzene (1.00 g, 2.6 mmol), K_2CO_3 (1.80 g, 13 mmol) and Bu_4NI (tip of spatula) in DMF (50 mL). Purification by column chromatography (eluent: *n*-hexane). Colourless liquid, $C_{34}H_{60}I_2O_2$, *M* = 754.27 g/mol, yield: 0.67 g (33%), ^{1}**H-NMR** (400 MHz, $CDCl_3$) δ 7.15 (s, 2H, Ar–*H*), 3.80 (d, ^{3}J(H,H) = 5.4 Hz, 4H, –OCH_2–), 1.85 – 1.71 (m, 2H, –C*H*–), 1.60 – 1.19 (m, 40H, –CH_2–), 0.95 – 0.83 (m, 12H, –CH_3) ppm.

1,4-Bis(2-octyldecyl-1-oxy)-2,5-diiodobenzene (7d): Synthesized according to P5 from **21d** (1.25 g, 3.8 mmol), 1,4-dihydroxy-2,5-diiodobenzene (0.68 g, 1.9 mmol), K_2CO_3 (1.30 g, 9.4 mmol) and Bu_4NI (tip of spatula) in DMF (50 mL). Purification by column chromatography (eluent: *n*-hexane). Colourless liquid, $C_{42}H_{76}I_2O_2$, *M* = 866.39 g/mol, yield: 1.11 g (68%), ^{1}**H-NMR** (400 MHz, $CDCl_3$) δ 7.15 (s, 2H, Ar–*H*), 3.80 (d, ^{3}J(H,H) = 5.4 Hz, 4H, –OCH_2–), 1.87 – 1.70 (m, 2H, –C*H*–), 1.68 – 1.15 (m, 56H, –CH_2–), 0.88 (t, ^{3}J(H,H) = 6.8 Hz, 12H, –CH_3) ppm.

1,4-Bis(2-decyldodecyl-1-oxy)-2,5-diiodobenzene (7e): Synthesized according to P5 from **21e** (2.22 g, 5.8 mmol), 1,4-dihydroxy-2,5-diiodobenzene (1.00 g, 2.7 mmol), K_2CO_3 (2.00 g, 14.0 mmol) and Bu_4NI (tip of spatula) in DMF (50 mL). Purification by column chromatography (eluent: *n*-hexane). Colourless liquid, $C_{50}H_{92}I_2O_2$, *M* = 978.52 g/mol, mp. 44 °C, yield: 1.37 g (52%), ^{1}**H-NMR** (400 MHz, $CDCl_3$) δ 7.15 (s, 2H, Ar–*H*), 3.80 (d, ^{3}J(H,H) = 5.4 Hz, 4H, –OCH_2–), 1.84 – 1.73 (m, 2H, –C*H*–), 1.58 – 1.19 (m, 72H, –CH_2–), 0.88 (t, ^{3}J(H,H) = 6.9 Hz, 12H, –CH_3) ppm.

1,4-Bis(2-dodecyltetradecyl-1-oxy)-2,5-diiodobenzene (7f): Synthesized according to P5 from **21f** (1.90 g, 4.2 mmol), 1,4-dihydroxy-2,5-diiodobenzene (0.75 g, 2.1 mmol), K_2CO_3 (0.70 g, 5.0 mmol) and Bu_4NI (tip of spatula) in DMF (50 mL). Purification by column chromatography (eluent: *n*-hexane). White solid, $C_{58}H_{108}I_2O_2$, *M* = 1090.64 g/mol, mp. 58 °C, yield: 1.20 g (53%), ^{1}**H-NMR** (400 MHz, $CDCl_3$) δ 7.15 (s, 2H, Ar–*H*), 3.79 (d, ^{3}J(H,H) =

5.4 Hz, 4H, –OCH_2–), 1.83 – 1.75 (m, 2H, –C*H*–), 1.59 – 1.16 (m, 88H, –CH_2–), 0.88 (t, ^3J(H,H) = 6.8 Hz, 12H, –CH_3) ppm.

1,4-Bis(2-tridecylpentadecyl-1-oxy)-2,5-diiodobenzene (7g): Synthesized according to P5 from **21g** (2.00 g, 4.8 mmol), 1,4-dihydroxy-2,5-diiodobenzene (0.80 g, 2.3 mmol), K_2CO_3 (1.60 g, 11.5 mmol) and Bu_4NI (tip of spatula) in DMF (50 mL). Purification by column chromatography (eluent: *n*-hexane). White solid, $C_{62}H_{116}I_2O_2$, *M* = 1146.71 g/mol, mp. 89 °C, yield: 0.85 g (36%), **^{1}H-NMR** (400 MHz, $CDCl_3$) δ 7.15 (s, 2H, Ar–*H*), 3.80 (d, ^3J(H,H) = 5.3 Hz, 4H, –OCH_2–), 1.86 – 1.72 (m, 2H, –C*H*–), 1.62 – 1.15 (m, 96H, –CH_2–), 0.88 (t, ^3J(H,H) = 6.7 Hz, 12H, –CH_3) ppm.

1,4-Bis(2-tetradecylhexadecyl-1-oxy)-2,5-diiodobenzene (7h): Synthesized according to P5 from **21h** (0.63 g, 3.6 mmol), 1,4-dihydroxy-2,5-diiodobenzene (0.63 g, 1.7 mmol), K_2CO_3 (1.17 g, 8.5 mmol) and Bu_4NI (tip of spatula) in DMF (50 mL). Purification by column chromatography (eluent: *n*-hexane). White solid, $C_{66}H_{124}I_2O_2$, *M* = 1202.77 g/mol, mp. 64 °C, yield: 0.57 g (28%), **^{1}H-NMR** (500 MHz, $CDCl_3$) δ 7.15 (s, 2H, Ar–*H*), 3.80 (d, ^3J(H,H) = 5.4 Hz, 4H, –OCH_2–), 1.83 – 1.72 (m, 2H, –C*H*–), 1.57 – 1.09 (m, 104H, –CH_2–), 0.88 (t, ^3J(H,H) = 7.0 Hz, 12H, –CH_3) ppm.

1,4-Bis(2-hexadecyloctadecyl-1-oxy)-2,5-diiodobenzene (7i): Synthesized according to P5 from **21i** (1.75 g, 3.2 mmol), 1,4-dihydroxy-2,5-diiodobenzene (0.50 g, 1.4 mmol), K_2CO_3 (1.00 g, 7.0 mmol) and Bu_4NI (tip of spatula) in DMF (50 mL). Purification by column chromatography (eluent: *n*-hexane). White solid, $C_{74}H_{140}I_2O_2$, *M* = 1314.89 g/mol, mp. 70 °C, yield: 0.63 g (35%), **^{1}H-NMR** (500 MHz, $CDCl_3$) δ 7.15 (s, 2H, Ar–*H*), 3.80 (d, ^3J(H,H) = 5.4 Hz, 4H, –OCH_2–), 1.82 – 1.72 (m, 2H, –C*H*–), 1.56 – 1.11 (m, 120H, –CH_2–), 0.88 (t, ^3J(H,H) = 7.0 Hz, 12H, –CH_3) ppm.

2.3 Synthesis of the 1,4-disubstituted-2,5-dihalobenzenes with semiperfluorinated alkyl chains (7)

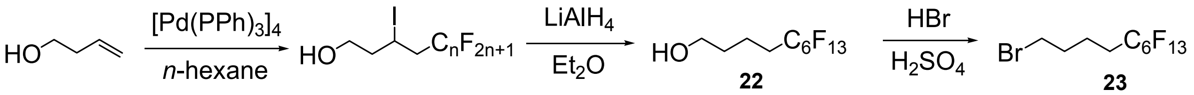

Scheme S2. Synthesis of the semiperfluorinated alkyl bromides.

2.3.1 1,4-Diiodo-2,4-bis(5,5,6,6,7,7,8,8,9,9,10,10,10-tridecafluoro-*n*-decyl-1-oxy)benzene (7k)

5,5,6,6,7,7,8,8,9,9,10,10,10-Tridecafluorodecane-1-ol (22): 3-Buten-1-ol (1.62 g, 22.00 mmol) was dissolved in *n*-hexane and 1-iodo-1,1,2,2,3,3,4,4,5,5,6,6,6-tridecafluordecane (10.00g, 22.00 mmol) was added and the solution was degassed. [Pd(PPh_3)$_4$] (1.27 g, 1.10 mmol) was added and the mixture was stirred for 10 days at room temperature. After the reaction the solvent was removed under reduced pressure. The residue was dissolved in diethylther (100 mL) and slowly dropped in a suspension of $LiAlH_4$ (2.50 g, 66.00 mmol) in Diethylether (50 mL). After addition the mixture was refluxed for 6 h. $LiAlH_4$ was hydrolyzed using MeOH and the resulting residue was dissolved using diluted hydrochloric acid. After extraction with diethylether (3x 50 mL) the organic phase was washed with $Na_2S_2O_3$ solution, water and brine. The solvent was removed and the residue purified by flash chromatography (eluent: CH_2Cl_2/Et_2O = 10:0.2). Colourless oil, $C_{10}H_9F_{13}O$, *M* = 392.16

g/mol, yield: 6.51 g (78%), **^{1}H-NMR** (400 MHz, $CDCl_3$) δ 3.70 (t, ^3J(H,H) = 6.0 Hz, 2H, –CH_2OH), 2.10 (m, 2H, –C$H_2$$CF_2$–), 1.69 (m, 4 H, –C$H_2$$CH_2CF_2$–, –C$H_2$$CH_2CH_2CF_2$–) ppm.

.

1-Bromo-5,5,6,6,7,7,8,8,9,9,10,10-tridecafluorodecane (23): Syntheszied according to P4 from **23** (6.51 g, 0.02 mol), hydrobromic acid (50 mL), Bu_4NHSO_4 (0.05 g, 0.2 mmol) and H_2SO_4 (2 mL). Purificationby column chromatography (eluent: *n*-pentane). Colourless oil, $C_{10}H_8BrF_{13}$, *M* = 455.05 g/mol, yield: 2.50 g (33%), **^{1}H-NMR** (400 MHz, $CDCl_3$) δ 3.43 (t, ^3J(H,H) = 6.5 Hz, 2H, –CH_2Br), 2.10 (m, 2H, –CH_2Br), 1.96 (m, 2H, –C$H_2$$CH_2$Br), 1.81 (m, 2H, –C$H_2$$CF_2$–) ppm.

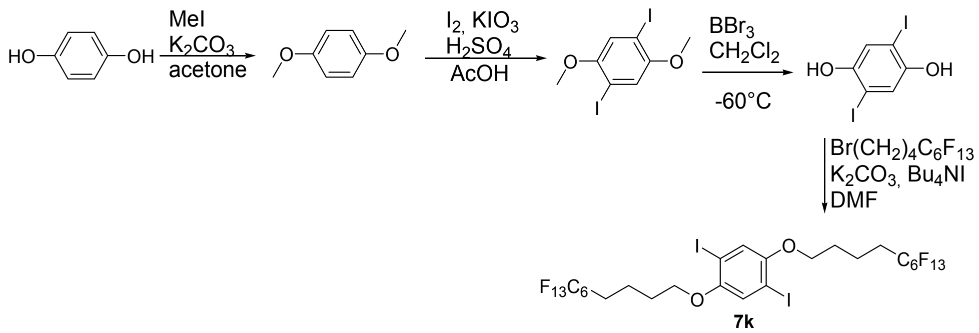

Scheme S3. Synthesis of the 2,5-disubstituted 1,4-diiodohydroquinones with two semiperfluorinated chains (**7k**).

1,4-Diiodo-2,4-bis(5,5,6,6,7,7,8,8,9,9,10,10,10-tridecafluoro-*n*-decyl-1-oxy)benzene (7k): Synthesized according to P5 from **23** (1.51 g, 3.3 mmol), 1,4-dihydroxy-2,5-diiodobenzene (0.60 g, 1.7 mmol), K_2CO_3 (0.55 g, 4.0 mmol) and Bu_4NI (tip of spatula) in DMF (50 mL). Purification by column chromatography (eluent: *n*-hexane/EtOAc = 9/1). White solid, $C_{26}H_{28}F_{26}I_2O_2$, *M* = 1110.19 g/mol, mp. 89 °C, yield: 1.30 g (73%), **^{1}H-NMR** ($CDCl_3$, 400 MHz) δ 7.18 (s, 2H, Ar–*H*), 3.98 (t, ^3J(H,H) = 5.4 Hz, 4H, –OCH_2–), 2.14 (m, 4H, –OCH_2CH_2–), 1.90 (m, 8 H, –CH_2–) ppm.

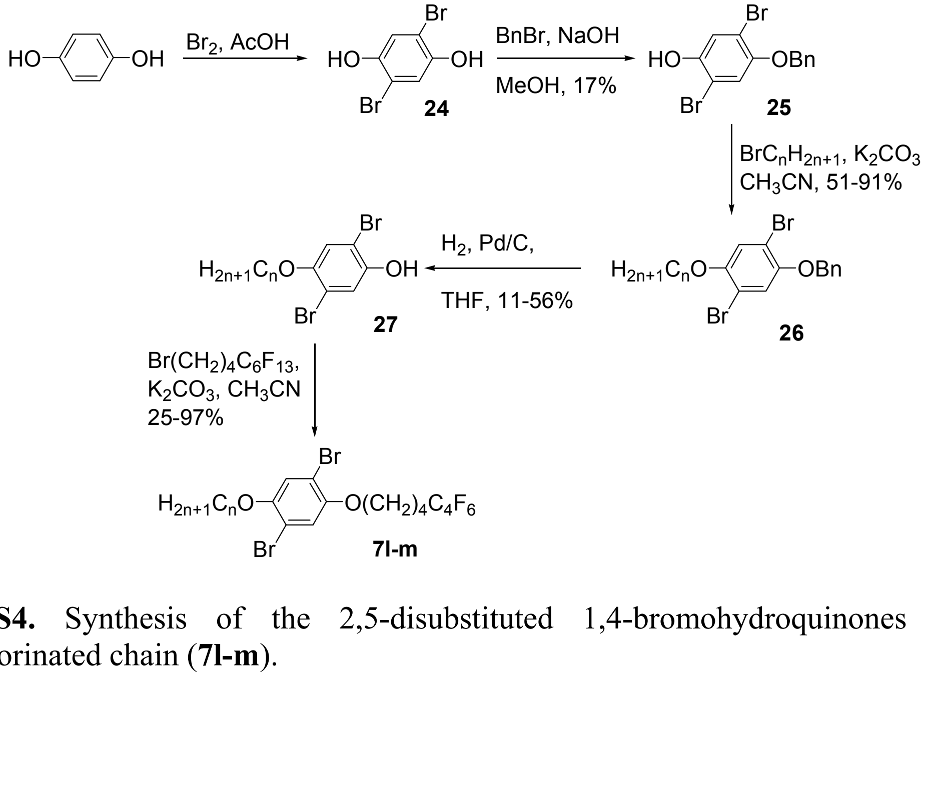

Scheme S4. Synthesis of the 2,5-disubstituted 1,4-bromohydroquinones with one semiperfluorinated chain (**7l-m**).

2.3.2 1,4-Dibromo-5-dodecyloxy-2-(5,5,6,6,7,7,8,8,9,9,10,10,10-tridecafluorodecyloxy)-benzene (19)

2,5-Dibromohydroquinone (24)[S17]: To a solution of hydroquinone (22.00 g, 0.2 mol) in acetic acid (100 mL) a solution of bromine (20.9 mL, 0.4 mol) in acetic acid (50 mL) was added slowly. The solution was stirred for 1 h at room temperature. The acetic acid was removed by distillation and the residue was recrystallized twice from MeOH/water (1:1). Colourless solid, $C_6H_4Br_2O_2$, *M* = 267.90 g/mol, yield: 15.20 g (28%), mp. 186 – 188 °C, **^{1}H-NMR** ($CDCl_3$, 400 MHz) δ 7.14 (s, 2H, Ar–*H*), 5.21 (s, 2H, –O*H*) ppm.

4-Benzyloxy-2,5-dibromophenol (25)[S10]: 2,5-Dibromohydroquinone (**24**) (15.23 g, 0.06 mol) and NaOH (2.28 g, 0.06 mol) are suspended in MeOH (150 mL). A solution of benzylchloride (7.20 g, 0.06 mol) in MeOH (30 mL) is dropped slowly to the suspension and the mixture is heated for 2 h at reflux. The mixture is allowed to cool to room temperature and conc. hydrochloric acid (20 mL) is added. After extraction with diethylether (3x 50 mL) the organic phase is washed with sat. $NaHCO_3$-sol. (50 mL) and water (50 mL). The solvent is removed under reduced pressure and the residue is purified by column chromatography (eluent: $CHCl_3$). Colourless solid, $C_{13}H_{10}Br_2O_2$, M = 358.01 g/mol, yield: 5.20 g (26%), mp. 110 – 112 °C, **^{1}H-NMR** ($CDCl_3$, 400 MHz) δ 7.45 – 7,31 (m, 5H, Ar–*H*), 7.25 (s, 1H, Ar–*H*), 7.03 (s, 1H, Ar–*H*), 5.13 (s, 1H, –O*H*), 5.04 (s, 2H, –OCH_2Ph) ppm.

1-Benzyloxy-2,5-dibromo-4-dodecyloxybenzene (26l): Synthesized according to P5 from 4-benzyloxy-2,5-dibromophenol (**25**) (2.00 g, 5.59 mmol), 1-bromododecane (1.53 g, 6.14 mmol), K_2CO_3 (0.85 g, 6.14 mmol) in acetonitrile (50 mL). Recrystallisation from petroleum ether. White solid, $C_{25}H_{34}Br_2O_2$, *M* = 526.35 g/mol, yield: 1.50 g (51%), mp. 49 – 51 °C, **^{1}H NMR** (400 MHz, $CDCl_3$) δ 7.45 – 7.31 (m, 5H, Ar–*H*), 7.14 (s, 1H, Ar–*H*), 7.09 (s, 1H, Ar–*H*), 5.05 (s, 2H, –OCH_2Ph), 3.94 (t, ^3J(H,H) = 6.4 Hz, 2H, –OCH_2–), 1.82 – 1.75 (m, 2H, –OCH$_2$CH_2–), 1.50 – 1.42 (m, 2H, –OCH$_2$CH$_2$CH_2–), 1.33 – 1.25 (m, 16H, –OCH$_2$CH$_2$CH$_2$(CH_2)$_8$–), 0.87 (t, ^3J(H,H) = 7.1 Hz, 3H, –CH$_2$CH_3) ppm.

1-Benzyloxy-2,5-dibromo-4-octadecyloxybenzene (26m): Synthesized according to P5 from 4-benzyloxy-2,5-dibromophenol (**25**) (2.57 g, 7.18 mmol), 1-bromooctadecane (2.63 g, 7.89 mmol), K_2CO_3 (1.09 g, 7.89 mmol) in acetonitrile (80 mL). Recrystallisation from $CHCl_3$/MeOH. White solid, $C_{31}H_{46}Br_2O_2$, *M* = 610.50 g/mol, yield: 3.5 g (79%), mp. 63 – 65 °C, **^{1}H-NMR** ($CDCl_3$, 400 MHz) δ 7.45 - 7,31 (m, 5H, Ar–*H*), 7.14 (s, 1H, Ar–*H*), 7.08 (s, 1H, Ar–*H*), 5.05 (s, 2H, –OCH_2Ph), 3.93 (t, ^3J(H,H) = 6.4 Hz, 2H, –OCH_2CH$_2$CH$_2$–), 1.79 (m, 2H, –OCH$_2$CH_2CH$_2$–), 1.46 (m, 2H, –OCH$_2$CH$_2$CH_2–), 1.40-1,24 (m, 28H, –CH_2–), 0.86 (t, ^3J(H,H) = 6.6 Hz, 3H, –CH_3) ppm.

2,5-Dibromo-4-dodecyloxyphenol (27l): Synthesized according to P13 from 1-benzyloxy-2,5-dibromo-4-dodecyloxybenzol (**26l**) (1.50 g, 2.85 mmol), Pd/C (10 wt % Pd, 0.30 g) in THF (100 mL). Purification by column chromatography (eluent: *n*-hexane/$CHCl_3$ = 2:1). Recrystallization from *n*-hexane. White solid, $C_{18}H_{28}Br_2O_2$, *M* = 436.22 g/mol, yield: 0.70 g (56%), mp. 56 – 59 °C, **^{1}H NMR** (400 MHz, $CDCl_3$) δ 7.22 (s, 1H, Ar–*H*), 6.96 (s, 1H, Ar–*H*), 5.09 (s, 1H, –O*H*), 3.91 (t, ^3J(H,H) = 6.5 Hz, 2H, –OCH_2), 1.87 – 1.70 (m, 2H, –OCH$_2$CH_2–), 1.50 – 1.40 (m, 2H, –OCH$_2$CH$_2$CH_2–), 1.40 – 1.17 (m, 16H, –OCH$_2$CH$_2$CH$_2$(C*H2*)$_8$–), 0.86 (t, ^3J(H,H) = 6.8 Hz, 3H, –CH$_2$CH_3–) ppm.

2,5-Dibromo-4-octadecyloxyphenol (27m): Synthesized according to P13 from 1-benzyloxy-2,5-dibromo-4-octadecyloxybenzene (**26m**) (1.50 g, 2.5 mmol), Pd/C (10 wt % Pd, 0.3 g) in THF (100 mL). Purification by column chromatography (eluent: $CHCl_3$). White

solid, $C_{24}H_{40}Br_2O_2$, *M* = 520.38 /mol, yield: 1.00 g (81%), mp. 71 – 73 °C, **^{1}H-NMR** ($CDCl_3$, 400 MHz) δ 7.22 (s, 1H, Ar–*H*), 6.96 (s, 1H, Ar–*H*), 5.09 (s, 1H, –O*H*), 3.91 (t, ^3J(H,H) = 6.4 Hz, 2H, $–OCH_2CH_2CH_2–$), 1.78 (m, 2H, $–OCH_2CH_2CH_2–$), 1.46 (m, 2H, –$OCH_2CH_2CH_2–$), 1.40-1,25 (m, 28H, $–CH_2–$), 0.86 (t, ^3J(H,H) = 6.6 Hz, 3H, $–CH_3$) ppm.

1,4-Dibromo-5-dodecyloxy-2-(5,5,6,6,7,7,8,8,9,9,10,10,10-tridecafluorodecyl-1-oxy)benzene (7l): Synthesized according to P5 from 2,5-dibromo-4-dodecyloxyphenole (**27l**) (470 mg, 1.08 mmol), 5,5,6,6,7,7,8,8,9,9,10,10,10-tridecafluordecylbromide (490 mg , 1.08 mmol), K_2CO_3 (744 mg, 5.39 mmol) in acetonitrile (40 mL). No further purification. White solid, $C_{28}H_{35}Br_2F_{13}O_2$, *M* = 810.37 g/mol, yield: 720 mg (82%), mp. 50-52 °C, **^{1}H NMR** (400 MHz, CDCl3) δ 7.07 (s, 2H, Ar–*H*), 3.98 (t, ^3J(H,H) = 5.6 Hz, 2H, $–OCH_2–$), 3.93 (t, ^3J(H,H) = 6.5 Hz, 2H, $–OCH_2–$), 2.28 – 2.10 (m, 2H, $–CH_2CF_2–$), 1.95 – 1.82 (m, 4H, $–OCH_2CH_2–$), 1.82 – 1.65 (m, 2H $–CH_2CH_2CF_2–$), 1.50 – 1.40 (m, 2H, $–OCH_2CH_2CH_2–$), 1.38 – 1.17 (m, 16H, $–OCH_2CH_2CH_2(CH_2)_8–$), 0.86 (t, ^3J(H,H) = 6.8 Hz, 3H, $–CH_3$) ppm.

1,4-Dibromo-2-octadecyloxy-5-(5,5,6,6,7,7,8,8,9,9,10,10,10-tridecafluorodecyl-1-oxy)-benzene (7m): Synthesized according to P5 from 2,5-dibromo-4-octadecyloxyphenol (**27m**) (0.50 g, 1.0 mmol), 5,5,6,6,7,7,8,8,9,9,10,10,10-tridecafluordecylbromide (0.48 g, 1.1 mmol), K_2CO_3 (0.26 g, 1.9 mmol) in acetonitrile (40 mL). Purification by column chromatography (eluent: *n*-hexane/$CHCl_3$ = 3:1). White solid, $C_{34}H_{47}Br_2F_{13}O_2$, *M* = 894.53 g/mol, yield: 630 mg (73%), mp. 63 – 67 °C, **^{1}H-NMR** ($CDCl_3$, 400 MHz) δ 7.07 (s, 2H, Ar–*H*), 3.98 (t, ^3J(H,H) = 6.8 Hz, 2H, $–OCH_2(CH_2)_2CH_2CF_2–$), 3.93 (t, ^3J(H,H)= 6.4 Hz, 2H, $–OCH_2–$), 2.19 (m, 2H, $–CH_2CF_2–$), 1.88 (m, 4H, $–OCH_2(CH_2)_2CH_2CF_2–$), 1.78 (m, 2H, $–OCH_2CH_2–$), 1.46 (m, 2H, $–OCH_2CH_2CH_2–$), 1.40-1.24 (m, 28H, $–CH_2–$), 0.86 (t, ^3J(H,H) = 6.6 Hz, 3H, $-CH_3$) ppm.

2.4 Preparation of the hydrophilic building blocks (1,2-diols 17 and corresponding acetonides 16)

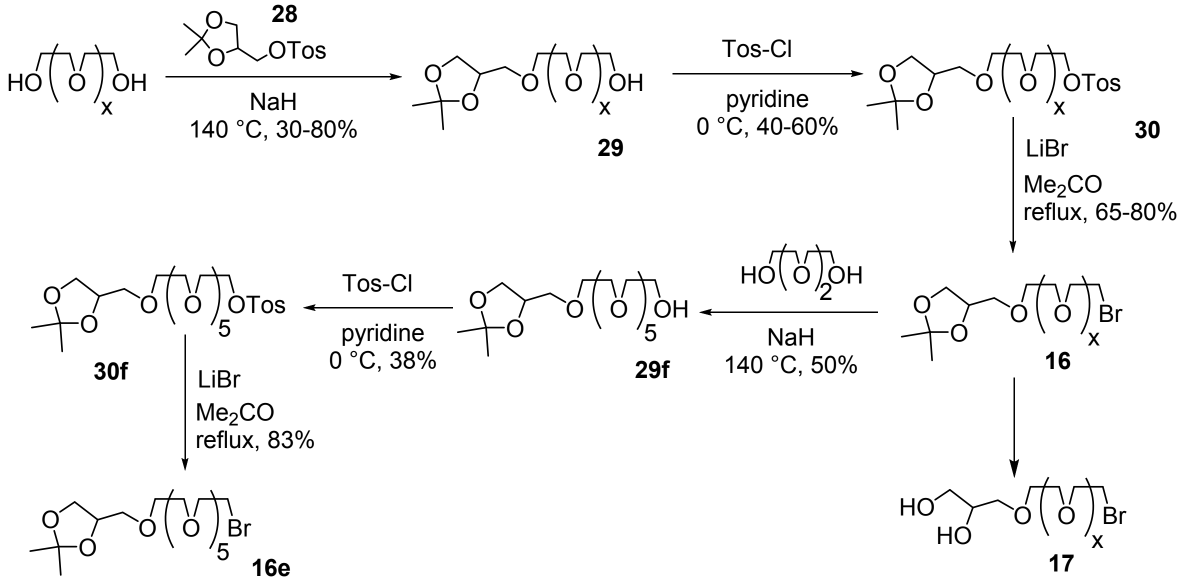

Scheme S5. Synthesis of the isopropylidene protected hydrophilic units (**16**) and hydrophilic units (**17**).

2.4.1 1,2-*O*-Isopropylidene-3-bromopropane-1,2-diol (16a) and 3-bromopropane-1,2-diol (17a)

1,2-*O*-Isopropylidene-3-bromopropane-1,2-diol (16a): Synthesized according to P9 from 1,2-*O*-isopropylidene-3-(*p*-toluolsulfonyloxy)propane-1,2-diol (**28**)[S8] (21.44 g, 0.1 mol), LiBr (19.28 g, 0.2 mol) in acetone (100 mL). Purification by column chromatography (eluent: $CHCl_3$), colourless oil, $C_6H_{11}BrO_2$, *M* = 195.05 g/mol, yield: 14.67 g (99%), **^{1}H-NMR** ($CDCl_3$, 400 MHz) δ 4.34 (m, 1H, –CH_2C*H*CH_2–), 4.13 (dd, ^3J(H,H) = 6.4 Hz, ^4J(H,H)= 1.4 Hz, 1H, –OCH_2–), 3.86 (m, 1H, –OCH_2–), 3.43 (m, 1H, BrCH_2–), 3.31 (m, 1H, BrCH_2–), 1.44 (s, 3H, –OCH(CH_3)$_2$), 1.35 (s, 3H, –OCH(CH_3)$_2$) ppm.

3-Bromopropane-1,2-diol (17a): Synthesized according to P12 from **16a** (14.67 g, 0.1 mol), HCl (10%, 5 mL) in MeOH (100mL). colourless oil, $C_3H_7BrO_2$, *M* = 154.99 g/mol, yield: 4.62 g (41%), **^{1}H-NMR** ($CDCl_3$, 400 MHz) δ 3.94 (m, 1H, –CH_2C*H*CH_2–), 3.78 (m, 1H, –OCH_2–), 3.69 (m, 1H, –OCH_2–), 3.56 – 3.43 (m, 2H, BrCH_2–) ppm.

2.4.2 6-Bromo-4-oxahexane-1,2-diol (17b)

1,2-*O*-Isopropylidene-4-oxahexane-1,2,6-triol (29b): Synthesized according to P7 from **28** (10.00 g, 35.0 mmol), ethyleneglycol (21.7 g, 0.35 mol) and NaH (3.50 g, 87.5 mmol) in DMF (100 mL). Purification by column chromatography (eluent: $CHCl_3$/MeOH = 9:1). Colourless liquid, $C_8H_{16}O_4$, *M* = 176.10 g/mol, K_p = 105 °C (0.8 mbar), yield: 5.10 g (83%), **^{1}H-NMR** (400 MHz, $CDCl_3$) δ 4.32 – 4.26 (m, 1H, –C*H*–), 4.06 (dd, ^3J(H,H) = 8.2 Hz, ^3J(H,H) = 6.6 Hz, 1H, –CH_2–), 3.80 – 3.69 (m, 3H, –CH_2–), 3.67 – 3.50 (m, 4H, –CH_2–), 2.15 (s, 1H, –O*H*), 1.43 (s, 3H, –CH_3), 1.37 (s, 3H, –CH_3) ppm.

1,2-*O*-Isopropylidene-4-oxa-1,2-dihydroxyhexane-6-toluene-4-sulfonate (30b): Synthesized according to P8 from **29b** (5.10 g, 29.0 mmol) and TosCl (6.10 g, 31.8 mmol) in dry pyridine (50 mL). Purification by column chromatography (eluent: *n*-hexane/ethylacetate = 1:1). Colourless liquid, $C_{15}H_{22}O_6S$, *M* = 330.11 g/mol, yield: 1.84 g (42%), **^{1}H-NMR** (400 MHz, $CDCl_3$) δ 7.84 – 7.76 (m, 2H, Ar–*H*), 7.39 – 7.31 (m, 2H, Ar–*H*), 4.20 – 4.12 (m, 3H, –C*H*–, –CH_2–), 4.00 (dd, ^3J(H,H) = 8.2 Hz, ^3J(H,H) = 6.6 Hz, 1H, –CH_2–), 3.73 – 3.61 (m, 3H, –CH_2–), 3.50 (dd, ^3J(H,H) = 10.0 Hz, ^3J(H,H) = 5.7 Hz, 1H, –CH_2–), 3.44 (dd, ^3J(H,H) = 10.0 Hz, ^3J(H,H) = 5.2 Hz, 1H, –CH_2–), 2.45 (s, 3H, –CH_3), 1.39 (s, 3H, –CH_3), 1.34 (s, 3H, –CH_3) ppm.

1,2-*O*-Isopropylidene-6-bromo-4-oxahexane-1,2-diol (16b): Synthesized according to P9 from **30b** (1.84 g, 5.6 mmol) and LiBr (1.45 g, 16.7 mmol) in acetone (100 mL). Purification by column chromatography (eluent: $CHCl_3$). Colourless liquid, $C_8H_{15}BrO_3$, *M* = 238.02 g/mol, yield: 1.25 g (95%), **^{1}H-NMR** (400 MHz, $CDCl_3$) δ 4.34 – 4.21 (m, 1H, –C*H*–), 4.07 (dd, ^3J(H,H) = 8.2 Hz, ^3J(H,H) = 6.5 Hz, 1H, –CH_2–), 3.90 – 3.73 (m, 3H, –CH_2–), 3.68 – 3.41 (m, 4H, –O–CH_2–CH_2–Br), 1.43 (s, 3H, –CH_3), 1.36 (s, 3H, –CH_3) ppm.

6-Bromo-4-oxahexane-1,2-diol (17b): Synthesized according to P12 from **16b** (2.04 g, 0.01 mol), HCl (10%, 5 mL) in MeOH (50 mL). Colourless oil, $C_5H_{11}BrO_3$, *M* = 199.04 g/mol, yield: 800 mg (50%), **^{1}H-NMR** ($CDCl_3$, 400 MHz) δ 3.80 (m, 1H, –CH_2C*H*CH_2–), 3.76 – 3.55 (m, 6H, –OCH_2–), 3.49 (t, ^3J(H,H) = 5.8 Hz, 2H, BrCH_2–), 2.62 (s, 1H, –O*H*), 2.09 (s, 1 H, –O*H*).

2.4.3 9-Bromo-4,7-dioxanonan-1,2-diol (17c)

1,2-*O*-Isopropylidene-4,7-dioxanonan-1,2,9-triol (29c): Synthesized according to P7 from **28** (10.00 g, 35.0 mmol), diethyleneglycol (68.60 g, 0.70 mol) and NaH (4.90 g, 0.12 mol) in DMF (50 mL). Purification by column chromatography (eluent: $CHCl_3$/MeOH = 95:5). Colourless liquid, $C_{10}H_{20}O_5$, M = 220.13 g/mol, yield: 2.15 g (29%), **^{1}H-NMR** (400 MHz, $CDCl_3$) δ 4.35 – 4.22 (m, 1H), 4.12 – 3.98 (m, 1H, –C*H*–), 3.85 – 3.45 (m, 11H, –CH_2–), 2.38 (t, ^3J(H,H) = 5.4 Hz, 1H, –O*H*), 1.42 (s, 3H, –CH_3), 1.35 (s, 3H, –CH_3) ppm.

1,2-*O*-Isopropylidene-1,2-dihydroxy-4,7-dioxanon-9-yltoluene-4-sulfonate (30c): Synthesized according to P8 from **29c** (2.15 g, 10.1 mmol) and TosCl (3.20 g, 11.2 mmol) in dry pyridine (50 mL). Purification by column chromatography (eluent: *n*-hexane/ethylacetate = 1:1). Colourless liquid, $C_{17}H_{26}O_7S$, M = 374.14 g/mol, yield: 1.60 g (43%), **^{1}H-NMR** (400 MHz, $CDCl_3$) δ 7.84 – 7.77 (m, 2H, Ar–*H*), 7.37 – 7.32 (m, 2H, Ar–*H*), 4.32 – 4.21 (m, 1H, –C*H*–), 4.18 – 4.14 (m, 2H, –CH_2–), 4.04 (dd, ^3J(H,H) = 8.3 Hz, ^3J(H,H) = 6.4 Hz, 1H, –CH_2–), 3.73 – 3.66 (m, 3H, –CH_2–), 3.63 – 3.43 (m, 6H, –CH_2–), 2.45 (s, 3H, –CH_3), 1.41 (s, 3H, –CH_3), 1.35 (s, 3H, –CH_3) ppm.

9-Bromo-1,2-*O*-isopropylidene-1,2-dihydroxy-4,7-dioxanonane (16c): Synthesized according to P9 from **30c** (1.60 g, 4.4 mmol) and LiBr (1.14 g, 13.1 mmol) in acetone (50 mL). Purification by column chromatography (eluent: $CHCl_3$/MeOH = 95:5). Colourless liquid, $C_{10}H_{19}BrO_4$, M = 282.05 g/mol, yield: 1.08 g (87%), **^{1}H-NMR** (400 MHz, $CDCl_3$) δ 4.33 – 4.26 (m, 1H, –C*H*–), 4.06 (dd, ^3J(H,H) = 8.3 Hz, ^3J(H,H) = 6.4 Hz, 1H, –CH_2–), 3.81 (t, ^3J(H,H) = 6.3 Hz, 2H, –CH_2–), 3.77 – 3.63 (m, 5H, –CH_2–), 3.59 (dd, ^3J(H,H) = 10.0 Hz, ^3J(H,H) = 5.7 Hz, 1H, –CH_2–), 3.52 (dd, ^3J(H,H) = 10.1 Hz, ^3J(H,H) = 5.4 Hz, 1H, –CH_2–), 3.47 (t, ^3J(H,H) = 6.3 Hz, 2H, –CH_2–), 1.42 (s, 3H, –CH_3), 1.36 (s, 3H, –CH_3) ppm.

9-Bromo-4,7-dioxanonan-1,2-diol (17c): Synthesized according to P12 from **16c** (1.17 g, 4.00 mmol), HCl (10%, 5 mL) in MeOH (50 mL). Colourless oil, $C_7H_{15}BrO_4$, M = 243.10 g/mol, yield: 530 mg (53%), **^{1}H-NMR** ($CDCl_3$, 400 MHz) δ 3.88 (m, 1H, –CH_2C*H*CH_2–), 3.81 (t, ^3J(H,H) = 6.2 Hz, 2H, –OCH_2–), 3.78 – 3.53 (m, 8H, –OCH_2–), 3.48 (t, ^3J(H,H) = 6.2 Hz, 2H, BrCH_2–), 2.88 (s, 1H, –O*H*), 2.25 (s, 1H, –O*H*).

2.4.4 12-Bromo-4,7,10-trioxadodecane-1,2-diol (17d)

1,2-*O*-Isopropylidene-4,7,10-trioxadodecane-1,2,12-triol (29d): Synthesized according to P7 from **28** (10.00 g, 35.0 mmol), triethyleneglycol (52.50 g, 0.35 mol) and NaH (4.90 g, 0.12 mol) in DMF (50 mL). Purification by column chromatography (eluent: *n*-hexane/ethylacetate = 1:1). Colourless liquid, $C_{12}H_{24}O_6$, M = 264.16 g/mol, yield: 5.60 g (61%), **^{1}H-NMR** (400 MHz, $CDCl_3$) δ 4.37 – 4.23 (m, 1H, –C*H*–), 4.14 – 4.00 (m, 1H, –CH_2–), 3.84 – 3.45 (m, 15H, –CH_2–), 2.51 (t, ^3J(H,H) = 6.3 Hz, 1H, –O*H*), 1.42 (s, 3H, –CH_3), 1.35 (s, 3H, –CH_3) ppm.

1,2-*O*-Isopropylidene-1,2-dihydroxy-4,7,10-trioxadodec-12-yltoluene-4-sulfonate (30d): Synthesized according to P8 from **29d** (5.60 g, 21.2 mmol) and TosCl (4.50 g, 23.3 mmol) in dry pyridine (50 mL). Purification by column chromatography (eluent: $CHCl_3$/MeOH = 95:5). Colourless liquid, $C_{19}H_{30}O_8S$, M = 418.17 g/mol, yield: 5.05 g (57%), **^{1}H-NMR** (400 MHz, $CDCl_3$) δ 7.83 – 7.76 (m, 2H, Ar–*H*), 7.37 – 7.30 (m, 2H, Ar–*H*), 4.32 – 4.22 (m, 1H, –C*H*–), 4.20 – 4.11 (m, 2H, –CH_2–), 4.11 – 3.98 (m, 1H, –CH_2–), 3.82 – 3.41 (m, 13H, –CH_2–), 2.45 (s, 3H, –CH_3), 1.41 (s, 3H, –CH_3), 1.35 (s, 3H, –CH_3) ppm.

12-Bromo-1,2-*O*-isopropylidene-1,2-dihydroxy-4,7,10-trioxadodecane (16d): Synthesized according to P9 from **30d** (5.05 g, 12.1 mmol) and LiBr (3.15 g, 36.2 mmol) in acetone (150 mL). Purification by column chromatography (eluent: $CHCl_3$/MeOH = 95:5). Colourless liquid, $C_{12}H_{23}BrO_5$, *M* = 326.07 g/mol, yield: 2.57 g (65%), **^{1}H-NMR** (400 MHz, $CDCl_3$) δ 4.33 – 4.23 (m, 1H, –C*H*–), 4.05 (dd, ^3J(H,H) = 8.1 Hz, ^3J(H,H) = 6.5 Hz, 1H, –CH_2–), 3.81 (t, ^3J(H,H) = 6.3 Hz, 2H, –CH_2–), 3.73 (dd, ^3J(H,H) = 10.4 Hz, ^3J(H,H) = 8.6 Hz, 1H, –CH_2–), 3.72 – 3.65 (m, 8H, –CH_2–), 3.58 (dd, ^3J(H,H) = 10.0 Hz, ^3J(H,H) = 5.7 Hz, 1H, –CH_2–), 3.48 – 3.42 (m, 2H, –CH_2–), 1.42 (s, 3H, –CH_3), 1.36 (s, 3H, –CH_3) ppm.

12-Bromo-4,7,10-trioxadodecane-1,2-diol (17d): Synthesized according to P12 from **16d** (1.76 g, 4.4 mmol), HCl (10%, 5 mL) in MeOH (50 mL). Colourless oil, $C_9H_{19}BrO_5$, *M* = 287.15 g/mol, yield: 630 mg (51%), **^{1}H-NMR** ($CDCl_3$, 400 MHz) δ 3.86 (m, 1H, –CH_2C*H*OH), 3.80 (t, ^3J(H,H) = 6.3 Hz, 2H, –CH_2OCH_2–), 3.73 – 3.51 (m, 12H, –OCH_2CH_2O–), 3.47 (t, ^3J(H,H) = 6.3 Hz, 2H, BrCH_2–), 3.24 (s, 1H, –CH_2O*H*), 2.57 (s, 1H, –CH_2CHO*H*).

2.4.5 15-Bromo-4,7,10,13-tetraoxapentadecane-1,2-diol (17e)

1,2-*O*-Isopropylidene-4,7,10,13-tetraoxapentadecane-1,2,15-triol (29e): Synthesized according to P7 from **28** (10.00 g, 35.0 mmol), tetraethyleneglycol (68.0 g, 0.12 mol) and NaH (4.90 g, 0.12 mol) in DMF (50 mL). Purification by column chromatography (eluent: n-hexane/ethylacetate = 1:1). Colourless liquid, $C_{14}H_{28}O_7$, *M* = 308.18 g/mol, yield: 6.40 g (60%), **^{1}H-NMR** (400 MHz, $CDCl_3$) δ 4.36 – 4.22 (m, 1H, –C*H*–), 4.13 – 4.00 (m, 1H, –CH_2–), 3.85 – 3.44 (m, 19H, –CH_2–), 2.53 (t, ^3J(H,H) = 5.7 Hz, 1H, –O*H*), 1.41 (s, 3H, –CH_3), 1.35 (s, 3H, –CH_3) ppm.

1,2-*O*-Isopropylidene-1,2-dihydroxy-4,7,10,13-tetraoxapentadec-15-yltoluene-4-sulfonate (30e): Synthesized according to P8 from **29e** (6.40 g, 20.8 mmol) and TosCl (6.54 g, 22.9 mmol) in dry pyridine (50 mL). Purification by column chromatography (eluent: *n*-hexane/ethylacetate = 1:1). Colourless liquid, $C_{21}H_{34}O_9S$, *M* = 462.19 g/mol, yield: 5.66 g (59%), **^{1}H-NMR** (400 MHz, $CDCl_3$) δ 7.83 – 7.77 (m, 2H, Ar–*H*), 7.38 – 7.31 (m, 2H, Ar–*H*), 4.31 – 4.24 (m, 1H, –C*H*–), 4.18 – 4.13 (m, 2H, –CH_2–), 4.05 (dd, ^3J(H,H) = 8.3 Hz, ^3J(H,H) = 6.4 Hz, 1H, –CH_2–), 3.73 (dd, ^3J(H,H) = 8.3 Hz, ^3J(H,H) = 6.4 Hz, 1H, –CH_2–), 3.71 – 3.54 (m, 15H, –CH_2–), 3.49 (dd, ^3J(H,H) = 10.0 Hz, ^3J(H,H) = 5.5 Hz, 1H, –CH_2–), 2.45 (s, 3H, –CH_3), 1.41 (s, 3H, –CH_3), 1.35 (s, 3H, –CH_3) ppm.

15-Bromo-1,2-*O*-isopropylidene-1,2-dihydroxy-4,7,10,13-tetraoxapentadecane (16e): Synthesized according to P9 from **30e** (5.66 g, 12.3 mmol) and LiBr (3.19 g, 36.8 mmol) in acetone (100 mL). Purification by column chromatography (eluent: $CHCl_3$/MeOH = 95:5). Colourless liquid, $C_{14}H_{27}BrO_6$, *M* = 370.10 g/mol, yield: 3.97 g (87%), **^{1}H-NMR** (500 MHz, $CDCl_3$) δ 4.32 – 4.25 (m, 1H, –C*H*–), 4.06 (dd, ^3J(H,H) = 8.3 Hz, ^3J(H,H) = 6.4 Hz, 1H, –CH_2–), 3.82 (t, ^3J(H,H) = 6.4 Hz, 1H, –CH_2–), 3.74 (dd, ^3J(H,H) = 8.3 Hz, ^3J(H,H) = 6.4 Hz, 1H, –CH_2–), 3.72 – 3.63 (m, 14H, –CH_2–), 3.59 (dd, ^3J(H,H) = 10.0 Hz, ^3J(H,H) = 5.7 Hz, 1H, –CH_2–), 3.54 – 3.45 (m, 2H, –CH_2–), 1.43 (s, 3H, –CH_3), 1.37 (s, 3H, –CH_3) ppm.

2.4.6 18-Bromo-4,7,10,13,16-pentaoxaoctadecane-1,2-diol (17f):

1,2-*O*-Isopropylidene-4,7,10,13,16-pentaoxaoctadecane-1,2,18-triol (29f): Synthesized according to P7 from **16e** (2.36 g, 7.2 mmol), diethyleneglycol (7.10 g, 72.4 mol) and NaH (1.00 g, 25.2 mmol) in DMF (100 mL). Purification by column chromatography (eluent: $CHCl_3$/MeOH = 9:1). Colourless liquid, $C_{16}H_{32}O_8$, *M* = 352.21 g/mol, yield: 1.23 g (50%),

^{1}H-NMR (500 MHz, $CDCl_3$) δ 4.38 – 4.24 (m, 1H, –C*H*–), 4.06 (dd, ^3J(H,H) = 8.3 Hz, ^3J(H,H) = 6.4 Hz, 1H, –CH_2–), 3.81 – 3.61 (m, 21H, –CH_2–), 3.59 (dd, ^3J(H,H) = 10.0 Hz, ^3J(H,H) = 5.7 Hz, 1H, –CH_2–), 3.51 (dd, ^3J(H,H) = 10.0 Hz, ^3J(H,H) = 5.5 Hz, 1H, –CH_2–), 2.63 (t, ^3J(H,H) = 6.2 Hz, 1H, –O*H*), 1.43 (s, 3H, –CH_3), 1.37 (s, 3H, –CH_3) ppm.

1,2-*O*-Isopropylidene-1,2-dihydroxy-4,7,10,13,16-pentaoxaoctadec-18-yltoluene-4-sulfonate (30f): Synthesized according to P8 from **29f** (1.23 g, 3.6 mmol) and TosCl (0.75 g, 3.9 mmol) in dry pyridine (50 mL). Purification by column chromatography (eluent: $CHCl_3$/MeOH = 95:5). Colourless liquid, $C_{23}H_{38}O_{10}S$, M = 506.22 g/mol, yield: 0.67 g (38%), **^{1}H-NMR** (400 MHz, $CDCl_3$) δ 7.83 – 7.77 (m, 2H, Ar–*H*), 7.38 – 7.31 (m, 2H, Ar–*H*), 4.31 – 4.24 (m, 1H, –C*H*–), 4.18 – 4.12 (m, 2H, –CH_2–), 4.05 (dd, ^3J(H,H) = 8.3 Hz, ^3J(H,H) = 6.4 Hz, 1H, –CH_2–), 3.73 (dd, ^3J(H,H) = 8.3 Hz, ^3J(H,H) = 6.4 Hz, 1H, –CH_2–), 3.71 – 3.54 (m, 18H, –CH_2–), 3.49 (dd, ^3J(H,H) = 10.0 Hz, ^3J(H,H) = 5.5 Hz, 1H, –CH_2–), 2.45 (s, 3H, –CH_3), 1.41 (s, 3H, –CH_3), 1.35 (s, 3H, –CH_3) ppm.

18-Bromo-1,2-*O*-isopropylidene-4,7,10,13,16-pentaoxaoctadecane (16f): Synthesized according to P9 from **30f** (0.67 g, 1.3 mmol) and LiBr (0.35 g, 4.0 mmol) in acetone (50 mL). Purification by column chromatography (eluent: $CHCl_3$/MeOH = 95:5). Colourless liquid, $C_{16}H_{31}BrO_7$, M = 414.13 g/mol, yield: 0.45 g (83%), **^{1}H-NMR** (400 MHz, $CDCl_3$) δ 4.32 – 4.24 (m, 1H, –C*H*–), 4.05 (dd, ^3J(H,H) = 8.3 Hz, ^3J(H,H) = 6.4 Hz, 1H, –CH_2–), 3.81 (t, ^3J(H,H) = 6.3 Hz, 2H, –CH_2–), 3.78 – 3.70 (m, 1H, –CH_2–), 3.70 – 3.61 (m, 16H, –CH_2–), 3.58 (dd, ^3J(H,H) = 10.0 Hz, ^3J(H,H) = 5.7 Hz, 1H, –CH_2–), 3.53 – 3.44 (m, 3H, –CH_2–), 1.42 (s, 3H, –CH_3), 1.36 (s, 3H, –CH_3) ppm.

18-Bromo-4,7,10,13,16-pentaoxaoctadecane-1,2-diol (17f): Synthesized according to P12 from **16f** (820 mg, 2.0 mmol), HCl (10%, 5 mL) in MeOH. colourless oil, $C_{13}H_{27}BrO_7$, M = 375.25 g/mol, yield: 420 mg (56%), **^{1}H-NMR** ($CDCl_3$, 400 MHz) δ 3.88 (m, 1H, –CH_2C*H*CH_2–), 3.81 (t, ^3J(H,H) = 6.3 Hz, 2H, –OCH_2–), 3.78 – 3.53 (m, 20H, –OCH_2–), 3.48 (t, ^3J(H,H) = 6.3 Hz, 2H, BrCH_2–), 1.67 (s, 2H, –O*H*) ppm.

2.5 Synthesis of compounds C*m/m*

2.5.1 1,4-Dialkoxy-2,5-bis{4-[4-(2,3-*O*-isopropylidene-2,3-dihydroxypropyl-1-oxy)-phenylethynyl]phenylethynyl}benzenes (8C*m/m*)

1,4-Bis(2-ethylbutyl-1-oxy)-2,5-bis{4-[4-(2,3-*O*-isopropylidene-2,3-dihydroxypropyl-1-oxy)phenylethynyl]phenylethynyl}benzene (8C2/2): Synthesized according to P6 from **7a** (161 mg, 0.30 mmol), **6** (229 mg, 0.69 mmol), [Pd(PPh_3)$_4$] (10.0 mg, 0.009 mmol) and CuI (1.1 mg, 0.006 mmol) in NEt_3 (50 mL). After evaporation of the solvent the residue was purified by column chromatography (eluent: $CHCl_3$). Yellow solid, $C_{62}H_{66}O_8$, M = 938.48 g/mol, mp. 218 °C, yield: 270 mg (96%), **^{1}H-NMR** (500 MHz, $CDCl_3$) δ 7.50 – 7.44 (m, 12H, Ar–*H*), 7.01 (s, 2H, Ar–*H*), 6.93 – 6.88 (m, 4H, Ar–*H*), 4.52–4.47 (m, 2H, –OC*H*–), 4.18 (dd, ^3J(H,H) = 8.5 Hz, ^3J(H,H) = 6.5 Hz, 2H, –OCH_2–), 4.08 (dd, ^3J(H,H) = 9.5 Hz, ^3J(H,H) = 5.4 Hz, 2H, –OCH_2–), 3.97 (dd, ^3J(H,H) = 9.5 Hz, ^3J(H,H) = 5.9 Hz, 2H, –OCH_2–), 3.95 – 3.88 (m, 6H, –OCH_2–), 1.79 – 1.70 (m, 2H, –C*H*–), 1.65 – 1.49 (m, 8H, –CH_2–), 1.47 (s, 6H, –OCH_3), 1.41 (s, 6H, –CH_3), 0.98 (t, ^3J(H,H) = 7.5 Hz, 12H, –CH_3) ppm.

1,4-Bis(2-butylhexyl-1-oxy)-2,5-bis{4-[4-(2,3-*O*-isopropylidene-2,3-dihydroxypropyl-1-oxy)phenylethynyl]phenylethynyl}benzene (8C4/4): Synthesized according to P6 from **7b** (173 mg, 0.27 mmol), **6** (209 mg, 0.63 mmol), [Pd(PPh_3)$_4$] (9.3 mg, 0.008 mmol) and CuI (1.0 mg, 0.006 mmol) in NEt_3 (50 mL). After evaporation of the solvent the residue was purified

by column chromatography (eluent: $CHCl_3$). Yellow solid, $C_{70}H_{82}O_8$, M = 1050.60 g/mol, mp. 165 °C, yield: 280 mg (98%), **^{1}H-NMR** (500 MHz, $CDCl_3$) δ 7.49 – 7.44 (m, 12H, Ar–*H*), 7.00 (s, 2H, Ar–*H*), 6.92 – 6.87 (m, 4H, Ar–*H*), 4.52 – 4.45 (m, 2H, –OC*H*–), 4.18 (dd, ^3J(H,H) = 8.5 Hz, ^3J(H,H) = 6.4 Hz, 2H, –OCH_2–), 4.08 (dd, ^3J(H,H) = 9.5 Hz, ^3J(H,H) = 5.4 Hz, 2H, –OCH_2–), 3.97 (dd, ^3J(H,H) = 9.5 Hz, ^3J(H,H) = 5.9 Hz, 2H, –OCH_2–), 3.95 – 3.88 (m, 6H, –OCH_2–), 1.91 – 1.81 (m, 2H, –C*H*–), 1.62 – 1.16 (m, 36H, –CH_2–, –CH_3), 0.89 (t, ^3J(H,H) = 7.1 Hz, 12H, –CH_3) ppm.

1,4-Bis(2-hexyloctyl-1-oxy)-2,5-bis{4-[4-(2,3-*O*-isopropylidene-2,3-dihydroxypropyl-1-oxy)phenylethynyl]phenylethynyl}benzene (8C6/6): Synthesized according to P6 from **7c** (152 mg, 0.20 mmol), **6** (139 mg, 0.42 mmol), [Pd(PPh_3)$_4$] (7.0 mg, 0.006 mmol), CuI (0.8 mg, 0.004 mmol) in NEt_3 (50 mL). Purification by column chromatography (eluent: $CHCl_3$). Yellow solid, $C_{78}H_{98}O_8$, M = 1163.61 g/mol, mp. 126 °C, yield: 223 mg (95%), ^{1}H NMR (500 MHz, $CDCl_3$) δ 7.52 – 7.43 (m, 12H, Ar–*H*), 7.00 (s, 2H, Ar–*H*), 6.94 – 6.86 (m, 4H, Ar–*H*), 4.53 – 4.45 (m, 2H, –OC*H*–), 4.18 (dd, ^3J(H,H) = 8.5 Hz, ^3J(H,H) = 6.4 Hz, 2H, –OCH_2–), 4.08 (dd, ^3J(H,H) = 9.5 Hz, ^3J(H,H) = 5.4 Hz, 2H, –OCH_2–), 3.97 (dd, ^3J(H,H) = 9.5, ^3J(H,H) = 5.9 Hz, 2H, –OCH_2–), 3.94 – 3.88 (m, 6H, –OCH_2–), 1.91 – 1.80 (m, 2H, –C*H*–), 1.62 – 1.50 (m, 8H, –CH_2–), 1.50 – 1.18 (m, 44H, –CH_2–), 0.86 (t, ^3J(H,H) = 6.9 Hz, 12H, –OCH_3) ppm.

1,4-Bis(2-octyldecyl-1-oxy)-2,5-bis{4-[4-(2,3-*O*-isopropylidene-2,3-dihydroxypropyl-1-oxy)phenylethynyl]phenylethynyl}benzene (8C8/8): Synthesized according to P6 from **7d** (194 mg, 0.22 mmol), **6** (148 mg, 0.45 mmol), [Pd(PPh_3)$_4$] (7.8 mg, 0.007 mmol) and CuI (1.0 mg, 0.005 mmol) in NEt_3 (50 mL). After evaporation of the solvent the residue was purified by column chromatography (eluent: $CHCl_3$). Yellow solid, $C_{86}H_{114}O_8$, M = 1274.85 g/mol, mp. 126 °C, yield: 260 mg (93%), **^{1}H-NMR** (400 MHz, $CDCl_3$) δ 7.60 – 7.36 (m, 12H, Ar–*H*), 7.00 (s, 2H, Ar–*H*), 6.90 (m, 4H, Ar–*H*), 4.52–4.45 (m, 2H, –OC*H*–), 4.25 – 4.13 (m, 2H, –OCH_2–), 4.08 (dd, ^3J(H,H) = 9.6 Hz, ^3J(H,H) = 5.3 Hz, 2H, –OCH_2–), 4.01 – 3.86 (m, 8H, –OCH_2–), 1.84 (m, 2H, –C*H*–), 1.74 – 1.02 (m, 68H, –CH_2–,–CH_3), 0.87 (t, ^3J(H,H) = 6.8 Hz, 12H, –CH_3) ppm.

1,4-Bis(2-decyldodecyl-1-oxy)-2,5-bis{4-[4-(2,3-*O*-isopropylidene-2,3-dihydroxypropyl-1-oxy)phenylethynyl]phenylethynyl}benzene (8C10/10): Synthesized according to P6 from **7e** (175 mg, 0.18 mmol), **6** (125 mg, 0.38 mmol), [Pd(PPh_3)$_4$] (6.2 mg, 0.005 mmol) and CuI (0.7 mg, 0.004 mmol) in NEt_3 (50 mL). After evaporation of the solvent the residue was purified by column chromatography (eluent: $CHCl_3$). Yellow solid, $C_{94}H_{130}O_8$, M = 1386.98 g/mol, mp. 117 °C, yield: 180 mg (73%), **^{1}H-NMR** (400 MHz, $CDCl_3$) δ 7.53 – 7.43 (m, 12H, Ar–*H*), 7.00 (s, 2H, Ar–*H*), 6.95 – 6.88 (m, 4H, Ar–*H*), 4.54 – 4.43 (m, 2H, –OC*H*–), 4.18 (dd, ^3J(H,H) = 8.5 Hz, ^3J(H,H) = 6.4 Hz, 2H, –OCH_2–), 4.08 (dd, ^3J(H,H) = 9.5 Hz, ^3J(H,H) = 5.4 Hz, 2H, –OCH_2–), 3.97 (dd, ^3J(H,H) = 9.6 Hz, ^3J(H,H) = 5.9 Hz, 2H, –OCH_2–), 3.95 – 3.88 (m, 6H), 1.91 – 1.80 (m, 2H, –C*H*–), 1.61 – 1.13 (m, 84H, –CH_2–, –CH_3), 0.87 (t, ^3J(H,H) = 6.9 Hz, 12H, –CH_3) ppm.

1,4-Bis(2-dodecyltetradecyl-1-oxy)-2,5-bis{4-[4-(2,3-*O*-isopropylidene-2,3-dihydroxy-propyl-1-oxy)phenylethynyl]phenylethynyl}benzene (8C12/12): Synthesized according to P6 from **7f** (312 mg, 0.29 mmol), **6** (200 mg, 0.60 mmol), [Pd(PPh_3)$_4$] (10.0 mg, 0.009 mmol) and CuI (1.7 mg, 0.009 mmol) in NEt_3 (50 mL). After evaporation of the solvent the residue was purified by column chromatography (eluent: $CHCl_3$). Yellow solid, $C_{102}H_{146}O_8$, M = 1499.10 g/mol, mp = 116 °C, yield: 380 mg (88%), **^{1}H-NMR** (400 MHz, $CDCl_3$) δ 7.56 – 7.39 (m, 12H, Ar–*H*), 6.99 (s, 2H, Ar–*H*), 6.94 – 6.86 (m, 4H, Ar–*H*), 4.52–4.45 (m, 2H, –OC*H*–), 4.18 (dd, ^3J(H,H) = 8.5 Hz, ^3J(H,H) = 6.5 Hz, 2H, –OCH_2–), 4.08 (dd, ^3J(H,H) = 9.5

Hz, ${}^{3}J$(H,H) = 5.4 Hz, 2H, –OCH_2–), 3.97 (dd, ${}^{3}J$(H,H) = 9.5 Hz, ${}^{3}J$(H,H) = 5.9 Hz, 2H, –OCH_2–), 3.91 (dd, ${}^{3}J$(H,H) = 8.2 Hz, ${}^{3}J$(H,H) = 5.8 Hz, 4H, –OCH_2–), 1.85 (s, 2H, –C*H*–), 1.70 – 1.14 (m, 88H, –CH_2–,–CH_3), 0.86 (t, ${}^{3}J$(H,H) = 6.8 Hz, 12H, –CH_3) ppm.

1,4-Bis(2-tridecylpentadecyl-1-oxy)-2,5-bis{4-[4-(2,3-*O*-isopropylidene-2,3-dihydroxy-propyl-1-oxy)phenylethynyl]phenylethynyl}benzene (8C13/13): Synthesized according to P6 from **7g** (187 mg, 0.18 mmol), **6** (120 mg, 0.36 mmol), [Pd(PPh_3)$_4$] (6.2 mg, 0.005 mmol) and CuI (0.7 mg, 0.004 mmol) in NEt_3 (50 mL). After evaporation of the solvent the residue was purified by column chromatography (eluent: $CHCl_3$). Yellow solid, $C_{106}H_{154}O_8$, M = 1555.16 g/mol, mp. 117 °C, yield: 240 mg (92%), **^{1}H-NMR** (400 MHz, $CDCl_3$) δ 7.51 – 7.43 (m, 12H, Ar–*H*), 7.00 (s, 2H, Ar–*H*), 6.93 – 6.85 (m, 4H, Ar–*H*), 4.53 – 4.45 (m, 2H, –OC*H*–), 4.18 (dd, ${}^{3}J$(H,H) = 8.4 Hz, ${}^{3}J$(H,H) = 6.5 Hz, 2H, –OCH_2–), 4.08 (dd, ${}^{3}J$(H,H) = 9.5 Hz, ${}^{3}J$(H,H) = 5.4 Hz, 2H, –OCH_2–), 3.97 (dd, ${}^{3}J$(H,H) = 9.5 Hz, ${}^{3}J$(H,H) = 5.9 Hz, 2H, –OCH_2–), 3.94 – 3.87 (m, 6H, –OCH_2–), 1.92 – 1.80 (m, 2H, –C*H*–), 1.67 – 1.12 (m, 108H, –CH_2–, –CH_3), 0.87 (t, ${}^{3}J$(H,H) = 6.7 Hz, 12H, –CH_3) ppm.

1,4-Bis(2-tetradecylhexadecyl-1-oxy)-2,5-bis{4-[4-(2,3-*O*-isopropylidene-2,3-dihydroxy-propyl-1-oxy)phenylethynyl]phenylethynyl}benzene (8C14/14): Synthesized according to P6 from **7h** (264 mg, 0.22 mmol), **6** (152 mg, 0.46 mmol), [Pd(PPh_3)$_4$] (7.6 mg, 0.007 mmol) and CuI (0.8 mg, 0.004 mmol) in NEt_3 (50 mL). After evaporation of the solvent the residue was purified by column chromatography (eluent: $CHCl_3$). Yellow solid, $C_{110}H_{162}O_8$, M = 1611.23 g/mol, mp. 117 °C, yield: 340 mg (95%), **^{1}H-NMR** (400 MHz, $CDCl_3$) δ 7.50 – 7.43 (m, 12H, Ar–*H*), 6.99 (s, 2H, Ar–*H*), 6.92–6.88 (m, 4H, Ar–*H*), 4.53 – 4.46 (m, 2H, –OC*H*–), 4.25 – 4.18 (m, 2H, –OCH_2–), 4.08 (dd, ${}^{3}J$(H,H) = 9.5 Hz, ${}^{3}J$(H,H) = 5.4 Hz, 2H, –OCH_2–), 3.97 (dd, ${}^{3}J$(H,H) = 9.6 Hz, ${}^{3}J$(H,H) = 5.9 Hz, 2H, –OCH_2–), 3.94 – 3.87 (m, 6H, –OCH_2–), 1.90 – 1.80 (m, 2H, –C*H*–), 1.64 – 1.12 (m, 116H, –CH_2–, –CH_3), 0.87 (t, ${}^{3}J$(H,H) = 6.8 Hz, 12H, –CH_3) ppm.

1,4-Bis(2-hexadecyloctadecyl-1-oxy)-2,5-bis{4-[4-(2,3-*O*-isopropylidene-2,3-dihydroxy-propyl-1-oxy)phenylethynyl]phenylethynyl}benzene (8C16/16): Synthesized according to P6 from **7i** (217 mg, 0.16 mmol), **6** (127 mg, 0.38 mmol), [Pd(PPh_3)$_4$] (5.5 mg, 0.005 mmol) and CuI (0.6 mg, 0.003 mmol) in NEt_3 (50 mL). After evaporation of the solvent the residue was purified by column chromatography (eluent: $CHCl_3$). Yellow solid, $C_{118}H_{178}O_8$, M = 1723.35 g/mol, mp. 117 °C, yield: 270 mg (98%), **^{1}H-NMR** (500 MHz, $CDCl_3$) δ 7.51 – 7.44 (m, 12H, Ar–*H*), 7.00 (s, 2H, Ar–*H*), 6.92 – 6.87 (m, 4H, Ar–*H*), 4.52 – 4.45 (m, 2H, –OC*H*–), 4.18 (dd, ${}^{3}J$(H,H) = 8.5 Hz, ${}^{3}J$(H,H) = 6.4 Hz, 2H, –OCH_2–), 4.08 (dd, ${}^{3}J$(H,H) = 9.5 Hz, ${}^{3}J$(H,H) = 5.4 Hz, 2H, –OCH_2–), 3.97 (dd, ${}^{3}J$(H,H) = 9.6 Hz, ${}^{3}J$(H,H) = 5.9 Hz, 2H, –OCH_2–), 3.94 – 3.87 (m, 6H, –OCH_2–), 1.90 – 1.81 (m, 2H, –C*H*–), 1.63 – 1.08 (m, 132H, –CH_2–, –CH_3), 0.87 (t, ${}^{3}J$(H,H) = 7.0 Hz, 12H, –CH_3) ppm.

2.5.2 1,4-Dialkoxy-2,5-bis{4-[4-(2,3-dihydroxypropyl-1-oxy)phenylethynyl]phenyl-ethynyl}benzene (C*m*/*m*)

1,4-Bis(2-ethylbutyl-1-oxy)-2,5-bis{4-[4-(2,3-dihydroxypropyl-1-oxy)phenylethynyl]-phenylethynyl}benzene (C2/2): Synthesized according to P11 from **8C2/2** (270 mg, 0.29 mmol) and PPTS (tip of spatula) in MeOH/THF (1:1, 60 mL). Purification by column chromatography (eluent: $CHCl_3$/MeOH = 9:1). Yellow-greenish solid, $C_{56}H_{58}O_8$, M = 858.41 g/mol, yield: 170 mg (68%), **^{1}H-NMR** (500 MHz, pyridine-d_5) δ 7.80 – 7.75 (m, 4H, Ar–*H*), 7.72 – 7.67 (m, 4H, Ar–*H*), 7.66 – 7.62 (m, 4H, Ar–*H*), 7.48 (s, 2H, Ar–*H*), 7.13 – 7.07 (m, 4H, Ar–*H*), 6.95 (d, ${}^{3}J$(H,H) = 4.3 Hz, 2H, –O*H*), 6.54 (t, ${}^{3}J$(H,H) = 5.3 Hz, 2H, –O*H*), 4.61 – 4.54 (m, 2H, –OC*H*–), 4.52 (dd, ${}^{3}J$(H,H) = 9.6 Hz, ${}^{3}J$(H,H) = 4.3 Hz, 2H, –OCH_2–), 4.43 (dd, ${}^{3}J$(H,H) = 9.6 Hz, (H,H) = 6.3 Hz, 2H, –OCH_2–), 4.27 – 4.19 (m, 4H, –OCH_2–), 4.02 (d,

^3J(H,H) = 5.6 Hz, 4H, –OCH_2–), 1.81 – 1.72 (m, 2H, –CH–), 1.72 – 1.61 (m, 4H, –CH_2–), 1.61 – 1.50 (m, 4H, –CH_2–), 0.98 (t, ^3J(H,H) = 7.5 Hz, 12H, –CH_3) ppm. **^{13}C-NMR** (126 MHz, pyridine-d$_5$) δ 160.05 (–OCH$_2$–), 154.29 (–OCH$_2$–), 133.37 (C_{Ar}–H), 131.80, 131.73, 116.98, 115.18, 114.95, 114.25 (C_{Ar}–H), 95.21 (—C≡C—), 92.46 (—C≡C—), 88.87 (—C≡C—), 88.31 (—C≡C—), 71.55 (–OCH$_2$–), 71.11, 70.85, 64.02 (–OCH$_2$–), 41.24 (–CH–), 23.69 (–CH$_2$–), 11.23 (–CH$_3$) ppm. Anal. Calcd. for $C_{56}H_{58}O_8 \cdot H_2O$: C, 76.69; H, 6.90. Found: C, 76.59; H, 6.63.

1,4-Bis(2-butylhexyl-1-oxy)-2,5-bis{4-[4-(2,3-dihydroxypropyl-1-oxy)phenylethynyl]-phenylethynyl}benzene (C4/4): Synthesized according to P11 from **8C4/4** (280 mg, 0.26 mmol) and PPTS (tip of spatula) in MeOH/THF (1:1, 60 mL). Purification by column chromatography (eluent: $CHCl_3$/MeOH = 9:1). Yellow-greenish solid, $C_{64}H_{74}O_8$, M = 970.54 g/mol, yield: 141 mg (56%), **^{1}H-NMR** (400 MHz, pyridine-d$_5$) δ 7.83 – 7.76 (m, 4H, Ar–H), 7.76 – 7.68 (m, 4H, Ar–H), 7.67 – 7.62 (m, 4H, Ar–H), 7.53 (s, 2H, Ar–H), 7.14 – 7.06 (m, 4H, Ar–H), 6.95 (d, ^3J(H,H) = 4.6 Hz, 2H, –OH), 6.54 (t, ^3J(H,H) = 5.0 Hz, 2H, –OH), 4.62 – 4.48 (m, 4H, –OCH–), 4.43 (dd, ^3J(H,H) = 9.5 Hz, ^3J(H,H) = 6.3 Hz, 2H, –OCH_2–), 4.29 – 4.18 (m, 4H, –OCH_2–), 4.06 (d, ^3J(H,H) = 5.4 Hz, 4H, –OCH_2–), 2.00 – 1.88 (m, 2H, –CH–), 1.75 – 1.60 (m, 4H, –CH_2–), 1.60 – 1.27 (m, 20H, –CH_2–), 0.92 (t, (H,H) = 7.0 Hz, 12H, –CH_3) ppm. **^{13}C-NMR** (101 MHz, pyridine-d$_5$) δ 158.86 (–OCH$_2$–), 153.10 (–OCH$_2$–), 132.17 (C_{Ar}–H), 130.59, 130.57, 122.77, 115.78, 113.99, 113.75, 113.02 (C_{Ar}–H), 94.02 (—C≡C—), 91.28 (—C≡C—), 87.70 (—C≡C—), 71.12 (–OCH$_2$–), 69.91, 69.66, 62.83 (–OCH$_2$–), 37.07 (–CH–), 30.15 (–CH$_2$–), 27.99, 21.98, 12.85 (–CH$_3$) ppm. HRMS (m/z): [M]+Li$^+$-calcd. for $C_{64}H_{74}F_8O_8Li$, 977.554; found 977.558. Anal. Calcd. for $C_{64}H_{74}O_8 \cdot H_2O$: C, 77.70; H, 7.74. Found: C, 77.35; H, 7.47.

1,4-Bis(2-hexyloctyl-1-oxy)-2,5-bis{4-[4-(2,3-dihydroxypropyl-1-oxy)-phenylethynyl]-phenylethynyl}benzene (C6/6): Synthesized according to P11 from **8C6/6** (223 mg, 0.19 mmol) and PPTS (tip of a spatula) in MeOH/THF (1:1, 30 mL:30 mL). Purification by column chromatography (eluent: $CHCl_3$:MeOH = 9:1). Yellow-greenish solid, M = 1082.66, yield: 173 mg (83%), ^{1}H NMR (500 MHz, pyridine-d$_5$) δ 7.84 – 7.76 (m, 4H, Ar–H), 7.76 – 7.69 (m, 4H, Ar–H), 7.69 – 7.61 (m, 4H, Ar–H), 7.56 (s, 2H, Ar–H), 7.15 – 7.07 (m, 4H, Ar–H), 6.95 (br, 2H, -OH), 6.53 (br, 2H, -OH), 4.62 – 4.53 (m, 2H, –OCH–), 4.51 (dd, ^3J (H,H)= 9.5 Hz, ^3J (H,H) = 4.3 Hz, 2H, –OCH_2–), 4.43 (dd, ^3J(H,H) = 9.4 Hz, ^3J(H,H) = 6.3 Hz, 2H, –OCH_2–), 4.29 – 4.17 (m, 4H, –OCH_2–), 4.11 (d, ^3J(H,H) = 5.5 Hz, 4H, –OCH_2–), 2.05 – 1.92 (m, 2H, –CH_2–), 1.80 – 1.66 (m, 4H, –CH_2–), 1.63 – 1.53 (m, 4H, –CH_2–), 1.53 – 1.13 (m, 32H, –CH_2–), 0.88 (t, ^3J(H,H) = 7.0 Hz, 12H, –CH_3) ppm. ^{13}C NMR (126 MHz, pyridine-d$_5$) δ 160.06 (–OCH$_2$–), 154.34 (–OCH$_2$–), 133.37 (C_{Ar}–H), 131.81 (C_{Ar}–H), 131.77 (C_{Ar}–H), 117.07 (C_{Ar}–H), 115.19 (C_{Ar}–H), 114.96, 114.29, 95.24 (—C≡C—), 92.46 (—C≡C—), 88.91 (—C≡C—), 88.31 (—C≡C—), 72.36 (–OCH$_2$–), 71.12 (–OCH$_2$–), 70.86 (–OCH$_2$–), 64.03 (–OCH$_2$–), 38.39 (–CH$_2$–), 31.87, 31.71, 29.86, 27.02, 22.74, 14.04 (–CH$_3$) ppm. HRMS (m/z): [M]+Li+-calcd. for $C_{72}H_{90}O_8Li$, 1089.679; found 1089.677. Anal. Calcd. for $C_{72}H_{90}O_8 \cdot H_2O$: C, 78.51; H, 8.42. Found: C, 78.81; H, 8.49.

1,4-Bis(2-octyldecyl-1-oxy)-2,5-bis{4-[4-(2,3-dihydroxypropyl-1-oxy)phenylethynyl]-phenylethynyl}benzene (C8/8): Synthesized according to P11 from **8C8/8** (260 mg, 0.20 mmol) and PPTS (tip of spatula) in MeOH/THF (1:1, 60 mL). Purification by column chromatography (eluent: $CHCl_3$/MeOH = 9:1). Yellow-greenish solid, $C_{80}H_{106}O_8$, M = 1194.79 g/mol, yield: 153 mg (64%), **^{1}H-NMR** (400 MHz, pyridine-d$_5$) δ 7.85 – 7.78 (m, 4H, Ar–H), 7.77 – 7.70 (m, 4H, Ar–H), 7.67 – 7.62 (m, 4H, Ar–H), 7.57 (s, 2H, Ar–H), 7.15 – 7.07 (m, 4H, Ar–H), 4.61 – 4.48 (m, 4H, –OCH–, –OCH_2–), 4.43 (dd, ^3J(H,H) = 9.4 Hz, ^3J(H,H) = 6.4 Hz, 2H, –OCH_2–), 4.28 – 4.19 (m, 4H, –OCH_2–), 4.13 (d, ^3J(H,H) = 5.4 Hz,

4H, –OC*H*$_2$–), 2.08 – 1.96 (m, 2H, –C*H*–), 1.83 – 1.69 (m, 4H, –C*H*$_2$–), 1.68 – 1.16 (m, 56H, –C*H*$_2$–), 0.89 (t, ^3J(H,H) = 6.7 Hz, 12H, –C*H*$_3$) ppm. **^{13}C-NMR** (101 MHz, pyridine-d$_5$) δ 158.85 (–O*C*H$_2$–), 153.15 (–O*C*H$_2$–), 132.16 (*C*$_{Ar}$–H), 130.61, 130.58, 115.85, 113.98, 113.76, 113.09 (*C*$_{Ar}$–H), 94.05 (—C≡C—), 91.25 (—C≡C—), 87.72 (—C≡C—), 87.10 (—C≡C—), 71.19 (–O*C*H$_2$–), 69.92, 69.66, 62.83 (–O*C*H$_2$–), 37.21 (–*C*H–), 30.72 (–*C*H$_2$–), 30.53, 29.04, 28.49, 28.24, 25.89, 21.54, 12.87 (–*C*H$_3$) ppm. HRMS (m/z): [M]+Li$^+$-calcd. for $C_{80}H_{106}O_8Li$, 1201.804; found 1201.804. Anal. Calcd. for $C_{80}H_{106}O_8 \cdot H_2O$: C, 80.36; H, 8.94. Found: C, 79.95; H, 9.00.

1,4-Bis(2-decyldodecyl-1-oxy)-2,5-bis{4-[4-(2,3-dihydroxypropyl-1-oxy)phenylethynyl]-phenylethynyl}benzene (C10/10): Synthesized according to P11 from **8C10/10** (180 mg, 0.13 mmol) and PPTS (tip of spatula) in MeOH/THF (1:1, 60 mL). Purification by column chromatography (eluent: $CHCl_3$/MeOH = 9:1). Yellow-greenish solid, $C_{88}H_{122}O_8$, *M* = 1306.91 g/mol, yield: 108 mg (63%), **^{1}H-NMR** (500 MHz, pyridine-d$_5$) δ 7.84 – 7.80 (m, 4H, Ar–*H*), 7.76 – 7.71 (m, 4H, Ar–*H*), 7.68 – 7.62 (m, 4H, Ar–*H*), 7.57 (s, 2H, Ar–*H*), 7.14 – 7.08 (m, 4H, Ar–*H*), 4.60 – 4.55 (m, 2H, –OC*H*–), 4.52 (dd, ^3J(H,H) = 9.6 Hz, ^3J(H,H) = 4.4 Hz, 2H, –OC*H*$_2$–), 4.43 (dd, ^3J(H,H) = 9.6 Hz, ^3J(H,H) = 6.3 Hz, 2H, –OC*H*$_2$–), 4.27 – 4.19 (m, 4H, –OC*H*$_2$–), 4.14 (d, ^3J(H,H) = 5.5 Hz, 4H, –OC*H*$_2$–), 2.08 – 1.99 (m, 2H, –C*H*–), 1.82 – 1.72 (m, 4H, –C*H*$_2$–), 1.68 – 1.14 (m, 68H, –C*H*$_2$–), 0.89 (t, ^3J(H,H) = 7.0 Hz, 6H, –C*H*$_3$) ppm. **^{13}C-NMR** (126 MHz, pyridine-d$_5$) δ 160.06 (–O*C*H$_2$–), 154.35 (–O*C*H$_2$–), 133.37 (*C*$_{Ar}$–H), 131.82, 131.78, 117.05, 115.18, 114.98, 114.30 (*C*$_{Ar}$–H), 95.25 (—C≡C—), 92.46 (—C≡C—), 88.94 (—C≡C—), 88.31 (—C≡C—), 72.40 (–O*C*H$_2$–), 71.11, 70.87, 64.03 (–O*C*H$_2$–), 38.41 (–*C*H–), 31.93 (–*C*H$_2$–), 31.75, 30.26, 29.81, 29.76, 29.73, 29.43, 27.10, 22.74, 14.07 (–*C*H$_3$) ppm. HRMS (m/z): [M]+Li$^+$-calcd. for $C_{88}H_{122}O_8Li$, 1313.929; found 1313.931. Anal. Calcd. for $C_{88}H_{122}O_8 \cdot H_2O$: C, 79.71; H, 9.43. Found: C, 79.93; H, 9.58.

1,4-Bis(2-dodecyltetradecyl-1-oxy)-2,5-bis{4-[4-(2,3-dihydroxypropyl-1-oxy)phenyl-ethynyl]phenylethynyl}benzene (C12/12): Synthesized according to P11 from **8C12/12** (380 mg, 0.25 mmol) and PPTS (tip of spatula) in MeOH/THF (1:1, 60 mL). Purification by column chromatography (eluent: $CHCl_3$/MeOH = 9:1). Yellow-greenish solid, $C_{96}H_{138}O_8$, *M* = 1419.04 g/mol, yield: 218 mg (61%), **^{1}H-NMR** (400 MHz, pyridine-d$_5$) δ 7.85 – 7.80 (m, 4H, Ar–*H*), 7.77 – 7.72 (m, 4H, Ar–*H*), 7.67 – 7.63 (m, 4H, Ar–*H*), 7.57 (s, 2H, Ar–*H*), 7.14 – 7.08 (m, 4H, Ar–*H*), 4.63 – 4.48 (m, 4H, –OC*H*–, –OC*H*$_2$–), 4.43 (dd, ^3J(H,H) = 9.5 Hz, ^3J(H,H) = 6.3 Hz, 2H, –OC*H*$_2$–), 4.27 – 4.18 (m, 4H, –OC*H*$_2$–), 4.17 – 4.12 (m, 4H, –OC*H*$_2$–), 2.11 – 1.97 (m, 2H, –C*H*–), 1.85 – 1.72 (m, 4H, –C*H*$_2$–), 1.70 – 1.18 (m, 82H, –C*H*$_2$–), 0.88 (t, ^3J(H,H) = 6.8 Hz, 12H, –C*H*$_3$) ppm. **^{13}C-NMR** (126 MHz, pyridine-d$_5$) δ 160.05 (–O*C*H$_2$–), 154.33 (–O*C*H$_2$–), 133.36, 131.81, 131.77, 117.04, 115.16, 114.97, 114.29, 95.24 (—C≡C—), 92.44 (—C≡C—), 88.93 (—C≡C—), 88.30 (—C≡C—), 79.53 (–O*C*H$_2$–), 72.39, 71.11, 70.86, 64.03 (–O*C*H$_2$–), 38.40 (–*C*H–), 31.91 (–*C*H$_2$–), 31.73, 30.25, 29.82, 29.79, 29.76, 29.73, 29.41, 27.09, 22.72, 14.05 (–*C*H$_3$) ppm. HRMS (m/z): [M]+Li$^+$-calcd. for $C_{96}H_{138}O_8Li$, 1426.054; found 1426.054. Anal. Calcd. for $C_{96}H_{138}O_8 \cdot H_2O$: C, 80.18; H, 9.81. Found: C, 80.62; H, 9.52.

1,4-Bis(2-tridecylpentadecyl-1-oxy)-2,5-bis{4-[4-(2,3-dihydroxypropyl-1-oxy)phenyl-ethynyl]phenylethynyl}benzene (C13/13): Synthesized according to P11 from **8C13/13** (240 mg, 0.16 mmol) and PPTS (tip of spatula) in MeOH/THF (1:1, 60 mL). Purification by column chromatography (eluent: $CHCl_3$/MeOH = 9:1). Yellow-greenish solid, $C_{100}H_{146}O_8$, *M* = 1475.10 g/mol, yield: 163 mg (74%), **^{1}H-NMR** (400 MHz, pyridine-d$_5$) δ 7.86 – 7.79 (m, 4H, Ar–*H*), 7.77 – 7.71 (m, 4H, Ar–*H*), 7.70 – 7.63 (m, 4H, Ar–*H*), 7.57 (s, 2H, Ar–*H*), 7.15 – 7.08 (m, 4H, Ar–*H*), 6.94 (d, ^3J(H,H) = 5.1 Hz, 2H, –O*H*), 6.53 (t, ^3J(H,H) = 5.7 Hz, 2H, Ar–*H*), 4.61 – 4.49 (m, 4H, –OC*H*–, –OC*H*$_2$–), 4.43 (dd, ^3J(H,H) = 9.4 Hz, ^3J(H,H) = 6.3 Hz, 2H,

–OCH_2–), 4.28 – 4.19 (m, 4H, –OCH_2–), 4.14 (d, ^3J(H,H) = 5.2 Hz, 4H, –OCH_2–), 2.11 – 1.98 (m, 2H, –C*H*–), 1.87 – 1.71 (m, 4H, –CH_2–), 1.71 – 1.09 (m, 92H, –CH_2–), 0.89 (t, ^3J(H,H) = 6.5 Hz, 12H, –CH_3) ppm. **^{13}C-NMR** (101 MHz, pyridine-d$_5$) δ 158.86 (–OCH_2–), 153.14 (–OCH_2–), 132.17 (C_{Ar}–H), 130.62, 130.59, 115.85, 113.97, 113.78, 113.10 (C_{Ar}–H), 94.05 (—C≡C—), 91.26 (—C≡C—), 87.74 (—C≡C—), 87.11 (—C≡C—), 71.20 (–OCH_2–), 69.92, 69.67, 62.84 (–OCH_2–), 37.20 (–C*H*–), 30.72 (–CH_2–), 30.54, 29.05, 28.61, 28.57, 28.54, 28.22, 25.89, 21.53, 12.86 (–CH_3) ppm. HRMS (m/z): [M]+Li^+-calcd. for $C_{100}H_{146}O_8Li$, 1482.117; found 1482.120. Anal. Calcd. for $C_{100}H_{146}O_8 \cdot H_2O$: C, 80.38; H, 9.98. Found: C, 80.40; H, 9.96.

1,4-Bis(2-tetradecylhexadecyl-1-oxy)-2,5-bis{4-[4-(2,3-dihydroxypropyl-1-oxy)phenyl-ethynyl]phenylethynyl}benzene (C14/14): Synthesized according to P11 from **8C14/14** (340 mg, 0.21 mmol) and PPTS (tip of spatula) in MeOH/THF (1:1, 60 mL). Purification by column chromatography (eluent: $CHCl_3$/MeOH = 9:1). Yellow-greenish solid, $C_{104}H_{154}O_8$, *M* = 1531.16 g/mol, yield: 253 mg (79%), **^{1}H-NMR** (400 MHz, pyridine-d$_5$) δ 7.86 – 7.79 (m, 4H, Ar–*H*), 7.78 – 7.71 (m, 4H, Ar–*H*), 7.69 – 7.62 (m, 4H, Ar–*H*), 7.57 (s, 2H, Ar–*H*), 7.15 – 7.08 (m, 4H, Ar–*H*), 6.95 (d, ^3J(H,H) = 4.1 Hz, 2H, –O*H*), 6.53 (t, ^3J(H,H) = 6.3 Hz, 2H, –O*H*), 4.63 – 4.48 (m, 4H, –OC*H*–,–OCH_2–), 4.43 (dd, ^3J(H,H) = 9.8 Hz, ^3J(H,H) = 6.5 Hz, 2H, –OCH_2–), 4.29 – 4.19 (m, 4H, –OCH_2–), 4.14 (d, ^3J(H,H) = 5.8 Hz, 4H, –OCH_2–), 2.11 – 1.99 (m, 2H, –C*H*–), 1.84 – 1.71 (m, 4H, –CH_2–), 1.71 – 1.17 (m, 100H, –CH_2–), 0.89 (t, ^3J(H,H) = 6.4 Hz, 12H, –CH_3) ppm. **^{13}C-NMR** (101 MHz, pyridine-d$_5$) δ 158.86 (–OCH_2–), 153.14 (–OCH_2–), 132.17 (C_{Ar}–H), 130.62, 130.59, 113.97, 113.78, 113.10 (C_{Ar}–H), 94.05 (—C≡C—), 91.26 (—C≡C—), 87.74 (—C≡C—), 69.92 (–OCH_2–), 69.67, 62.84 (–OCH_2–), 37.20 (–C*H*–), 30.72 (–CH_2–), 30.54, 29.05, 28.62, 28.57, 28.53, 28.21, 25.89, 21.53, 12.86 (–CH_3) ppm. HRMS (m/z): [M]+Li^+-calcd. for $C_{104}H_{154}O_8Li$, 1538.179; found 1538.175. Anal. Calcd. for $C_{104}H_{154}O_8 \cdot H_2O$: C, 80.57; H, 10.14. Found: C, 80.30; H, 10.00.

1,4-Bis(2-hexadecyloctadecyl-1-oxy)-2,5-bis{4-[4-(2,3-dihydroxypropyl-1-oxy)phenyl-ethynyl]phenylethynyl}benzene (C16/16): Synthesized according to P11 from **8C16/16** (270 mg, 0.15 mmol) and PPTS (tip of spatula) in MeOH/THF (1:1, 60 mL). Purification by column chromatography (eluent: $CHCl_3$/MeOH = 9:1). Yellow-greenish solid, $C_{112}H_{170}O_8$, *M* = 1643.29 g/mol, yield: 120 mg (49%), **^{1}H-NMR** (500 MHz, pyridine-d$_5$) δ 7.86 – 7.80 (m, 4H, Ar–*H*), 7.80 – 7.72 (m, 4H, Ar–*H*), 7.70 – 7.64 (m, 4H, Ar–*H*), 7.57 (s, 2H, Ar–*H*), 7.15 – 7.08 (m, 4H, Ar–*H*), 6.95 (d, ^3J(H,H) = 4.8 Hz, 2H, –O*H*), 6.54 (t, ^3J(H,H) = 5.5 Hz, 2H, –O*H*), 4.61 – 4.54 (m, 2H, –OC*H*–), 4.52 (dd, ^2J(H,H) = 9.4 Hz, ^3J(H,H) = 4.2 Hz, 2H, –OCH_2–), 4.43 (dd, ^3J(H,H) = 9.3 Hz, ^3J(H,H) = 6.4 Hz, 2H, –OCH_2–), 4.26 – 4.20 (m, 4H, –OCH_2–), 4.14 (d, ^3J(H,H) = 5.2 Hz, 4H, –OCH_2–), 2.09 – 1.99 (m, 2H, –C*H*–), 1.84 – 1.73 (m, 4H, –CH_2–), 1.71 – 1.13 (m, 116H, –CH_2–), 0.89 (t, ^3J(H,H) = 6.8 Hz, 12H, –CH_3) ppm. **^{13}C-NMR** (126 MHz, pyridine-d$_5$) δ 160.05 (–OCH_2–), 154.33 (–OCH_2–), 133.36 (C_{Ar}–H), 131.81, 131.78, 117.02, 115.16, 114.98, 114.30 (C_{Ar}–H), 95.24 (—C≡C—), 92.39 (—C≡C—), 88.94 (—C≡C—), 88.32 (—C≡C—), 72.38 (–OCH_2–), 71.11, 70.86, 64.03 (–OCH_2–), 38.39 (–C*H*–), 31.90 (–CH_2–), 31.72, 30.24, 29.81, 29.79, 29.75, 29.71, 29.40, 27.07, 22.71, 14.05 (–CH_3) ppm. HRMS (m/z): [M]+Li^+-calcd. for $C_{112}H_{170}O_8Li$, 1650.305; found 1650.308. Anal. Calcd. for $C_{112}H_{170}O_8 \cdot H_2O$: C, 80.91; H, 10.43. Found: C, 80.98; H, 10.72.

2.6 Synthesis of compounds C$_x$*m/m*

2.6.1 1,4-Bis(dodecyltetradecyl-1-oxy)-2,5-bis[4-(4-hydroxyphenylethynyl)phenyl-ethynyl]benzene (15f)

1,4–Bis(dodecyltetradecyl-1-oxy)-2,5-bis{4-[4-(triisopropylsilyloxy)phenylethynyl]-phenylethynyl}benzene (14f): Synthesized according to P6 from **7f** (0.8 g, 0.7 mmol), **13** (0.51 g, 1.5 mmol), [Pd(PPh$_3$)$_4$] (25 mg, 0.02 mmol) and CuI (2.8 mg, 0.01 mmol) in NEt$_3$ (50 mL). After evaporation of the solvent the residue was purified by column chromatography (eluent: CHCl$_3$). Yellow solid, $C_{108}H_{166}O_4Si_2$, *M* = 1583.23 g/mol, mp. 84 °C, yield: 1.08 g (96%), **^{1}H-NMR** (400 MHz, CDCl$_3$) δ 7.53 – 7.42 (m, 8H, Ar–*H*), 7.43 – 7.34 (m, 4H, Ar–*H*), 7.00 (s, 2H, Ar–*H*), 6.93 – 6.80 (m, 4H, Ar–*H*), 3.90 (d, ^3J(H,H) = 5.5 Hz, 4H, –OC*H*$_2$–), 1.93 – 1.77 (m, 2H, –C*H*–), 1.67 – 1.02 (m, 122H, –C*H*–, –C*H*$_2$–, –CH(CH$_3$)$_2$), 0.87 (t, ^3J(H,H) = 6.7 Hz, 12H, –C*H*$_3$) ppm.

1,4-Bis(dodecyltetradecyl-1-oxy)-2,5-bis[4-(4-hydroxyphenylethynyl)phenylethynyl]-benzene (15f): Synthesized according to P10 from **14d** (1.08 g, 0.7 mmol) and Bu$_4$NF (0.42 g, 1.6 mmol) in THF (50 mL). After evaporation of the solvent the residue was purified by column chromatography (eluent: CHCl$_3$). Yellow solid, $C_{90}H_{126}O_4$, *M* = 1270.97 g/mol, mp. 118 °C, yield: 0.45 g (53%), **^{1}H-NMR** (400 MHz, CDCl$_3$) δ 7.53 – 7.35 (m, 12H, Ar–*H*), 7.00 (s, 2H, Ar–*H*), 6.89 – 6.72 (m, 4H, Ar–*H*), 4.82 (s, 2H, –O*H*), 3.91 (d, ^3J(H,H) = 5.4 Hz, 4H, –OC*H*$_2$–), 1.84 (m, 2H, –C*H*–), 1.66 – 1.11 (m, 88H, –C*H*$_2$–), 0.87 (t, ^3J(H,H) = 6.7 Hz, 12H, –C*H*$_3$) ppm.

2.6.2 1,4-Bis(2-dodecyltetradecyl-1-oxy)-2,5-bis(4-subst.-phenylethynyl]phenylethynyl}-benzenes (8C$_x$12/12)

1,4-Bis(2-dodecyltetradecyl-1-oxy)-2,5-bis{4-[4-(5,6-*O*-isopropylidene-5,6-dihydroxy-3-oxahexyl-1-oxy)phenylethynyl]phenylethynyl}benzene (8C$_1$12/12): Synthesized according to P5 from **15f** (60 mg, 0.05 mmol), **16b** (24 mg, 0.10 mmol), K$_2$CO$_3$ (36 mg, 0.26 mmol) and Bu$_4$NI (tip of spatula) in DMF (20 mL). Purification by column chromatography (eluent: CHCl$_3$). Yellow solid, $C_{106}H_{154}O_{10}$, *M* = 1587.15 g/mol, mp. 94 °C, yield: 70 mg (92%), **^{1}H-NMR** (400 MHz, CDCl$_3$) δ 7.53 – 7.41 (m, 12H, Ar–*H*), 7.00 (s, 2H, Ar–*H*), 6.95 – 6.88 (m, 4H, Ar–*H*), 4.36 – 4.27 (m, 2H, –OC*H*–), 4.15 (t, ^3J(H,H) = 4.8 Hz, 4H, –OC*H*$_2$–), 4.07 (dd, ^3J(H,H) = 8.2 Hz, ^3J(H,H) = 6.5 Hz, 2H, –OC*H*$_2$–), 3.98 – 3.88 (m, 8H, –OC*H*$_2$–), 3.76 (dd, ^3J(H,H) = 8.2 Hz, ^3J(H,H) = 6.4 Hz, 2H, –OC*H*$_2$–), 3.66 (dd, ^3J(H,H) = 10.0 Hz, ^3J(H,H) = 5.7 Hz, 2 H, –OC*H*$_2$–), 3.59 (dd, ^3J(H,H) = 10.0 Hz, ^3J(H,H) = 5.4 Hz, 2H, –OC*H*$_2$–), 1.93 – 1.79 (m, 2H, –C*H*–), 1.72 – 1.04 (m, 98H, –C*H*$_2$–, –C*H*$_3$), 0.86 (t, ^3J(H,H) = 6.7 Hz, 12H, –C*H*$_3$) ppm.

1,4-Bis(2-dodecyltetradecyl-1-oxy)-2,5-bis{4-[4-(8,9-*O*-isopropylidene-8,9-dihydroxy-3,6-dioxanonyl-1-oxy)phenylethynyl]phenylethynyl}benzene (8C$_2$12/12): Synthesized according to P5 from **15f** (60 mg, 0.05 mmol), **16c** (29 mg, 0.10 mmol), K$_2$CO$_3$ (35 mg, 0.25 mmol) and Bu$_4$NI (tip of spatula) in DMF (20 mL). Purification by column chromatography (eluent: CHCl$_3$). Yellow solid, $C_{110}H_{162}O_{12}$, *M* = 1675.21 g/mol, mp. 84 °C, yield: 50 mg (52%), **^{1}H-NMR** (400 MHz, CDCl$_3$) δ 7.50 – 7.42 (m, 12H, Ar–*H*), 7.00 (s, 2H, Ar–*H*), 6.93 – 6.86 (m, 4H, Ar–*H*), 4.33 – 4.24 (m, 2H, –OC*H*–), 4.19 – 4.12 (m, 4H, –OC*H*$_2$–), 4.05 (dd, ^3J(H,H) = 8.2 Hz, ^3J(H,H) = 6.4 Hz, 2H, –OC*H*$_2$–), 3.93 – 3.83 (m, 8H, –OC*H*$_2$–), 3.79 – 3.63 (m, 10H, –OC*H*$_2$–), 3.60 (dd, ^3J(H,H) = 10.1 Hz, ^3J(H,H) = 5.7 Hz, 2H, –OC*H*$_2$–), 3.52 (dd, ^3J(H,H) = 10.0 Hz, ^3J(H,H) = 5.5 Hz, 2H, –OC*H*$_2$–), 1.91 – 1.80 (m, 2H, –C*H*–), 1.63 – 1.15 (m, 100H, –C*H*$_2$–,–C*H*$_3$), 0.86 (t, ^3J(H,H) = 6.8 Hz, 12H, –C*H*$_3$) ppm.

1,4-Bis(2-dodecyltetradecyl-1-oxy)-2,5-bis{4-[4-(11,12-*O*-isopropylidene-11,12-dihydroxy-3,6,9-trioxadodecyl-1-oxy)phenylethynyl]phenylethynyl}benzene (8$C_3$12/12): Synthesized according to P5 from **15f** (60 mg, 0.05 mmol), **16d** (34 mg, 0.10 mmol), K_2CO_3 (35 mg, 0.25 mmol) and Bu_4NI (tip of spatula) in DMF (20 mL). Purification by column chromatography (eluent: $CHCl_3$). Yellow solid, $C_{114}H_{170}O_{14}$, M = 1763.26 g/mol, mp. 90 °C, yield: 80 mg (93%), **^{1}H-NMR** (400 MHz, $CDCl_3$) δ 7.52 – 7.44 (m, 12H, Ar–*H*), 6.99 (s, 2H, Ar–*H*), 6.95 – 6.88 (m, 4H, Ar–*H*), 4.34 – 4.23 (m, 2H, –OC*H*–), 4.19 – 4.12 (m, 4H, –OCH_2–), 4.05 (dd, ^3J(H,H) = 8.2 Hz, ^3J(H,H) = 6.5 Hz, 2H, –OCH_2–), 3.95 – 3.84 (m, 8H, –OCH_2–), 3.80 – 3.63 (m, 18H, –OCH_2–), 3.58 (dd, ^3J(H,H) = 10.0 Hz, ^3J(H,H) = 5.7 Hz, 2H, –OCH_2–), 3.50 (dd, ^3J(H,H) = 10.0 Hz, ^3J(H,H) = 5.5 Hz, 2H, –OCH_2–), 1.90 – 1.78 (m, 2H, –C*H*–), 1.61 – 1.05 (m, 100H, –CH_2–, –CH_3), 0.87 (t, ^3J(H,H) = 6.8 Hz, 12H, –CH_3). ppm.

1,4-Bis(2-dodecyltetradecyl-1-oxy)-2,5-bis{4-[4-(14,15-*O*-isopropylidene-14,15-dihydroxy-3,6,9,12-tetraoxapentadecyl-1-oxy)phenylethynyl]phenylethynyl}benzene (8$C_4$12/12): Synthesized according to P5 from **15f** (60 mg, 0.05 mmol), **16e** (38 mg, 0.10 mmol), K_2CO_3 (34 mg, 0.25 mmol) and Bu_4NI (tip of spatula) in DMF (20 mL). Purification by column chromatography (eluent: $CHCl_3$). Yellow solid, $C_{118}H_{178}O_{16}$, M = 1851.31 g/mol, mp. 82 °C, yield: 80 mg (91%) **^{1}H-NMR** (400 MHz, $CDCl_3$) δ 7.50 – 7.42 (m, 12H, Ar–*H*), 7.00 (s, 2H, Ar–*H*), 6.93 – 6.86 (m, 4H, Ar–*H*), 4.32 – 4.23 (m, 2H, –OC*H*–), 4.18 – 4.12 (m, 4H, –OCH_2–), 4.05 (dd, ^3J(H,H) = 8.2 Hz, ^3J(H,H) = 6.5 Hz, 2H, –OCH_2–), 3.94 – 3.83 (m, 8H, –OCH_2–), 3.78 – 3.62 (m, 26H, –OCH_2–), 3.58 (dd, ^3J(H,H) = 10.0 Hz, ^3J(H,H) = 5.7 Hz, 2H, –OCH_2–), 3.49 (dd, ^3J(H,H) = 10.0 Hz, ^3J(H,H) = 5.5 Hz, 2H, –OCH_2–), 1.91 – 1.79 (m, 2H, –C*H*–), 1.63 – 1.13 (m, 100H, –CH_2–, –CH_3), 0.86 (t, ^3J(H,H) = 6.8 Hz, 12H, –CH_3) ppm.

1,4-Bis(2-dodecyltetradecyl-1-oxy)-2,5-bis{4-[4-(17,18-*O*-isopropylidene-17,18-dihydroxy-3,6,9,12,15-pentaoxaoctadecyl-1-oxy)phenylethynyl]phenylethynyl}benzene (8$C_5$12/12): Synthesized according to P5 from **15f** (60 mg, 0.05 mmol), **16f** (43 mg, 0.10 mmol), K_2CO_3 (35 mg, 0.25 mmol) and Bu_4NI (tip of spatula) in DMF (20 mL). Purification by column chromatography (eluent: $CHCl_3$). Yellow solid, $C_{122}H_{186}O_{18}$, M = 1939.36 g/mol, mp. 94 °C, yield: 80 mg (86%), **^{1}H-NMR** (400 MHz, $CDCl_3$) δ 7.53 – 7.42 (m, 12H, Ar–*H*), 7.00 (s, 2H, Ar–*H*), 6.95 – 6.86 (m, 4H, Ar–*H*), 4.32 – 4.24 (m, 2H, –OC*H*–), 4.19 – 4.12 (m, 4H, –OCH_2–), 4.05 (dd, ^3J(H,H) = 8.3 Hz, ^3J(H,H) = 6.4 Hz, 2H, –OCH_2–), 3.94 – 3.83 (m, 8H, –OCH_2–), 3.79 – 3.61 (m, 34H, –OCH_2–), 3.58 (dd, ^3J(H,H) = 10.0 Hz, ^3J(H,H) = 5.7 Hz, 2H, –OCH_2–), 3.49 (dd, ^3J(H,H) = 10.0 Hz, ^3J(H,H) = 5.6 Hz, 2H, –OCH_2–), 1.91 – 1.80 (m, 2H, –C*H*–), 1.64 – 1.14 (m, 100H, –CH_2–, –CH_3), 0.86 (t, ^3J(H,H) = 6.9 Hz, 12H, –CH_3) ppm.

2.6.3 1,4-Bis(octadecyleicosyl-1-oxy)-2,5-bis[4-(4-hydroxyphenylethynyl)phenylethynyl]-benzene (15h)

1,4–Bis(octadecyleicosyl-1-oxy)-2,5-bis{4-[4-(triisopropylsilyloxy)phenylethynyl]phenyl-ethynyl}benzene (14j): Synthesized according to P6 from **7j** (1.00 g, 0.7 mmol), **13** (0.53 g, 1.5 mmol), [Pd(PPh_3)$_4$] (25 mg, 0.02 mmol) and CuI (2.7 mg, 0.01 mmol) in NEt_3 (50 mL). After evaporation of the solvent the residue was purified by column chromatography (eluent: $CHCl_3$). Yellow solid, $C_{132}H_{214}O_4Si_2$, M = 1919.61 g/mol, mp. 84 °C, yield: 1.30 g (98%), **^{1}H-NMR** (400 MHz, $CDCl_3$) δ 7.51 – 7.44 (m, 8H, Ar–*H*), 7.43 – 7.37 (m, 4H, Ar–*H*), 6.99 (s, 2H, Ar–*H*), 6.90 – 6.81 (m, 4H, Ar–*H*), 3.90 (d, ^3J(H,H) = 5.5 Hz, 4H, –OCH_2–), 1.90 – 1.80 (m, 2H, –C*H*–), 1.64 – 1.18 (m, 142H, –C*H*–, –CH_2–), 1.11 (d, ^3J(H,H) = 7.3 Hz, 36H, –CH(CH_3)$_2$), 0.87 (t, ^3J(H,H) = 6.8 Hz, 12H, –CH_3) ppm.

1,4-Bis(octadecyleicosyl-1-oxy)-2,5-bis[4-(4-hydroxyphenylethynyl)phenylethynyl]-benzene (15j): Synthesized according to P10 from **14h** (1.30 g, 0.7 mmol) and Bu_4NF (0.42 g, 1.6 mmol) in THF (50 mL). After evaporation of the solvent the residue was purified by column chromatography (eluent: $CHCl_3$). Yellow solid, $C_{114}H_{174}O_4$, M = 1607.34 g/mol, mp 85 °C, yield: 0.42 g (38%), **^{1}H-NMR** (400 MHz, $CDCl_3$) δ 7.53 – 7.38 (m, 12H, Ar–*H*), 7.00 (s, 2H, Ar–*H*), 6.85 – 6.77 (m, 4H, Ar–*H*), 4.85 (s, 2H, –O*H*), 3.90 (d, ^3J(H,H) = 5.4 Hz, 4H, –OC*H*$_2$–), 1.93 – 1.77 (m, 2H, –C*H*–), 1.64 – 1.11 (m, 136H, –C*H*$_2$–), 0.87 (t, ^3J(H,H) = 6.7 Hz, 12H, –C*H*$_3$) ppm.

2.6.4 1,4-Bis(2-octadecyleicosyl-1-oxy)-2,5-bis(4-subst.-phenyl-ethynyl]phenylethynyl}-benzenes (8C$_x$18/18)

1,4-Bis(2-octadecyleicosyl-1-oxy)-2,5-bis{4-[4-(5,6-*O*-isopropylidene-5,6-dihydroxy-3-oxahexyl-1-oxy)phenylethynyl]phenylethynyl}benzene (8C$_1$18/18): Synthesized according to P5 from **15j** (80 mg, 0.05 mmol), **16b** (26 mg, 0.11 mmol), K_2CO_3 (35 mg, 0.25 mmol) and Bu_4NI (tip of spatula) in DMF (20 mL). Purification by column chromatography (eluent: $CHCl_3$). Yellow solid, $C_{130}H_{202}O_{10}$, M = 1923.53 g/mol, mp. 99 °C, yield: 90 mg (94%), **^{1}H-NMR** (400 MHz, $CDCl_3$) δ 7.56 – 7.41 (m, 12H, Ar–*H*), 6.99 (s, 2H, Ar–*H*), 6.89 (m, 4H, Ar–*H*), 4.37 – 4.26 (m, 2H, –OC*H*–), 4.15 (t, ^3J(H,H) = 4.8 Hz, 4H, –OC*H*$_2$–), 4.07 (dd, ^3J(H,H) = 8.1 Hz, ^3J(H,H) = 6.6 Hz, 2H, –OC*H*$_2$–), 3.95 – 3.83 (m, 8H, –OC*H*$_2$–), 3.76 (dd, ^3J(H,H) = 8.2 Hz, ^3J(H,H) = 6.4 Hz, 2H, –OC*H*$_2$–), 3.66 (dd, ^3J(H,H) = 10.0 Hz, ^3J(H,H) = 5.5 Hz, 2H, –OC*H*$_2$–) 3.59 (dd, ^3J(H,H) = 10.0 Hz, ^3J(H,H) = 5.4 Hz, 2H, –OC*H*$_2$–), 1.94 – 1.80 (m, 2H, –C*H*–), 1.68 – 1.14 (m, 148H, –C*H*$_2$–, –C*H*$_3$), 0.87 (t, ^3J(H,H) = 6.7 Hz, 12H, –C*H*$_3$) ppm.

1,4-Di(2-octadecyleicosyl-1-oxy)-2,5-bis{4-[4-(8,9-*O*-isopropylidene-8,9-dihydroxy-3,6-dioxanonyl-1-oxy)phenylethynyl]phenylethynyl}benzene (8C$_2$18/18): Synthesized according to P5 from **15j** (80 mg, 0.05 mmol), **16c** (30 mg, 0.11 mmol), K_2CO_3 (36 mg, 0.25 mmol) and Bu_4NI (tip of spatula) in DMF (20 mL). Purification by column chromatography (eluent: $CHCl_3$). Yellow solid, $C_{134}H_{210}O_{12}$, M = 2011.58 g/mol, mp. 93 °C, yield: 100 mg (96%), **^{1}H-NMR** (400 MHz, $CDCl_3$) δ 7.51 – 7.41 (m, 12H, Ar–*H*), 7.00 (s, 2H, Ar–*H*), 6.93 – 6.86 (m, 4H, Ar–*H*), 4.34 – 4.25 (m, 2H, –OC*H*–), 4.19 – 4.13 (m, 4H, –OC*H*$_2$–), 4.05 (dd, ^3J(H,H) = 8.3 Hz, ^3J(H,H) = 6.4 Hz, 2H, –OC*H*$_2$–), 3.95 – 3.84 (m, 8H, –OC*H*$_2$–), 3.78 – 3.66 (m, 10H, –OC*H*$_2$–), 3.60 (dd, ^3J(H,H) = 10.0 Hz, ^3J(H,H) = 5.7 Hz, 2H, –OC*H*$_2$–), 3.52 (dd, ^3J(H,H) = 10.1 Hz, ^3J(H,H) = 5.4 Hz, 2H, –OC*H*$_2$–), 1.91 – 1.79 (m, 2H, –C*H*–), 1.63 – 1.15 (m, 148H, –C*H*$_2$–, –C*H*$_3$), 0.87 (t, ^3J(H,H) = 6.9 Hz, 12H, –C*H*$_3$) ppm.

1,4-Bis(2-octadecyleicosyl-1-oxy)-2,5-bis{4-[4-(11,12-*O*-isopropylidene-11,12-dihydroxy-3,6,9-trioxadodecyl-1-oxy)phenylethynyl]phenylethynyl}benzene (8C$_3$18/18): Synthesized according to P5 from **15j** (80 mg, 0.05 mmol), **16d** (36 mg, 0.11 mmol), K_2CO_3 (36 mg, 0.26 mmol) and Bu_4NI (tip of spatula) in DMF (20 mL). Purification by column chromatography (eluent: $CHCl_3$). Yellow solid, $C_{138}H_{218}O_{14}$, M = 2099.63 g/mol, mp. 92 °C, yield: 90 mg (88%), **^{1}H-NMR** (400 MHz, $CDCl_3$) δ 7.53 – 7.43 (m, 12H, Ar–*H*), 6.99 (s, 2H, Ar–*H*), 6.94 – 6.88 (m, 4H, Ar–*H*), 4.30 – 4.25 (m, 2H, –OC*H*–), 4.19 – 4.12 (m, 4H, –OC*H*$_2$–), 4.05 (dd, ^3J(H,H) = 8.2 Hz, ^3J(H,H) = 6.4 Hz, 2H, –OC*H*$_2$–), 3.94 – 3.85 (m, 8H, –OC*H*$_2$–), 3.79 – 3.63 (m, 18H, –OC*H*$_2$–), 3.58 (dd, ^3J(H,H) = 10.0 Hz, ^3J(H,H) = 5.6 Hz, 2H, –OC*H*$_2$–), 3.50 (dd, ^3J(H,H) = 10.0 Hz, ^3J(H,H) = 5.5 Hz, 2H, –OC*H*$_2$–), 1.92 – 1.78 (m, 2H, –C*H*–), 1.62 – 1.12 (m, 148H, –C*H*$_2$–, –C*H*$_3$), 0.87 (t, ^3J(H,H) = 6.8 Hz, 12H, –C*H*$_3$) ppm.

1,4-Bis(2-octadecyleicosyl-1-oxy)-2,5-bis{4-[4-(14,15-*O*-isopropylidene-14,15-dihydroxy-3,6,9,12-tetraoxapentadecyl-1-oxy)phenylethynyl]phenylethynyl}benzene ($8C_418/18$): Synthesized according to P5 from **15j** (80 mg, 0.05 mmol), **16e** (40 mg, 0.11 mmol), K_2CO_3 (35 mg, 0.25 mmol) and Bu_4NI (tip of spatula) in DMF (20 mL). Purification by column chromatography (eluent: $CHCl_3$). Yellow solid, $C_{142}H_{226}O_{16}$, M = 2187.69 g/mol, mp. 76 °C, yield: 100 mg (91%), **^{1}H-NMR** (500 MHz, $CDCl_3$) δ 7.53 – 7.43 (m, 12H, Ar–*H*), 7.01 (s, 2H, Ar–*H*), 6.95 – 6.88 (m, 4H, Ar–*H*), 4.34 – 4.25 (m, 2H, –OC*H*–), 4.19 – 4.15 (m, 4H, –OCH_2–), 4.06 (dd, ^3J(H,H) = 8.3 Hz, ^3J(H,H) = 6.4 Hz, 2H, –OCH_2–), 3.94 – 3.84 (m, 8H, –OCH_2–), 3.80 – 3.62 (m, 26H, –OCH_2–), 3.59 (dd, ^3J(H,H) = 10.0 Hz, ^3J(H,H) = 5.7 Hz, 2H, –OCH_2–), 3.51 (dd, ^3J(H,H) = 10.0 Hz, ^3J(H,H) = 5.5 Hz, 2H, –OCH_2–), 1.91 – 1.81 (m, 2H, –C*H*–), 1.61 – 1.11 (m, 148H, –CH_2–, –CH_3), 0.89 (t, ^3J(H,H) = 7.0 Hz, 12H, –CH_3) ppm.

1,4-Bis(2-octadecyleicosyl-1-oxy)-2,5-bis{4-[4-(17,18-*O*-isopropylidene-17,18-dihydroxy-3,6,9,12,15-pentaoxaoctadecyl-1-oxy)phenylethynyl]phenylethynyl}benzene ($8C_518/18$): Synthesized according to P5 from **15j** (80 mg, 0.05 mmol), **16f** (45 mg, 0.11 mmol), K_2CO_3 (35 mg, 0.25 mmol) and Bu_4NI (tip of spatula) in DMF (20 mL). Purification by column chromatography (eluent: $CHCl_3$). Yellow solid, $C_{146}H_{234}O_{18}$, M = 2275.74 g/mol, mp. 78 °C, yield: 110 mg (96%), **^{1}H-NMR** (400 MHz, $CDCl_3$) δ 7.51 – 7.42 (m, 12H, Ar–*H*), 6.99 (s, 2H, Ar–*H*), 6.95 – 6.85 (m, 4H, Ar–*H*), 4.32 – 4.24 (m, 2H, –OC*H*–), 4.18 – 4.13 (m, 4H, –OCH_2–), 4.05 (dd, ^3J(H,H) = 8.2 Hz, ^3J(H,H) = 6.4 Hz, 2H, –OCH_2–), 3.93 – 3.81 (m, 8H, –OCH_2–), 3.78 – 3.61 (m, 34H, –OCH_2–), 3.58 (dd, ^3J(H,H) = 10.0 Hz, ^3J(H,H) = 5.7 Hz, 2H, –OCH_2–), 3.49 (dd, ^2J(H,H) = 10.0 Hz, ^3J(H,H) = 5.6 Hz, 2H, –OCH_2–), 1.91 – 1.80 (m, 2H, –C*H*–), 1.63 – 1.13 (m, 148H, –CH_2–, –CH_3), 0.87 (t, ^3J(H,H) = 6.9 Hz, 12H, –CH_3) ppm.

2.6.5 Compounds $C_x12/12$

1,4-Bis(2-dodecyltetradecyl-1-oxy)-2,5-bis{4-[4-(5,6-dihydroxy-3-oxahexyl-1-oxy)phenyl-ethynyl]phenylethynyl}benzene ($C_112/12$): Synthesized according to P11 from **$8C_112/12$** (70 mg, 0.05 mmol) and PPTS (tip of spatula) in MeOH/THF (1:1, 60 mL). Purification by column chromatography (eluent: $CHCl_3$/MeOH = 9:1). Yellow-greenish solid, $C_{100}H_{154}O_{10}$, M = 1507.09 g/mol, yield: 26 mg (36%), **^{1}H-NMR** (500 MHz, pyridine-d_5) δ 7.85 – 7.81 (m, 4H, Ar–*H*), 7.77 – 7.72 (m, 4H, Ar–*H*), 7.69 – 7.62 (m, 4H, Ar–*H*), 7.57 (s, 2H, Ar–*H*), 7.09 – 7.02 (m, 4H, Ar–*H*), 6.52 (br, 2H, –O*H*), 6.27 (br, 2H, –O*H*), 4.44 – 4.38 (m, 2H, –OC*H*–), 4.21 – 4.09 (m, 12H, –OCH_2–), 4.02 (dd, ^3J(H,H) = 9.7 Hz, ^3J(H,H) = 4.8 Hz, 2H, –OCH_2–), 3.97 – 3.91 (m, 6H, –OCH_2–), 2.09 – 1.99 (m, 2H, –C*H*–), 1.83 – 1.72 (m, 4H, –CH_2–), 1.69 – 1.16 (m, 80H, –CH_2–), 0.88 (t, ^3J(H,H) = 7.0 Hz, 12H, –CH_3) ppm. **^{13}C-NMR** (126 MHz, pyridine-d_5) δ 159.69 (–OCH$_2$–), 154.35 (–OCH$_2$–), 133.39 (C_{Ar}), 131.83, 131.80, 117.06, 115.14, 114.30 (C_{Ar}), 95.25 (—C≡C—), 92.38 (—C≡C—), 88.96 (—C≡C—), 88.38 (—C≡C—), 74.10 (–OCH$_2$–), 72.40, 71.81, 69.82, 67.86, 64.40 (–OCH$_2$–), 38.41 (–C*H*–), 31.93 (–CH_2–), 31.74, 30.26, 29.83, 29.80, 29.77, 29.74, 29.42, 27.10, 22.73, 14.07 (–CH$_3$) ppm.

1,4-Bis(2-dodecyltetradecyl-1-oxy)-2,5-bis{4-[4-(8,9-dihydroxy-3,6-dioxanonyl-1-oxy)-phenylethynyl]phenylethynyl}benzene ($C_212/12$): Synthesized according to P11 from **$8C_212/12$** (50 mg, 0.03 mmol) and PPTS (tip of spatula) in MeOH/THF (1:1, 60 mL). Purification by column chromatography (eluent: $CHCl_3$/MeOH = 9:1). Yellow-greenish solid, $C_{104}H_{154}O_{12}$, M = 1595.14 g/mol, yield: 22 mg (46%), **^{1}H-NMR** (500 MHz, pyridine-d_5) δ 7.85 – 7.80 (m, 4H, Ar–*H*), 7.76 – 7.72 (m, 4H, Ar–*H*), 7.69 – 7.63 (m, 4H, Ar–*H*), 7.57 (s, 2H, Ar–*H*), 7.09 – 7.04 (m, 4H, Ar–*H*), 6.39 (br, 2H, –O*H*), 6.20 (br, 2H, –O*H*), 4.42 – 4.35 (m, 2H, –OC*H*–), 4.19 – 4.07 (m, 12H, –OCH_2–), 3.97 (dd, ^3J(H,H) = 9.8 Hz, ^3J(H,H) = 4.9 Hz, 2H, –OCH_2–), 3.90 (dd, ^3J(H,H) = 9.8 Hz, ^3J(H,H) = 6.2 Hz, 2H, –OCH_2–), 3.87 – 3.83

(m, 4H, –OCH_2–), 3.79 – 3.71 (m, 8H, –OCH_2–), 2.08 – 1.99 (m, 2H, –CH–), 1.83 – 1.73 (m, 4H, –CH_2–), 1.69 – 1.19 (m, 80H, –CH_2–), 0.89 (t, ^3J(H,H) = 7.0 Hz, 12H, –CH_3) ppm. **^{13}C-NMR** (126 MHz, pyridine-d$_5$) δ 159.65 (–OCH$_2$–), 154.35 (–OCH$_2$–), 133.39 (C_{Ar}), 131.82, 131.80, 117.02, 115.14, 114.28 (C_{Ar}), 95.29 (—C≡C—), 92.38 (—C≡C—), 88.93 (—C≡C—), 88.35 (—C≡C—), 79.54 (–OCH$_2$–), 73.95, 71.79, 70.93, 70.80, 69.51, 67.80, 64.47 (–OCH$_2$–), 38.41 (–CH–), 31.93 (–CH$_2$–), 31.74, 30.26, 29.82, 29.80, 29.77, 29.74, 29.42, 27.10, 14.10 (–CH$_3$) ppm.

1,4-Bis(2-dodecyltetradecyl-1-oxy)-2,5-bis{4-[4-(11,12-dihydroxy-3,6,9-trioxadodecyl-1-oxy)phenylethynyl]phenylethynyl}benzene (C$_3$12/12): Synthesized according to P11 from **8C$_3$12/12** (80 mg, 0.05 mmol) and PPTS (tip of spatula) in MeOH/THF (1:1, 60 mL). Purification by column chromatography (eluent: $CHCl_3$/MeOH = 9:1). Yellow-greenish solid, $C_{108}H_{162}O_{14}$, M = 1683.20 g/mol, yield: 36 mg (47%), **^{1}H-NMR** (400 MHz, pyridine-d$_5$) δ 7.85 – 7.80 (m, 4H, Ar–H), 7.77 – 7.71 (m, 4H, Ar–H), 7.68 – 7.63 (m, 4H, Ar–H), 7.57 (s, 2H, Ar–H), 7.10 – 7.04 (m, 4H, Ar–H), 6.25 (br, 2H, –OH), 5.64 (br, 2H, –OH), 4.42 – 4.34 (m, 2H, –OCH–), 4.20 – 4.06 (m, 12H, –OCH_2–), 3.95 (dd, ^3J(H,H) = 9.8 Hz, ^3J(H,H) = 4.9 Hz, 2H, –OCH_2–), 3.92 – 3.86 (m, 2H, –OCH_2–), 3.86 – 3.81 (m, 2H, –OCH_2–), 3.79 – 3.72 (m, 4H, –OCH_2–), 3.72 – 3.65 (m, 16H, –OCH_2–), 2.09 – 1.98 (m, 2H, –CH–), 1.84 – 1.71 (m, 4H, –CH_2–), 1.71 – 1.17 (m, 80H, –CH_2–), 0.89 (t, ^3J(H,H) = 6.8 Hz, 12H, –CH_3) ppm. **^{13}C-NMR** (101 MHz, pyridine-d$_5$) δ 158.43 (–OCH$_2$–), 153.11 (–OCH$_2$–), 132.19 (C_{Ar}), 130.62, 130.60, 115.85, 113.93, 113.07 (C_{Ar}), 95.85 (—C≡C—), 94.04 (—C≡C—), 91.18 (—C≡C—), 87.75 (—C≡C—), 72.73 (–OCH$_2$–), 70.58, 69.73, 69.58, 69.47, 68.31, 66.59, 63.27, 60.22 (–OCH$_2$–), 57.98, 48.24, 37.18 (–CH–), 30.72 (–CH$_2$–), 30.54, 29.06, 28.62, 28.60, 28.57, 28.54, 28.22, 25.89, 21.53, 12.87 (–CH$_3$) ppm.

1,4-Bis(2-dodecyltetradecyl-1-oxy)-2,5-bis{4-[4-(14,15-dihydroxy-3,6,9,12-tetraoxa-pentadecyl-1-oxy)phenylethynyl]phenylethynyl}benzene (C$_4$12/12): Synthesized according to P11 from **8C$_4$12/12** (80 mg, 0.04 mmol) and PPTS (tip of spatula) in MeOH/THF (1:1, 60 mL). Purification by column chromatography (eluent: $CHCl_3$/MeOH = 9:1). Yellow-greenish solid, $C_{112}H_{170}O_{16}$, M = 1771.25 g/mol, yield: 23 mg (30%), **^{1}H-NMR** (500 MHz, pyridine-d$_5$) δ 7.86 – 7.80 (m, 4H, Ar–H), 7.77 – 7.72 (m, 4H, Ar–H), 7.70 – 7.64 (m, 4H, Ar–H), 7.57 (s, 2H, Ar–H), 7.09 – 7.04 (m, 4H, Ar–H), 6.36 (d, ^3J(H,H) = 4.2 Hz, 2H, –OH), 6.18 (d, ^3J(H,H) = 6.0 Hz, 2H, –OH), 4.41 – 4.33 (m, 2H, –OCH–), 4.21 – 4.05 (m, 12H, –OCH_2–), 3.95 (dd, ^3J(H,H) = 9.8 Hz, ^3J(H,H) = 4.9 Hz, 2H, –OCH_2–), 3.91 – 3.80 (m, 6H, –OCH_2–), 3.79 – 3.61 (m, 24H, –OCH_2–), 2.08 – 2.00 (m, 2H, –CH–), 1.83 – 1.73 (m, 4H, –CH_2–), 1.69 – 1.15 (m, 80H, –CH_2–), 0.89 (t, ^3J(H,H) = 7.0 Hz, 12H, –CH_3) ppm. **^{13}C-NMR** (126 MHz, pyridine-d$_5$) δ 159.66 (–OCH$_2$–), 154.36 (–OCH$_2$–), 133.40 (C_{Ar}), 131.82, 131.80, 117.07, 115.13, 114.23 (C_{Ar}), 95.24 (—C≡C—), 92.33 (—C≡C—), 88.97 (—C≡C—), 88.38 (—C≡C—), 79.54 (–OCH$_2$–), 73.93, 71.78, 70.93, 70.79, 70.64, 69.51, 67.81, 64.47 (–OCH$_2$–), 38.41 (–CH–), 31.92 (–CH$_2$–), 31.74, 30.25, 29.82, 29.80, 29.77, 29.74, 29.42, 27.10, 22.73, 14.07 (–CH$_3$) ppm.

1,4-Bis(2-dodecyltetradecyl-1-oxy)-2,5-bis{4-[4-(17,18-dihydroxy-3,6,9,12,15-penta-oxaoctadecyl-1-oxy)phenylethynyl]phenylethynyl}benzene (C$_5$12/12): Synthesized according to P11 from **8C$_5$12/12** (80 mg, 0.04 mmol) and PPTS (tip of spatula) in MeOH/THF (1:1, 60 mL). Purification by column chromatography (eluent: $CHCl_3$/MeOH = 9:1). Yellow-greenish solid, $C_{116}H_{178}O_{18}$, M = 1859.30 g/mol, yield: 13 mg (17%), **^{1}H-NMR** (500 MHz, pyridine-d$_5$) δ 7.85 – 7.80 (m, 4H, Ar–H), 7.76 – 7.71 (m, 4H, Ar–H), 7.68 – 7.64 (m, 4H, Ar–H), 7.57 (s, 2H, Ar–H), 7.11 – 7.04 (m, 4H, Ar–H), 6.35 (d, ^3J(H,H) = 5.2 Hz, 2H, –OH), 6.17 (t, ^3J(H,H) = 5.9 Hz, 2H, –OH), 4.40 – 4.34 (m, 2H, –OCH–), 4.20 – 4.06 (m, 12H, –OCH_2–), 3.94 (dd, ^3J(H,H) = 9.7 Hz, ^3J(H,H) = 4.9 Hz, 2H, –OCH_2–), 3.91 – 3.82 (m,

6H, –OCH_2–), 3.77 – 3.60 (m, 32H, –OCH_2–), 2.08 – 2.00 (m, 2H, –C*H*–), 1.82 – 1.73 (m, 4H, –CH_2–), 1.69 – 1.18 (m, 80H, –CH_2–), 0.89 (t, ^3J(H,H) = 7.0 Hz, 12H, –CH_3) ppm. **^{13}C-NMR** (126 MHz, pyridine-d$_5$) δ 159.66 (–O*C*H$_2$–), 154.35 (–O*C*H$_2$–), 133.40 (C_{Ar}), 131.82, 131.80, 117.07, 115.13, 114.30, 109.99 (C_{Ar}), 95.22 (—C≡C—), 92.35 (—C≡C—), 88.95 (—C≡C—), 88.37 (—C≡C—), 73.93 (–O*C*H$_2$–), 71.78, 70.93, 70.80, 70.65, 70.64, 69.51, 67.81, 64.47 (–O*C*H$_2$–), 38.41 (–*C*H–), 31.92 (–*C*H$_2$–), 31.74, 30.25, 29.82, 29.80, 29.77, 29.74, 29.42, 27.10, 22.73, 14.07 (–*C*H$_3$) ppm.

2.6.6 Compounds C_x18/18

1,4-Bis(2-octadecyleicosyl-1-oxy)-2,5-bis{4-[4-(5,6-dihydroxy-3-oxahexyl-1-oxy)phenylethynyl]phenylethynyl}benzene ($C_1$18/18): Synthesized according to P11 from **8$C_1$18/18** (90 mg, 0.05 mmol) and PPTS (tip of spatula) in MeOH/THF (1:1, 60 mL). Purification by column chromatography (eluent: $CHCl_3$/MeOH = 9:1). Yellow-greenish solid, $C_{124}H_{194}O_{10}$, M = 1843.47 g/mol, yield: 35 mg (41%), **^{1}H-NMR** (400 MHz, pyridine-d$_5$) δ 7.87 – 7.80 (m, 4H, Ar–*H*), 7.78 – 7.73 (m, 4H, Ar–*H*), 7.69 – 7.63 (m, 4H, Ar–*H*), 7.57 (s, 2H, Ar–*H*), 7.11 – 7.03 (m, 4H, Ar–*H*), 4.46 – 4.37 (m, 2H, –OC*H*–), 4.24 – 4.07 (m, 12H, –OCH_2–), 4.02 (dd, ^3J(H,H) = 9.8 Hz, ^3J(H,H) = 4.7 Hz, 2H, –OCH_2–), 3.98 – 3.89 (m, 6H, –OCH_2–), 2.10 – 1.99 (m, 2H, –C*H*–), 1.85 – 1.71 (m, 4H, –CH_2–), 1.71 – 1.14 (m, 128H, –CH_2–), 0.89 (t, ^3J(H,H) = 6.6 Hz, 12H, –CH_3) ppm. **^{13}C-NMR** (101 MHz, pyridine-d$_5$) δ 158.48 (–O*C*H$_2$–), 153.14 (–O*C*H$_2$–), 132.19 (C_{Ar}), 130.63, 130.60, 115.85, 113.93, 113.10 (C_{Ar}), 94.05 (—C≡C—), 91.18 (—C≡C—), 87.76 (—C≡C—), 87.18 (—C≡C—), 78.34 (–O*C*H$_2$–), 72.90, 71.23, 70.60, 68.62, 66.65, 63.19 (–O*C*H$_2$–), 37.20 (–*C*H–), 30.71 (–*C*H$_2$–), 30.53, 29.04, 28.62, 28.59, 28.58, 28.52, 28.20, 25.88, 21.52, 12.86 (–*C*H$_3$) ppm.

1,4-Bis(2-octadecyleicosyl-1-oxy)-2,5-bis{4-[4-(8,9-dihydroxy-3,6-dioxanonyl-1-oxy)-phenylethynyl]phenylethynyl}benzene ($C_2$18/18): Synthesized according to P11 from **8$C_2$18/18** (100 mg, 0.05 mmol) and PPTS (tip of spatula) in MeOH/THF (1:1, 60 mL). Purification by column chromatography (eluent: $CHCl_3$/MeOH = 9:1). Yellow-greenish solid, $C_{128}H_{202}O_{12}$, M = 1931.52 g/mol, yield: 54 mg (56%), **^{1}H-NMR** (400 MHz, pyridine-d$_5$) δ 7.85 – 7.80 (m, 4H, Ar–*H*), 7.77 – 7.72 (m, 4H, Ar–*H*), 7.69 – 7.63 (m, 4H, Ar–*H*), 7.57 (s, 2H, Ar–*H*), 7.10 – 7.04 (m, 4H, Ar–*H*), 4.43 – 4.35 (m, 2H, –OC*H*–), 4.21 – 4.07 (m, 12H, –OCH_2–), 3.97 (dd, ^3J(H,H) = 9.8 Hz, ^3J(H,H) = 4.9 Hz, 2H, –OCH_2–), 3.90 (dd, ^3J(H,H) = 9.8 Hz, ^3J(H,H) = 6.1 Hz, 2H, –OCH_2–), 3.88 – 3.82 (m, 4H, –OCH_2–), 3.81 – 3.71 (m, 8H, –OCH_2–), 2.10 – 1.98 (m, 2H, –C*H*–), 1.85 – 1.72 (m, 4H, –CH_2–), 1.72 – 1.18 (m, 128H, –CH_2–), 0.89 (t, ^3J(H,H) = 6.8 Hz, 12H, –CH_3) ppm. **^{13}C-NMR** (126 MHz, pyridine-d$_5$) δ 159.65 (–O*C*H$_2$–), 154.32 (–O*C*H$_2$–), 133.39 (C_{Ar}), 131.82, 131.80, 118.72, 115.21, 115.13, 114.28 (C_{Ar}), 95.25 (—C≡C—), 92.37 (—C≡C—), 88.94 (—C≡C—), 88.36 (—C≡C—), 79.54 (–O*C*H$_2$–), 73.95, 72.39, 71.79, 70.93, 70.80, 69.52, 67.80, 64.47 (–O*C*H$_2$–), 31.92 (–*C*H$_2$–), 31.73, 30.24, 29.82, 29.79, 29.78, 29.76, 29.72, 29.40, 27.08, 22.73, 14.07 (–*C*H$_3$) ppm.

1,4-Bis(2-octadecyleicosyl-1-oxy)-2,5-bis{4-[4-(11,12-dihydroxy-3,6,9-trioxadodecyl-1-oxy)phenylethynyl]phenylethynyl}benzene ($C_3$18/18): Synthesized according to P11 from **8$C_3$18/18** (90 mg, 0.04 mmol) and PPTS (tip of spatula) in MeOH/THF (1:1, 60 mL). Purification by column chromatography (eluent: $CHCl_3$/MeOH = 9:1). Yellow-greenish solid, $C_{132}H_{210}O_{14}$, M = 2019.57 g/mol, yield: 51 mg (58%), **^{1}H-NMR** (400 MHz, pyridine-d$_5$) δ 7.86 – 7.80 (m, 4H, Ar–*H*), 7.78 – 7.72 (m, 4H, Ar–*H*), 7.69 – 7.63 (m, 4H, Ar–*H*), 7.57 (s, 2H, Ar–*H*), 7.10 – 7.04 (m, 4H, Ar–*H*), 6.36 (br, 2H, –O*H*), 6.20 (br, 2H, –O*H*), 4.43 – 4.33 (m, 2H, –OC*H*–), 4.23 – 4.05 (m, 12H, –OCH_2–), 3.95 (dd, ^3J(H,H) = 9.8 Hz, ^3J(H,H) = 4.9 Hz, 2H, –OCH_2–), 3.92 – 3.81 (m, 6H, –OCH_2–), 3.80 – 3.65 (m, 16H, –OCH_2–), 2.11 – 1.99 (m, 2H, –C*H*–), 1.85 – 1.72 (m, 4H, –CH_2–), 1.72 – 1.17 (m, 128H, –CH_2–), 0.90 (t, ^3J(H,H)

= 6.8 Hz, 12H, –CH_3) ppm. **^{13}C-NMR** (101 MHz, pyridine-d$_5$) δ 159.97 (–OCH$_2$–), 154.65 (–OCH$_2$–), 133.71 (C_{Ar}), 132.11, 118.97, 115.44, 114.59 (C_{Ar}), 95.46 (—C≡C—), 92.76 (—C≡C—), 88.66 (—C≡C—), 87.97 (—C≡C—), 74.25 (–OCH$_2$–), 72.09, 71.24, 71.10, 70.99, 69.82, 68.11, 64.78 (–OCH$_2$–), 32.23 (–CH_2–), 32.04, 30.55, 30.13, 30.10, 30.03, 29.72, 27.39, 23.04, 14.38 (–CH$_3$) ppm.

1,4-Bis(2-octadecyleicosyl-1-oxy)-2,5-bis{4-[4-(14,15-dihydroxy-3,6,9,12-tetraoxapentadecyl-1-oxy)phenylethynyl]phenylethynyl}benzene ($C_4$18/18): Synthesized according to P11 from **8$C_4$18/18** (100 mg, 0.05 mmol) and PPTS (tip of spatula) in MeOH/THF (1:1, 60 mL). Purification by column chromatography (eluent: $CHCl_3$/MeOH = 9:1). Yellow-greenish solid, $C_{136}H_{218}O_{16}$, *M* = 2107.62 g/mol, yield: 27 mg (28%), **^{1}H-NMR** (500 MHz, pyridine-d$_5$) δ 7.86 – 7.80 (m, 4H, Ar–*H*), 7.77 – 7.72 (m, 4H, Ar–*H*), 7.69 – 7.64 (m, 4H, Ar–*H*), 7.57 (s, 2H, Ar–*H*), 7.11 – 7.05 (m, 4H, Ar–*H*), 4.41 – 4.34 (m, 2H, –OC*H*–), 4.21 – 4.06 (m, 12H, –OCH_2–), 3.94 (dd, ^3J(H,H) = 9.7 Hz, ^3J(H,H) = 4.9 Hz, 2H, –OCH_2–), 3.92 – 3.82 (m, 6H, –OCH_2–), 3.78 – 3.63 (m, 32H, –OCH_2–), 2.09 – 2.00 (m, 2H, –C*H*–), 1.83 – 1.74 (m, 4H, –CH_2–), 1.70 – 1.16 (m, 128H, –CH_2–), 0.90 (t, ^3J(H,H) = 7.0 Hz, 12H, –CH_3) ppm. **^{13}C-NMR** (126 MHz, pyridine-d$_5$) δ 159.66 (–OCH$_2$–), 154.38 (–OCH$_2$–), 133.40 (C_{Ar}), 131.82, 131.81, 117.02, 115.20, 115.13, 114.29 (C_{Ar}), 95.20 (—C≡C—), 92.36 (—C≡C—), 88.98 (—C≡C—), 88.40 (—C≡C—), 79.54 (–OCH$_2$–), 73.92, 72.32, 71.76, 70.92, 70.79, 70.64, 69.51, 67.80, 64.46 (–OCH$_2$–), 38.39 (–C*H*–), 31.92 (–CH_2–), 31.73, 30.24, 29.82, 29.79, 29.78, 29.76, 29.72, 29.40, 27.07, 22.73, 14.07 (–CH$_3$) ppm.

1,4-Bis(2-octadecyleicosyl-1-oxy)-2,5-bis{4-[4-(17,18-dihydroxy-3,6,9,12,15-pentaoxaoctadecyl-1-oxy)phenylethynyl]phenylethynyl}benzene ($C_5$18/18): Synthesized according to P11 from **8$C_5$18/18** (110 mg, 0.05 mmol) and PPTS (tip of spatula) in MeOH/THF (1:1, 60 mL). Purification by column chromatography (eluent: $CHCl_3$/MeOH = 9:1). Yellow-greenish solid, $C_{140}H_{226}O_{18}$, *M* = 2195.68 g/mol, yield: 75 mg (70%), **^{1}H-NMR** (500 MHz, pyridine-d$_5$) δ 7.87 – 7.80 (m, 4H, Ar–*H*), 7.77 – 7.72 (m, 4H, Ar–*H*), 7.70 – 7.64 (m, 4H, Ar–*H*), 7.57 (s, 2H, Ar–*H*), 7.10 – 7.04 (m, 4H, Ar–*H*), 4.41 – 4.34 (m, 2H, –OC*H*–), 4.21 – 4.06 (m, 12H, –OCH_2–), 3.94 (dd, ^3J(H,H) = 9.7 Hz, ^3J(H,H) = 4.9 Hz, 2H, –OCH_2–), 3.91 – 3.83 (m, 6H, –OCH_2–), 3.78 – 3.60 (m, 32H, –OCH_2–), 2.08 – 2.00 (m, 2H, –C*H*–), 1.84 – 1.73 (m, 4H, –CH_2–), 1.69 – 1.19 (m, 128H, –CH_2–), 0.90 (t, ^3J(H,H) = 7.0 Hz, 12H, –CH_3) ppm. **^{13}C-NMR** (126 MHz, pyridine-d$_5$) δ 159.66 (–OCH$_2$–), 154.35 (–OCH$_2$–), 133.40 (C_{Ar}), 131.83, 131.81, 117.06, 115.22, 115.13, 114.31, 109.99 (C_{Ar}), 95.25 (—C≡C—), 92.38 (—C≡C—), 88.96 (—C≡C—), 88.35 (—C≡C—), 73.92 (–OCH$_2$–), 72.39, 71.77, 70.92, 70.80, 70.66, 70.64, 69.51, 67.81, 65.93, 64.46 (–OCH$_2$–), 46.41, 38.40 (–C*H*–), 31.92 (–CH_2–), 31.73, 30.24, 29.82, 29.79, 29.78, 29.76, 29.72, 29.40, 27.07, 22.73, 14.07 (–CH$_3$) ppm.

2.7 Synthesis of the compounds with semiperfluorinated chains (D_x4F6, E_x12/4F6, E18/4F6)

2.7.1 1,4-Bis[4-(4-hydroxyphenylethynyl)phenylethynyl]-2,5-bis(5,5,6,6,7,7,8,8,9,9,-10,10,10-tridecafluoro-*n*-decyl-1-oxy)benzene (15k)

1,4-Bis[4-(4-triisopropylsilyloxyphenylethynyl)phenylethynyl]-2,5-bis(5,5,6,6,7,7,8,8,9,9,10,10,10-tridecafluorodecyl-1-oxy)benzene (14k): Synthesized according P6 from **7k** (700 mg, 0.6 mmol), **13** (514 mg, 1.4 mmol), [Pd(PPh_3)$_4$] (35 mg, 0.03 mmol), CuI (6 mg, 0.03 mmol), NEt_3 (10 mL) in THF (30 mL). Purification by column chromatography (eluent: *n*-hexane/EtOAc = 9:1). Yellowish oil, $C_{76}H_{76}F_{26}O_4Si_2$, *M* = 1603.54 g/mol, yield: 700 mg (70%), **^{1}H-NMR** ($CDCl_3$, 400 MHz) δ 7.46 (m, 8H, Ar–*H*), 7.40 (d, ^3J(H,H) = 8.3 Hz, 4H, Ar–*H*), 7.02 (s, 2H, Ar–*H*), 6.86 (d, ^3J(H,H) = 8.2 Hz, 4H, Ar–*H*), 4.09 (t, ^3J(H,H) = 5.4 Hz,

4H, –OCH_2–), 2.29 – 2.11 (m, 4H, –O$CH_2$$CH_2$–), 2.01 – 1.83 (m, 8H, –O$CH_2CH_2CH_2CH_2CF_2$–), 1.38 – 1.20 (m, 6H, –Si$CH(CH_3)_3$), 1.11 (d, ^3J(H,H) = 7.3 Hz, 36H, –Si$CH(CH_3)_3$) ppm.

1,4-Bis[4-(4-hydroxyphenylethynyl)phenylethynyl]-2,5-bis(5,5,6,6,7,7,8,8,9,9,10,10,10-tridecafluorodecyl-1-oxy)benzene (15k): Synthesized according P10 from **14k** (700 mg, 0.4 mmol), Bu_4NF (1 M in THF, 2 mL) in THF (50 mL). Purification by column chromatography (eluent: $CHCl_3$/MeOH = 49:1). Yellow solid, $C_{58}H_{36}F_{26}O_4$, *M* = 1290.86 g/mol, yield: 250 mg (44%), **^{1}H-NMR** (thf-d_8, 400 MHz) δ 7.47 (m, 8H, Ar–*H*), 7.34 (d, ^3J(H,H) = 8.4 Hz, 4H, Ar–*H*), 7.12 (s, 2H, Ar–*H*), 6.75 (d, ^3J(H,H) = 8.5 Hz, 4H, Ar–*H*), 4.69 (s, 2H, –O*H*), 4.13 (t, ^3J(H,H) = 5.5 Hz, 4H, –OCH_2–), 2.34 (m, 4H, –OCH_2CH_2–), 1.95 (m, 1H, –$CH_2CH_2CF_2$–) ppm.

2.7.2 Compounds D_x4F6

1,4-Bis{4-[4-(2,3-dihydroxypropyl-1-oxy)phenylethynyl]phenylethynyl}-2,5-bis(5,5,6,6,7,7,8,8,9,9,10,10,10-tridecafluorodecyl-1-oxy)benzene (D4F6): Synthesized according to P5 from **15k** (50 mg, 0.04 mmol), **17a** (15 mg, 0.09 mmol), K_2CO_3 (27 mg, 0.19 mmol), Bu_4NI (10 mg, 0.03 mmol) in DMF (50 mL). Purification by column chromatography (eluent: $CHCl_3$/MeOH = 9:1), recrystallization from MeOH. Yellowish solid, $C_{64}H_{48}F_{26}O_8$, *M* = 1439.02 g/mol, yield: 18 mg (33%), mp. 136 °C, **^{1}H-NMR** (pyridine-d_5, 400 MHz) δ 7.76 (d, ^3J(H,H) = 8.4 Hz, 4H, Ar–*H*), 7.70 (d, ^3J(H,H) = 8.4 Hz, 4H, Ar–*H*), 7.64 (d, ^3J(H,H) = 8.8 Hz, 4H, Ar–*H*), 7.50 (s, 2H, Ar–*H*), 7.11 (d, ^3J(H,H) = 8.9 Hz, 4H, Ar–*H*), 4.57 (m, 2H, –CH_2CHCH_2–), 4.52 (m, 2H, –OCH_2–), 4.43 (m, 2H, –OCH_2–), 4.23 (d, ^3J(H,H) = 5.4 Hz, 4H, –OCH_2–), 4.18 (m, 4H, –OCH_2–), 2.33 (m, 4H, –CH_2–), 1.97 (m, 8H, –CH_2–) ppm. **^{13}C-NMR** (pyridine-d_5, 100 MHz) δ 16.2 (–$CH_2CH_2CF_2$–), 27.5, 28.6, 29.2 (–CH_2CF_2–), 62.8, 67.6, 69.7, 69.9 (–OCH–, –OCH_2–), 87.1, 87.3, 91.2, 94.1, 113.1, 113.9, 116.1, 130.5, 130.6, 132.2, 152.6, 158.5 (C_{Ar}) ppm. **^{19}F-NMR** (pyridine-d_5, 376 MHz): δ -80.83 (tt, ^3J(F,F) = 9.8 Hz, ^4J(F,F) = 2.1 Hz, –CF_3), -115.25 (m, –CF_3), -122.97 (s, –CF_2–), -123.94 (s, –CF_2–), -124.39 (s, –CF_2–), -127.23 (s, –CF_2–) ppm. **HRMS** (m/z): $[M]^+Cl^-$ calcd. for $C_{64}H_{48}F_{26}O_8Cl$, 1473.2617; found, 1473.2632.

1,4-Bis{4-[4-(5,6-dihydroxy-3-oxahexyl-1-oxy)phenylethynyl]phenylethynyl}-2,5-bis(5,5,6,6,7,7,8,8,9,9,10,10,10-tridecafluorodecyl-1-oxy)benzene ($D_1$4F6): Synthesized according to P5 from **15k** (50 mg, 0.04 mmol), **17b** (17 mg, 0.09 mmol), K_2CO_3 (27 mg, 0.19 mmol), Bu_4NI (10 mg, 0.03 mmol) in DMF (50 mL). Purification by column chromatography (eluent: $CHCl_3$/MeOH = 9:1), recrystallization from MeOH, yellowish solid, $C_{68}H_{56}F_{26}O_{10}$, *M* = 1527.12 g/mol, yield: 55 mg (90%), mp. 137 °C, **^{1}H-NMR** (pyridine-d_5, 400 MHz) δ 7.77 (d, ^3J(H,H) = 8.0 Hz, 4H, Ar–*H*), 7.70 (d, ^3J(H,H) = 8.0 Hz, 4H, Ar–*H*), 7.64 (d, ^3J(H,H) = 8.2 Hz, 4H, Ar–*H*), 7.50 (s, 2H, Ar–*H*), 7.05 (d, ^3J(H,H) = 8.3 Hz, 4H, Ar–*H*), 6.52 (s, 2H, –O*H*), 6.27 (s, 2H, –O*H*), 4.47 – 4.34 (m, 2H, –CH_2CHCH_2–), 4.24 – 4.07 (m, 12H, –OCH_2–), 4.02 (m, 2H, –OCH_2–), 3.99 – 3.90 (m, 6H, – OCH_2–), 2.33 (m, 4H, –CH_2–), 1.97 (m, 8H, –CH_2–) ppm. **^{13}C-NMR** (pyridine-d_5, 100 MHz) δ 17.7 (t, ^3J(C,F) = 3.5 Hz, –$CH_2CH_2CF_2$–), 28.8, 30.0, 30.7 (t, ^2J(C,F) = 22.1 Hz, –CH_2CF_2–), 64.6, 68.1, 69.0, 70.0, 72.0, 74.3 (–OCH–, –OCH_2–), 88.5, 88.7, 92.6, 95.5, 114.5, 115.4, 117.5, 132.0, 132.0, 133.6, 154.1, 159.9 (C_{Ar}) ppm. **^{19}F-NMR** (pyridine-d_5, 470 MHz) δ -80.83 (tt, ^3J(F,F) = 9.8 Hz, ^4J(F,F) = 2.1 Hz, –CF_3), -113.94 (m, –CF_3), -121.90 (s, –CF_2–), -122.86 (s, –CF_2–), -123.29 (s, –CF_2–), -126.04 (m, –CF_2–) ppm. **HRMS** (m/z): $[M]^+Cl^-$ calcd. for $C_{68}H_{56}F_{26}O_{10}Cl$, 1561.3141; found, 1561.3327.

1,4-Bis{4-[4-(8,9-dihydroxy-3,6-dioxanonyl-1-oxy)phenylethynyl]phenylethynyl}-2,5-bis(5,5,6,6,7,7,8,8,9,9,10,10,10-tridecafluorodecyl-1-oxy)benzene (D_24F6): Synthesized according to P5 from **15k** (50 mg, 0.04 mmol), **17c** (21 mg, 0.09 mmol), K_2CO_3 (27 mg, 0.19 mmol), Bu_4NI (10 mg, 0.03 mmol) in DMF (50 mL). Purification by column chromatography (eluent: $CHCl_3$/MeOH = 9:1). Recrystallization from MeOH. Yellowish solid, $C_{72}H_{64}F_{26}O_{12}$, M = 1615.23 g/mol, yield: 38 mg (62%), mp. 103 °C, **^{1}H-NMR** (pyridine-d_5, 400 MHz) δ 7.76 (d, ^3J(H,H) = 7.0 Hz, 4H, Ar–*H*), 7.70 (d, ^3J(H,H) = 8.2 Hz, 4H, Ar–*H*), 7.64 (d, ^3J(H,H) = 7.2 Hz, 4H, Ar–*H*), 7.50 (s, 2H, Ar–*H*), 7.06 (d, ^3J(H,H) = 7.5 Hz, 4H, Ar–*H*), 4.39 (m, 2H, –CH_2C*H*CH_2–), 4.23 – 4.06 (m, 12H, –OCH_2–), 3.97 (m, 2H, –OCH_2–), 3.90 (m, 2H, –OCH_2–), 3.88 – 3.82 (m, 4H, –OCH_2–), 3.75 (m, 8H, –OCH_2–), 2.33 (m, 4H, –CH_2–), 1.97 (m, 8H, –CH_2–) ppm. **^{13}C-NMR** (pyridine-d_5, 100 MHz) δ 17.4 (–C$H_2$$CH_2$$CF_2$–), 28.6, 29.8, 30.4 (t, ^2J(C,F) = 21.8 Hz, –C$H_2$$CF_2$–), 64.4, 67.8, 68.8, 69.5, 70.8, 70.9, 71.8, 73.9 (–O*C*H–, –O*C*H_2–), 88.3, 88.5, 92.3, 95.3, 114.3, 115.1, 117.3, 131.7, 131.8, 133.4, 153.8 159.6 (C_{Ar}) ppm. **^{19}F-NMR** (pyridine-d_5, 470 MHz) δ -80.83 (tt, ^3J(F,F) = 9.8 Hz, ^4J(F,F) = 2.1 Hz, –CF_3), -114.04 (m, –CF_3), -121.78 (s, –CF_2–), -122.76 (s, –CF_2–), -123.20 (s, –CF_2–), -126.04 (s, –CF_2–) ppm. **HRMS** (m/z): $[M]^+Cl^-$ calcd. for $C_{72}H_{64}F_{26}O_{12}Cl$, 1649.3666; found, 1649.3895.

1,4-Bis{4-[4-(11,12-dihydroxy-3,6,9-trioxadodecyl-1-oxy)phenylethynyl]phenylethynyl}-2,5-bis(5,5,6,6,7,7,8,8,9,9,10,10,10-tridecafluorodecyl-1-oxy)benzene (D_34F6): Synthesized according to P5 from **15k** (50 mg, 0.04 mmol), **17d** (40 mg, 0.12 mmol), K_2CO_3 (30 mg, 0.19 mmol), Bu_4NI (10 mg, 0.03 mmol) in DMF. Purification by column chromatography (eluent: $CHCl_3$/MeOH = 9:1), recrystallization from MeOH. Yellowish solid, $C_{76}H_{72}F_{26}O_{14}$, M = 1703.33 g/mol, yield: 30 mg (45%), mp. 107 °C, **^{1}H-NMR** ($CDCl_3$, 400 MHz): δ 7.46 (m, 12H, Ar–*H*), 7.02 (s, 2H, Ar–*H*), 6.90 (d, ^3J(H,H) = 8.8 Hz, 4H, Ar–*H*), 4.19 – 4.14 (m, 4H, –CH_2C*H*CH_2–, –OCH_2–), 4.08 (m, 4H, –OCH_2–), 3.90 – 3.84 (m, 6H, –OCH_2–), 3.74 (m, 4H, –OCH_2–), 3.71 – 3.54 (m, 20H, –OCH_2–), 3.48 (s, 2H, –O*H*), 3.06 (s, 2H, –O*H*), 2.28 – 2.11 (m, 6H, –CH_2–), 1.95 (m, 8H, –CH_2–) ppm. **^{13}C-NMR** ($CDCl_3$, 100 MHz) δ 17.7 (t, ^3J(C,F) = 3.5 Hz, –C$H_2$$CH_2$$CF_2$–), 28.8, 30.0, 30.7 (t, ^2J(C,F) = 22.1 Hz, –C$H_2$$CF_2$–), 63.9, 67.5, 68.7, 69.6, 70.4, 70.5, 70.5, 70.7, 70.8, 73.0 (–O*C*H–, –O*C*H_2–), 88.5, 88.7, 92.6, 95.5 (CC), 114.5, 115.4, 117.5, 132.0, 132.0, 133.6, 154.1, 159.9 (C_{Ar}) ppm. **^{19}F-NMR** ($CDCl_3$, 376 MHz): δ / ppm = -80.78 (t, ^3J(F,F) = 10.0 Hz, –CF_3), -114.11 – -114.58 (m, –CF_2–), -121.89 (s, –CF_2–), -122.85 (s, –CF_2–), -123.45 (s, –CF_2–), -126.10 (m, –CF_2–) ppm. **HRMS** (m/z): $[M]^+Cl^-$ calcd. for $C_{76}H_{72}F_{26}O_{14}Cl$, 1737.4190; found, 1737.4383.

1,4-Bis{4-[4-(17,18-dihydroxy-3,6,9,12,15-pentaoxaoctadecyl-1-oxy)phenylethynyl]-phenylethynyl}-2,5-bis(5,5,6,6,7,7,8,8,9,9,10,10,10-tridecafluorodecyl-1-oxy)benzene (D_54F6): Synthesized according to P5 from **15k** (50 mg, 0.04 mmol), **17f** (33 mg, 0.09 mmol), K_2CO_3 (27 mg, 0.19 mmol), Bu_4NI (10 mg, 0.03 mmol) in DMF (50 mL). Purification by column chromatography (eluent: $CHCl_3$/MeOH = 9:1). Recrystallization from MeOH, yellowish solid, $C_{84}H_{88}F_{26}O_{18}$, M = 1879.55 g/mol, yield: 72 mg (85%), mp. 98 °C, **^{1}H-NMR** (pyridine-d_5, 400 MHz) δ 7.76 (d, ^3J(H,H) = 8.3 Hz, 4H, Ar–*H*), 7.70 (d, ^3J(H,H) = 8.2 Hz, 4H, Ar–*H*), 7.65 (d, ^3J(H,H) = 8.6 Hz, 4H, Ar–*H*), 7.50 (s, 2H, Ar–*H*), 7.07 (d, ^3J(H,H) = 8.7 Hz, 4H, Ar–*H*), 6.41 – 6.31 (m, 2H, –O*H*), 6.24 – 6.13 (m, 2H, –O*H*), 4.37 (m, 2H, –CH_2C*H*CH_2–), 4.13 (m, 12H, –OCH_2–), 3.99 – 3.92 (m, 2H, –OCH_2–), 3.89 (m, 2H, –OCH_2–), 3.87 – 3.81 (m, 4H, –OCH_2–), 3.80 – 3.59 (m, 32H, –OCH_2–), 2.33 (m, 4H, –CH_2–), 1.99 (m, 8H, –CH_2–) ppm. **^{13}C-NMR** (pyridine-d_5, 100 MHz) δ 16.2 (t, ^3J(C,F) = 4.0 Hz, –*C*$H_2$$CH_2$$CF_2$–), 27.5, 28.6, 29.2 (t, ^2J(C,F) = 21.9 Hz, –*C*$H_2$$CF_2$–), 63.3, 66.6, 67.6, 68.3, 69.4, 69.6, 69.7, 70.6, 72.7 (–O*C*H–, –O*C*H_2–), 87.1, 87.3, 91.2, 94.1, 113.1, 113.9, 116.1, 130.5, 130.6, 132.2, 152.6, 158.5 (C_{Ar}) ppm. **^{19}F-NMR** (pyridine-d_5, 376 MHz) δ -82.05 (t, ^3J(F,F) = 9.8 Hz, –CF_3), -114.75 – -115.17 (m, –CF_2–), -122.97 (s, –CF_2–), -123.95 (s, –CF_2–), -124.39

(s, $–CF_2–$), -127.06 – -127.34 (m, $–CF_2–$) ppm. **HRMS** (m/z): $[M]^+Cl^-$ calcd. for $C_{84}H_{88}F_{26}O_{18}Cl$, 1913.5239; found, 1913.5361.

2.7.3 Compounds E*n*/4F6

5-Dodecyloxy-2-(5,5,6,6,7,7,8,8,9,9,10,10,10-tridecafluorodecyl-1-oxy)-1,4-bis{4-[4-(triisopylsilyloxy)phenylethynyl]phenylethynyl}benzene (8E12/4F6):
Synthesized according to P6 from **7l** (300 mg, 0.37 mmol), **6** (295 mg, 0.89 mmol), $[Pd(PPh_3)_4]$ (13 mg, 0.01 mmol), CuI (1 mg, 0.01 mmol) in NEt_3 (30 mL). Purification by column chromatography (eluent: CH_2Cl_2), recrystallization from CH_2Cl_2/Et_2O. Yellow solid, $C_{72}H_{73}F_{13}O_8$, M = 1313.34 g/mol, yield: 53 mg (11%), mp. 147-149 °C, ^{1}**H NMR** (400 MHz, $CDCl_3$) δ 7.51 – 7.40 (m, 12H, Ar–*H*), 7.00 (s, 1H, Ar–*H*), 6.99 (s, 1H, Ar–*H*),6.92 – 6.85 (m, 4H, Ar–*H*), 4.52 – 4.42 (m, 2H, –C*H*–), 4.16 (dd, ^3J(H,H) = 8.4, ^3J(H,H) = 6.5 Hz, 2H, –OCH_2–), 4.11 – 3.99(m, 6H, –OCH_2CH_2–, –CH_2O–), 3.95 (dd, ^3J(H,H) = 9.5, ^3J(H,H) = 5.9 Hz, 2H, –CH_2O–), 3.89 (dd, ^3J(H,H) = 8.4, ^3J(H,H) = 5.8 Hz, 2H, –OCH_2–), 2.29 – 2.03 (m, 2H, –CH_2CF_2–), 2.03 – 1.78 (m, 6H, –$CH_2CH_2CF_2$–, –OCH_2CH_2–), 1.59 – 1.47 (m,2H, –$OCH_2CH_2CH_2$–), 1.45 (s, 6H, –CH_3), 1.42 – 1.15 (m, 22H, –CH_3, –$OCH_2CH_2CH_2(CH_2)_8$–), 0.85 (t, ^3J(H,H) = 6.6 Hz, 3H, –CH_2CH_3) ppm.

5-Octadecyloxy-2-(5,5,6,6,7,7,8,8,9,9,10,10,10-tridecafluorodecyl-1-oxy)-1,4-bis{4-[4-(2,3-*O*-isopropylidene-2,3-dihydroxypropyl-1-oxy)phenylethynyl]phenylethynyl)benzene (8E18/4F6): Synthesized according to P6 from **7m** (0.57 g, 0.64 mmol), **6** (0.53 g, 1.60 mmol), $[Pd(PPh_3)_4]$ (22 mg, 0.02 mmol), CuI (3 mg, 0.01 mmol) in NEt_3 (30 mL). Purification by column chromatography (eluent: $CHCl_3/Et_2O$ = 10:0.1). Yellow solid, $C_{78}H_{85}F_{13}O_8$, M = 1397.49 g/mol, yield: 290 mg (32%), mp. 137 – 139 °C, ^{1}**H-NMR** ($CDCl_3$, 400 MHz) δ 7.50-7.43 (m, 12H, Ar−*H*), 7.00 (s, 1H, Ar−*H*), 6.99 (s, 1H, Ar−*H*), 6.88 (m, 4H, Ar−*H*), 4.44 (dddd, ^3J(H,H) = 6.4 Hz, ^3J(H,H) = 5.4 Hz, ^3J(H,H) = 5.81 Hz, ^3J(H,H) = 5.84 Hz, 2H, –OCH–), 4.14 (dddd, ^2J(H,H) = 8.6 Hz, ^3J(H,H) = 6.4 Hz, ^4J(H,H) = 0.5 Hz, ^4J(H,H) = 0.1 Hz, 2H, –OCH_2–), 4.08-4.00 (m, 6H, –$OCH_2(CH_2)_2CH_2CF_2$–, –CH_2O–), 3.89 (dddd, ^2J(H,H) = 9.6 Hz, ^3J(H ,H) = 5.8 Hz, ^4J(H,H) = 0.1 Hz, ^4J(H,H) = 0.4 Hz, 2H, –CH_2O–), 3.87 (ddd, ^2J(H,H) = 8.6 Hz, ^3J(H,H) = 5.8 Hz, ^4J(H,H) = 0.4 Hz, 2H, –OCH_2–), 2.19 (m, 2H, –CH_2CF_2–), 1.88 (m, 4H, –$OCH_2(CH_2)_2CH_2CF_2$–), 1.78 (m, 2H, –OCH_2CH_2–), 1.46 (m, 2H, –$OCH_2CH_2CH_2$–), 1.45 (s, 6H, –CH_3), 1.39 (s, 6H, –CH_3), 1.40-1.24 (m, 28H, –CH_2–), 0.86 (t, ^3J(H,H) = 6.6 Hz, 3H, –CH_3) ppm.

5-Dodecyloxy-2-(5,5,6,6,7,7,8,8,9,9,10,10,10-tridecafluorodecyl-1-oxy)-1,4-bis{4-[4-(2,3-dihydroxypropyl-1-oxy)phenylethynyl]phenylethynyl}benzene (E12/4F6): Synthesized according to P11 from **8E12/4F6** (53 mg, 0.04 mmol), PPTS (2 mg, 0.01 mmol) in THF (30 mL) and MeOH (10 mL). Purification by column chromatography (eluent: EtOAc). Recrystallisation from THF/MeOH. Yellow solid, $C_{66}H_{65}F_{12}O_8$, M = 1233.21 g/mol, yield: 33 mg (67%), mp. 112 °C, ^{1}**H-NMR** (400 MHz, thf-d_8) δ 7.60 – 7.37 (m, 12H, Ar–*H*), 7.11 (s, 1H, Ar–*H*), 7.10 (s, 1H, Ar–*H*), 7.01 – 6.87 (m, 4H, Ar–*H*), 4.17 (d, ^3J(H,H) = 5.1 Hz, 2H, –CHO*H*), 4.15 – 4.09 (m, 2H, –OCH_2CH_2–), 4.09 – 4.00 (m, 4H, –OCH_2CH_2–), 3.96 (dd, ^3J(H,H) = 9.4, ^3J(H,H) = 6.1 Hz, 2H), 3.92 – 3.83 (m, 2H, –C*H*–), 3.78 – 3.70 (m, 2H, –CH_2OH), 3.64 – 3.50 (m, 4H), 2.33 (s, 2H, –CH_2CF_2–), 2.00 – 1.82 (m, 6H, –OCH_2CH_2–, –$CH_2CH_2CF_2$–), 1.64 – 1.53 (m, 2H, –$OCH_2CH_2CH_2$–), 1.49 – 1.17 (m, 16H, –$OCH_2CH_2CH_2(CH_2)_8$–), 0.88 (t, ^3J(H,H) = 6.3 Hz, 3H, –CH_3) ppm. ^{19}**F-NMR** (376 MHz, thf-d_8) δ -81.80 (t, ^3J(C,F) = 10.1 Hz), -114.66 – (-114.99) (m), -122.47 (s), -123.43 (s), -124.01 (s), -126.81 (s) ppm. **HRMS** (m/z): $[M]^+Cl^-$ calcd. for $C_{66}H_{65}F_{13}O_8Cl$, 1267.4155; found, 1267.3988.

5-Octadecyloxy-2-(5,5,6,6,7,7,8,8,9,9,10,10,10-tridecyfluorodecyl-1-oxy)-1,4-bis{4-[4-(2,3-dihydroxypropyl-1-oxy)phenylethynyl]phenylethynyl}benzene (E18/4F6): Synthesized according to P11 from **8E18/4F6** (290 mg, 0.21 mmol), PPTS (10 mg, 0.04 mmol) in THF (20 mL) and MeOH (20 mL). Purification by column chromatography, recrystallisation from MeOH/THF, yellow solid, $C_{72}H_{77}F_{13}O_8$, *M* = 1317.36 g/mol, yield: 170 mg (62%), mp. 140 °C, **^{1}H-NMR** ($CDCl_3$, 400 MHz) δ 7.47-7.44 (m, 12H, Ar–*H*), 7.00 (s, 1H, Ar–*H*), 6.99 (s, 1H, Ar–*H*), 6.88 (m, 4H, Ar–*H*), 4.12-4.00 (m, 8H, O*CH$_2$*, –*CH*OH), 3.86-3.82 (m, 2H, –O*CH$_2$*–), 3.78-3.71 (m, 4H, –O*CH$_2$*–, –*CH$_2$*OH), 2.18 (m, 2H, –*CH$_2$*CF_2–), 1.94-1.92 (m, 4H, –OCH_2(*CH$_2$*)$_2$$CH_2CF_2$–), 1.86-1.82 (m, 2H, –$OCH_2$*CH$_2$*–), 1.54-1.52 (m, 2H, –$OCH_2CH_2$*CH$_2$*–), 1.40-1.23 (m, 28H, –*CH$_2$* –), 0.86 (m, 3H, -*CH$_3$*) ppm. **^{19}F-NMR** ($CDCl_3$, 200 MHz,): δ -81.19 (m, 3F, –CF_3), -114.67 (m, 2F, –CF_2CH_2–), -122.28 (m, 2F, –CF_2–), -123.22 (m, 2F, –CF_2–), -123.85 (m, 2F, –CF_2–), -126.49 (m, 2F, –CF_2–) ppm. **HRMS** (m/z): $[M]^+Cl^-$ calcd. for $C_{72}H_{77}F_{13}O_8Cl$, 1351.5105; found, 1351.5269.

2.7.4 Compound E$_3$12/4F6

5-Dodecyloxy-2-(5,5,6,6,7,7,8,8,9,9,10,10,10-tridecafluorodecyl-1-oxy)-1,4-bis{4-[4-(triisopropylsilyloxy)phenylethynyl]phenylethynyl}benzene (14l): Synthesized according to P6 from **7l** (430 mg, 0.53 mmol), **13** (477 mg, 1.27 mmol), $[Pd(PPh_3)_4]$, CuI (4 mg, 0.03 mmol) in NEt_3 (30 mL). Purification by column chromatography (eluent: *n*-hexane/$CHCl_3$ = 3:1), yellow oil, $C_{78}H_{93}F_{13}O_4Si_2$, *M* = 1289.64 g/mol, yield: 100 mg (15%), ^{1}H NMR (400 MHz, CDCl3) δ 7.49 – 7.42 (m, 8H, Ar-*H*), 7.41 – 7.35 (m, 4H, Ar-*H*), 7.00 (s, 1H, Ar-*H*), 6.99 (s, 1H, Ar-*H*), 6.86 – 6.80 (m, 4H, Ar-*H*), 4.07 (t, ^3J(H,H) = 5.3 Hz, 2H, O*CH$_2$*(CH_2)$_2$$CH_2CF_2$), 4.02 (t, ^3J(H,H) = 6.4 Hz, 2H, O*CH$_2$*), 2.27 – 2.08 (m, 2H, *CH$_2$*CF_2), 1.99 – 1.88 (m, 4H, OCH_2(*CH$_2$*)$_2$$CH_2CF_2$), 1.87 – 1.77 (m, 2H, OCH_2*CH$_2$*), 1.62 – 1.46 (m, 2H, OCH_2CH_2*CH$_2$*), 1.40 – 1.18 (m, 22H, $OCH_2CH_2CH_2$(*CH$_2$*)$_8$, Si*CH*), 1.14 – 1.02 (m, 36H, CH*CH$_3$*), 0.85 (t, ^3J(H,H) = 6.3 Hz, 3H, CH_2*CH$_3$*).

5-Dodecyloxy-2-(5,5,6,6,7,7,8,8,9,9,10,10,10-tridecafluorodecyl-1-oxy)1,4-bis[4-(4-hydroxy-phenylethynyl)phenylethynyl]benzene (15l): Synthesized according to P10 from **14l** (100 mg, 0.08 mmol), Bu_4NF (1 M in THF, 0.03 mL, 0.03 mmol) in THF (10 mL). Purification by column chromatography (eluent: $CHCl_3$). Recrystallisation from THF/petroleum ether. Yellowish solid, $C_{60}H_{53}F_{13}O_4$, *M* = 1085.06 g/mol, yield: 80 mg (95%), mp. 200-202 °C, **^{1}H-NMR** (400 MHz, CDCl3) δ 7.49 – 7.44 (m, 8H, Ar–*H*), 7.43 – 7.38 (m, 4H, Ar–*H*), 7.00 (s, 1H, Ar–*H*), 6.99 (s, 1H, Ar–*H*), 6.83 – 6.76 (m, 4H, Ar–*H*), 4.82 – 4.80 (m, 2H, –O*H*), 4.07 (t, ^3J(H,H) = 5.4 Hz, 2H, –O*CH$_2$*(CH_2)$_2$$CH_2CF_2$–), 4.02 (t, ^3J(H,H) = 6.4 Hz, 2H, –O*CH$_2$*–), 2.26 – 2.00 (m, 2H, –*CH$_2$*CF_2–), 2.07 – 1.88 (m, 4H, –OCH_2(*CH$_2$*)$_2$$CH_2CF_2$–), 1.88 – 1.78 (m, 2H, –OCH_2*CH$_2$*–), 1.58 – 1.44 (m, 2H, –OCH_2CH_2*CH$_2$*–), 1.41 – 1.16 (m, 16H, –$OCH_2CH_2CH_2$(*CH$_2$*)$_8$–), 0.85 (t, ^3J(H,H) = 6.8 Hz, 3H, –CH_2*CH$_3$*) ppm.

5-Dodecyl-2-(5,5,6,6,7,7,8,8,9,9,10,10,10-tridecafluorodecyl-1-oxy)-1,4-bis{4-[4-(11,12-di-hydroxy-3,6,9-trioxadodecyl-1-oxy)phenylethynyl]phenylethynyl}benzene (E$_3$12/4F6): Synthesized according to P5 from **15l** (48 mg, 0.04 mmol), **17d** (28 mg, 0.10 mmol), K_2CO_3 (61 mg, 0.44 mmol) in DMF (30 mL). Purification by column chromatography (eluent: EtOAc), recrystallisation from THF/MeOH. Yellow solid, $C_{78}H_{89}F_{13}O_{14}$, *M* = 1497.53 g/mol, yield: 24 mg (36%), mp. 99 °C, **^{1}H-NMR** (400 MHz, CDCl3) δ 7.50 – 7.39 (m, 12H, Ar–*H*), 7.00 (s, 1H, Ar–*H*), 6.99 (s, 1H, Ar–*H*), 6.91 – 6.85 (m, 4H, Ar–*H*), 4.21 – 4.11 (m, 4H), 4.10 – 3.95 (m, 4H, –O*CH$_2$*CH_2CH_2–), 3.90 – 3.78 (m, 6H), 3.77 – 3.47 (m, 24H), 2.29 – 2.04 (m, 2H, –*CH$_2$*CF_2–), 2.01 – 1.77 (m, 6H, –OCH_2CH_2*CH$_2$*–, –*CH$_2$*CH_2CF_2–), 1.58 – 1.43 (m, 2H, –OCH_2CH_2*CH$_2$*–), 1.41 – 1.18 (m, 16H, –$OCH_2CH_2CH_2$(C*H$_2$*)$_8$–), 0.85 (t, ^3J(H,H) = 6.6 Hz,

3H, –CH_2CH_3) ppm. **^{19}F-NMR** (376 MHz, CDCl3) δ -80.78 (t, ^3J(C,F) = 9.9 Hz), -114.29 (s), -121.89 (s), -122.85 (s), -123.45 (s), -126.11 (s) ppm. **HRMS** (m/z): $[M]^+Cl^-$ calcd. for $C_{78}H_{89}F_{13}O_{14}Cl$, 1531.5728; found, 1531.5516.

2.8 Synthesis of compound E$_3$12

2,5-Didodecyloxy-1,4-bis{4-[4-(11,12-dihydroxy-3,6,9-trioxadodecyl-1-oxy)phenyl-ethynyl]phenylethynyl}benzene (B$_3$12): Synthesized according to P5 from **15n** (70 mg, 0.08 mmol), **17d** (50 mg, 0.18 mmol), K_2CO_3 (110 mg, 0.80 mmol) in DMF (30 mL). Purification by column chromatography (eluent: $CHCl_3$/MeOH = 10:0.3). Yellow solid, $C_{80}H_{106}O_{14}$, *M* = 1291.71 g/mol, yield: 52 mg (51%), mp. 131 °C, **^{1}H-NMR** (500 MHz, $CDCl_3$) δ 7.50 – 7.40 (m, 12H, Ar–*H*), 6.99 (s, 2H, Ar–*H*), 6.93 – 6.84 (m, 4H, Ar–*H*), 4.19 – 4.13 (m, 4H), 4.02 (t, ^3J(H,H) = 6.4 Hz, 4H, –$OCH_2CH_2CH_2$–), 3.90 – 3.80 (m, 6H), 3.74 – 3.58 (m, 22H), 3.56 (dd, ^2J(H,H) = 10.1, ^3J(H,H) = 6.3 Hz, 2H), 1.88 – 1.78 (m, 4H, –$OCH_2CH_2CH_2$–), 1.56 – 1.46 (m, 4H, –$OCH_2CH_2CH_2$–), 1.42 – 1.16 (m, 32H, –$OCH_2CH_2CH_2(CH_2)_8$–), 0.85 (t, ^3J(H,H) = 6.9 Hz, 6H, –CH_2CH_3) ppm. **HRMS** (m/z): $[M]^+Cl^-$ calcd. for $C_{80}H_{106}O_{14}Cl$, 1325.7266; found, 1325.7209.

3. Representative NMR Spectra

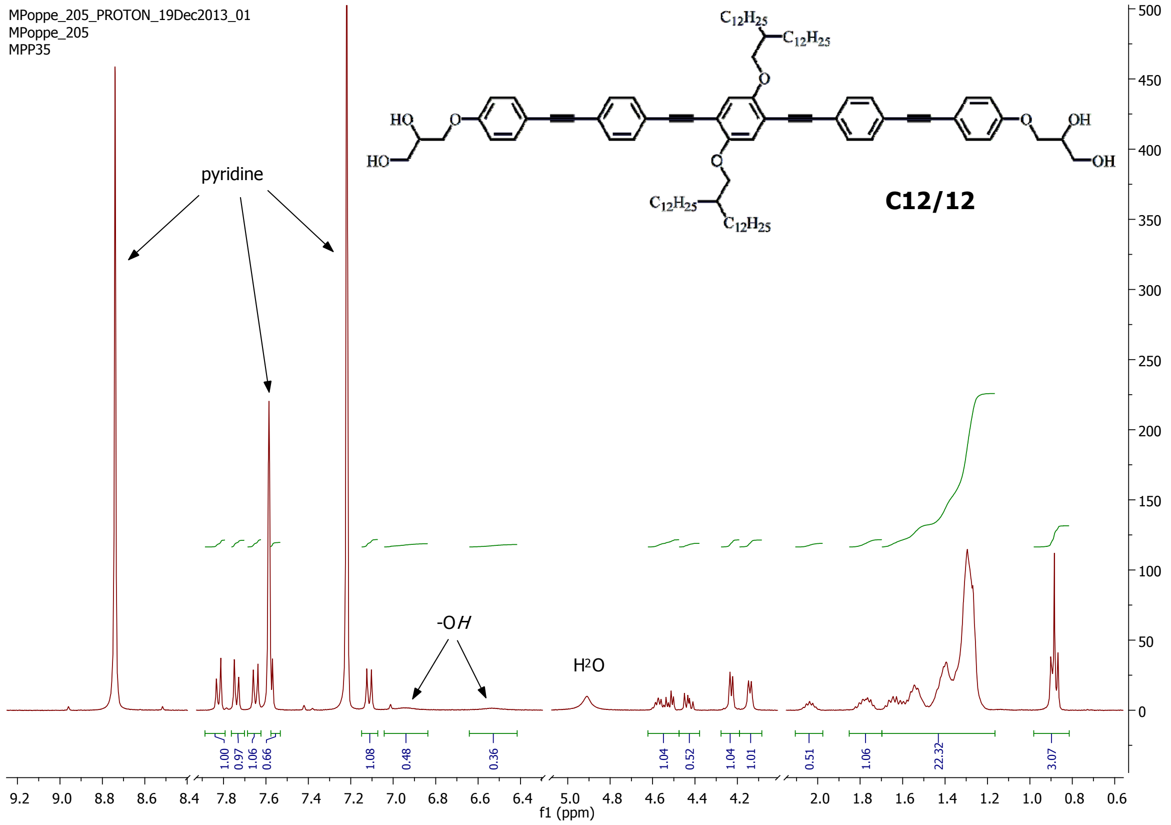

Figure S9. ^{1}H-NMR of compound **C12/12** (400 MHz, pyridine-d_5).

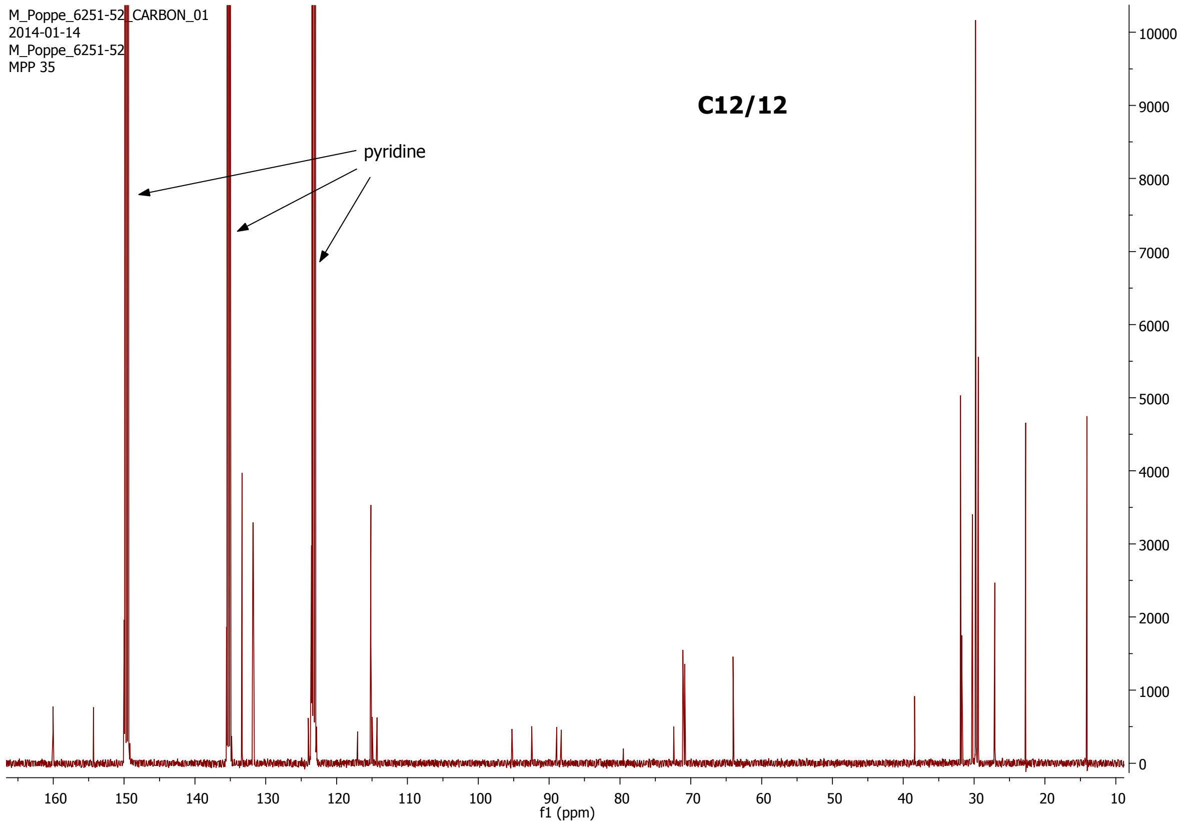

Figure S10. ^{13}C-NMR of compound **C12/12** (126 MHz, pyridine-d_5).

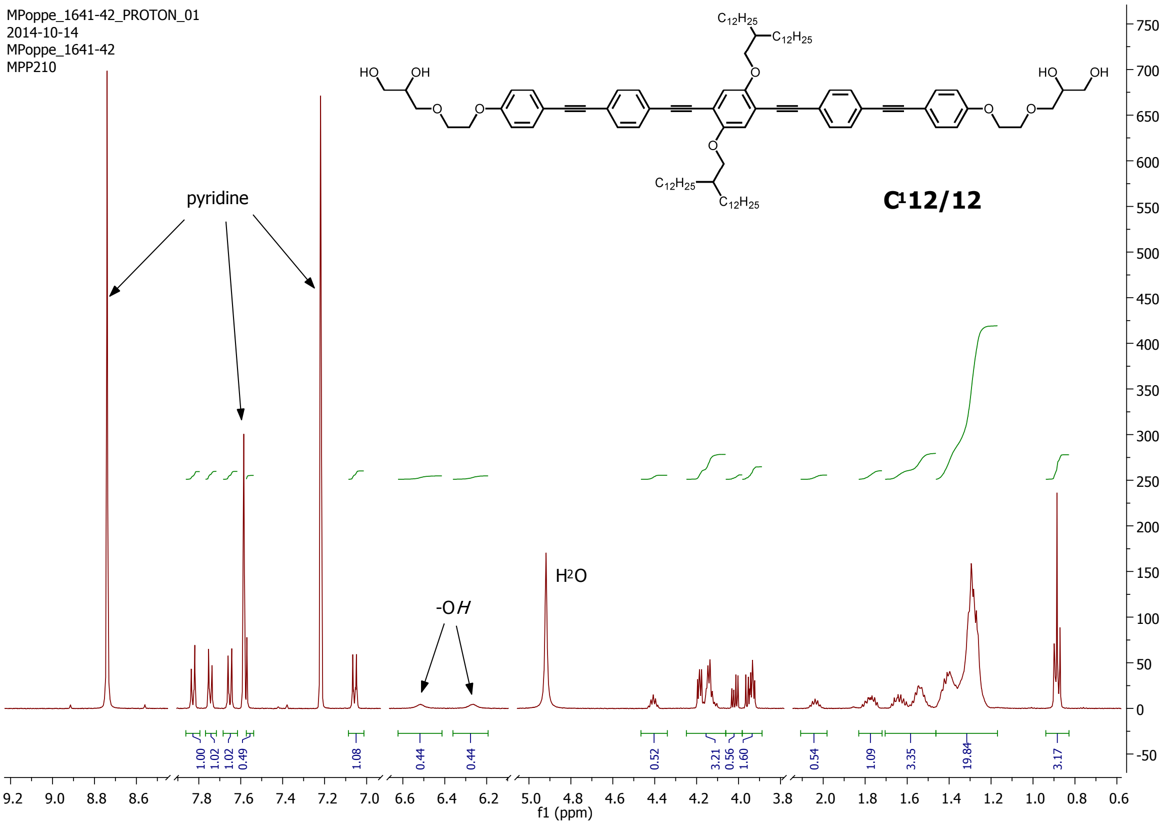

Figure S11. ^{1}H-NMR of compound **$C_1$12/12** (500 MHz, pyridine-d_5).

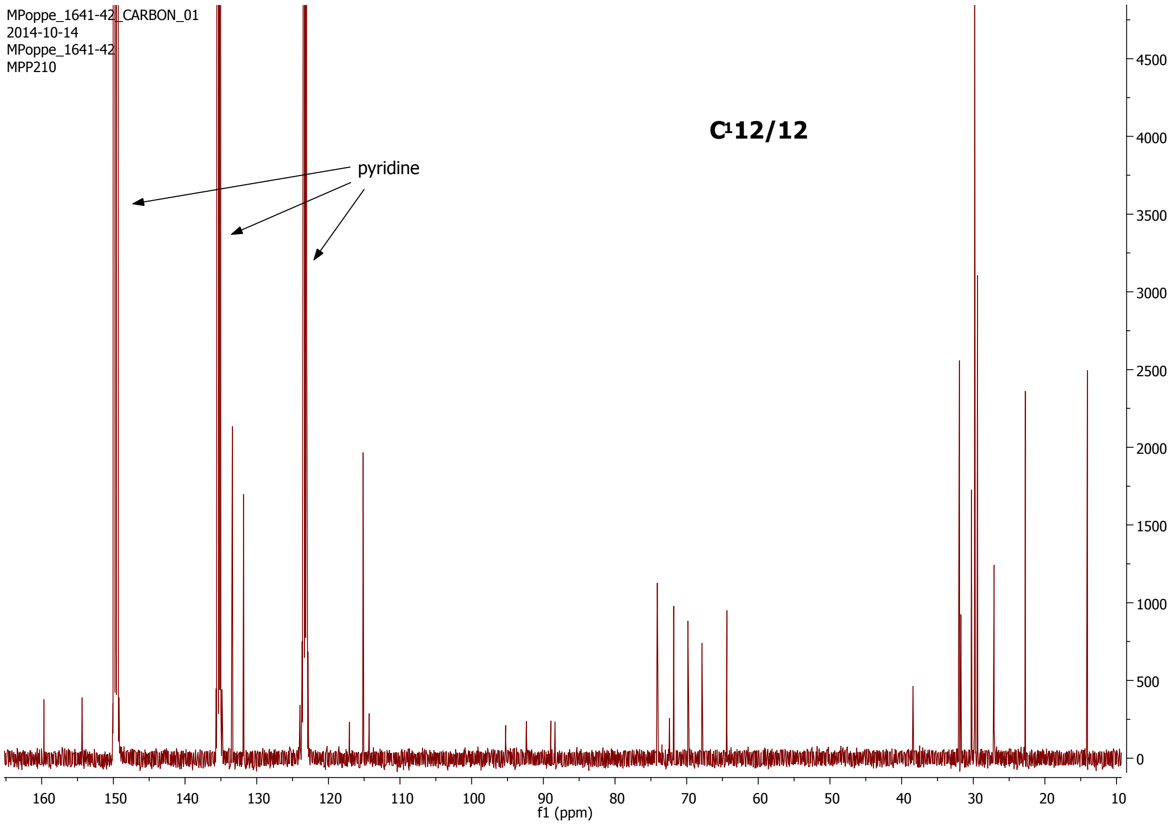

Figure S12. ^{13}C-NMR of compound **$C_1$12/12** (126 MHz, pyridine-d_5).

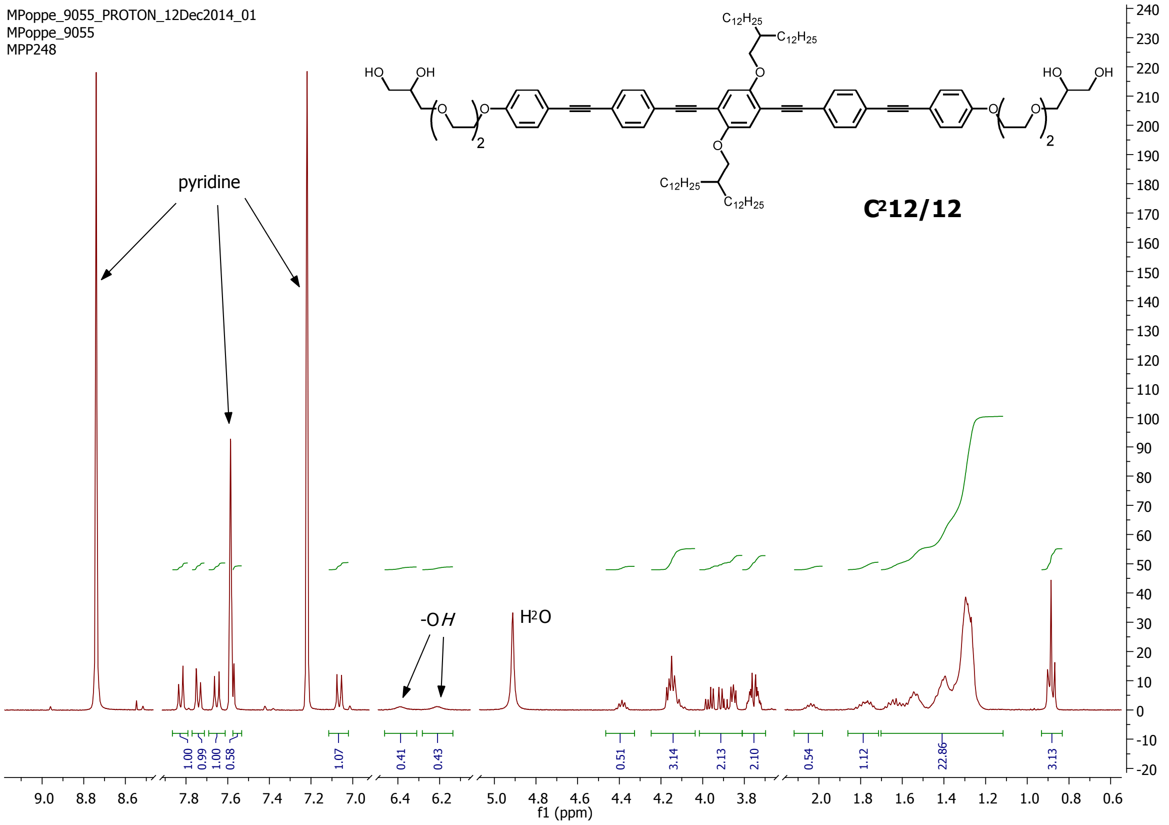

Figure S13. ^{1}H-NMR of compound **C$_2$12/12** (500 MHz, pyridine-d$_5$).

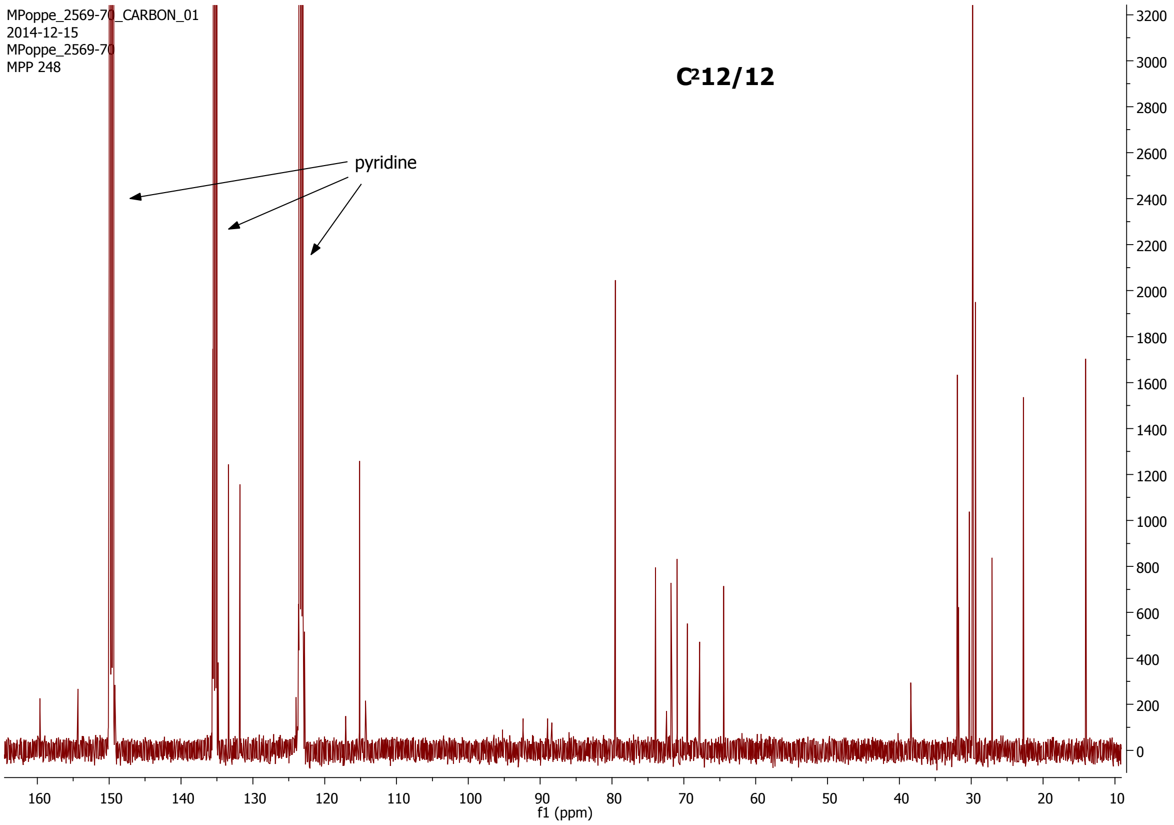

Figure S14. ^{13}C-NMR of compound **C$_2$12/12** (126 MHz, pyridine-d$_5$).

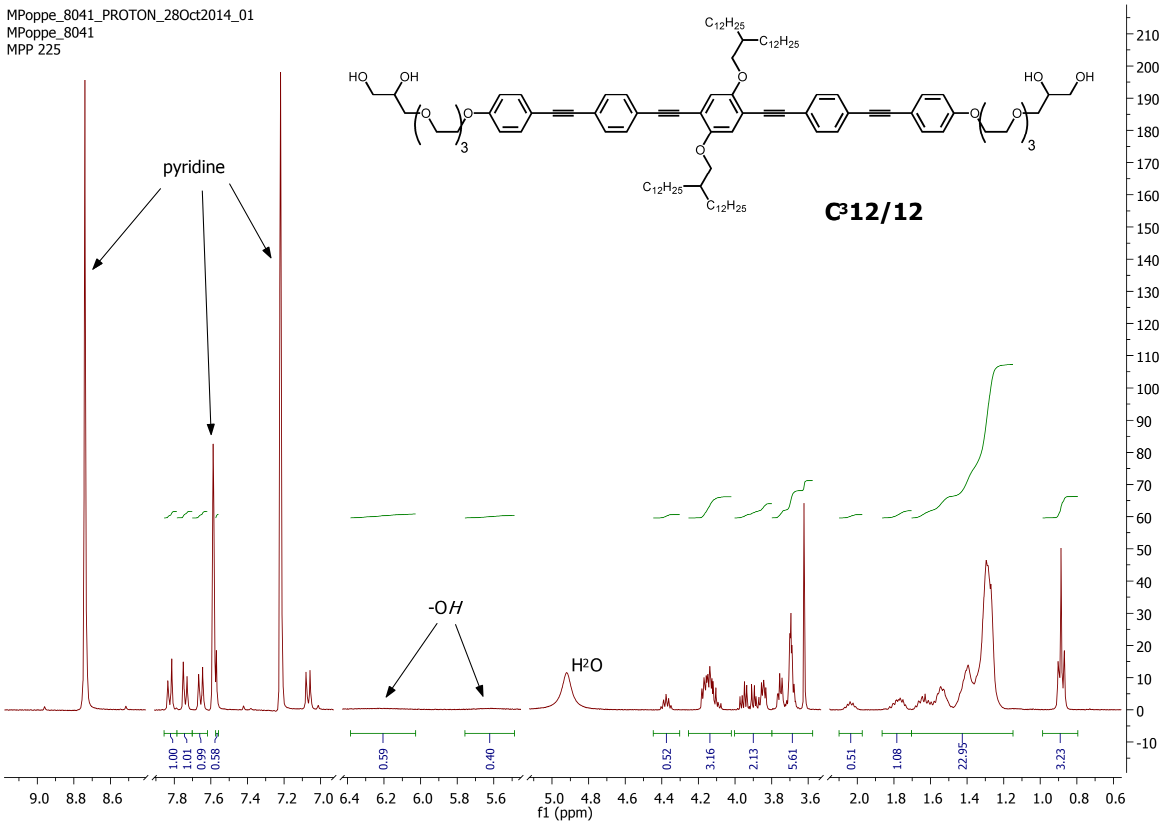

Figure S15. ^{1}H-NMR of compound **C$_{3}$12/12** (400 MHz, pyridine-d$_{5}$).

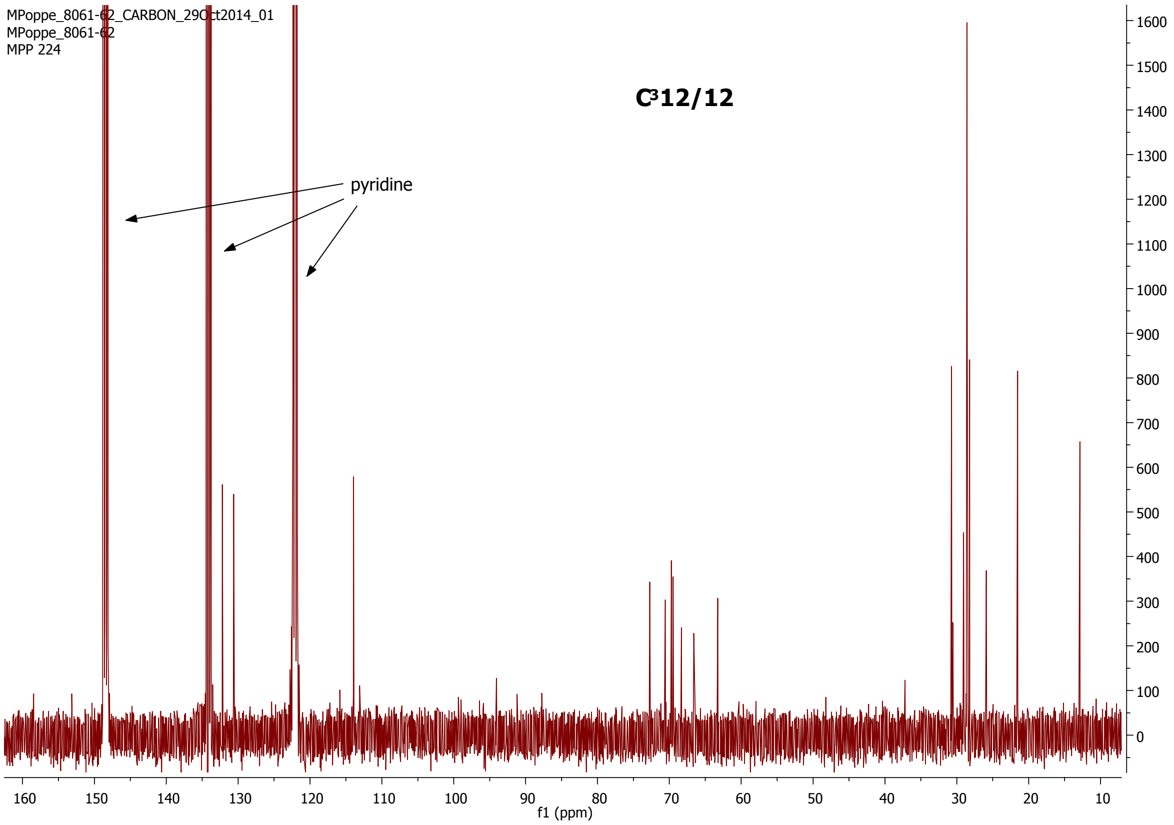

Figure S16. ^{13}C-NMR of compound **C$_{3}$12/12** (101 MHz, pyridine-d$_{5}$).

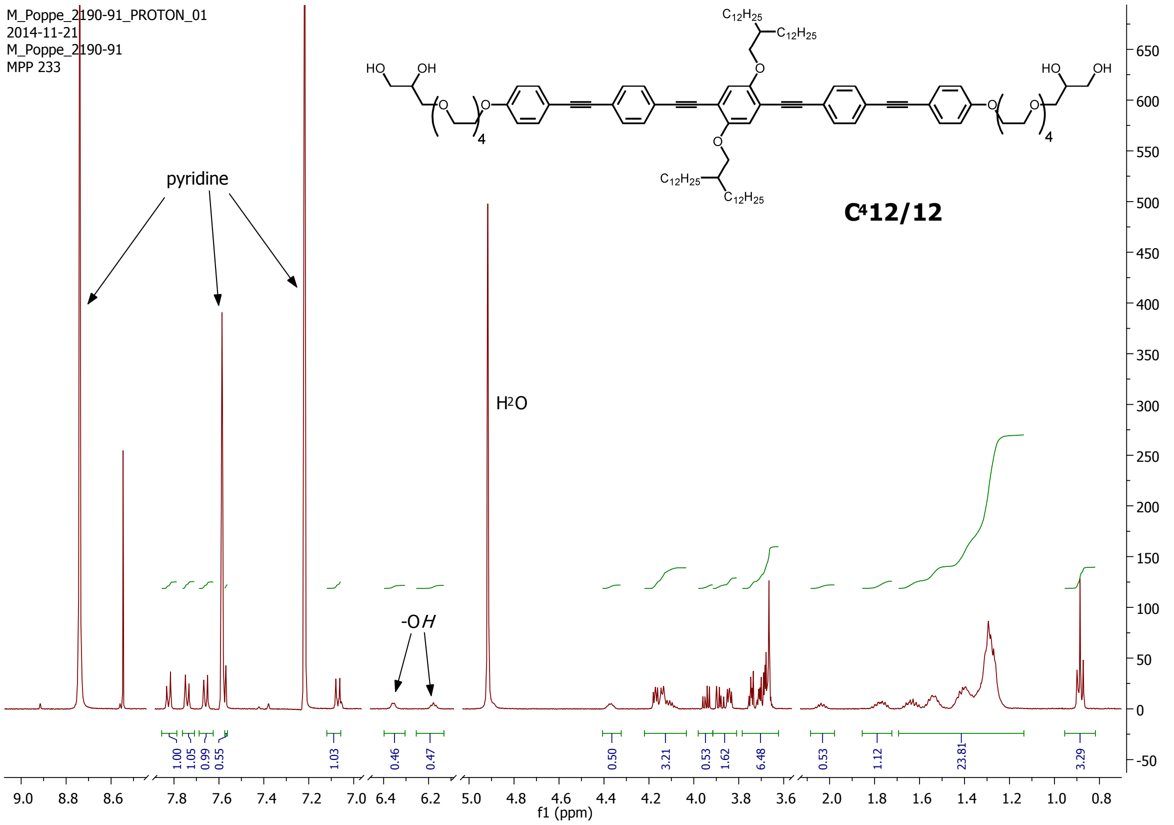

Figure S17. ^{1}H-NMR of compound **C$_4$12/12** (500 MHz, pyridine-d$_5$).

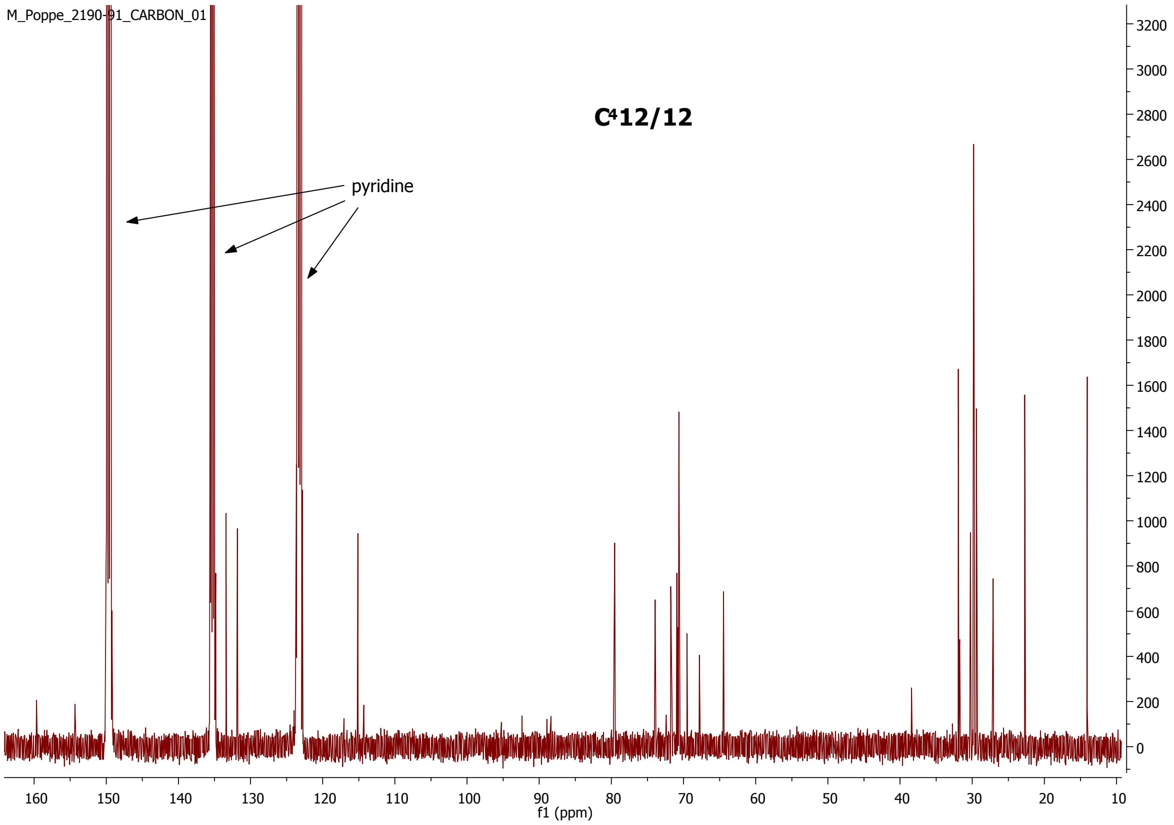

Figure S18. ^{13}C-NMR of compound **C$_4$12/12** (126 MHz, pyridine-d$_5$).

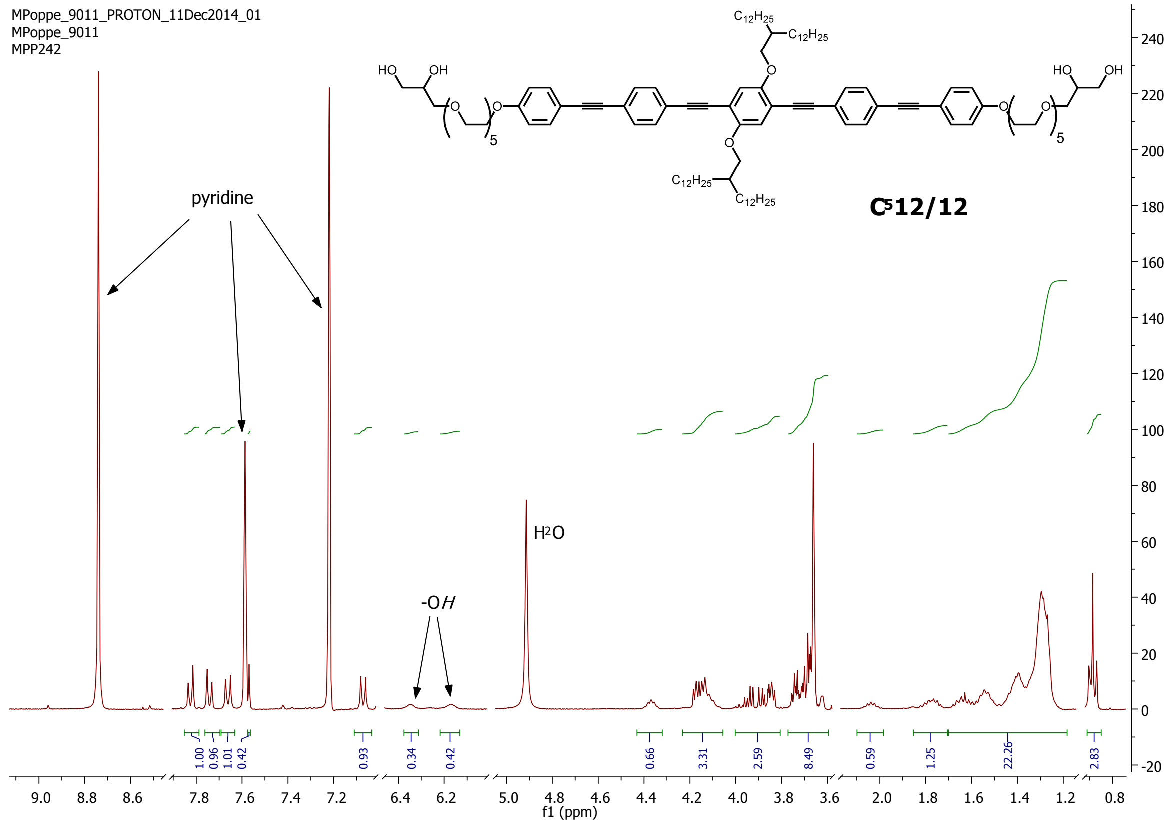

Figure S19. ^{1}H-NMR of compound **$C_5$12/12** (500 MHz, pyridine-d_5).

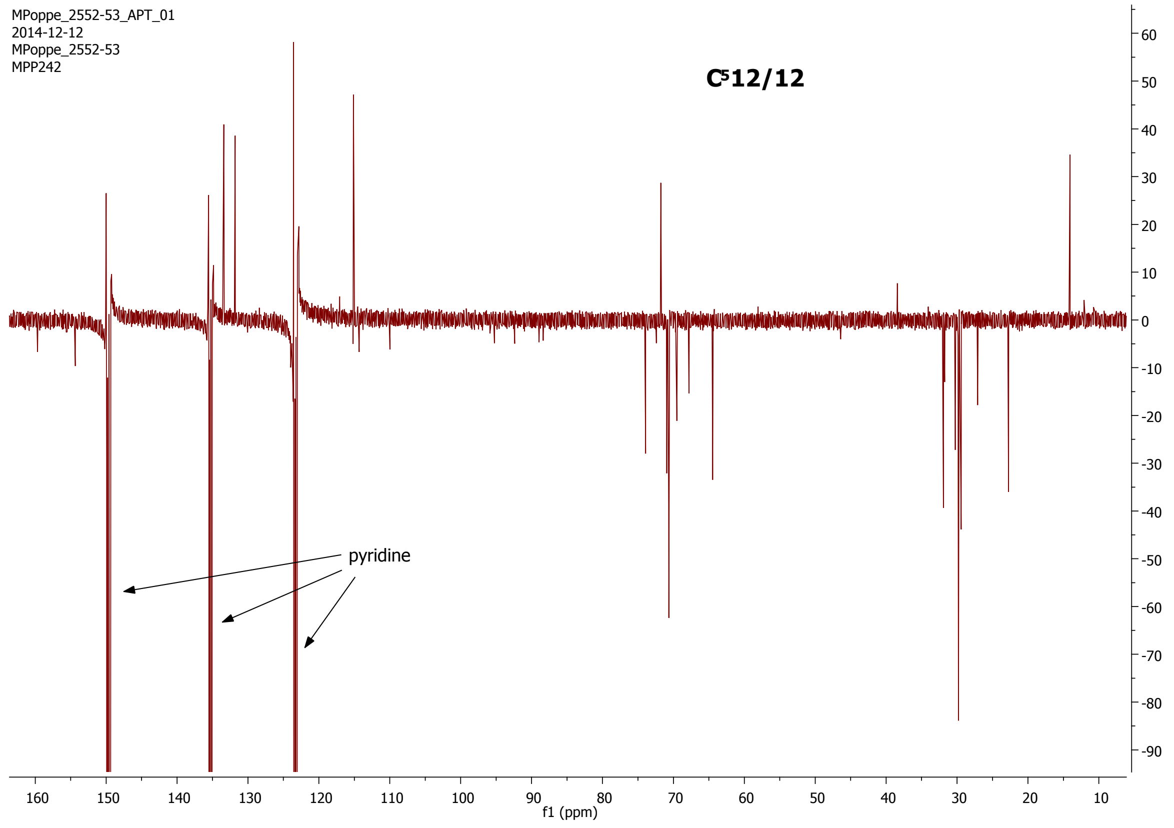

Figure S20. ^{13}C-NMR (APT) of compound **$C_5$12/12** (126 MHz, pyridine-d_5).

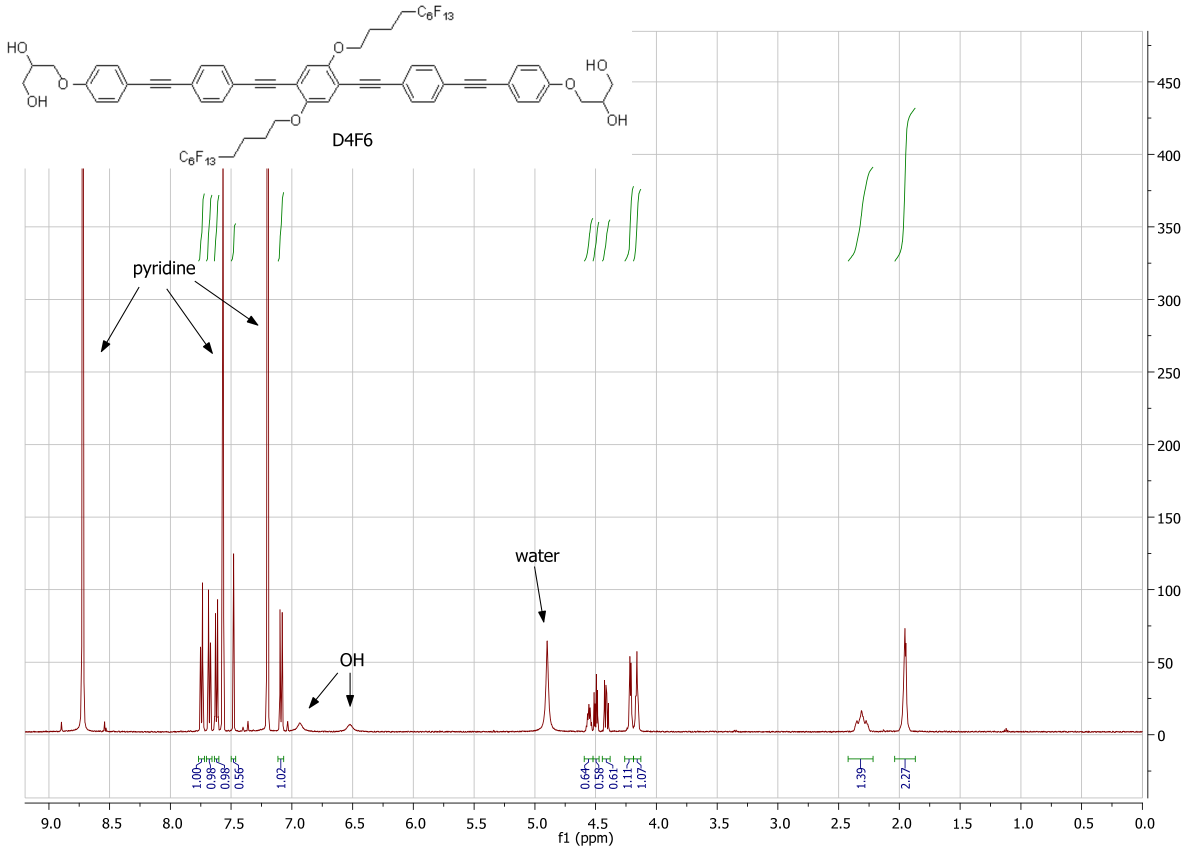

Figure S21. ^{1}H-NMR of compound **D4F6** (400 MHz, pyridine-d_5).

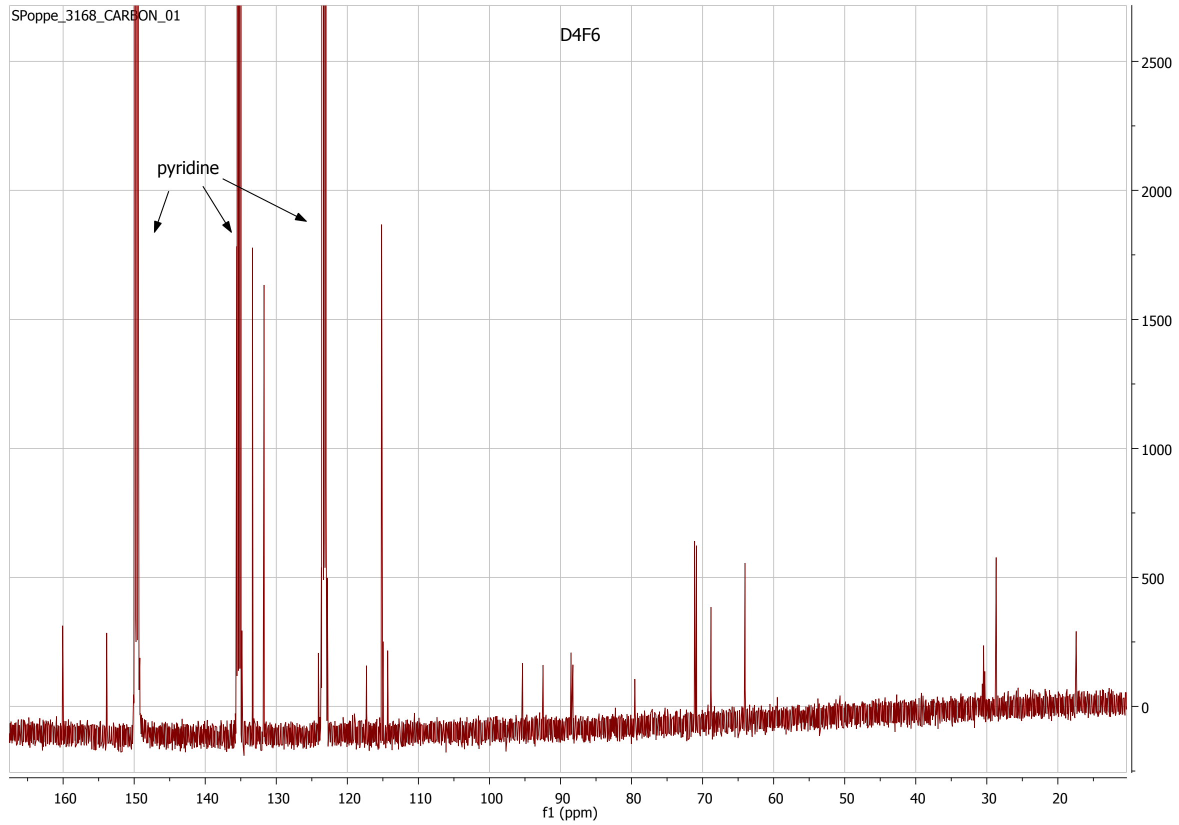

Figure S22. ^{13}C-NMR of compound **D4F6** (100 MHz, pyridine-d_5).

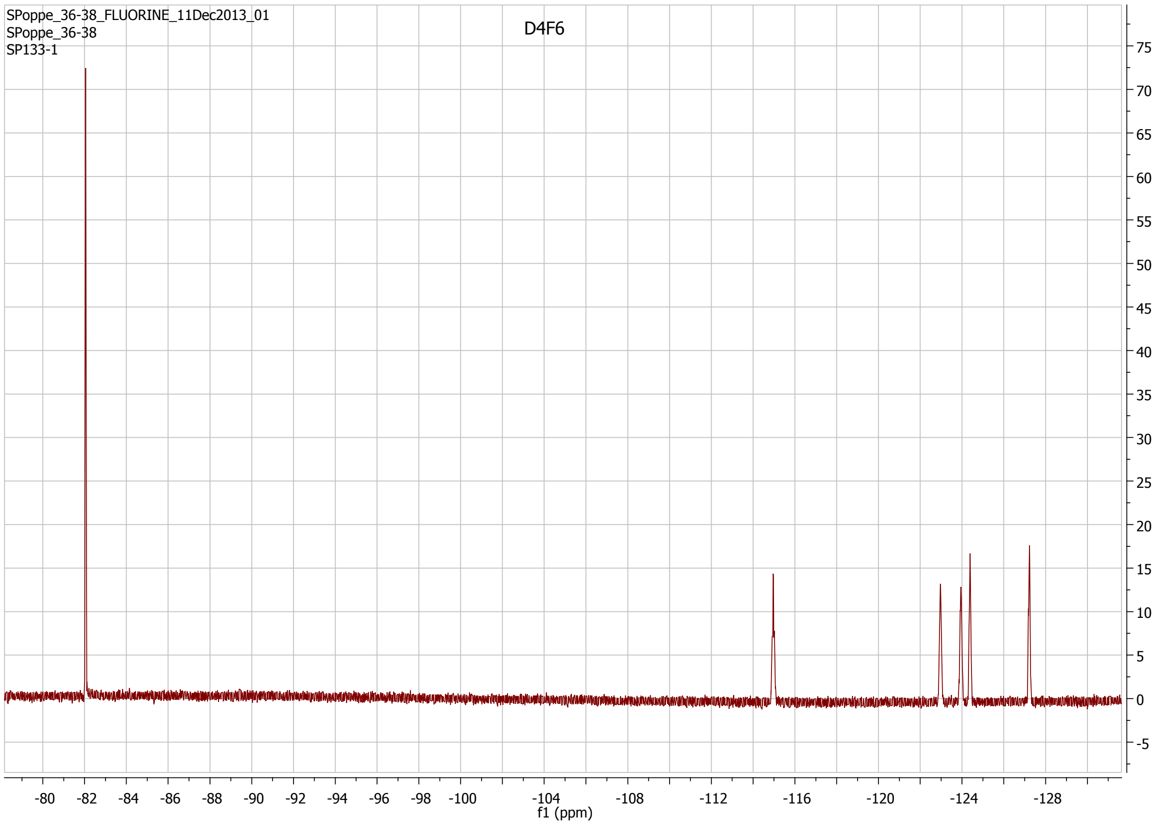

Figure S23. ^{19}F-NMR of compound **D4F6** (376 MHz, pyridine-d_5).

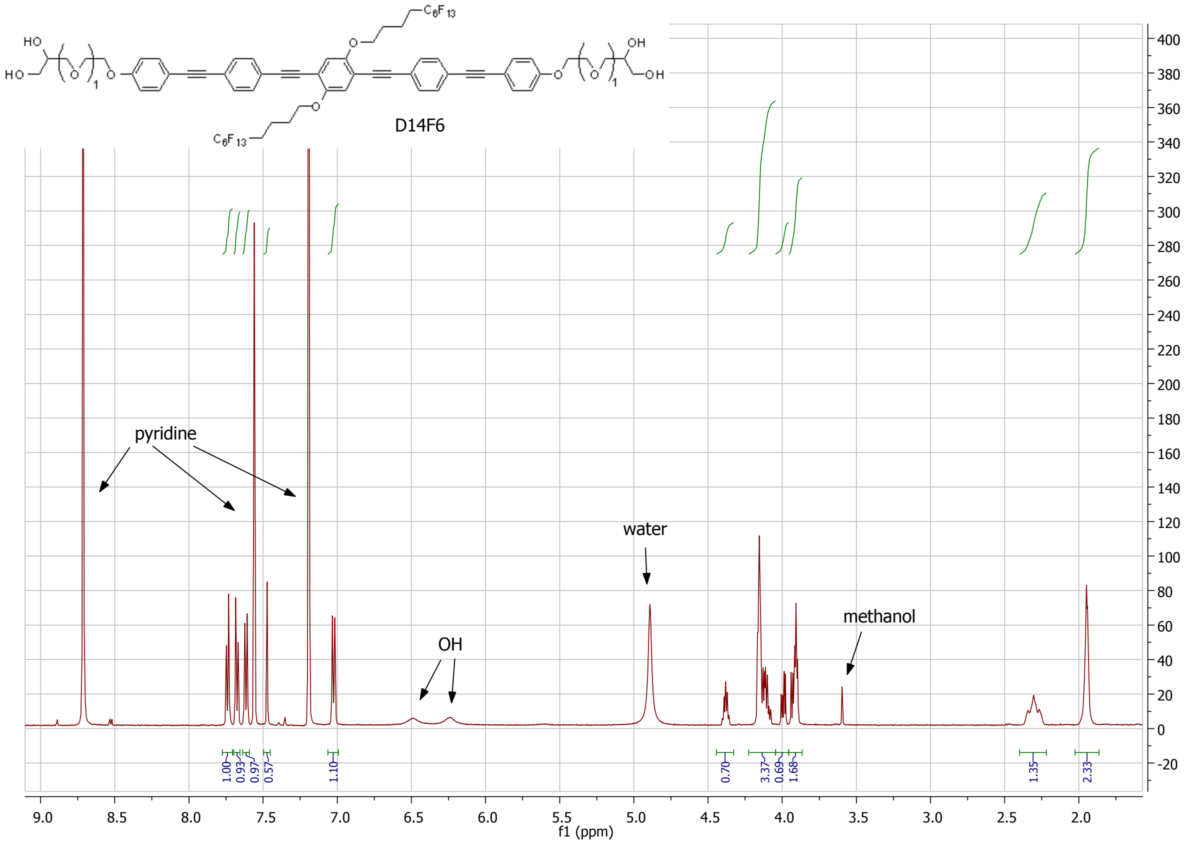

Figure S24. ^{1}H-NMR of compound **$D_1$4F6** (400 MHz, pyridine-d_5).

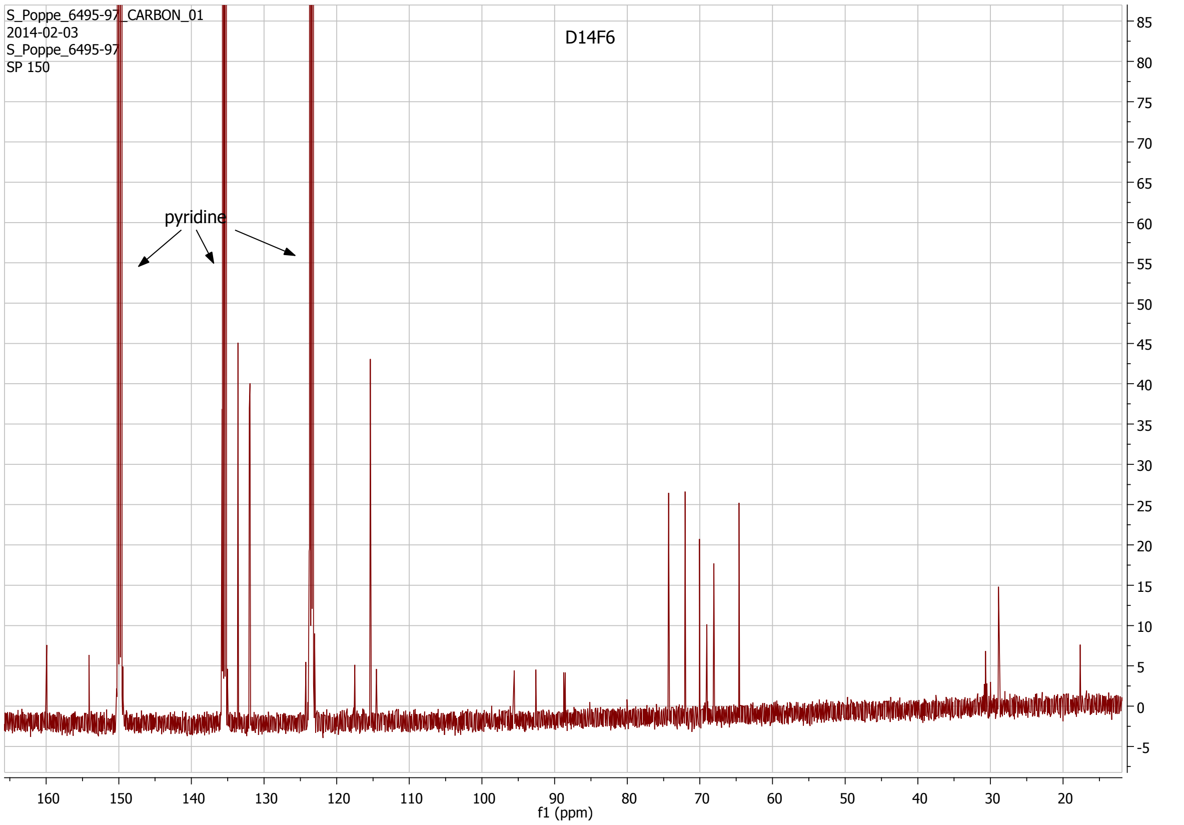

Figure S25. ^{13}C-NMR of compound $\mathbf{D_14F6}$ (100 MHz, pyridine-d_5).

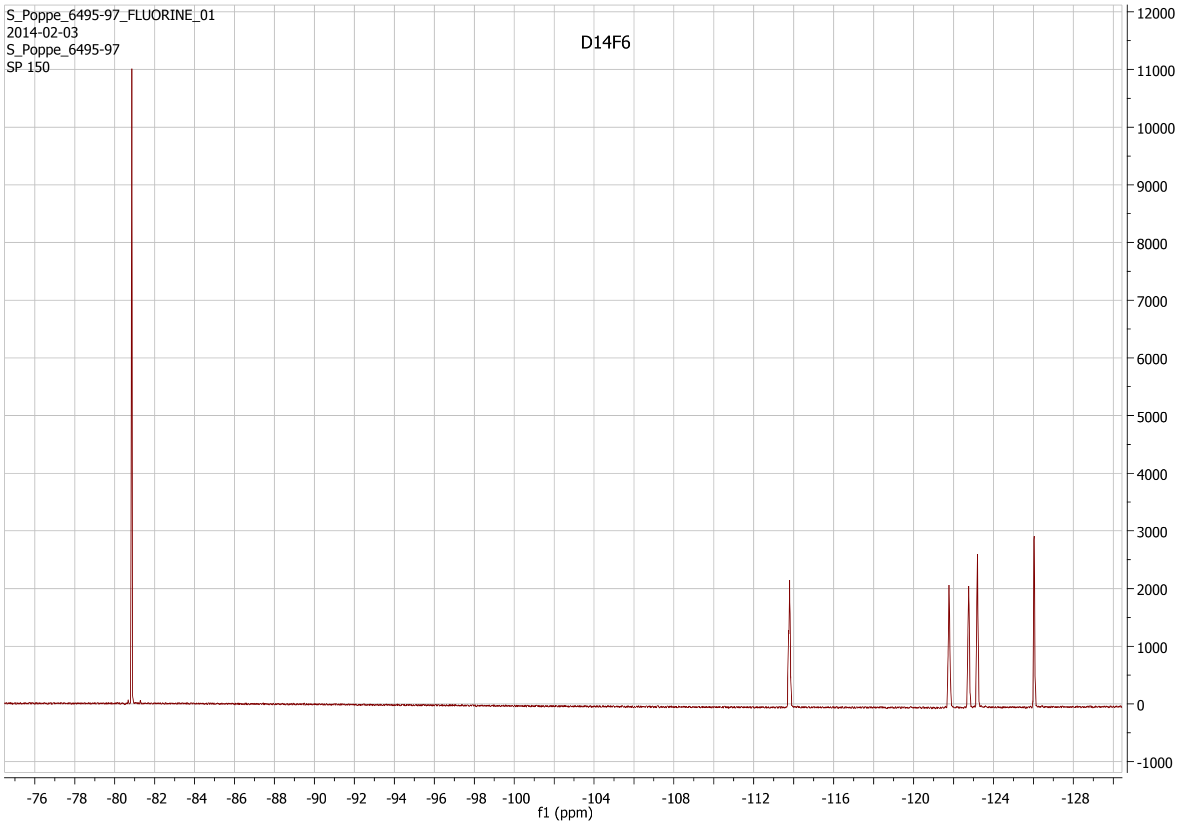

Figure S26. ^{19}F-NMR of compound $\mathbf{D_14F6}$ (470 MHz, pyridine-d_5).

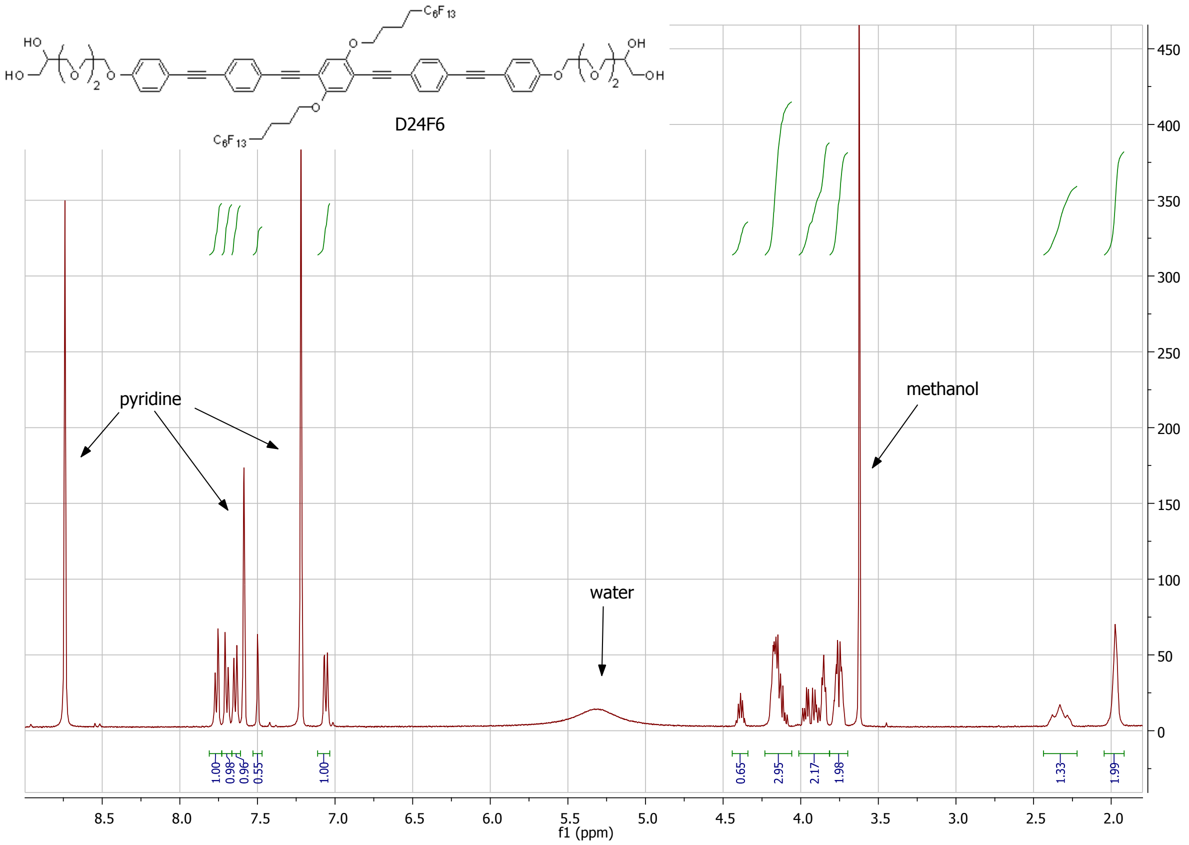

Figure S27. ^{1}H-NMR of compound **D$_2$4F6** (400 MHz, pyridine-d5).

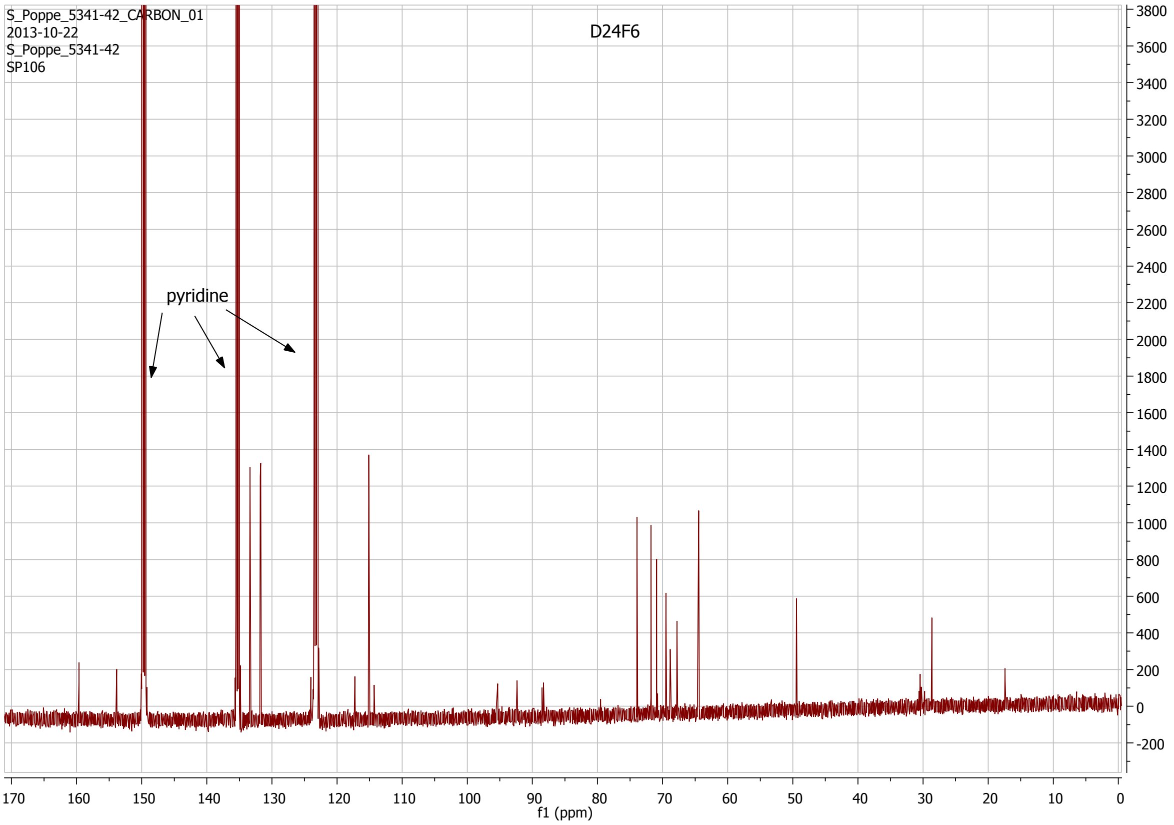

Figure S28. ^{13}C-NMR of compound **D$_2$4F6** (100 MHz, pyridine-d$_5$).

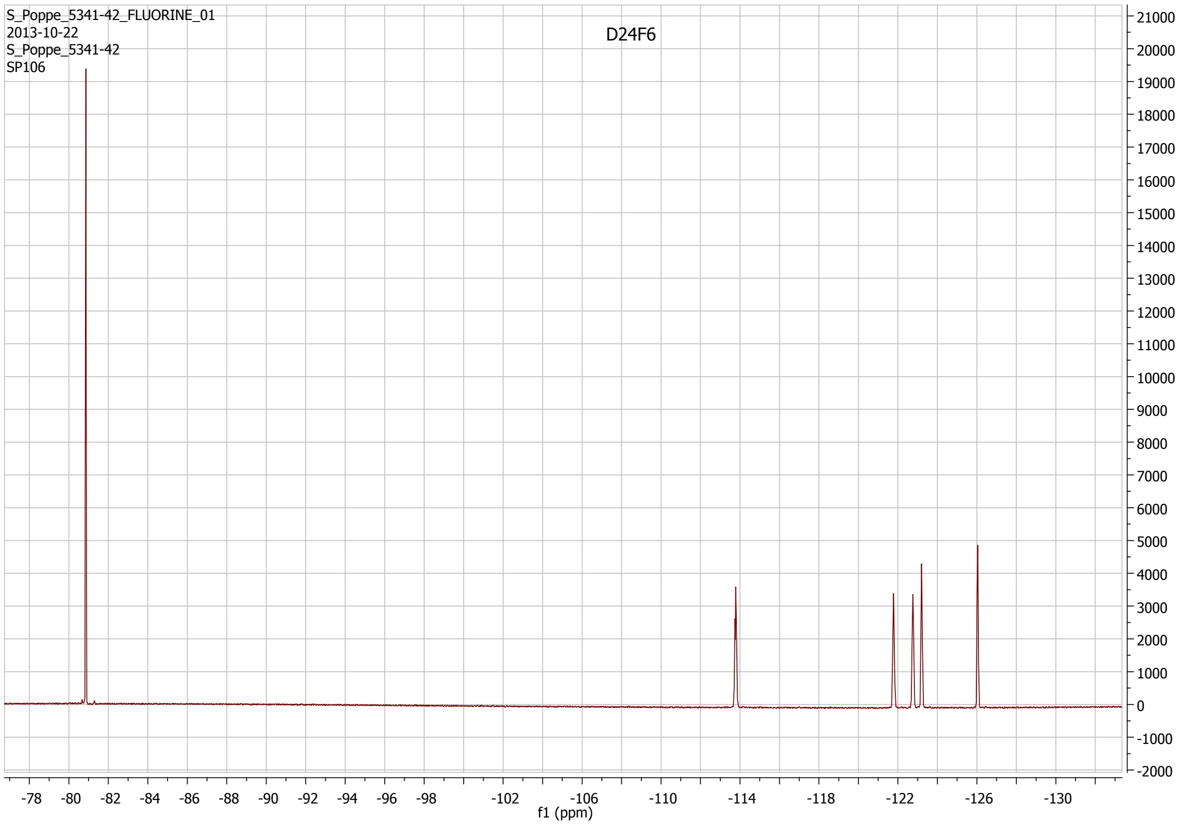

Figure S29. ^{19}F-NMR of compound **D_24F6** (470 MHz, pyridine-d_5).

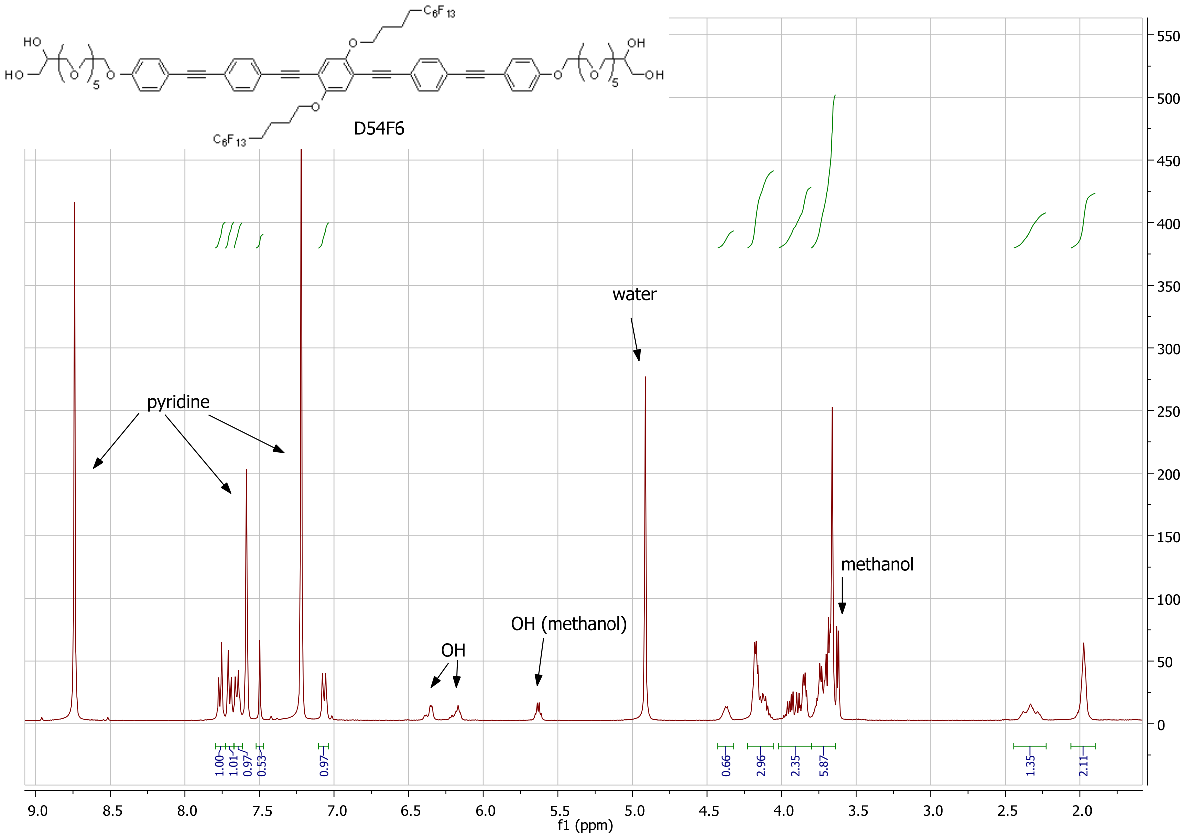

Figure S28. ^{1}H-NMR of compound **D_54F6** (400 MHz, pyridine-d_5).

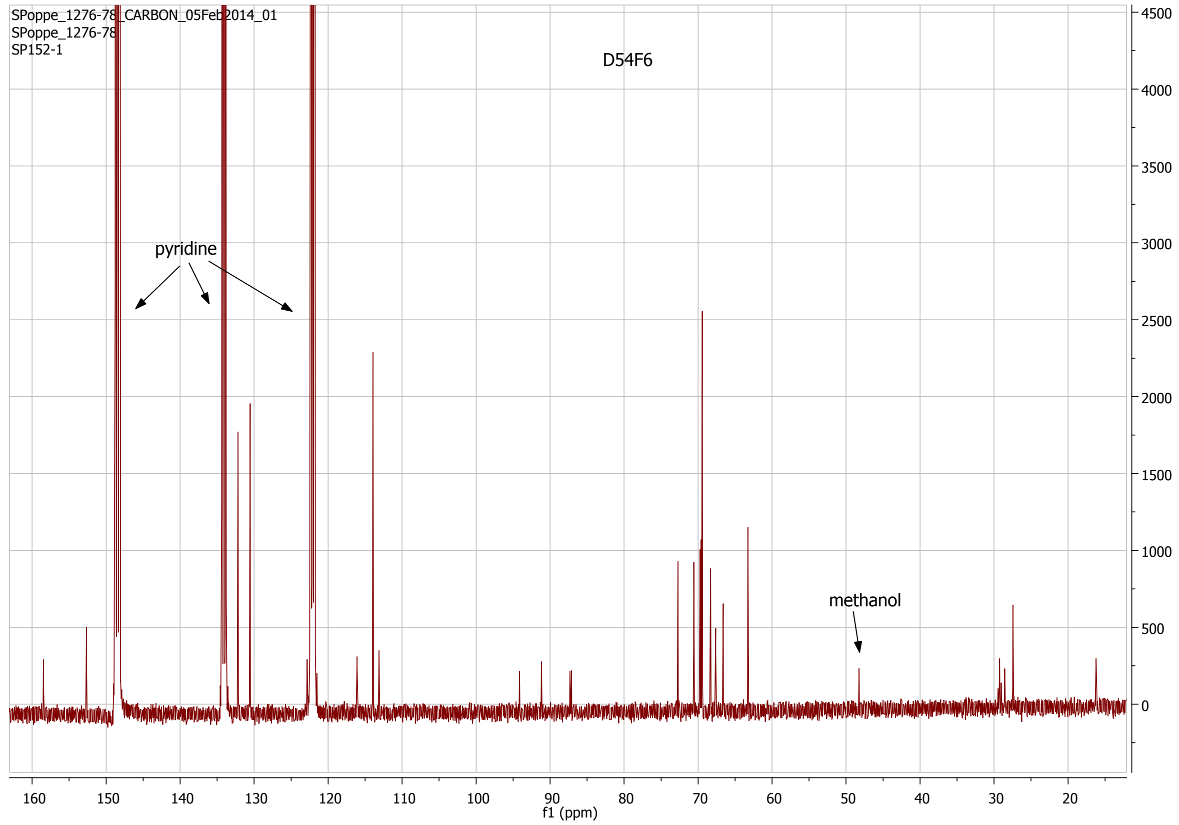

Figure S29. ^{13}C-NMR of compound **D$_5$4F6** (100 MHz, pyridine-d$_5$).

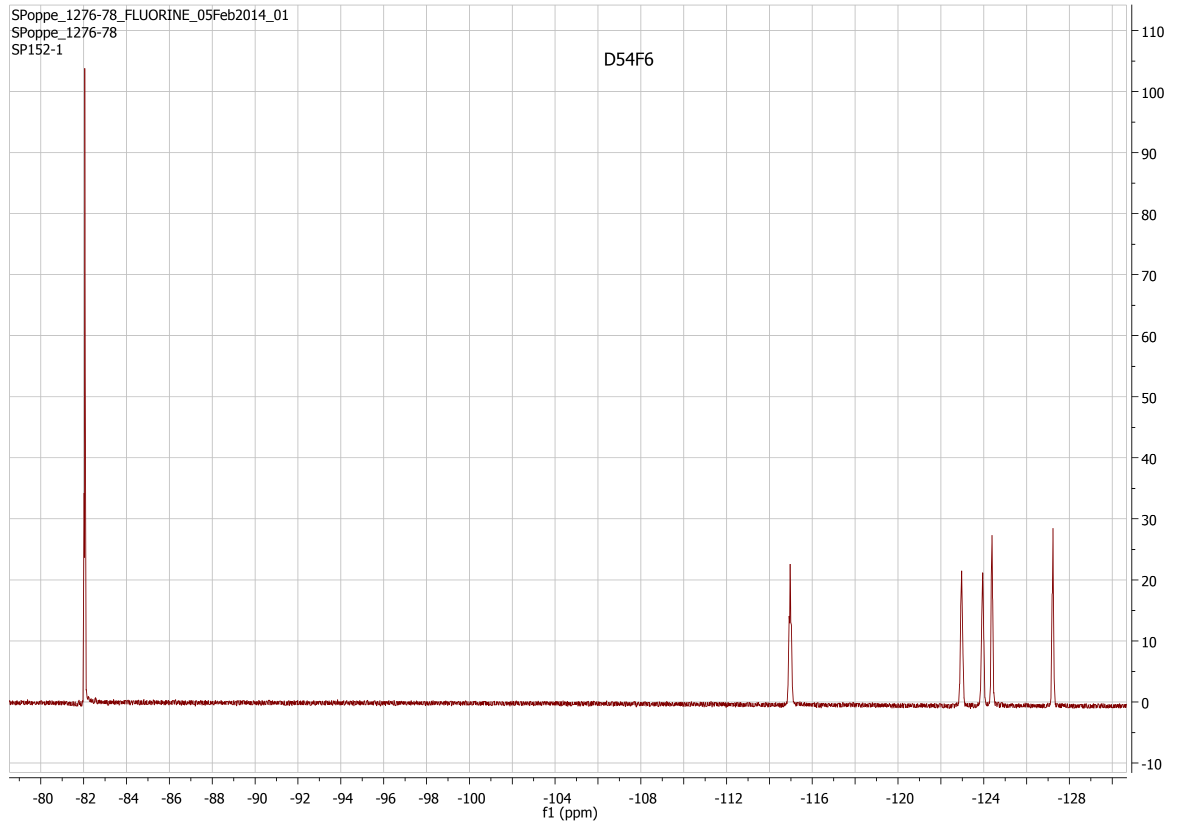

Figure S30. ^{19}F-NMR of compound **D$_5$4F6** (376 MHz, pyridine-d$_5$).

4. References

[S1] Immirzi, A.; Perini, B. Prediction of Density in Organic Crystals. *Acta Cryst. Sect. A* **1977**, *33*, 216–218.

[S2] Kitaigorodski, A. I. *Molekülkristalle*, Akademieverlag: Berlin, Germany, 1979.

[S3] Werner, S.; Ebert, H.; Lechner, B.-D.; Lange, F.; Achilles, A.; Bärenwald, R.; Poppe, S.; Blume, A.; Saalwächter, K.; Tschierske, C.; Bacia, K., Dendritic domains with hexagonal symmetry formed by X-shaped bolapolyphiles in lipid membranes. *Chem. Eur. J.* **2015**, *21*, 8840−8850.

[S4] R. H. Pawle, A. Agarwal, S. Malveira, Z. C. Smith and S. W. Thomas, *Macromolecules* **2014**, *47*, 2250–2256.

[S5] Poppe, M.; Chen, C.; Liu, F.; Prehm, M.; Poppe, S.; Tschierske, C. Emergence of tilt in square honeycomb liquid crystals. *Soft Matter* **2017**, *13*, 4676-4680.

[S6] Pokholenko, O.; Gissot, A.; Vialet, B.; Bathany, K.; Thiéry A.; Barthélémy P. Lipid oligonucleotide conjugates as responsive nanomaterials for drug delivery. *J. Mater. Chem. B* **2013**, *1*, 5329-5334.

[S7] Krapcho, A. P.; Weimaster, J. F.; Eldridge, J. M.; Jahngen Jr, E. G. E. ; Loves A. J.; Stephens, W. P. Synthetic applications and mechanism studies of the decarboxylations of geminal diesters and related systems effected in dimethyl sulfoxide by water and/or by water with added salts. *J. Org. Chem.* **1978**, *43*, 138-147.

[S8] Nystrom R. F.; Brown W. G. Reduction of Organic Compounds by Lithium Aluminium Hydride. I. Aldehydes, Ketones, Esters, Acid Chlorides and Acid Anhydrides. *J. Am. Chem. Soc.* **1947**, *69*, 1197-1199.

[S9] Dakka G.; Sasson, Y. Selective hydrobromination of branched alcohols using phase transfer catalysis. *Tetrahedron Lett.* **1987**, *28*, 1223-1224.

[S10] Williamson, A. Theory of Aetherification. *Philos. Mag.* **1850** *3*, 350-356.

[S11] Sonogashira, K.; Tohda, Y.; Hagihara, N. A convenient synthesis of acetylenes: catalytic substitutions of acetylenic hydrogen with bromoalkenes, iodoarenes and bromopyridines. *Tetrahedron Lett.* **1975**, *50*, 4467–4470.

[S12] Le Huérou, Y.; Doyon, J.; Grée, R. L. Stereocontrolled Synthesis of Key Advanced Intermediates toward Simplified Acetogenin Analogues. *J. Org. Chem.* **1999**, *64*, 6782–6790.

[S13] Tamiakia, H.; Ogawaa, K.; Enomotob, K.; Takia, K.; Hottab, A.; Tomaa, K. Supramolecular gelation of alcohol and water by synthetic amphiphilic gallic acid derivatives. *Tetrahedron* **2010**, *66*, 1661–1666.

[S14] Bird, P. R., Chadha, J. S. The Synthesis of Diacyl-L-Glycerol Bromohydrins. *Tetrahedron Lett*. **1966**, *38*, 4541–4546.

[S15] Cheng, J., Hacksell, U., Doyle Daves, G. Facile Synthesis of 2´-Deoxy-3´-kezo- and 2´-Deoxypseudouridine Derivatives and Analogues. Palladium(II)-Mediated Coupling reactions of Furanoid Glycals. *J. Org. Chem*. **1986**, *51*, 3093–3098.

[S16] van Rijsbergen, R.; Anteunis, M.-J.; de Bruyn, A. Selective Removal of the Isopropylidene Group in 4-O-Protected 1,6-Anhydro-2,3-O-Isopropylidene-β-D-Mannopyranose and the Conformational Impact of it. *J. Carbohydr. Chem.* **2006**, *2*, 395–404.

[S17] Liu, F.; Kieffer, R.; Zeng, X.; Pelz, K.; Prehm, M.; Ungar, G.; Tschierske, C. Arrays of giant octagonal and square cylinders by liquid crystalline self-assembly of X-shaped polyphilic molecules. *Nat. Commun.* **2012**, *3*, 1104.
